# “Naked Nickel”-Catalyzed Amination of Heteroaryl Bromides

**DOI:** 10.1021/acs.orglett.4c01738

**Published:** 2024-07-05

**Authors:** Rakan Saeb, Bryan Boulenger, Josep Cornella

**Affiliations:** Max-Planck-Institut für Kohlenforschung, Department of Organometallic Chemistry, Kaiser-Wilhelm-Platz 1, 45470 Mülheim an der Ruhr, North Rhine-Westphalia, Germany

## Abstract

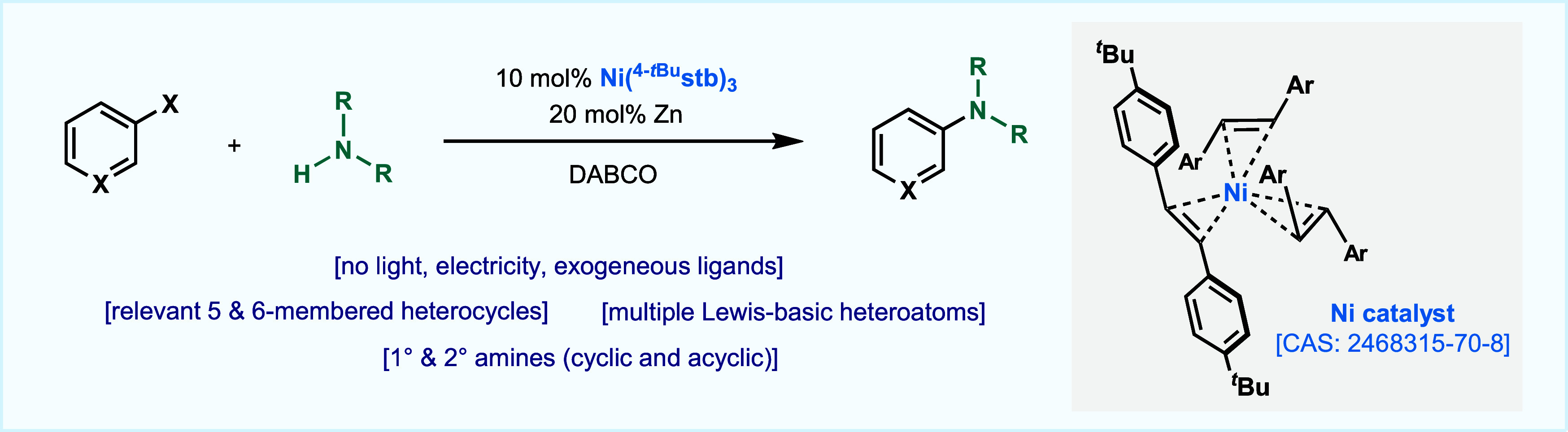

In this Letter, we report that the air-stable “naked nickel” [Ni(^4-*t*Bu^stb)_3_] is a competent catalyst in thermal C–N bond formation between (hetero)aryl bromides and *N*-based nucleophiles. The catalytic system is characterized by a “naked nickel” complex and Zn and by the absence of external light sources, photocatalysts, exogenous ligands, and electrical setups. Upon application of this method, various heteroaryls bearing Lewis-basic heteroatoms can be accommodated and directly aminated with a set of primary and secondary amines.

The catalytic formation of C–N bonds between aromatic (pseudo)halides and amines (Buchwald–Hartwig amination) is one of the cornerstone reactions in modern homogeneous catalysis (Figure 1).^1^ This simple but challenging disconnection has dramatically impacted our society: from the synthesis of medicines and crop-protection agents to polymers and functional materials.^1,2^ Mechanistic studies over the years in the initial Pd-catalyzed C–N cross-coupling reaction have established the canonical Pd^0^/Pd^II^ catalytic redox cycle (Figure 1A).^1b^ In the >50 years since its discovery, many iterations of ligands—mainly P-based—have appeared, which improved the reactivity for more challenging amines and aryl halides.^1b^ In recent years, alternatives to canonical Pd catalysis have appeared in the literature, identifying Cu and Ni as two of the most promising alternatives (Figure 1A).^3^ In 2016, Buchwald and MacMillan provided a new platform that permitted facile C–N bond formation merging photoredox and nickel catalysis (Figure 1B),^4^ avoiding the design and multistep synthesis of supporting ligands. Whereas in conventional metal-catalyzed C–N coupling reactions the reductive elimination generally occurs from a M^II^ species,^5^ in this metallaphotoredox approach the C–N bond is presumably forged from a Ni^III^ intermediate, which can be accessed *via* photocatalysis.^4,6^ Ni-catalyzed electrochemical approaches were also reported, pioneered by Baran, which complemented and even expanded the scope of opportunities for C–N bond formation with Ni (Figure 1B).^7,8^ Building upon this new mechanistic paradigm, in 2020, Nocera and co-workers reported a Ni-catalyzed protocol that allowed the formation of C–N bonds between electron-deficient aryl bromides and amines through a proposed Ni^I^/Ni^III^ redox process, without the need for light or a photocatalyst (Figure 1C). Similar to the photoredox processes, no additional ligands on the Ni catalyst were required.^9^ However, this proof-of-concept study was applied to simple bromoarenes. Importantly, no examples of heterocycles were reported;^9^ this is not surprising, as coordination of a heteroaryl group to the metal center could result in poisoning of the catalyst when no exogenous ligands are present.^10^ We hypothesized that this latter approach could be attractive to practitioners if expanded to accommodate heterocycles and various amines. In 1963 Wilke described Ni^0^-olefin complexes, such as the air-sensitive Ni(COD)_2_, as “naked nickel” due to the lability of the coordinated olefins.^11^ Herein, we report that a robust, air-stable, and commercially available “naked nickel” complex [Ni(^4-*t*Bu^stb)_3_, CAS Registry No. 2468315-70-8],^12^ developed by our group, can catalyze the coupling of heteroaryl bromides with various *N*-based nucleophiles in the absence of ancillary ligands and electro- or photochemical setups (Figure 1D).

**Figure 1:**
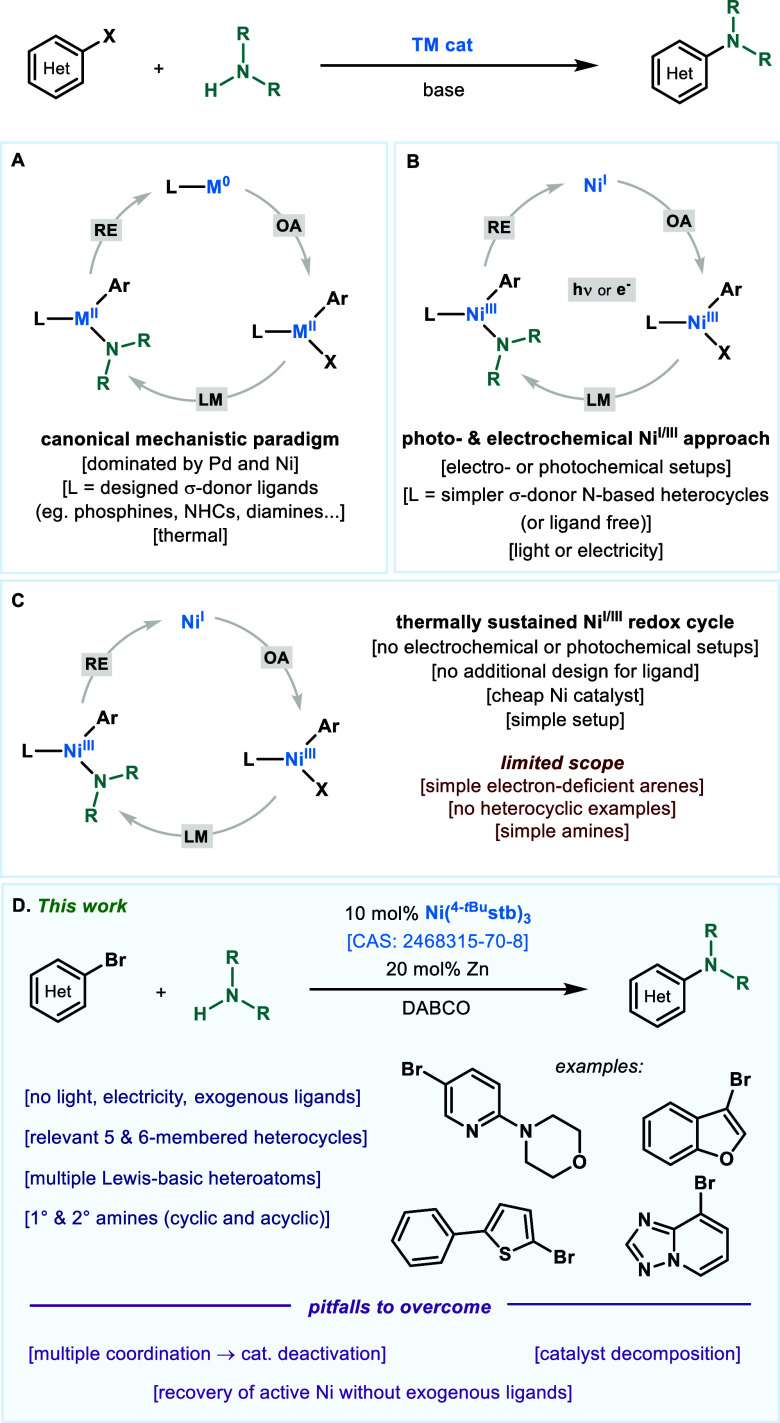
Transition metal-catalyzed C–N cross-coupling reaction (Buchwald–Hartwig amination). (A) Traditional M^0^/^II^ redox cycle. (B) Emerging Ni-catalyzed photo- and electrochemical strategies *via* SET. (C) Ligand-free, thermal Ni-catalyzed amination. (D) A “naked nickel”-catalyzed amination of heteroaryl bromides (this work).

We began to explore the process using 3-bromopyridine (**1**) due to its recalcitrance toward S_N_Ar reactivity with amines. The reaction of **1** with 2.0 equiv of piperidine (**2**) in the presence of DABCO (1.8 equiv), Ni(^4-*t*Bu^stb)_3_ (10 mol %), and Zn (20 mol %) in DMA (1 M) at 60 °C resulted in a 76% isolated yield of coupling product **3** (Scheme 1, entry 1). While the yield of **3** was only slightly reduced at 40 °C (Scheme 1, entry 2), only trace amounts of the product were observed at 25 °C (entry 3). Importantly, the storage of Ni(^4-*t*Bu^stb)_3_ in the freezer (−18 °C) under air for ∼6 months resulted in similar reactivity, highlighting the robustness of the protocol (Scheme 1, entry 4). High yields could also be obtained with NiBr_2_(dme) when it was handled inside the glovebox (Scheme 1, entry 5). NiBr_2_(bipy)_3_, a complex successfully employed in electrocatalytic amination,^7b^ led to no reactivity in our system (Scheme 1, entry 6). Expectedly, Ni(COD)_2_ also afforded **3** in good yields when the precatalyst was handled under an inert atmosphere (Scheme 1, entry 7).

**Scheme 1:**
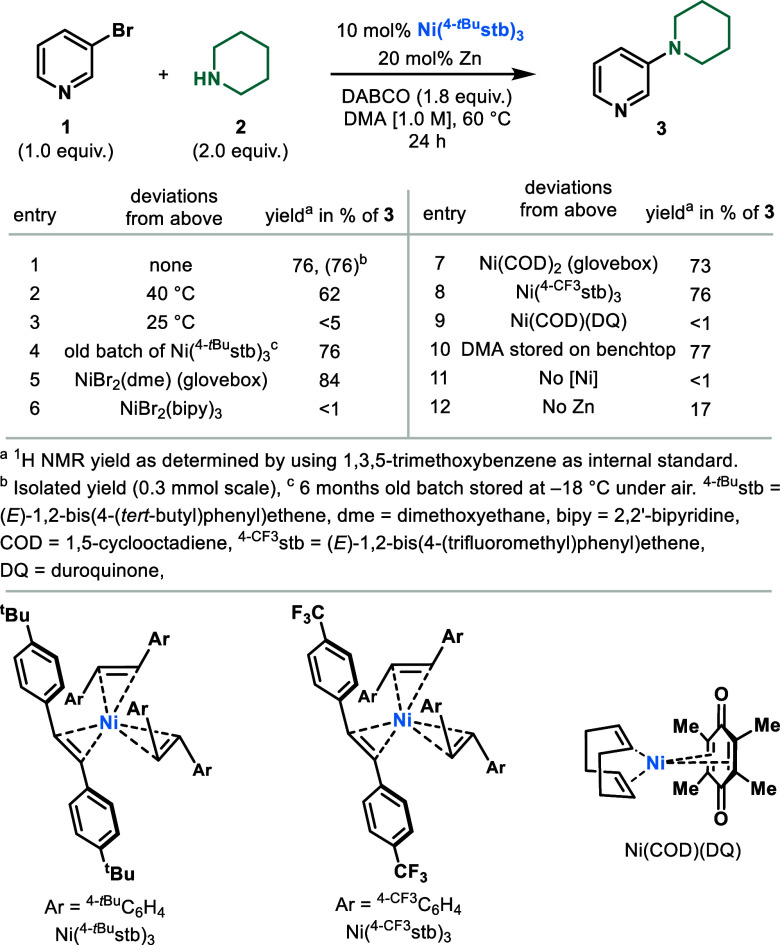
Optimization of the “Naked Nickel”-Catalyzed Coupling of 3-Bromopyridine with Piperidine

Ni(^4-CF3^stb)_3_ as an alternative air-stable Ni^0^ source also performed well (Scheme 1, entry 8), yet the utilization of Ni(COD)(DQ) did not result in the formation of **3** (Scheme 1, entry 9).^13^ Furthermore, the reaction could be carried out in DMA, which was stored on the benchtop without special precautions (Scheme 1, entry 10). Control experiments (entries 11 and 12) confirmed the requirement of both Ni and Zn for the C–N coupling to proceed. With the optimized conditions in hand, we explored the scope of the protocol with regard to heteroaryl bromides using piperidine as the model nucleophile (Figure 2). In addition to electronically unbiased 3-bromopyridine (**3**), derivatives bearing electron-withdrawing (**4** and **5**) or electron-donating substituents (**6**) could be coupled in good yields. Ester functionalities in **4** and **16** remained intact under the reaction conditions.

**Figure 2:**
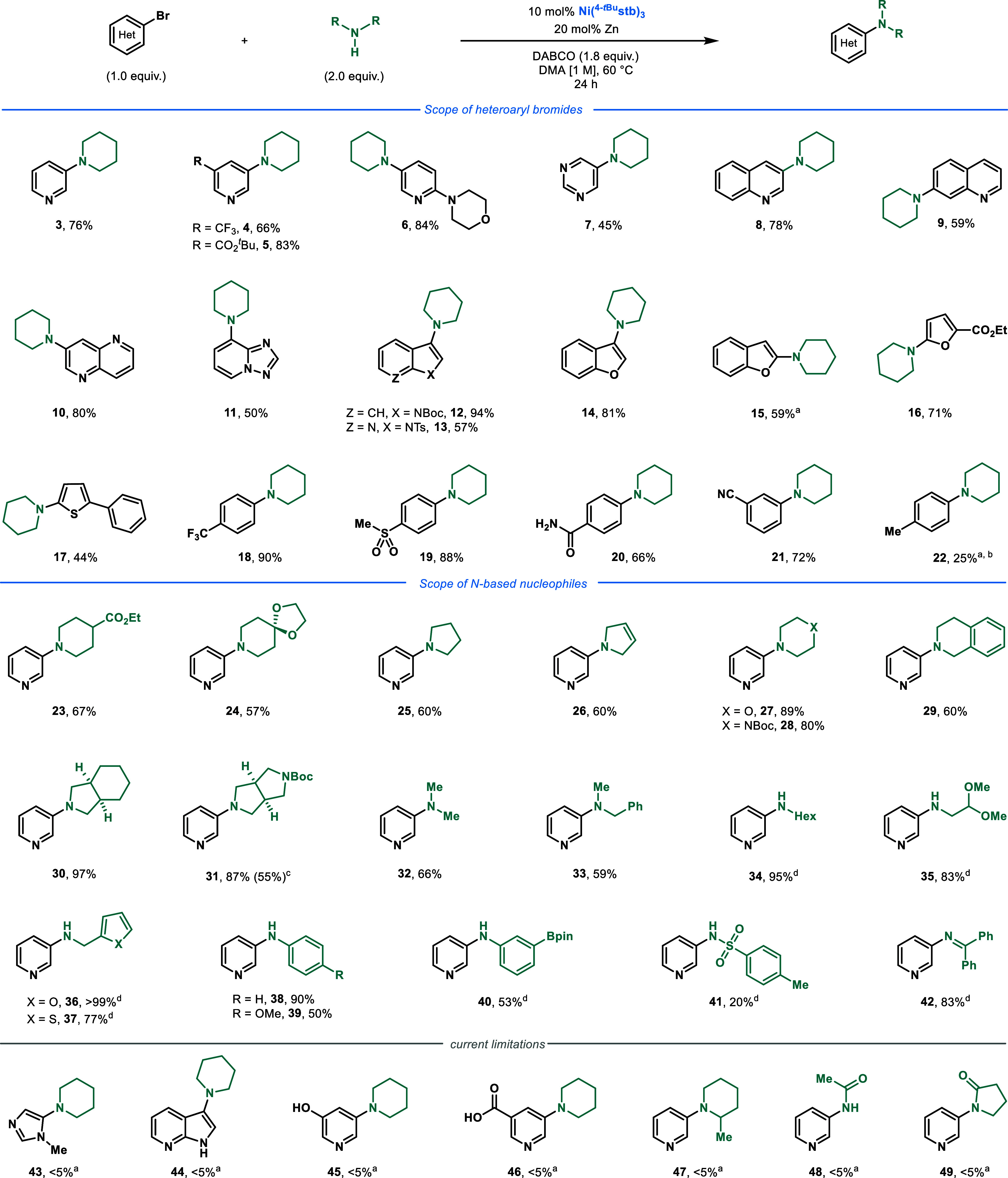
Scope of the amination protocol. Reaction conditions: (hetero)aryl bromide (1.0 equiv), amine (2.0 equiv), DABCO (1.8 equiv), Ni(^4-*t*Bu^stb)_3_ (10 mol %), Zn dust (<60 μm particle size, 20 mol %), DMA (1 M), 60 °C, 24 h, 0.3 mmol scale. Yields represent isolated yields. ^a1^H NMR yield, as determined by using 1,3,5-trimethoxybenzene as the internal standard. ^b^Quinuclidine (1.8 equiv) instead of DABCO. ^c^On a 5.0 mmol scale. ^d^Deviations from the standard reaction conditions: *^t^*Bu-TMG (1.8 equiv), Ni(^4-*t*Bu^stb)_3_ (5 mol %), Zn dust (<60 μm particle size, 10 mol %), 100 °C.

The coupling of 5-bromopyrimidine with piperidine delivered product **7** in moderate yield. We demonstrated that both 3- and 7-bromoquinolines could satisfactorily deliver the desired product in high yields (**8** and **9**, respectively). In addition to quinolines, fused heteroaromatics such as 3-bromo-1,5-naphthyridine could also be successfully coupled in high yield (**10**). A [5,6]-fused heterocycle such as 8-bromo[1,2,4]triazolo[1,5-*a*]pyridine (**11**) was also amenable for coupling. Electron-rich five-membered heterocycles such as *N*-Boc-protected 3-bromoindole resulted in the formation of the desired product (**12**) in excellent yield. 3-Bromo-1-tosyl-1*H*-pyrrolo[2,3-*b*]pyridine containing an additional Lewis-basic N atom could also be efficiently coupled (**13**). Furthermore, benzofuran with bromides at positions C-3 (**14**) and C-2 (**15**) smoothly underwent coupling to form the desired products in good yields. Ethyl 5-bromofuran-2-carboxylate underwent smooth coupling, resulting in the formation of **16** in good yield. Finally, 2-bromo-5-phenylthiophene could be coupled to piperidine, giving the desired product (**17**) in moderate yield. Additionally, we were interested in benchmarking our protocol in the coupling of aryl bromides. Indeed, aryl bromides containing electron-withdrawing substituents such as CF_3_, methylsulfone, CN, and unprotected carboxamide (**18**–**21**, respectively) could be coupled in good to excellent yields, thus comparing favorably to state-of-the-art protocols.^4,9^ Although coupling was observed for 4-bromotoluene, using quinuclidine as a base, the yield of **22** remained low. Next, we were interested in the scope of amines that could be used in our protocol (Figure 2). Piperidine derivatives bearing both ester (**23**) and cyclic acetal (**24**) functionalities could be coupled in good yields. Furthermore, the coupling of pyrrolidine (**25**) and 3-pyrroline (**26**) was also performed with high efficiency. It is important to mention that the absence of electron-rich ligands for the Ni catalyst permits the coupling of 3-pyrroline without undesired Heck or isomerization side reactivity.^14^ Amines with additional heteroatoms such as morpholine (**27**) and *N*-Boc-piperazine (**28**) also delivered high yields of the C–N coupling product.

Bicyclic secondary amines were smoothly coupled with 3-bromopyridine, resulting in the formation of products **29**–**31**. As exemplified by **31**, the reaction can also be scaled to 5 mmol, albeit in a lower yield. Furthermore, dimethylamine and *N*-methyl benzylamine could be successfully coupled in good yields (**32** and **33**, respectively). Next we turned our attention to primary amines, which required slight modifications (see the Supporting Information for details). Under these reaction conditions, hexylamine and 2,2-dimethoxyethylamine bearing an acylic acetal moiety underwent efficient coupling (**34** and **35**, respectively). Furthermore, aliphatic amines bearing pendant heteroaromatic substituents underwent efficient coupling with 3-bromopyridine (**36** and **37**). Different anilines also engaged smoothly in the desired reactivity (**38** and **39**) when using DABCO and 10 mol % Ni catalyst at 60 °C. An aniline bearing a *m*-Bpin functionality (which can act as a handle for further functionalization) was amenable under the readjusted reaction conditions, affording product **40** in moderate yield. Finally, other *N*-based nucleophiles could also be applied; while the coupling with tosylamide afforded **41** in low yields (20%), the coupling of benzophenone imine resulted in the formation of **42** in 83% isolated yield. Imidazoles (**43**), pyridines bearing protic functionalities (**44**–**46**), sterically encumbered amines (**47**), or amides (**48** and **49**) represent some of the current limitations of the protocol (for more details on limitations, see the Supporting Information).

In 2020, Nocera stated that the coupling of heteroaryl bromides was not feasible as a result of the unproductive substrate coordination, resulting in coordinatively saturated Ni^I^ species.^9^ In contrast, the use of NiBr_2_(dme) seemed feasible under our optimized conditions when **1** was used (Scheme 1, entry 5). Hence, we benchmarked the use of NiBr_2_(dme) and Ni(^4-*t*Bu^stb)_3_ with other heteroaryl bromides. In agreement with Nocera,^9^ the yields were substantially decreased when using NiBr_2_(dme). For example, in the case of **6**, the yield with Ni^II^ plummeted from 84% to 45% (Figure 3). Furthermore, in the case of **7**, the yield decreased to 18%. Addition of 30 mol %^4-*t*Bu^stb did not help and further reduced the yield to 12%, showcasing the importance of applying Ni(^4-*t*Bu^stb)_3_ over ill-defined low-valent Ni species formed *in situ* from Ni^II^.^15^ Finally, in the case of **15**, the yield decreased to 45% and that of **17** to 22%.

**Figure 3:**
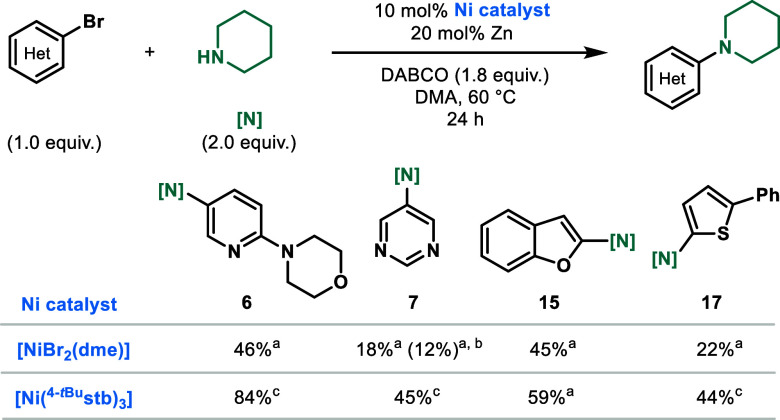
Direct comparison between NiBr_2_(dme) and Ni(^4-*t*Bu^stb)_3_ as catalysts. ^a1^H NMR yields using 1,3,5-trimethoxybenzene as the internal standard. ^b^With 30 mol %^4-*t*Bu^stb added. ^c^Isolated yields.

Through analysis of the reaction mixture and on the basis of the work by Mizoroki, secondary amines should readily coordinate to the Ni center in preference of tertiary amines.^16^ Slowly cooling a solution of Ni(^4-*t*Bu^stb)_3_ with excess piperidine in toluene resulted in the formation of crystals of Ni(^4-*t*Bu^stb)_2_(piperidine) (**50**) suitable for X-ray diffraction (XRD) analysis (Figure 4). Coordination of the N atom to the Ni^0^ center has a relatively weak effect on the elongation of the C(sp^2^)=C(sp^2^) bond, suggesting minimal π back-donation [average of 1.40 Å in **50** vs 1.39 Å in Ni(^4-*t*Bu^stb)_3_],^12^ which is in accordance with a tetrahydrofuran (THF) derivative derived from Ni(^4-CF3^stb)_3_ [average of 1.41 Å in Ni(^4-CF3^stb)_2_(THF) vs 1.39 Å in Ni(^4-^^CF3^stb)_3_].^17^

**Figure 4:**
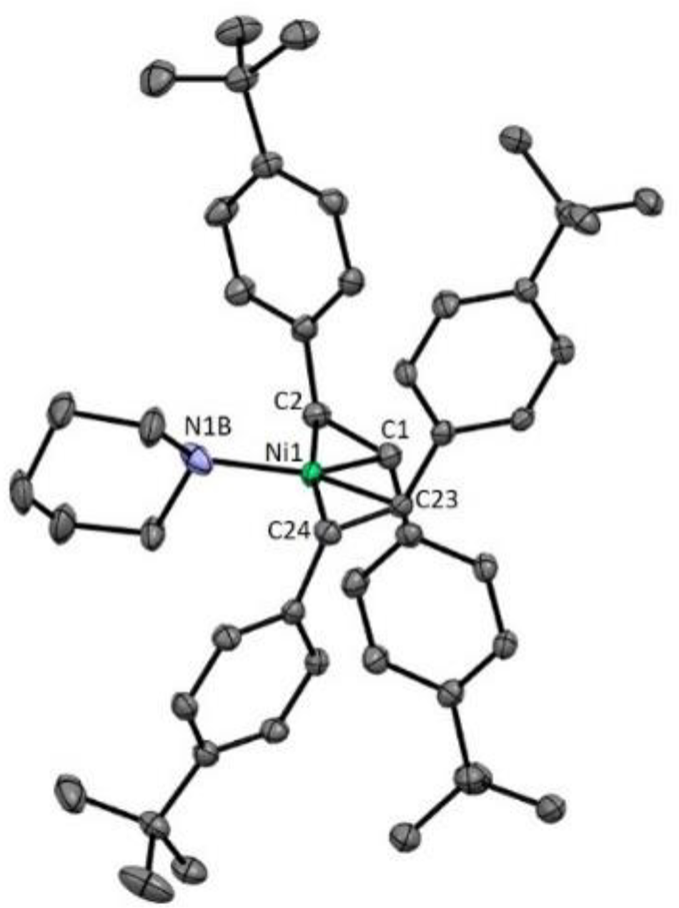
XRD structure of Ni(^4-*t*Bu^stb)_2_(piperidine) (**50**) visualized with 50% probability ellipsoids. Color code: green for Ni, black for C, and purple for N. Disordered piperidine and hydrogen atoms have been omitted for the sake of clarity. C(sp^2^)=C(sp^2^) bond distances of the olefinic bonds: 1.405(3) and 1.403(3) Å.

By analogy to the thermally sustained Ni^I^/Ni^III^ protocol reported by Nocera et al., we are inclined to propose that a similar pathway might be operative in our system, and species similar to **50** might prevent catalyst deactivation in our system. However, this hypothesis remains purely speculative at this point.

In conclusion, we have developed a catalytic protocol based on a “naked nickel” complex, which permits the coupling of various heteroaryl bromides bearing Lewis-basic heteroatoms with a variety of primary and secondary alkyl- and arylamines. The catalytic protocol proceeds under thermal conditions without the need for light or electrochemical setups. The protocol also allows the coupling to proceed in the absence of finely tuned exogenous ligands. These results do not aim to replace current catalytic strategies for C–N bond formation but serve as a proof-of-concept protocol for C–N cross-couplings, in which certain heteroaryl bromides can be coupled to amines using a catalytic system with Ni and olefins.

## Data Availability

The data underlying this study are available in the published article and its Supporting Information.

## Supporting Information Available

General considerations, ligand and catalyst preparation, synthesis of known starting materials, general procedures, optimization, products, mechanistic investigations, spectra, and crystallographic data used in this study (PDF)
