# Supplementary material for: “Naked Nickel”-Catalyzed Amination of Heteroaryl Bromides
Source: Org Lett. 2024 Jul 5;26(28):5928–33. doi: 10.1021/acs.orglett.4c01738 (PMC11267598; doi:10.1021/acs.orglett.4c01738)
Supplement: Supplementary file 1 — ol4c01738_si_001.pdf [file ol4c01738_si_001.pdf]

Supporting Information

"Naked Nickel"-Catalyzed Amination of Heteroaryl Bromides

Rakan Saeb, Bryan Boulenger, and Josep Cornella*

Max-Planck-Institut für Kohlenforschung, Department of Organometallic Chemistry, Kaiser-Wilhelm-Platz 1, 45470, Mülheim an der Ruhr, North Rhine-Westphalia, Germany

Table of Contents

General Considerations 3

Ligand and Catalyst Preparation 4

Preparation of Starting Materials 6

General Procedures for Catalytic C–N Bond Forming Reactions 7

Reaction Optimization 9

Characterization Data 13

5 mmol Scale Synthesis of *tert*-butyl 5-(pyridin-3-yl)hexahydropyrrolo[3,4-*c*]pyrrole-2(1*H*)-carboxylate (31): 33

Limitations of the Scope 34

Coordination of Piperidine to Ni($^{4\text{-}t\text{Bu}}$stb)$_3$ 35

References 42

NMR Spectra 44

Crystallographic Data 89

General Considerations

Unless otherwise stated, all manipulations were performed using simple Schlenk techniques (weigh in solids, one cycle of vacuum/argon and then add liquids/solvent) under dry argon in heatgun-dried glassware. Unless otherwise stated all reactions were carried out using anhydrous solvents. Ni($^{4\text{-}t\text{Bu}}$stb)$_3$ (tris(*trans*-1,2-bis(4-*tert*-butylphenyl)ethene)nickel(0), CAS: 2468315-70-8) was stored in a screw cap vial under air in a freezer (−18 °C) and was handled open to air under ambient conditions. Unless otherwise noted, all reagents were obtained from commercial suppliers and used without further purification. Aniline was distilled prior to use. Anhydrous DMA (250 mL, 99.8%, SureSeal), was purchased from Sigma-Aldrich. Zinc dust (< 60 µm particle size) was purchased from Merck, stored on the bench, and used as received without further activation. Column chromatography: Merck silica gel 60 (40-63 µm). MS (EI): Finnigan MAT 8200 (70 eV), ESI-MS: ESQ 3000 (Bruker). Accurate mass determinations: Bruker APEX III FT-MS (7 T magnet), QExactiveGC (Thermo Fischer), or MAT 95 (Finnigan). Mass analyzer type: Quadrupol-Orbitrap. NMR spectra for characterization were recorded on a Bruker AVIII HD 300, AVIII HD 400, AVIII 500 MHz or AVNeo 600 MHz NMR spectrometer (at 298-300 K, unless otherwise stated). Single crystals were measured on a Bruker-AXS Kappa Mach3 with APEX-II detector and IµS microfocus source. IR spectra were recorded on an Alpha Platinum ATR instrument (Bruker); wavenumbers ($\tilde{\nu}$) in cm^{-1}. Melting points were recorded on an E-Z melt (MPA120) melting point apparatus (Stanford Research Systems) or on a Büchi melting point apparatus, Model B-540 (Büchi, Switzerland) and are uncorrected. ^{1}H NMR spectra were referenced to the residual protons of the deuterated solvent used (δ ($CDCl_3$) = 7.26 ppm) (neutralized with Na_2CO_3), δ (THF-d_8) = 3.58 ppm) (dried over Na, distilled, and stored in the glovebox under argon). ^{13}C NMR spectra were referenced internally to the D-coupled ^{13}C resonances of the NMR solvent. Chemical shifts (δ) are given in ppm, relative to TMS (tetramethylsilane), and coupling constants (J) are provided in Hz. ^{19}F NMR spectra were referenced externally to the ^{19}F resonances of $CFCl_3$. Multiplicities are reported as (s = singlet, d = doublet, t = triplet, q = quartet, p = pentet, m = multiplet or unresolved, brs = broad signal). Structural assignments were made with additional information from gCOSY, gHSQC, and gHMBC experiments.

Note: Many of the amination products tend to be rather unstable; hence, it is advised to store such substrates in a freezer at −18 °C under argon.

Ligand and Catalyst Preparation

(*E*)-1,2-bis(4-(*tert*-butyl)phenyl)ethene ($^{4\text{-}t\text{Bu}}$stb):

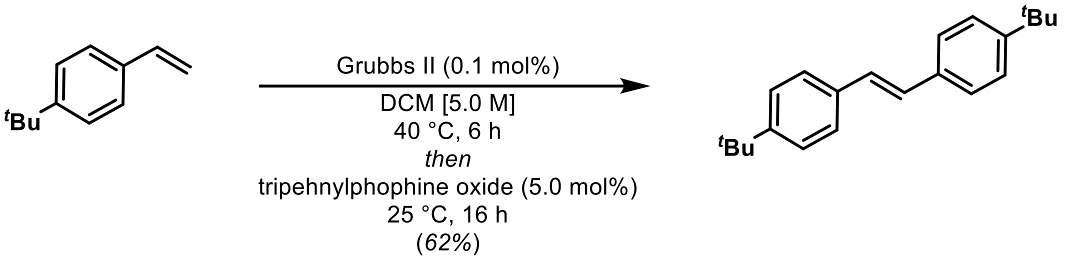

A 100 ml two-necked round-bottomed flask, equipped with a reflux condenser and stirring bar and held under argon was charged with $^{4\text{-}t}$Bu-styrene (10.9 mL, 60.13 mmol, 1.0 equiv.), Grubbs II catalyst (51.5 mg, 0.06 mmol, 0.1 mol%) and DCM (12 mL). The reaction was refluxed under vigorous stirring for 6 h, after which the reaction was allowed to cool down to ambient temperature, followed by addition of triphenylphosphine oxide (0.86 g, 3.09 mmol, 5.0 mol%). After stirring over night at the same temperature, the reaction mixture was concentrated under reduced pressure. The solid material was dissolved in DMF (400 mL), and the resulting solution was complemented with H_2O (200 mL), where upon an off-white solid precipitated. The solid was filtered with a D3 fritted filter and washed with H_2O (300 mL). The remainings were dissolved in DCM (400 mL) and washed with H_2O (200 mL). The organic layer was dried over $MgSO_4$, and partially concentrated under reduced pressure (approximately 150 mL). The solution was loaded onto a column and the material was purified *via* column chromatography over silica gel (hexanes), to afford $^{4\text{-}t\text{Bu}}$stb as a white solid (5.43 g, 18.56 mmol, 62%).

Spectroscopic data match those reported in the literature.[1]

***R_f*:** 0.25 (hexanes)

^{1}H NMR (300 MHz, $CDCl_3$) δ 7.51 – 7.43 (m, 4H), 7.41 – 7.34 (m, 4H), 7.07 (s, 2H), 1.34 (s, 18H).

^{13}C NMR (75 MHz, $CDCl_3$) δ 150.7, 135.0, 127.9, 126.3, 125.7, 34.8, 31.5.

Note: As Grubbs II catalyst tends to co-elute with the metathesis product, the addition of triphenylphosphine oxide is advised, as it generates a more polar Ru-complex, which can be more easily separated from the product.[2]

Ni($^{4\text{-}t\text{Bu}}$stb)$_3$:

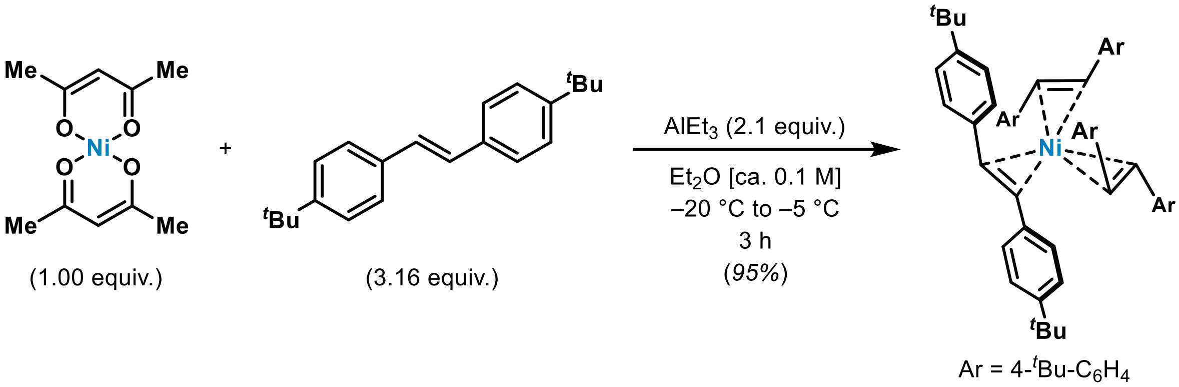

Ni($^{4\text{-}t\text{Bu}}$stb)$_3$ was prepared using a slightly modified procedure.[1]

A Schlenk flask equipped with a stirring bar, held under argon was charged with anhydrous Ni(acac)$_2$ (1.50 g, 5.84 mmol, 1.00 equiv.) *via* argon trousers, followed by addition of $^{4\text{-}t\text{Bu}}$stb (5.40 g, 18.46 mmol, 3.16 equiv.). One cycle of vacuum/ argon was performed. Anhydrous Et_2O (60 mL) was added and the solution was cooled to −20 °C using a cryostat. A solution of $AlEt_3$ (1 M in hexanes, 12.3 mL, 2.11 equiv.) was added dropwise over 2 minutes to the reaction vessel. The reaction was allowed to warm up to −5 °C and stirred at that temperature for 3 hours. The suspension was cooled down to −35 °C *via* the addition of dry ice, stirred for 5 minutes at that temperature and then transferred (by using PE tubing) to a jacketed argon frit cooled to −35 °C. The suspension was filtered, leaving the product on the frit. The solid was washed with Et_2O (5 x 5 mL). The argon frit was transferred onto another Schlenk tube held under argon and dried under high vacuum, to afford the desired product as an orange solid (5.18 g, 5.54 mmol, 95%).

Spectroscopic data match those reported in the literature.[1]

Note: When dissolved in THF-d_8 one ligand is being exchanged by one molecule of THF-d_8, resulting in a mixture consisting of Ni($^{4\text{-}t\text{Bu}}$stb)$_2$(THF-d_8) and $^{4\text{-}t\text{Bu}}$stb (1:1).

^{1}H NMR (−80 °C, 500 MHz, THF-d_8) δ 7.81 (d, J = 8.1 Hz, 4H), 7.41 (d, J = 8.0 Hz, 4H), 6.51 – 6.45 (m, 4H), 4.94 (s, 4H), 4.68 (d, J = 8.5 Hz, 4H), 1.32 (s, 36H).

Free ligand: **^{1}H NMR (−80 °C, 500 MHz, THF-d_8)** δ 7.54 (d, J = 8.1 Hz, 4H), 7.41 (d, J = 8.0 Hz, 4H), 7.25 (s, 2H), 1.32 (s, 18H).

Preparation of Starting Materials

tert-Butyl 5-bromonicotinate (SM1):

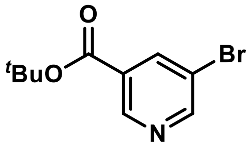

tert-Butyl 5-bromonicotinate was prepared according to a procedure reported in the literature.[3]

^{1}H NMR spectroscopic data matched those reported in the literature.[3]

^{1}H NMR (300 MHz, CDCl$_3$) δ 9.07 (d, *J* = 1.8 Hz, 1H), 8.80 (d, *J* = 2.3 Hz, 1H), 8.36 (dd, *J* = 2.3, 1.8 Hz, 1H), 1.61 (s, 9H).

3-Bromo-1-tosyl-1*H*-pyrrolo[2,3-*b*]pyridine (SM2):

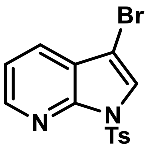

3-Bromo-1-tosyl-1*H*-pyrrolo[2,3-*b*]pyridine was prepared according to a procedure reported in the literature.[4]

^{1}H NMR spectroscopic data matched those reported in the literature.[5]

^{1}H NMR (300 MHz, CDCl$_3$) δ 8.47 (dd, *J* = 4.8, 1.6 Hz, 1H), 8.08 (d, *J* = 8.4 Hz, 2H), 7.85 – 7.76 (m, 2H), 7.33 – 7.20 (m, 3H), 2.38 (s, 3H).

General Procedures for Catalytic C–N Bond Forming Reactions

General procedure 1 (GP-1) for the coupling of (hetero)aryl bromides with secondary amines and anilines:

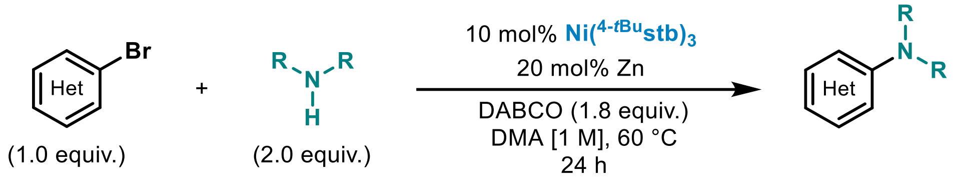

A 12-mL screwcap vial equipped with a stirring bar was dried under vacuum using a heatgun set to 550 °C, followed by three cycles of vacuum/ argon. The vial was charged with Ni($^{4\text{-}t\text{Bu}}$stb)$_3$ (10 mol%), Zn dust (< 60 µm, 20 mol%), DABCO (1.8 equiv.) and with solid (hetero)aryl bromide (1.0 equiv.), and amine (2.0 equiv.), weighed out under air. One cycle of vacuum/ argon was performed. The reaction vessel was charged with liquid coupling partners using a Hamilton syringe through a rubber septum, while bubbling argon through the liquid materials. DMA (1 M, corresponding to the heteroaryl bromide) was added using a syringe through a rubber septum, the cap of the vial was switched for a new one, and the reaction tube was placed into a pre-heated oil bath held at 60 °C. The reaction was stirred for 24 h at that temperature and was then allowed to cool down to ambient temperature. The reaction was diluted with EtOAc, quenched by the addition of an aqueous solution of HCl (1 M), followed by neutralizing with a saturated aqueous solution of $NaHCO_3$. A saturated aqueous solution of LiCl was added, and the phases were separated. The organic phase was washed with a saturated aqueous solution of LiCl (3 x), followed by back extraction of the combined aqueous layers with EtOAc (1-2 x). The combined organic layers were dried over $MgSO_4$ and concentrated under reduced pressure. Purification *via* column chromatography over silica gel afforded the pure desired product.

General procedure 2 (GP-2) for the coupling of 3-bromopyridine with aliphatic primary amines, less nucleophilic anilines, imines and sulfonamides:

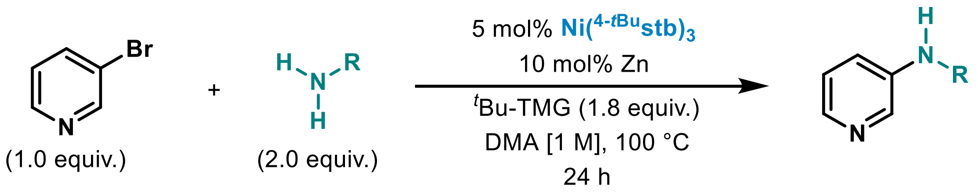

A 12-mL screwcap vial equipped with a stirring bar was dried under vacuum using a heatgun set to 550 °C, followed by three cycles of vacuum/ argon. The vial was charged with Ni($^{4\text{-}t\text{Bu}}$stb)$_3$ (5 mol%), Zn dust (< 60 µm, 10 mol%), and with the solid amine (2.0 equiv.), weighed out under air. One cycle of vacuum/ argon was performed. The reaction vessel was charged with 3-bromopyridine (1.0 equiv.) using a Hamilton syringe, ^{t}Bu-TMG (2-*tert*-butyl-1,1,3,3-tetramethylguanidine) (1.8 equiv.), and with the liquid amine (2.0 equiv.) through a rubber septum, while bubbling argon through the liquid materials. DMA (1 M, corresponding to 3-bromo pyridine) was added through the rubber septum, the

cap of the vial was switched for a new one, and the reaction tube was placed into a pre-heated oil bath held at 100 °C. The reaction was stirred for 24 h at that temperature and was then allowed to cool down to ambient temperature. The reaction was diluted with EtOAc, quenched by the addition of an aqueous solution of HCl (1 M), followed by neutralizing with a saturated aqueous solution of $NaHCO_3$. A saturated aqueous solution of LiCl was added, and the phases were separated. The organic phase was washed with a saturated aqueous solution of LiCl (3 x), followed by back extraction of the combined aqueous layers with EtOAc (1-2 x). The combined organic layers were dried over $MgSO_4$ and concentrated under reduced pressure. Purification *via* column chromatography over silica gel afforded the pure desired product.

Reaction Optimization

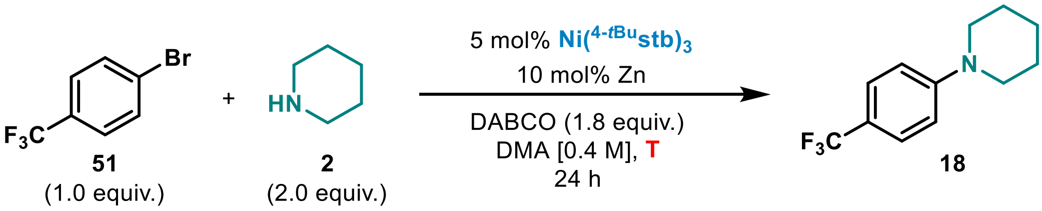

entry	reaction temperature	conversion[a] of **51** into **18** in %
1	25 °C	28
2	40 °C	93

[a] represents % of product peak in the crude reaction mixture as determined by ^{19}F NMR.

Scheme S1: Initial Reaction Optimization – Reaction Temperature.

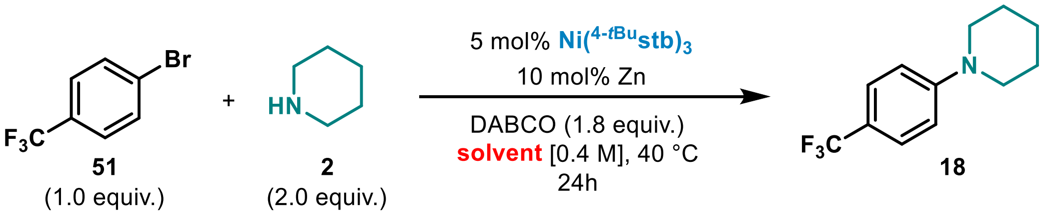

entry	solvent used	conversion[a] of **51** into **18** in %
1	DMA	93
2	DMF	67
3	1,4-Dioxane	7
4	THF	10
5	Toluene	4
6	MeCN	18
7	THF/DMA (3/1)	42

[a] represents % of product peak in the crude reaction mixture as determined by ^{19}F NMR.

Scheme S2: Initial Reaction Optimization – Solvents.

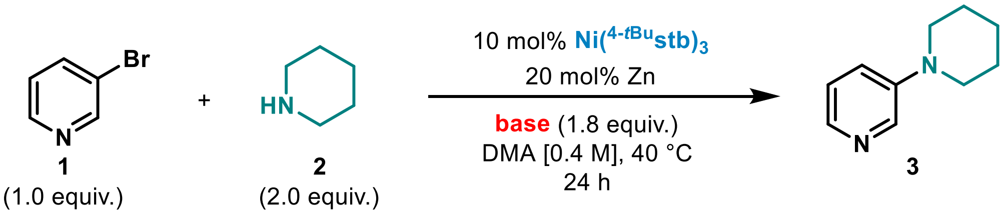

entry	base used	yield[a] in % of **3**
1	DABCO	46
2	Quinuclidine	34
3	NEt_3	22
4	DBU	n.d.
5	N^iPr_2Et	18
6	^{t}Bu-TMG	<5

[a] ^{1}H NMR yield as determined by using 1,3,5-trimethoxybenzene as internal standard.
n.d. = not detected

Scheme S3: Reaction Optimization for Heteroaryl Bromides – Bases.

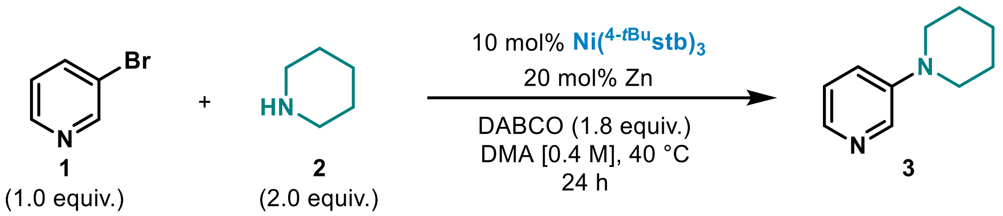

entry	deviations from above	yield[a] in % of **3**
1	none	46
2	5 mol% [Ni], 10 mol% Zn	40
3	DMA [1 M]	62
4	20 mol% activated Zn, DMA [1 M]	54
5	20 mol% Mn, DMA [1 M]	<5
6	DMA [1 M], NaI (1.5 equiv.)	<5
7	DMA [1 M], rt	<5
8	DMA [1 M], 60 °C	76
9	no Zn, DMA [1 M], 60 °C	17
10	"wet" DMA [1 M], 60 °C	77

[a] ^{1}H NMR yield as determined by using 1,3,5-trimethoxybenzene as internal standard.
n.d. = not detected

Scheme S4: Reaction Optimization for Heteroaryl Bromides – Further Optimizations.

Note: Activated Zn refers to Zn dust that was treated with HCl (37%), followed by washing with deionized H_2O, acetone and pentane, followed by drying under high vacuum prior to use.

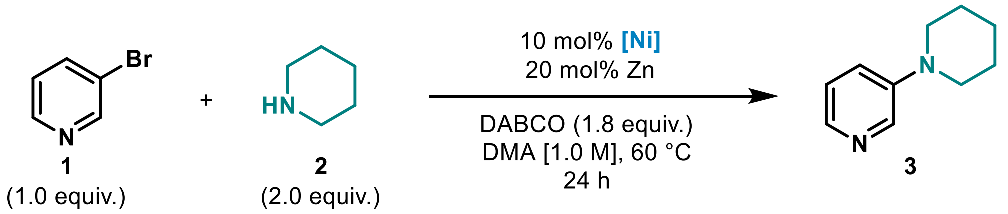

entry	Ni catalyst used	yield[a] in % of **3**
1	Ni($^{4\text{-}t\text{Bu}}$stb)$_3$	76
2	Ni($^{4\text{-}t\text{Bu}}$stb)$_3$ (stored for >6 months under air at –18 °C)	76
3	Ni($^{4\text{-}t\text{Bu}}$stb)$_3$ (stored for 3 days under air for 25 °C)	76
4	Ni($^{4\text{-CF3}}$stb)$_3$	76
5	Ni(COD)$_2$	73
6	Ni(COD)(DQ)	n.d.
7	NiBr$_2$(dme)	84
8	NiBr$_2$(bipy)$_3$	n.d.
9	No [Ni]	n.d.

[a] ^{1}H NMR yield as determined by using 1,3,5-trimethoxybenzene as internal standard. n.d. = not detected, $^{4\text{-CF3}}$stb = (*E*)-1,2-bis(4-(triflurومethyl)phenyl)ethene, COD = 1,5-cyclooctadiene, DQ = duroquinone, dme = 1,2-dimethoxyethane, bipy = 2,2'-bipyridine

Scheme S5: Reaction Optimization for Heteroaryl Bromides – Nickel Sources.

Note 1: Ni($^{4\text{-CF3}}$stb)$_3$ exhibits the same reactivity as Ni($^{4\text{-}t\text{Bu}}$stb)$_3$, however latter complex is known to be stable for a longer time in solid state. Hence, Ni($^{4\text{-}t\text{Bu}}$stb)$_3$ was chosen as the catalyst.

Note 2: Reactions with Ni($^{4\text{-}t\text{Bu}}$stb)$_3$, Ni($^{4\text{-CF3}}$stb)$_3$, and Ni(COD)(DQ) were set up on the benchtop, reactions involving Ni(COD)$_2$ and NiBr$_2$(dme) were set up in the glovebox.

Note 3: The application of $NiBr_2$(dme) result in the formation of **3** in slightly higher yields, however this Ni-source is not a modular catalyst (*vide infra*, Scheme S6). Hence, Ni($^{4\text{-}t\text{Bu}}$stb)$_3$ was chosen as the catalyst.

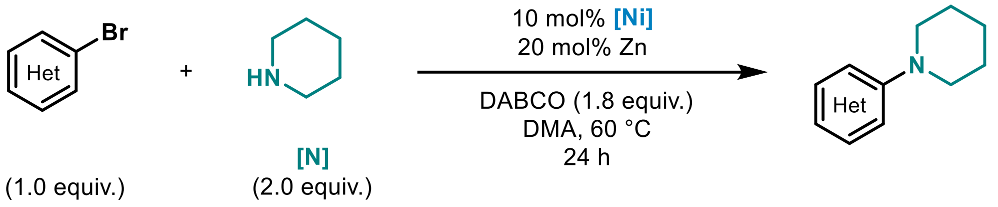

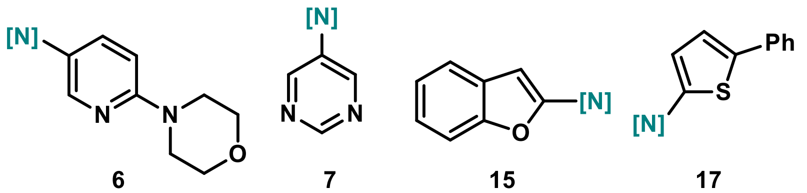

Ni catalyst	6	7	15	17
[$NiBr_2$(dme)]	46%[a]	18%[a] (12%)[a, b]	45%[a]	22%[a]
[Ni($^{4\text{-}t\text{Bu}}$stb)$_3$]	84%[c]	45%[c]	59%[a]	44%[c]

[a] 1H NMR yields using 1,3,5-trimethoxybenzene as internal standard. [b] 30 mol% of $^{4\text{-}t\text{Bu}}$stb was added. [c] Isolated yields.

Scheme S6: Benchmarking against $NiBr_2$(dme).

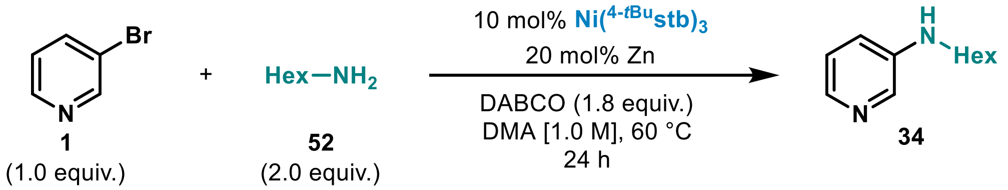

entry	deviations from above	yield[a] in % of **34**
1	none	6
2	20 mol% pyrrolidine as additive	8
3	80 °C	n.d.
4	80 °C, ^{t}Bu-TMG (1.8 equiv.) as base	62
5	100 °C, ^{t}Bu-TMG (1.8 equiv.) as base	72
6	20 mol% [Ni], 40 mol% Zn, 100 °C, ^{t}Bu-TMG (1.8 equiv.) as base	59
7	5 mol% [Ni], 10 mol% Zn, 100 °C, ^{t}Bu-TMG (1.8 equiv.) as base	95[b]

[a] ^{1}H NMR yield as determined by using 1,3,5-trimethoxybenzene as internal standard.
[b] Isolated yield (0.3 mmol scale), n.d. = not detected,

Scheme S7: Reaction Optimization for Coupling Heteroaryl Bromides with Primary Amines.

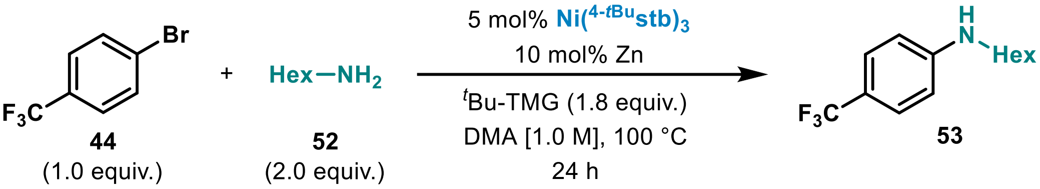

entry	deviations from above	yield[a] in % of **53**
1	none	20
2	10 mol% [Ni], 20 mol% Zn	72
3	10 mol% [Ni], 20 mol% Zn, 80 °C	66

[a] ^{1}H NMR yield as determined by using 1,3,5-trimethoxybenzene as internal standard. n.d. = not detected,

Scheme S8: Reaction Optimization for Coupling Aryl Bromides with Primary Amines.

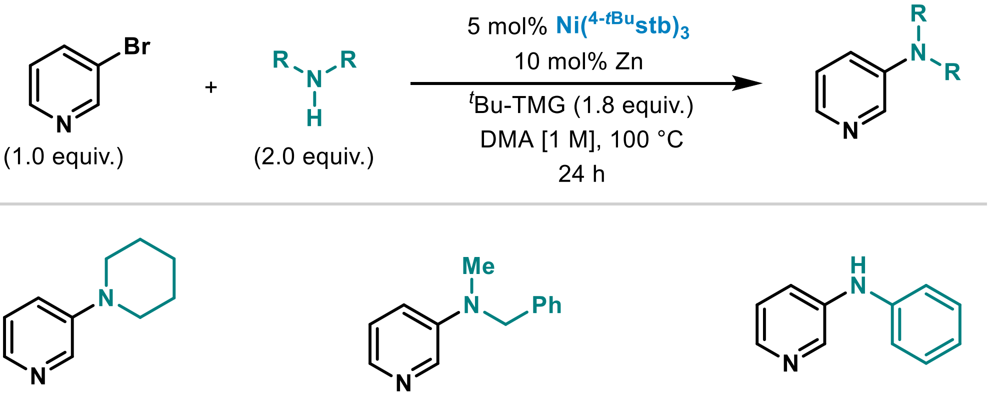

3, 10%[a] (76%)[b]

33, <5%[a] (59%)[b]

38, 39%[a] (90%)[b]

[a] ^{1}H NMR yield as determined by using 1,3,5-trimethoxybenzene as internal standard.
[b] Isolated yield (0.3 mmol scale), following **GP-1**.

Scheme S9: Compatibility of Secondary Amines or Aniline with the Conditions for Primary Amines.

Note: The yield for all three substrates was substantially decreased when using conditions optimized for the coupling of primary amines. Hence, **GP-2** was not used as a general protocol for C–N bond coupling, but only for such coupling reactions involving aliphatic primary amines, electron poor anilines, sulfonamides and imines (*vide infra*).

Characterization Data

3-(Piperidin-1-yl)pyridine (3):

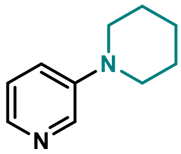

3-(Piperidin-1-yl)pyridine was prepared according to **GP-1**, using Zn dust (3.9 mg, 20 mol%), Ni($^{4\text{-}tBu}$stb)$_3$ (28.2 mg, 10 mol%), DABCO (60.9 mg, 0.54 mmol, 1.8 equiv.), 3-bromopyridine (29 µL, 0.3 mmol, 1.0 equiv.), and piperidine (60 µL, 0.6 mmol, 2.0 equiv.) in DMA (0.3 mL) at 60 °C. Purification *via* column chromatography (silica gel, 50:50 hexanes/EtOAc) afforded the title compound as a colorless oil (36.9 mg, 76%).
Spectroscopic data matched those reported in the literature.[6]
***R$_f$*:** 0.25 (50:50 hexanes/EtOAc)
^{1}H NMR (300 MHz, CDCl$_3$) δ 8.31 (s, 1H), 8.05 (d, J = 4.4 Hz, 1H), 7.21 – 7.09 (m, 2H), 3.24 – 3.11 (m, 4H), 1.76 – 1.67 (m, 4H), 1.65 – 1.55 (m, 2H).
^{13}C NMR (101 MHz, CDCl$_3$) δ 147.9, 140.2, 139.1, 123.5, 122.8, 50.0, 25.7, 24.2.

3-(Piperidin-1-yl)-5-(trifluoromethyl)pyridine (4):

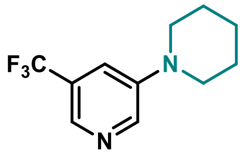

3-(Piperidin-1-yl)-5-(trifluoromethyl)pyridine was prepared according to **GP-1**, using Zn dust (4.0 mg, 20 mol%), Ni($^{4\text{-}tBu}$stb)$_3$ (28.1 mg, 10 mol%), DABCO (60.5 mg, 0.54 mmol, 1.8 equiv.), 3-bromo-5-(trifluoromethyl)pyridine (67.8 mg, 0.3 mmol, 1.0 equiv.), and piperidine (60 µL, 0.6 mmol, 2.0 equiv.) in DMA (0.3 mL) at 60 °C. Purification *via* column chromatography (silica gel, 85:15 hexanes/ EtOAc) afforded the title compound as an orange oil (45.3 mg, 66%).
***R$_f$*:** 0.51 (85:15 hexanes/EtOAc)
^{1}H NMR (300 MHz, CDCl$_3$) δ 8.44 (d, J = 2.9 Hz, 1H), 8.26 (d, J = 1.8 Hz, 1H), 7.31 – 7.28 (m, 1H), 3.31 – 3.21 (m, 4H), 1.79 – 1.56 (m, 6H).
^{13}C NMR (75 MHz, CDCl$_3$) δ 147.1, 141.4 (d, J = 1.6 Hz), 135.6 (q, J = 4.2 Hz), 126.7 (q, J = 32.0 Hz), 123.9 (q, J = 272.7 Hz), 118.1 (q, J = 3.7 Hz), 49.3, 25.4, 24.0.
^{19}F NMR (282 MHz, CDCl$_3$) δ −62.55.
HRMS (EI) m/z: [M] calc'd for $C_{11}H_{13}N_2F_3$: 230.1025, found: 230.1029
FTIR (ATR): $\tilde{\nu}$ [cm^{-1}] = 2938, 2857, 2814, 1596, 1466, 1359, 1304, 1245, 1119, 1090, 1010, 945, 869, 725, 708.

***tert*-Butyl 5-(piperidin-1-yl)nicotinate (5):**

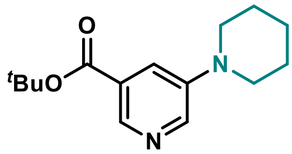

tert-Butyl 5-(piperidin-1-yl)nicotinate was prepared according to **GP-1**, using Zn dust (4.0 mg, 20 mol%), Ni($^{4\text{-}t\text{Bu}}$stb)$_3$ (28.1 mg, 10 mol%), DABCO (60.6 mg, 0.54 mmol, 1.80 equiv.), **SM1** (77.4 mg, 0.3 mmol, 1.0 equiv.), and piperidine (60 µL, 0.6 mmol, 2.0 equiv.) in DMA (0.3 mL) at 60 °C. Purification *via* column chromatography (silica gel, 80:20 hexanes/EtOAc) afforded the title compound as a yellow oil (65.3 mg, 83%).

***R*$_f$:** 0.32 (80:20 hexanes/EtOAc)

^{1}H NMR (400 MHz, CDCl$_3$) δ 8.56 (d, *J* = 1.7 Hz, 1H), 8.40 (d, *J* = 3.0 Hz, 1H), 7.70 (dd, *J* = 3.0, 1.8 Hz, 1H), 3.26 – 3.19 (m, 4H), 1.76 – 1.66 (m, 4H), 1.65 – 1.56 (m, 11H).

^{13}C NMR (101 MHz, CDCl$_3$) δ 165.3, 147.4, 142.0, 140.7, 127.7, 122.8, 81.8, 49.7, 28.3, 25.5, 24.2.

HRMS (ESI) m/z: [M+H]$^+$ calc'd for $C_{15}H_{23}N_2O_2$: 263.1754, found: 263.1754

FTIR (ATR): $\tilde{\nu}$ [cm^{-1}] = 2974, 2929, 2854, 2811, 1713, 1587, 1452, 1367, 1287, 1257, 1160, 1119, 1013, 966, 849, 769, 701.

4-(5-(Piperidin-1-yl)pyridin-2-yl)morpholine (6):

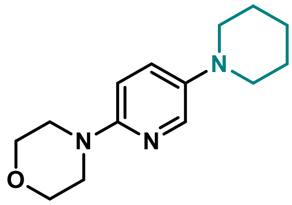

4-(5-(Piperidin-1-yl)pyridin-2-yl)morpholine was prepared according to **GP-1**, using Zn dust (3.9 mg, 20 mol%), Ni($^{4\text{-}t\text{Bu}}$stb)$_3$ (28.1 mg, 10 mol%), DABCO (60.6 mg, 0.54 mmol, 1.80 equiv.), 4-(5-bromopyridin-2-yl)morpholine (73.2 mg, 0.30 mmol, 1.00 equiv.), and piperidine (60 µL, 0.6 mmol, 2.0 equiv.) in DMA (0.3 mL) at 60 °C. Purification *via* column chromatography (silica gel, 80:20 hexanes/EtOAc to EtOAc) afforded the title compound as a beige solid (62.5 mg, 84%).

m.p.: 83.3–84.3 °C

***R*$_f$:** 0.57 (EtOAc)

^{1}H NMR (300 MHz, CDCl$_3$) δ 7.95 (dd, *J* = 3.0, 0.7 Hz, 1H), 7.25 (d, *J* = 8.9 Hz, 1H), 6.62 (dd, *J* = 9.1, 0.7 Hz, 1H), 3.88 – 3.78 (m, 4H), 3.43 – 3.34 (m, 4H), 3.05 – 2.96 (m, 4H), 1.72 (p, *J* = 5.7 Hz, 4H), 1.61 – 1.47 (m, 2H).

^{13}C NMR (101 MHz, CDCl$_3$) δ 154.5, 141.5, 136.8, 129.0, 107.9, 66.9, 52.1, 46.8, 25.9, 24.0.

HRMS (EI) m/z: [M] calc'd for $C_{14}H_{21}N_3O$: 247.1679, found: 247.1683

FTIR (ATR): $\tilde{\nu}$ [cm^{-1}] = 2959, 2922, 2850, 1493, 1446, 1399, 1373, 1330, 1244, 1213, 1115, 941, 913, 801, 557.

5-(Piperidin-1-yl)pyrimidine (7):

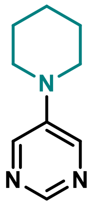

5-(Piperidin-1-yl)pyrimidine was prepared according to **GP-1**, using Zn dust (4.0 mg, 20 mol%), Ni($^{4\text{-}tBu}$stb)$_3$ (28.1 mg, 10 mol%), DABCO (60.8 mg, 0.54 mmol, 1.8 equiv.), 5-bromopyrimidine (47.7 mg, 0.3 mmol, 1.0 equiv.), and piperidine (60 µL, 0.6 mmol, 2.0 equiv.) in DMA (0.3 mL) at 60 °C. Purification *via* column chromatography (silica gel, EtOAc) afforded the title compound as a yellow oil (21.8 mg, 45%).

Spectroscopic data matched those reported in the literature.[7]

***R$_f$*:** 0.38 (EtOAc)

^{1}H NMR (300 MHz, CDCl$_3$) δ 8.62 (s, 1H), 8.34 (s, 2H), 3.21 (t, J = 5.2 Hz, 4H), 1.78 – 1.54 (m, 6H).

^{13}C NMR (75 MHz, CDCl$_3$) δ 149.0, 144.9, 143.8, 48.7, 25.3, 24.0.

3-(Piperidin-1-yl)quinoline (8):

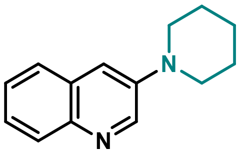

3-(Piperidin-1-yl)quinoline was prepared according to **GP-1**, using Zn dust (4.0 mg, 20 mol%), Ni($^{4\text{-}tBu}$stb)$_3$ (28.2 mg, 10 mol%), DABCO (61.0 mg, 0.54 mmol, 1.8 equiv.), 3-bromoquinoline (41 µL, 0.3 mmol, 1.0 equiv.), and piperidine (60 µL, 0.6 mmol, 2.0 equiv.) in DMA (0.3 mL) at 60 °C. Purification *via* column chromatography (silica gel, 80:20 hexanes/EtOAc) afforded the title compound as a yellow oil (50.0 mg, 78%).

***R$_f$*:** 0.30 (80:20 hexanes/EtOAc)

^{1}H NMR (400 MHz, CDCl$_3$) δ 8.80 (d, J = 2.9 Hz, 1H), 8.01 – 7.94 (m, 1H), 7.69 – 7.62 (m, 1H), 7.53 – 7.40 (m, 2H), 7.34 (dd, J = 2.8, 0.8 Hz, 1H), 3.31 – 3.24 (m, 4H), 1.84 – 1.74 (m, 4H), 1.69 – 1.59 (m, 2H).

^{13}C NMR (101 MHz, CDCl$_3$) δ 145.84, 145.81, 142.7, 129.1, 129.0, 126.9, 126.6, 126.2, 116.8, 50.7, 25.8, 24.2.

HRMS (EI) m/z: [M] calc'd for $C_{14}H_{16}N_2$: 212.1308, found: 212.1309

FTIR (ATR): $\tilde{\nu}$ [cm^{-1}] = 3059, 2932, 2851, 2802, 1592, 1428, 1383, 1352, 1273, 1259, 1229, 1205, 1118, 976, 926, 875, 848, 781, 747, 614.

7-(Piperidin-1-yl)quinoline (9):

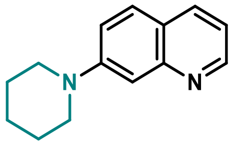

7-(Piperidin-1-yl)quinoline was prepared according to **GP-1**, using Zn dust (4.0 mg, 20 mol%), Ni($^{4\text{-}tBu}$stb)$_3$ (28.3 mg, 10 mol%), DABCO (60.7 mg, 0.54 mmol, 1.8 equiv.), 7-bromoquinoline (62.9, 0.3 mmol, 1.0 equiv.), and piperidine (60 µL, 0.6 mmol, 2.0 equiv.) in DMA (0.3 mL) at 60 °C. Purification *via* column chromatography (silica gel, 80:20 hexanes/EtOAc to 50:50 hexanes/EtOAc) afforded the title compound as a yellow solid (37.6 mg, 59%).

m.p.: 111.7–112.7 °C

***R$_f$*:** 0.43 (50:50 hexanes/EtOAc)

^{1}H NMR (300 MHz, CDCl$_3$) δ 8.75 (dd, *J* = 4.3, 1.8 Hz, 1H), 7.97 (ddd, *J* = 8.2, 1.8, 0.8 Hz, 1H), 7.64 (d, *J* = 8.9 Hz, 1H), 7.39 – 7.27 (m, 2H), 7.15 (dd, *J* = 8.1, 4.3 Hz, 1H), 3.40 – 3.30 (m, 4H), 1.80 – 1.70 (m, 4H), 1.69 – 1.59 (m, 2H).

^{13}C NMR (101 MHz, CDCl$_3$) δ 152.8, 150.6, 150.2, 135.5, 128.2, 122.5, 120.0, 118.3, 110.8, 50.2, 25.7, 24.5.

HRMS (EI) m/z: [M] calc'd for $C_{14}H_{16}N_2$: 212.1308, found: 212.1308

FTIR (ATR): $\tilde{\nu}$ [cm^{-1}] = 2932, 2919, 2849, 2814, 1613, 1495, 1444, 1382, 1335, 1245, 1204, 1195, 1112, 934, 833, 767, 672.

3-(Piperidin-1-yl)-1,5-naphthyridine (10):

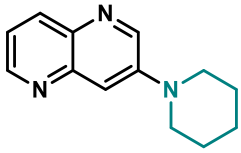

3-(Piperidin-1-yl)-1,5-naphthyridine was prepared according to **GP-1**, using Zn dust (4.0 mg, 20 mol%), Ni($^{4\text{-}tBu}$stb)$_3$ (28.1 mg, 10 mol%), DABCO (60.7 mg, 0.54 mmol, 1.8 equiv.), 3-bromo-1,5-naphthyridine (62.7 mg, 0.3 mmol, 1.0 equiv.), and piperidine (60 µL, 0.6 mmol, 2.0 equiv.) in DMA (0.3 mL) at 60 °C. Purification *via* column chromatography (silica gel, 50:50 hexanes/EtOAc) afforded the title compound as a yellow solid (51.3 mg, 80%).

m.p.: 129.9–130.9 °C

***R$_f$*:** 0.36 (50:50 hexanes/EtOAc)

^{1}H NMR (400 MHz, CDCl$_3$) δ 8.84 (dd, *J* = 2.9, 0.9 Hz, 1H), 8.81 (dt, *J* = 4.3, 1.4 Hz, 1H), 8.23 (ddt, *J* = 8.3, 1.7, 0.9 Hz, 1H), 7.53 (d, *J* = 2.6 Hz, 1H), 7.37 (ddd, *J* = 8.4, 4.3, 1.1 Hz, 1H), 3.39 – 3.34 (m, 4H), 1.82 – 1.73 (m, 4H), 1.71 – 1.62 (m, 2H).

^{13}C NMR (101 MHz, CDCl$_3$) δ 151.3, 148.3, 145.6, 145.4, 137.2, 136.7, 121.0, 116.6, 49.9, 25.5, 24.2.

HRMS (EI) m/z: [M] calc'd for $C_{13}H_{15}N_3$: 213.1260, found: 213.1260

FTIR (ATR): $\tilde{\nu}$ [cm^{-1}] = 3058, 2928, 2849, 1589, 1438, 1404, 1350, 1277, 1259, 1239, 1198, 1121, 871, 855, 810, 761, 666, 605.

8-(Piperidin-1-yl)-[1,2,4]triazolo[1,5-*a*]pyridine (11):

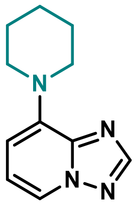

8-(Piperidin-1-yl)-[1,2,4]triazolo[1,5-*a*]pyridine was prepared according to **GP-1**, using Zn dust (4.0 mg, 20 mol%), Ni($^{4\text{-}t\text{Bu}}$stb)$_3$ (28.2 mg, 10 mol%), DABCO (60.8 mg, 0.54 mmol, 1.8 equiv.), 8-bromo-[1,2,4]triazolo[1,5-*a*]pyridine (59.4 mg, 0.3 mmol, 1.0 equiv.), and piperidine (60 µL, 0.6 mmol, 2.0 equiv.) in DMA (0.3 mL) at 60 °C. Purification *via* column chromatography (silica gel, 6:4 hexanes/EtOAc) afforded the title compound as a colorless oil (30.6 mg, 50%).

***R$_f$*:** 0.48 (6:4 hexanes/EtOAc)

^{1}H NMR (400 MHz, CDCl$_3$) δ 8.25 (s, 1H), 8.16 (dd, *J* = 6.7, 1.0 Hz, 1H), 6.86 (dd, *J* = 7.7, 6.6 Hz, 1H), 6.66 (dd, *J* = 7.8, 1.0 Hz, 1H), 3.51 – 3.44 (m, 4H), 1.80 (p, *J* = 5.9 Hz, 4H), 1.68 – 1.61 (m, 2H).

^{13}C NMR (101 MHz, CDCl$_3$) δ 151.9, 146.5, 141.8, 120.3, 114.4, 110.6, 50.6, 25.8, 24.5.

HRMS (EI) m/z: [M] calc'd for $C_{11}H_{14}N_4$: 202.1213, found: 202.1214

FTIR (ATR): $\tilde{\nu}$ [cm^{-1}] =3107, 2931, 2852, 2814, 1546, 1497, 1449, 1391, 1304, 1265, 1251, 1181, 1121, 1089,1009, 734.

***tert*-Butyl 3-(piperidin-1-yl)-1*H*-indole-1-carboxylate (12):**

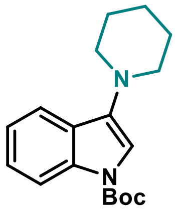

tert-Butyl 3-(piperidin-1-yl)-1*H*-indole-1-carboxylate was prepared according to **GP-1**, using Zn dust (3.9 mg, 20 mol%), Ni($^{4\text{-}t\text{Bu}}$stb)$_3$ (28.1 mg, 10 mol%), DABCO (60.6 mg, 0.54 mmol, 1.8 equiv.), *tert*-butyl 3-bromo-1*H*-indole-1-carboxylate (88.9 mg, 0.3 mmol, 1.0 equiv.), and piperidine (60 µL, 0.6 mmol, 2.0 equiv.) in DMA (0.3 mL) at 60 °C. Purification *via* column chromatography (silica gel, 98:2 hexanes/EtOAc) afforded the title compound as a yellowish oil (85.1 mg, 94%).

***R$_f$*:** 0.29 (98:2 hexanes/EtOAc))

^{1}H NMR (400 MHz, CDCl$_3$) δ 8.17 – 8.10 (m, 1H), 7.60 (dt, *J* = 7.8, 1.0 Hz, 1H), 7.31 (ddd, *J* = 8.4, 7.2, 1.3 Hz, 1H), 7.22 (ddd, *J* = 8.2, 7.2, 1.1 Hz, 1H), 7.00 (d, *J* = 14.3 Hz, 1H), 3.09 – 3.02 (m, 4H), 1.81 (p, *J* = 5.8 Hz, 4H), 1.68 (s, 9H), 1.65 – 1.58 (m, 2H).

^{13}C NMR (101 MHz, CDCl$_3$) δ 150.0, 136.5, 135.0, 126.3, 124.5, 122.0, 119.5, 115.5, 110.2, 83.1, 53.3, 28.3, 25.9, 24.4.

HRMS (EI) m/z: [M] calc'd for $C_{18}H_{24}N_2O_2$: 300.1833, found: 300.1833

FTIR (ATR): $\tilde{\nu}$ [cm^{-1}] = 2977, 2934, 2854, 2798, 1720, 1451, 1368, 1295, 1251, 1229, 1152, 1116, 1047, 1004, 908, 764, 728.

3-(Piperidin-1-yl)-1-tosyl-1*H*-pyrrolo[2,3-*b*]pyridine (13):

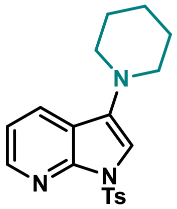

3-(Piperidin-1-yl)-1-tosyl-1*H*-pyrrolo[2,3-*b*]pyridine was prepared according to **GP-1**, using Zn dust (4.0 mg, 20 mol%), Ni($^{4\text{-}t\text{Bu}}$stb)$_3$ (28.2 mg, 10 mol%), DABCO (60.7 mg, 0.54 mmol, 1.8 equiv.), **SM2** (105.7 mg, 0.3 mmol, 1.0 equiv.), and piperidine (60 µL, 0.6 mmol, 2.0 equiv.) in DMA (0.3 mL) at 60 °C. Purification *via* column chromatography (silica gel, DCM to 100:2 DCM/MeOH) afforded the title compound as a beige-yellowish solid (61.5 mg, 57%).

m.p.: 135.3–136.3 °C

***R$_f$*:** 0.68 (100:2 DCM/MeOH)

^{1}H NMR (400 MHz, CDCl$_3$) δ 8.40 (dd, *J* = 4.8, 1.6 Hz, 1H), 8.01 – 7.93 (m, 2H), 7.84 (dd, *J* = 7.9, 1.6 Hz, 1H), 7.23 – 7.19 (m, 2H), 7.12 (dd, *J* = 7.9, 4.8 Hz, 1H), 7.08 (s, 1H), 3.05 – 2.98 (m, 4H), 2.34 (s, 3H), 1.76 (p, *J* = 5.6 Hz, 4H), 1.65 – 1.55 (m, 2H).

^{13}C NMR (101 MHz, CDCl$_3$) δ 147.2, 145.1, 144.8, 135.7, 135.0, 129.6, 128.5, 127.8, 119.1, 118.2, 110.3, 52.9, 25.9, 24.3, 21.7.

HRMS (ESI) m/z: [M+H]$^+$ calc'd for $C_{19}H_{22}N_3O_2S$: 356.1427, found: 356.1427

FTIR (ATR): $\tilde{\nu}$ [cm^{-1}] = 3167, 2929, 2853, 2804, 1585, 1400, 1363, 1163, 1143, 1098, 754, 669, 589, 574, 537, 500.

1-(Benzofuran-3-yl)piperidine (14):

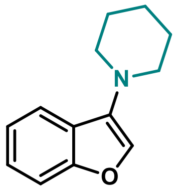

1-(Benzofuran-3-yl)piperidine was prepared according to **GP-1**, using Zn dust (3.9 mg, 20 mol%), Ni($^{4\text{-}t\text{Bu}}$stb)$_3$ (28.1 mg, 10 mol%), DABCO (60.8 mg, 0.54 mmol, 1.8 equiv.), 3-bromobenzofuran (59.1 mg, 0.3 mmol, 1.0 equiv.), and piperidine (60 µL, 0.6 mmol, 2.0 equiv.) in DMA (0.3 mL) at 60 °C. Purification *via* column chromatography (silica gel, 90:10 hexanes/EtOAc) afforded the title compound as a colorless-yellowish oil (49.0 mg, 81%).

***R*$_f$:** 0.48 (90:10 hexanes/EtOAc)

^{1}H NMR (300 MHz, CDCl$_3$) δ 7.66 – 7.57 (m, 1H), 7.41 (ddd, J = 8.2, 1.2, 0.7 Hz, 1H), 7.30 – 7.24 (m, 1H), 7.23 – 7.17 (m, 1H), 7.16 (s, 1H), 3.10 – 3.00 (m, 4H), 1.79 (p, J = 5.7 Hz, 4H), 1.68 – 1.54 (m, 2H).

^{13}C NMR (101 MHz, CDCl$_3$) δ 155.0, 136.7, 129.8, 124.4, 124.0, 121.9, 120.3, 111.9, 52.9, 25.9, 24.4.

HRMS (EI) m/z: [M] calc'd for $C_{13}H_{15}NO$: 201.1148, found: 201.1151

FTIR (ATR): $\tilde{\nu}$ [cm^{-1}] = 2934, 2853, 2802, 1575, 1454, 1373, 1348, 1275, 1260, 1205, 1119, 1097, 1016, 855, 739.

1-(Benzofuran-2-yl)piperidine (15):

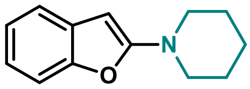

1-(Benzofuran-2-yl)piperidine was prepared according to **GP-1**, using Zn dust (4.0 mg, 20 mol%), Ni($^{4\text{-}t\text{Bu}}$stb)$_3$ (28.3 mg, 10 mol%), DABCO (60.8 mg, 0.54 mmol, 1.8 equiv.), 2-bromobenzofuran (37 µL, 0.3 mmol, 1.0 equiv.), and piperidine (60 µL, 0.6 mmol, 2.0 equiv.) in DMA (0.3 mL) at 60 °C. As several attempts of isolation failed, 1,3,5-trimethoxybenzene (16.9 mg, 0.10 mmol, 0.333 equiv.) was added after aqueous workup and the yield was determined *via* ^{1}H NMR analysis to be 59%. Furthermore, **15** was detected *via* HRMS (EI).

Spectroscopic data matches those reported in the literature.[8]

^{1}H NMR (300 MHz, CDCl$_3$) δ 7.27 – 7.18 (m, 2H), 7.09 – 7.03 (m, 1H), 6.96 (ddd, J = 7.9, 7.3, 1.4 Hz, 1H), 5.37 (d, J = 0.8 Hz, 1H), 3.30 – 3.20 (m, 4H), 1.73 – 1.64 (m, 4H), 1.64 – 1.55 (m, 2H)

HRMS (EI) m/z: [M] calc'd for $C_{13}H_{15}NO$: 201.1148, found: 201.1152

Ethyl 5-(piperidin-1-yl)furan-2-carboxylate (16):

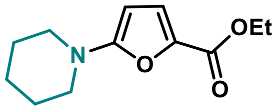

Ethyl 5-(piperidin-1-yl)furan-2-carboxylate was prepared according to **GP-1**, using Zn dust (3.9 mg, 20 mol%), Ni($^{4\text{-}t\text{Bu}}$stb)$_3$ (28.1 mg, 10 mol%), DABCO (60.7 mg, 0.54 mmol, 1.8 equiv.), ethyl 5-bromofuran-2-carboxylate (43 µL, 0.3 mmol, 1.0 equiv.), and piperidine (60 µL, 0.6 mmol, 2.0 equiv.) in DMA (0.3 mL) at 60 °C. Purification *via* column chromatography (silica gel, 90:10 hexanes/EtOAc) afforded the title compound as a yellow-orangish oil (47.5 mg, 71%).

***R*$_f$:** 0.27 (90:10 hexanes/EtOAc)

^{1}H NMR (400 MHz, CDCl$_3$) δ 7.13 (d, J = 3.7 Hz, 1H), 5.14 (d, J = 3.7 Hz, 1H), 4.28 (q, J = 7.1 Hz, 2H), 3.29 – 3.24 (m, 4H), 1.69 – 1.55 (m, 6H), 1.32 (t, J = 7.1 Hz, 3H).

^{13}C NMR (101 MHz, CDCl$_3$) δ 162.3, 158.9, 135.1, 122.8, 85.1, 60.1, 47.6, 25.0, 24.1, 14.7.

HRMS (ESI) m/z: [M+Na]$^+$ calc'd for $C_{12}H_{17}NO_3Na$: 246.1101, found: 246.1101

FTIR (ATR): $\tilde{\nu}$ [cm^{-1}] =2934, 2853, 1702, 1588, 1534, 1450, 1367, 1303, 1254, 1110, 1054, 1013, 975, 883, 734.

1-(5-Phenylthiophen-2-yl)piperidine (17):

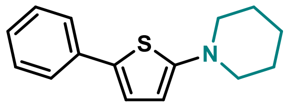

1-(5-Phenylthiophen-2-yl)piperidine was prepared according to **GP-1**, using Zn dust (4.1 mg, 21 mol%), Ni($^{4\text{-}t\text{Bu}}$stb)$_3$ (28.0 mg, 10 mol%), DABCO (60.9 mg, 0.54 mmol, 1.8 equiv.), 2-bromo-5-phenylthiophene (72.0, 0.3 mmol, 1.0 equiv.), and piperidine (60 µL, 0.6 mmol, 2.0 equiv.) in DMA (0.3 mL) at 60 °C. Purification *via* column chromatography (silica gel, 98:2 hexanes/EtOAc) afforded the title compound as a yellowish solid (32.5 mg, 44%).

m.p.: 68.2–69.2 °C

R**$_f$:** 0.40 (98:2 hexanes/EtOAc)

^{1}H NMR (400 MHz, $CDCl_3$) δ 7.54 – 7.45 (m, 2H), 7.34 – 7.29 (m, 2H), 7.19 – 7.14 (m, 1H), 7.04 (d, *J* = 3.9 Hz, 1H), 6.08 (d, *J* = 4.0 Hz, 1H), 3.22 – 3.14 (m, 4H), 1.75 (p, *J* = 5.7 Hz, 4H), 1.64 – 1.53 (m, 2H).

^{13}C NMR (101 MHz, $CDCl_3$) δ 159.5, 135.3, 129.8, 128.8, 126.0, 124.6, 122.4, 105.7, 52.6, 25.4, 23.9.

HRMS (EI) m/z: [M] calc'd for $C_{15}H_{17}NS$: 243.1076, found: 243.1079

FTIR (ATR): $\tilde{\nu}$ [cm^{-1}] = 3052, 3021, 2925, 2852, 2818, 1495, 1477, 1460, 1442, 1379, 1238, 1193, 1119, 885, 861, 772, 748, 687, 608, 477.

1-(4-(Trifluoromethyl)phenyl)piperidine (18):

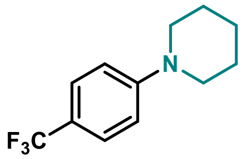

1-(4-(Trifluoromethyl)phenyl)piperidine was prepared according to **GP-1**, using Zn dust (4.0 mg, 20 mol%), Ni($^{4\text{-}t\text{Bu}}$stb)$_3$ (28.1 mg, 10 mol%), DABCO (60.7 mg, 0.54 mmol, 1.8 equiv.), 4-bromobenzotrifluoride (42 µL, 0.3 mmol, 1.0 equiv.), and piperidine (60 µL, 0.6 mmol, 2.0 equiv.) in DMA (0.3 mL) at 60 °C. Purification *via* column chromatography (silica gel, 96:4 hexanes/EtOAc) afforded the title compound as a colorless oil (61.6 mg, 90%).

Spectroscopic data matched those reported in the literature.[9]

R**$_f$:** 0.43 (96:4 hexanes/EtOAc)

^{1}H NMR (400 MHz, $CDCl_3$) δ 7.48 – 7.44 (m, 2H), 6.96 – 6.87 (m, 2H), 3.31 – 3.23 (m, 4H), 1.74 – 1.61 (m, 6H).

^{13}C NMR (101 MHz, $CDCl_3$) δ 153.9, 126.5 (q, *J* = 3.7 Hz), 125.0 (d, *J* = 270.5 Hz), 119.7 (q, *J* = 32.6 Hz), 114.7, 49.5, 25.6, 24.4.

^{19}F NMR (282 MHz, CDCl$_3$) δ −61.2.

1-(4-(Methylsulfonyl)phenyl)piperidine (19):

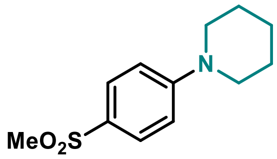

1-(4-(Methylsulfonyl)phenyl)piperidine was prepared according to **GP-1**, using Zn dust (4.0 mg, 20 mol%), Ni($^{4\text{-}t\text{Bu}}$stb)$_3$ (28.1 mg, 10 mol%), DABCO (60.7 mg, 0.54 mmol, 1.8 equiv.), 4-bromophenyl methyl sulfone (70.5 mg, 0.3 mmol, 1.0 equiv.), and piperidine (60 µL, 0.6 mmol, 2.0 equiv.) in DMA (0.3 mL) at 60 °C. Purification *via* column chromatography (silica gel, 80:20 pentane/EtOAc to 50:50 pentane/EtOAc) afforded the title compound as a white solid (63.0 mg, 88%).

Spectroscopic data matched those reported in the literature.[9]

m.p.: 116.1–117.1 °C

***R$_f$*:** 0.52 (90:10 hexanes/EtOAc)

^{1}H NMR (300 MHz, CDCl$_3$) δ 7.76 – 7.70 (m, 2H), 6.93 – 6.87 (m, 2H), 3.36 (t, *J* = 4.7 Hz, 4H), 3.00 (s, 3H), 1.73 – 1.63 (m, 6H).

^{13}C NMR (75 MHz, CDCl$_3$) δ 154.7, 129.2, 127.5, 113.8, 48.7, 45.1, 25.4, 24.4.

4-(Piperidin-1-yl)benzamide (20):

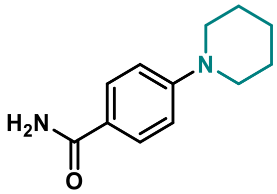

4-(Piperidin-1-yl)benzamide was prepared according to **GP-1**, using Zn dust (4.0 mg, 20 mol%), Ni($^{4\text{-}t\text{Bu}}$stb)$_3$ (28.1 mg, 10 mol%), DABCO (60.7 mg, 0.54 mmol, 1.8 equiv.), 4-bromobenzamide (60.0 mg, 0.3 mmol, 1.0 equiv.), and piperidine (60 µL, 0.6 mmol, 2.0 equiv.) in DMA (0.3 mL) at 60 °C. Purification was performed as follows: The reaction was allowed to cool down to room temperature after stirring for 24 h. The reaction was diluted with EtOAc (2 mL), and quenched by addition of water (0.1 mL). An aqueous solution of HCl (1M) was added (pH = 3-4), and the aqueous layer was washed with EtOAc (4 x 15 mL). A saturated aqueous solution of $NaHCO_3$ was added to the aqueous layer (pH = 8), followed by extraction with EtOAc (4 x 15 ml). The combined organic layers were dried over $MgSO_4$ and the solvent was removed under reduced pressure to afford the title compound as a white solid (40.3 mg, 66%).

Spectroscopic data matched those reported in the literature.[9]

m.p.: 233.1–234.1 °C

***R$_f$*:** 0.49 (EtOAc)

^{1}H NMR (400 MHz, DMSO-d_6) δ 7.72 (d, J = 8.9 Hz, 2H), 7.65 (s, 1H), 6.96 (s, 1H), 6.90 (d, J = 9.0 Hz, 2H), 3.30 – 3.22 (m, 4H), 1.63 – 1.54 (m, 6H).

^{13}C NMR (101 MHz, DMSO-d_6) δ 167.7, 153.0, 128.9, 122.8, 113.5, 48.3, 24.9, 24.0.

3-(Piperidin-1-yl)benzonitrile (21):

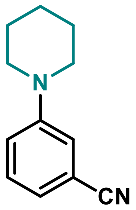

3-(Piperidin-1-yl)benzonitrile was prepared according to **GP-1**, using Zn dust (4.1 mg, 21 mol%), Ni($^{4\text{-}t\text{Bu}}$stb)$_3$ (28.1 mg, 10 mol%), DABCO (60.9 mg, 0.54 mmol, 1.8 equiv.), 3-bromobenzonitrile (54.8 mg, 0.3 mmol, 1.0 equiv.), and piperidine (60 µL, 0.6 mmol, 2.0 equiv.) in DMA (0.3 mL) at 60 °C. Purification *via* preparative TLC (hexanes to 90:10 hexanes/EtOAc) afforded the title compound as a colorless-yellowish oil (40.1 mg, 72%).

Spectroscopic data matched those reported in the literature.[10]

***R$_f$*:** 0.52 (90:10 hexanes/EtOAc)

^{1}H NMR (400 MHz, CDCl$_3$) δ 7.31 – 7.26 (m, 1H), 7.12 – 7.08 (m, 2H), 7.03 (dt, J = 7.5, 1.2 Hz, 1H), 3.23 – 3.16 (m, 4H), 1.75 – 1.66 (m, 4H), 1.64 – 1.57 (m, 2H).

^{13}C NMR (101 MHz, CDCl$_3$) δ 152.1, 129.9, 121.9, 120.3, 119.7, 118.7, 113.0, 49.8, 25.6, 24.2.

1-(*p*-Tolyl)piperidine (22):

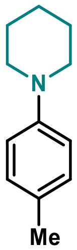

1-(*p*-Tolyl)piperidine was prepared according to **GP-1**, using Zn dust (4.0 mg, 20 mol%), Ni($^{4\text{-}t\text{Bu}}$stb)$_3$ (28.2 mg, 10 mol%), quinuclidine (60.2 mg, 0.54 mmol, 1.8 equiv.), 4-bromotoluene (37 µL, 0.3 mmol, 1.0 equiv.), and piperidine (60 µL, 0.6 mmol, 2.0 equiv.) in DMA (0.3 mL) at 60 °C. 1,3,5-trimethoxybenzene (16.8 mg, 0.10 mmol, 0.332 equiv.) was added after aqueous workup and the yield was determined *via* ^{1}H NMR analysis to be 25%. Furthermore, **22** was detected *via* HRMS (EI).

Spectroscopic data matches those reported in the literature.[9]

^{1}H NMR (300 MHz, CDCl$_3$) δ 7.01 (d, J = 3.3 Hz, 2H), 6.84 (d, J = 8.5 Hz, 2H), 3.10 – 3.03 (m, 4H), 2.24 (s, 3H), 1.69 (p, J = 5.6 Hz, 4H), 1.58 – 1.50 (m, 2H).

HRMS (EI) m/z: [M] calc'd for $C_{12}H_{17}N$: 175.1355, found: 175.1353

Ethyl 1-(pyridin-3-yl)piperidine-4-carboxylate (23):

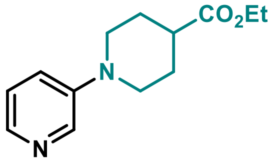

Ethyl 1-(pyridin-3-yl)piperidine-4-carboxylate was prepared according to **GP-1**, using Zn dust (4.0 mg, 20 mol%), Ni($^{4\text{-}t\text{Bu}}$stb)$_3$ (28.2 mg, 10 mol%), DABCO (60.9 mg, 0.54 mmol, 1.8 equiv.), 3-bromopyridine (29 µL, 0.3 mmol, 1.0 equiv.), and ethyl isonipecotate (94 µL, 0.6 mmol, 2.0 equiv.) in DMA (0.3 mL) at 60 °C. Purification *via* column chromatography (silica gel, EtOAc) afforded the title compound as an orange/yellowish oil (47.4 mg, 67%).

***R_f*:** 0.54 (EtOAc)

^{1}H NMR (300 MHz, CDCl$_3$) δ 8.30 (s, 1H), 8.07 (s, 1H), 7.28 – 7.17 (m, 2H), 4.16 (q, *J* = 7.1 Hz, 2H), 3.71 – 3.61 (m, 2H), 2.95 – 2.80 (m, 2H), 2.55 – 2.39 (m, 1H), 2.11 – 1.98 (m, 2H), 1.97 – 1.77 (m, 2H), 1.27 (t, *J* = 7.1 Hz, 3H).

^{13}C NMR (75 MHz, CDCl$_3$) δ 174.7, 147.3, 140.2, 138.7, 123.7, 123.2, 60.7, 48.5, 40.8, 27.9, 14.3.

HRMS (EI) m/z: [M] calc'd for $C_{13}H_{18}N_2O_2$: 234.1363, found: 234.1365

FTIR (ATR): $\tilde{\nu}$ [cm^{-1}] = 2978, 2953, 2930, 2814, 1724, 1581, 1487, 1448, 1424, 1311, 1243, 1165, 1039, 1101, 1010, 915, 798, 707, 610.

8-(Pyridin-3-yl)-1,4-dioxa-8-azaspiro[4.5]decane (24):

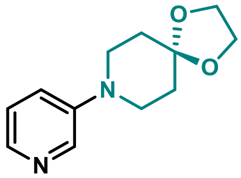

8-(Pyridin-3-yl)-1,4-dioxa-8-azaspiro[4.5]decane was prepared according to **GP-1**, using Zn dust (3.9 mg, 20 mol%), Ni($^{4\text{-}t\text{Bu}}$stb)$_3$ (28.2 mg, 10 mol%), DABCO (60.8 mg, 0.54 mmol, 1.8 equiv.), 3-bromopyridine (29 µL, 0.3 mmol, 1.0 equiv.), and 1,4-dioxa-8-azaspiro[4.5]decane (86.0 mg, 0.6 mmol, 2.0 equiv.) in DMA (0.3 mL) at 60 °C. Purification *via* column chromatography (silica gel, EtOAc) afforded the title compound as an orange oil (37.8 mg, 57%).

Spectroscopic data matches those reported in the literature.[11]

***R_f*:** 0.25 (EtOAc)

^{1}H NMR (400 MHz, CDCl$_3$) δ 8.30 (d, *J* = 2.9 Hz, 1H), 8.04 (dd, *J* = 4.5, 1.4 Hz, 1H), 7.17 (ddd, *J* = 8.5, 3.0, 1.4 Hz, 1H), 7.12 (dd, *J* = 8.4, 4.5 Hz, 1H), 3.97 (s, 4H), 3.37 – 3.29 (m, 4H), 1.85 – 1.78 (m, 4H).

^{13}C NMR (101 MHz, CDCl$_3$) δ 146.7, 140.4, 139.1, 123.6, 122.9, 106.9, 64.5, 47.2, 34.4.

3-(Pyrrolidin-1-yl)pyridine (25):

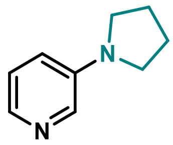

3-(Pyrrolidin-1-yl)pyridine was prepared according to **GP-1**, using Zn dust (4.0 mg, 20 mol%), Ni($^{4-tBu}$stb)$_3$ (28.2 mg, 10 mol%), DABCO (60.9 mg, 0.54 mmol, 1.8 equiv.), 3-bromopyridine (29 µL, 0.3 mmol, 1.0 equiv.), and pyrrolidine (50 µL, 0.6 mmol, 2.0 equiv.) in DMA (0.3 mL) at 60 °C. Purification *via* column chromatography (silica gel, EtOAc) afforded the title compound as an orange oil (26.8 mg, 60%).

Spectroscopic data matches those reported in the literature.[6]

***R$_f$*:** 0.32 (EtOAc)

^{1}H NMR (400 MHz, CDCl$_3$) δ 7.99 (dd, *J* = 3.0, 0.7 Hz, 1H), 7.92 (dd, *J* = 4.6, 1.4 Hz, 1H), 7.10 (ddd, *J* = 8.4, 4.6, 0.7 Hz, 1H), 6.81 (ddd, *J* = 8.5, 3.0, 1.3 Hz, 1H), 3.36 – 3.23 (m, 4H), 2.09 – 1.96 (m, 4H).

^{13}C NMR (101 MHz, CDCl$_3$) δ 143.9, 137.0, 134.5, 123.7, 117.9, 47.4, 25.5.

3-(2,5-Dihydro-1*H*-pyrrol-1-yl)pyridine (26):

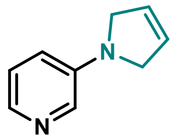

3-(2,5-Dihydro-1*H*-pyrrol-1-yl)pyridine was prepared according to **GP-1**, using Zn dust (4.0 mg, 20 mol%), Ni($^{4-tBu}$stb)$_3$ (28.2 mg, 10 mol%), DABCO (60.9 mg, 0.54 mmol, 1.8 equiv.), 3-bromopyridine (29 µL, 0.3 mmol, 1.0 equiv.), and 3-pyrroline (46 µL, 0.6 mmol, 2.0 equiv.) in DMA (0.3 mL) at 60 °C. Purification *via* column chromatography (silica gel, 50:50 hexanes/EtOAc) resulted in partial oxidation and afforded the title compound (26.5 mg, 60%) together with 3-(1*H*-pyrrol-1-yl)pyridine (ca. 4%) as an inseparable mixture as an orange oil.

***R$_f$*:** 0.16 (50:50 hexanes/EtOAc)

^{1}H NMR (400 MHz, CDCl$_3$) δ 7.98 – 7.93 (m, 2H), 7.13 (dd, *J* = 8.4, 4.6 Hz, 1H), 6.78 (ddd, *J* = 8.4, 3.0, 1.3 Hz, 1H), 5.96 (s, 2H), 4.12 (s, 4H).

^{13}C NMR (101 MHz, CDCl$_3$) δ 143.0, 137.1, 133.8, 126.2, 123.8, 117.6, 54.3.

HRMS (EI) m/z: [M] calc'd for $C_9H_{10}N_2$: 146.0838, found: 146.0840

FTIR (ATR): $\tilde{\nu}$ [cm^{-1}] = 3385, 3081, 3036, 2939, 2821, 1582, 1491, 1472, 1432, 1372, 1357, 1247, 1176, 1004, 946, 790, 709, 672, 615.

4-(Pyridin-3-yl)morpholine (27):

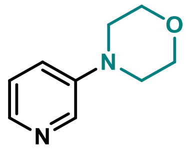

4-(Pyridin-3-yl)morpholine was prepared according to **GP-1**, using Zn dust (3,9 mg, 20 mol%), Ni($^{4\text{-}t\text{Bu}}$stb)$_3$ (28.2 mg, 10 mol%), DABCO (60.9 mg, 0.54 mmol, 1.8 equiv.), 3-bromopyridine (29 µL, 0.3 mmol, 1.0 equiv.), and morpholine (54 µL, 0.6 mmol, 2.1 equiv.) in DMA (0.3 mL) at 60 °C. Purification *via* column chromatography (silica gel, EtOAc) afforded the title compound as an orange oil (43.9 mg, 89%).

Spectroscopic data matches those reported in the literature.[12]

***R$_f$*:** 0.2 (EtOAc)

^{1}H NMR (300 MHz, CDCl$_3$) *δ* 8.31 (t, *J* = 1.9 Hz, 1H), 8.13 (dd, *J* = 3.5, 2.5 Hz, 1H), 7.23 – 7.14 (m, 2H), 3.94 – 3.76 (m, 4H), 3.28 – 3.13 (m, 4H).

^{13}C NMR (101 MHz, CDCl$_3$) *δ* 147.1, 141.2, 138.4, 123.7, 122.3, 66.8, 48.7.

***tert*-Butyl 4-(pyridin-3-yl)piperazine-1-carboxylate (28):**

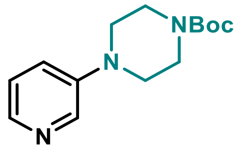

tert-Butyl 4-(pyridin-3-yl)piperazine-1-carboxylate was prepared according to **GP-1**, using Zn dust (4.0 mg, 20 mol%), Ni($^{4\text{-}t\text{Bu}}$stb)$_3$ (28.2 mg, 10 mol%), DABCO (60.8 mg, 0.54 mmol, 1.8 equiv.), 3-bromopyridine (29 µL, 0.3 mmol, 1.0 equiv.), and *tert*-butyl piperazine-1-carboxylate (112.2 mg, 0.6 mmol, 2.0 equiv.) in DMA (0.3 mL) at 60 °C. Purification *via* column chromatography (silica gel, EtOAc) afforded the title compound as a beige solid (63.7 mg, 80%).

m.p.: 58.7–59.7 °C

***R$_f$*:** 0.41 (EtOAc)

^{1}H NMR (400 MHz, CDCl$_3$) *δ* 8.31 (dd, *J* = 2.4, 1.3 Hz, 1H), 8.25 – 8.10 (m, 1H), 7.19 – 7.17 (m, 2H), 3.62 – 3.55 (m, 4H), 3.20 – 3.14 (m, 4H), 1.48 (s, 9H).

^{13}C NMR (101 MHz, CDCl$_3$) *δ* 154.8, 147.1, 141.5, 139.3, 123.7, 123.1, 80.2, 48.9, 43.6, 28.6.

HRMS (EI) m/z: [M] calc'd for $C_{14}H_{21}N_3O_2$: 263.1628, found: 263.1631

FTIR (ATR): $\tilde{\nu}$ [cm^{-1}] = 3002, 2978, 2923, 2852, 2828, 1673, 1580, 1482, 1414, 1389, 1363, 1285, 1266, 1234, 1159, 1120, 1087, 1040, 1001, 924, 798, 706, 610.

2-(Pyridin-3-yl)-1,2,3,4-tetrahydroisoquinoline (29):

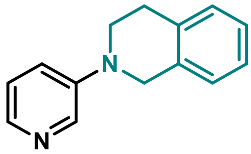

2-(Pyridin-3-yl)-1,2,3,4-tetrahydroisoquinoline was prepared according to **GP-1**, using Zn dust (4.0 mg, 20 mol%), Ni($^{4\text{-}t\text{Bu}}$stb)$_3$ (28.2 mg, 10 mol%), DABCO (60.8 mg, 0.54 mmol, 1.8 equiv.), 3-bromopyridine (29 µL, 0.3 mmol, 1.0 equiv.), and 1,2,3,4-tetrahydroisoquinoline (75 µL, 0.6 mmol, 2.0 equiv.) in DMA (0.3 mL) at 60 °C. Purification *via* column chromatography (silica gel, Et_2O) afforded the title compound as an orange oil (37.8 mg, 60%).

Spectroscopic data matches those reported in the literature.[13]

***R_f*:** 0.40 (Et_2O)

^{1}H NMR (400 MHz, $CDCl_3$) δ 8.37 (d, *J* = 2.9 Hz, 1H), 8.07 (dd, *J* = 4.5, 1.6 Hz, 1H), 7.25 – 7.11 (m, 6H), 4.43 (s, 2H), 3.59 (t, *J* = 5.9 Hz, 2H), 3.00 (t, *J* = 5.9 Hz, 2H).

^{13}C NMR (101 MHz, $CDCl_3$) δ 146.2, 139.5, 137.3, 134.7, 133.8, 128.6, 126.8, 126.6, 126.4, 123.7, 121.2, 49.9, 45.8, 28.9.

2-(Pyridin-3-yl)octahydro-1*H*-isoindole (30):

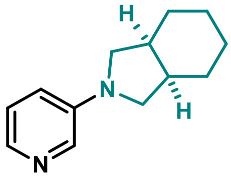

2-(Pyridin-3-yl)octahydro-1*H*-isoindole was prepared according to **GP-1**, using Zn dust (4.0 mg, 20 mol%), Ni($^{4\text{-}t\text{Bu}}$stb)$_3$ (28.2 mg, 10 mol%), DABCO (60.9 mg, 0.54 mmol, 1.8 equiv.), 3-bromopyridine (29 µL, 0.3 mmol, 1.0 equiv.), and *cis*-octahydroisoindol (78 µL, 0.6 mmol, 2.0 equiv.) in DMA (0.3 mL) at 60 °C. Purification *via* column chromatography (silica gel, 50:50 hexanes/EtOAc) afforded the title compound as an orange oil (58.8 mg, 97%).

***R_f*:** 0.32 (50:50 hexanes/EtOAc)

^{1}H NMR (400 MHz, $CDCl_3$) δ 7.94 (d, *J* = 3.0 Hz, 1H), 7.89 (dd, *J* = 4.6, 1.3 Hz, 1H), 7.09 (ddd, *J* = 8.4, 4.6, 0.7 Hz, 1H), 6.75 (ddd, *J* = 8.4, 3.0, 1.4 Hz, 1H), 3.31 (dd, *J* = 9.0, 6.5 Hz, 2H), 3.17 (dd, *J* = 9.0, 5.1 Hz, 2H), 2.41 – 2.24 (m, 2H), 1.70 – 1.60 (m, 2H), 1.59 – 1.37 (m, 6H).

^{13}C NMR (101 MHz, $CDCl_3$) δ 144.2, 136.6, 134.0, 123.7, 117.3, 51.6, 37.4, 26.4, 23.1.

HRMS (EI) m/z: [M] calc'd for $C_{13}H_{18}N_2$: 202.1464, found: 202.1466

FTIR (ATR): $\tilde{\nu}$ [cm^{-1}] = 3035, 2921, 2851, 1583, 1493, 1480, 1447, 1430, 1371, 1246, 1168, 1002, 787, 706, 614.

tert-Butyl 5-(pyridin-3-yl)hexahydropyrrolo[3,4-_c_]pyrrole-2(1_H_)-carboxylate (31):

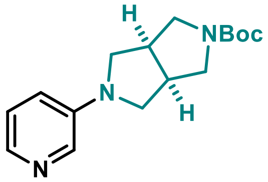

tert-Butyl 5-(pyridin-3-yl)hexahydropyrrolo[3,4-*c*]pyrrole-2(1*H*)-carboxylate was prepared according to **GP-1**, using Zn dust (4.0 mg, 20 mol%), Ni($^{4\text{-}t\text{Bu}}$stb)$_3$ (28.2 mg, 10 mol%), DABCO (60.9 mg, 0.54 mmol, 1.8 equiv.), 3-bromopyridine (29 µL, 0.3 mmol, 1.0 equiv.), and *tert*-butyl hexahydropyrrolo[3,4-*c*]pyrrole-2(1*H*)-carboxylate (127.8 mg, 0.6 mmol, 2.0 equiv.) in DMA (0.3 mL) at 60 °C. Purification *via* column chromatography (silica gel, EtOAc) afforded the title compound as a beige solid (75.7 mg, 87%).

m.p.: 107.0–108.0 °C

R**$_f$:** 0.17 (EtOAc)

^{1}H NMR (400 MHz, CDCl$_3$) δ 7.99 – 7.93 (m, 2H), 7.11 (ddd, *J* = 8.4, 4.6, 0.7 Hz, 1H), 6.80 (ddd, *J* = 8.4, 3.0, 1.4 Hz, 1H), 3.65 (dt, *J* = 12.0, 5.1 Hz, 2H), 3.54 (t, *J* = 8.6 Hz, 2H), 3.40 – 3.19 (m, 4H), 3.01 (d, *J* = 4.0 Hz, 2H), 1.45 (s, 9H).

^{13}C NMR (101 MHz, CDCl$_3$) δ 154.6, 143.6, 137.8, 134.6, 123.7, 118.4, 79.7, 51.9, 50.5, 50.2, 42.3, 41.4, 28.6.

HRMS (EI) m/z: [M] calc'd for $C_{16}H_{23}N_3O_2$: 289.1785, found: 289.1787

FTIR (ATR): $\tilde{\nu}$ [cm^{-1}] = 3053, 2967, 2923, 2890, 2852, 1692, 1582, 1493, 1479, 1403, 1365, 1341, 1248, 1169, 1128, 1096, 1002, 873, 787, 765, 706, 612, 536, 459, 419.

_N,N_-Dimethylpyridin-3-amine (32):

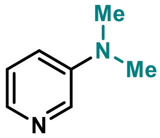

N,*N*-Dimethylpyridin-3-amine was prepared according to **GP-1**, using Zn dust (4.0 mg, 20 mol%), Ni($^{4\text{-}t\text{Bu}}$stb)$_3$ (28.2 mg, 10 mol%), DABCO (60.8 mg, 0.54 mmol, 1.8 equiv.), 3-bromopyridine (29 µL, 0.3 mmol, 1.0 equiv.), and dimethylamine (2 M in THF, 0.3 mL, 0.6 mmol, 2.0 equiv.) in DMA (0.3 mL) at 60 °C. Purification *via* column chromatography (silica gel, EtOAc) afforded the title compound as an orange oil (24.4 mg, 66%).

Spectroscopic data matches those reported in the literature.[14]

R**$_f$:** 0.37 (EtOAc)

^{1}H NMR (300 MHz, CDCl$_3$) δ 8.15 (d, *J* = 3.1 Hz, 1H), 7.98 (dd, *J* = 4.6, 1.3 Hz, 1H), 7.12 (ddd, *J* = 8.5, 4.6, 0.7 Hz, 1H), 6.98 (ddd, *J* = 8.5, 3.1, 1.3 Hz, 1H), 2.97 (s, 6H).

^{13}C NMR (75 MHz, CDCl$_3$) δ 146.3, 137.9, 135.2, 123.5, 118.8, 40.2.

***N*-Benzyl-*N*-methylpyridin-3-amine (33):**

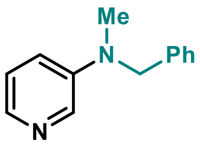

N-Benzyl-*N*-methylpyridin-3-amine was prepared according to **GP-1**, using Zn dust (3.9 mg, 20 mol%), Ni($^{4\text{-}t\text{Bu}}$stb)$_3$ (28.2 mg, 10 mol%), DABCO (60.9 mg, 0.54 mmol, 1.8 equiv.), 3-bromopyridine (29 μL, 0.3 mmol, 1.0 equiv.), and *N*-methylbenzylamine (78 μL, 0.6 mmol, 2.0 equiv.) in DMA (0.3 mL) at 60 °C. Purification *via* column chromatography (silica gel, 50:50 hexanes/EtOAc) afforded the title compound as an orange oil (35.3 mg, 59%).

Spectroscopic data matches those reported in the literature.[15]

***R$_f$*:** 0.29 (50:50 hexanes/EtOAc)

^{1}H NMR (400 MHz, CDCl$_3$) δ 8.19 (dd, *J* = 3.2, 0.7 Hz, 1H), 7.98 (dd, *J* = 4.6, 1.3 Hz, 1H), 7.35 – 7.30 (m, 2H), 7.28 – 7.23 (m, 1H), 7.24 – 7.17 (m, 2H), 7.10 (ddd, *J* = 8.5, 4.6, 0.7 Hz, 1H), 6.98 (ddd, *J* = 8.5, 3.1, 1.3 Hz, 1H), 4.55 (s, 2H), 3.06 (s, 3H).

^{13}C NMR (101 MHz, CDCl$_3$) δ 145.5, 138.1, 138.0, 135.1, 128.9, 127.3, 126.8, 123.7, 118.8, 56.3, 38.5.

***N*-Hexylpyridin-3-amine (34):**

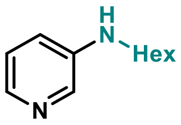

N-Hexylpyridin-3-amine was prepared according to **GP-2**, using Zn dust (2.0 mg, 10 mol%), Ni($^{4\text{-}t\text{Bu}}$stb)$_3$ (14.1 mg, 5 mol%), ^{t}Bu-TMG (0.11 mL, 0.55 mmol, 1.8 equiv.), 3-bromopyridine (29 μL, 0.3 mmol, 1.0 equiv.), and hexylamine (80 μL, 0.6 mmol, 2.0 equiv.) in DMA (0.3 mL) at 100 °C. Purification *via* column chromatography (silica gel, 50:50 hexanes/EtOAc) afforded the title compound as a white solid (51.2 mg, 95%).

Spectroscopic data matches those reported in the literature.[16]

m.p.: 51.9–52.9 °C

***R$_f$*:** 0.32 (50:50 hexanes/EtOAc)

^{1}H NMR (400 MHz, CDCl$_3$) δ 8.02 (dd, *J* = 3.0, 0.7 Hz, 1H), 7.94 (dd, *J* = 4.7, 1.4 Hz, 1H), 7.07 (ddd, *J* = 8.3, 4.7, 0.7 Hz, 1H), 6.85 (ddd, *J* = 8.3, 2.9, 1.4 Hz, 1H), 3.64 (brs, 1H), 3.11 (td, *J* = 7.1, 5.0 Hz, 2H), 1.62 (p, *J* = 7.2 Hz, 2H), 1.46 – 1.36 (m, 2H), 1.36 – 1.29 (m, 4H), 0.93 – 0.88 (m, 3H).

^{13}C NMR (101 MHz, CDCl$_3$) δ 144.6, 138.7, 136.2, 123.9, 118.5, 43.8, 31.7, 29.5, 26.9, 22.8, 14.2.

_N_-(2,2-Dimethoxyethyl)pyridin-3-amine (35):

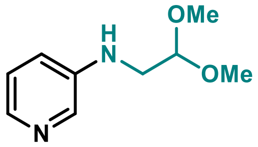

N-(2,2-Dimethoxyethyl)pyridin-3-amine was prepared according to **GP-2**, using Zn dust (2.0 mg, 10 mol%), Ni($^{4\text{-}t\text{Bu}}$stb)$_3$ (14.1 mg, 5 mol%), ^{t}Bu-TMG (0.11 mL, 0.55 mmol, 1.8 equiv.), 3-bromopyridine (29 µL, 0.3 mmol, 1.0 equiv.), and 2,2-dimethoxyethan-1-amine (65 µL, 0.6 mmol, 2.0 equiv.) in DMA (0.3 mL) at 100 °C. Purification *via* column chromatography (silica gel, 50:50 hexanes/EtOAc to EtOAc) afforded the title compound as a colorless oil (45.6 mg, 83%).

R**$_f$:** 0.38 (EtOAc)

^{1}H NMR (300 MHz, CDCl$_3$) δ 8.06 (dd, *J* = 2.9, 0.7 Hz, 1H), 7.98 (dd, *J* = 4.7, 1.4 Hz, 1H), 7.09 (ddd, *J* = 8.3, 4.7, 0.7 Hz, 1H), 6.90 (ddd, *J* = 8.3, 2.9, 1.4 Hz, 1H), 4.56 (t, *J* = 5.4 Hz, 1H), 3.92 (brs, 1H), 3.42 (s, 6H), 3.26 (t, *J* = 5.1 Hz, 2H).

^{13}C NMR (101 MHz, CDCl$_3$) δ 144.0, 139.0, 136.2, 123.8, 118.9, 102.5, 54.1, 45.0.

HRMS (EI) m/z: [M] calc'd for $C_9H_{14}N_2O_2$: 182.1050, found: 182.1052

FTIR (ATR): $\tilde{\nu}$ [cm^{-1}] = 3367, 3265, 3101, 3041, 2931, 2833, 1587, 1508, 1483, 1302, 1241, 1190, 1126, 1055, 974, 912, 793, 730, 707, 625, 542, 414.

_N_-(Furan-2-ylmethyl)pyridin-3-amine (36):

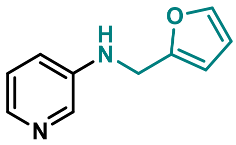

N-(Furan-2-ylmethyl)pyridin-3-amine was prepared according to **GP-2**, using Zn dust (2.0 mg, 10 mol%), Ni($^{4\text{-}t\text{Bu}}$stb)$_3$ (14.1 mg, 5 mol%), ^{t}Bu-TMG (0.11 mL, 0.55 mmol, 1.8 equiv.), 3-bromopyridine (29 µL, 0.3 mmol, 1.0 equiv.), and furfurylamine (53 µL, 0.6 mmol, 2.0 equiv.) in DMA (0.3 mL) at 100 °C. Purification *via* column chromatography (silica gel, 80:20 hexanes/EtOAc to EtOAc) afforded the title compound as a yellowish oil that freezes upon cooling to 0 °C (52.7 mg, >99%).

m.p.: not determined as the product is an oil that freezes upon cooling to 0 °C

R**$_f$:** 0.58 (EtOAc)

^{1}H NMR (400 MHz, CDCl$_3$) δ 8.09 (dd, *J* = 2.9, 0.7 Hz, 1H), 7.99 (dd, *J* = 4.7, 1.4 Hz, 1H), 7.36 (dd, *J* = 1.9, 0.8 Hz, 1H), 7.09 (ddd, *J* = 8.3, 4.7, 0.7 Hz, 1H), 6.94 (ddd, *J* = 8.3, 2.9, 1.4 Hz, 1H), 6.32 (dd, *J* = 3.2, 1.8 Hz, 1H), 6.24 (dq, *J* = 3.3, 0.8 Hz, 1H), 4.33 (d, *J* = 5.7 Hz, 2H), 4.17 (brs, 1H).

^{13}C NMR (101 MHz, CDCl$_3$) δ 152.0, 143.7, 142.4, 139.6, 136.5, 123.8, 119.1, 110.5, 107.6, 41.1.

HRMS (EI) m/z: [M] calc'd for $C_{10}H_{10}N_2O$: 174.0788, found: 174.0791

FTIR (ATR): $\tilde{\nu}$ [cm^{-1}] = 3252, 3101, 3035, 2924, 2852, 1581, 1504, 1482, 1419, 1303, 1241, 1186, 1145, 1072, 1010, 918, 884, 793, 732, 704, 628, 598, 560, 514, 413.

***N*-(Thiophen-2-ylmethyl)pyridin-3-amine (37):**

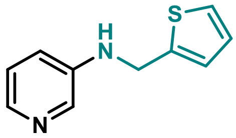

N-(Thiophen-2-ylmethyl)pyridin-3-amine was prepared according to **GP-2**, using Zn dust (2.0 mg, 10 mol%), Ni($^{4\text{-}t\text{Bu}}$stb)$_3$ (14.1 mg, 5 mol%), ^{t}Bu-TMG (0.11 mL, 0.55 mmol, 1.8 equiv.), 3-bromopyridine (29 µL, 0.3 mmol, 1.0 equiv.), and thiophen-2-ylmethanamine (62 µL, 0.6 mmol, 2.0 equiv.) in DMA (0.3 mL) at 100 °C. Purification *via* column chromatography (silica gel, 80:20 hexanes/EtOAc to EtOAc) afforded the title compound as a yellow solid (44.0 mg, 77%).

Spectroscopic data matches those reported in the literature.[17]

m.p.: 77.5–78.5 °C

***R*$_f$:** 0.59 (EtOAc)

^{1}H NMR (400 MHz, CDCl$_3$) δ 8.09 (dd, *J* = 2.9, 0.7 Hz, 1H), 7.99 (dd, *J* = 4.7, 1.4 Hz, 1H), 7.23 (dd, *J* = 5.1, 1.2 Hz, 1H), 7.09 (ddd, *J* = 8.3, 4.7, 0.7 Hz, 1H), 7.02 (dq, *J* = 3.3, 1.1 Hz, 1H), 6.97 (dd, *J* = 5.1, 3.5 Hz, 1H), 6.93 (ddd, *J* = 8.3, 2.9, 1.4 Hz, 1H), 4.52 (dd, *J* = 5.7, 1.0 Hz, 2H), 4.20 (brs, 1H).

^{13}C NMR (101 MHz, CDCl$_3$) δ 143.6, 142.1, 139.6, 136.5, 127.1, 125.5, 125.0, 123.9, 119.1, 43.2.

***N*-Phenylpyridin-3-amine (38):**

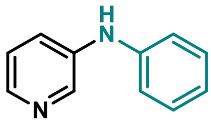

N-Phenylpyridin-3-amine was prepared according to **GP-1**, using Zn dust (4.0 mg, 20 mol%), Ni($^{4\text{-}t\text{Bu}}$stb)$_3$ (28.2 mg, 10 mol%), DABCO (60.8 mg, 0.54 mmol, 1.8 equiv.), 3-bromopyridine (29 µL, 0.3 mmol, 1.0 equiv.), and aniline (55 µL, 0.6 mmol, 2.0 equiv.) in DMA (0.3 mL) at 60 °C. Purification *via* column chromatography (silica gel, 50:50 hexanes/EtOAc) afforded the title compound as a beige solid (46.3 mg, 90%).

Spectroscopic data matches those reported in the literature.[18]

m.p.: 140.2–141.2 °C

***R*$_f$:** 0.27 (50:50 hexanes/EtOAc)

^{1}H NMR (300 MHz, CDCl$_3$) δ 8.38 (dd, *J* = 2.8, 0.7 Hz, 1H), 8.17 (dd, *J* = 4.7, 1.4 Hz, 1H), 7.41 (ddd, *J* = 8.3, 2.9, 1.4 Hz, 1H), 7.35 – 7.26 (m, 2H), 7.17 (ddd, *J* = 8.3, 4.7, 0.7 Hz, 1H), 7.13 – 7.05 (m, 2H), 7.05 – 6.95 (m, 1H), 5.73 (brs, 1H).

^{13}C NMR (101 MHz, CDCl$_3$) δ 142.09, 142.08, 140.3, 140.0, 129.7, 123.8, 123.6, 122.2, 118.5.

N-(4-Methoxyphenyl)pyridin-3-amine (39):

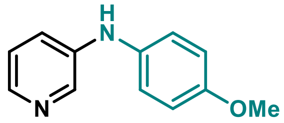

N-(4-Methoxyphenyl)pyridin-3-amine was prepared according to **GP-1**, using Zn dust (4.0 mg, 20 mol%), Ni($^{4\text{-}t\text{Bu}}$stb)$_3$ (28.2 mg, 10 mol%), DABCO (60.9 mg, 0.54 mmol, 1.8 equiv.), 3-bromopyridine (29 µL, 0.3 mmol, 1.0 equiv.), and *p*-anisidine (74.1 mg, 0.6 mmol, 2.0 equiv.) in DMA (0.3 mL) at 60 °C. Purification *via* column chromatography (silica gel, 50:50 hexanes/EtOAc) afforded the title compound as a red solid (30.0 mg, 50%).

Spectroscopic data matches those reported in the literature.[18]

m.p.: 136.3–137.3 °C

R**$_f$:** 0.23 (50:50 hexanes/EtOAc)

^{1}H NMR (400 MHz, CDCl$_3$) δ 8.25 (dd, *J* = 2.8, 0.8 Hz, 1H), 8.07 (dd, *J* = 4.6, 1.4 Hz, 1H), 7.20 (ddt, *J* = 8.3, 2.9, 1.6 Hz, 1H), 7.14 – 7.04 (m, 3H), 6.93 – 6.84 (m, 2H), 5.52 (brs, 1H), 3.81 (s, 3H).

^{13}C NMR (101 MHz, CDCl$_3$) δ 156.1, 141.8, 140.9, 138.6, 134.5, 123.8, 122.9, 121.3, 115.0, 55.7.

N-(3-(4,4,5,5-Tetramethyl-1,3,2-dioxaborolan-2-yl)phenyl)pyridin-3-amine (40):

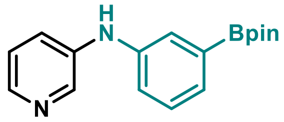

N-(3-(4,4,5,5-Tetramethyl-1,3,2-dioxaborolan-2-yl)phenyl)pyridin-3-amine was prepared according to **GP-2**, using Zn dust (2.0 mg, 10 mol%), Ni($^{4\text{-}t\text{Bu}}$stb)$_3$ (14.1 mg, 5 mol%), ^{t}Bu-TMG (0.11 mL, 0.55 mmol, 1.8 equiv.), 3-bromopyridine (29 µL, 0.3 mmol, 1.0 equiv.), and 3-(4,4,5,5-tetramethyl-1,3,2-dioxaborolan-2-yl)aniline (131.9 mg, 0.6 mmol, 2.0 equiv.) in DMA (0.3 mL) at 100 °C. Purification *via* column chromatography (silica gel, 50:50 hexanes/EtOAc), followed by precipitation in cold pentane (−18 °C) afforded the title compound as a beige solid (47.2 mg, 53%).

Note: The product suffers from trace decomposition during column chromatography in form of proto-deboronation. The isolated material contains besides the title compound (47.2 mg, 53%), additional amounts of *N*-phenylpyridin-3-amine (ca. 2%).

m.p.: 125.9–126.9 °C

R**$_f$:** 0.36 (50:50 hexanes/EtOAc)

^{1}H NMR (400 MHz, CDCl$_3$) δ 8.39 – 8.34 (m, 1H), 8.14 (dd, *J* = 4.7, 1.4 Hz, 1H), 7.51 (dd, *J* = 2.6, 1.0 Hz, 1H), 7.44 (dt, *J* = 7.3, 1.1 Hz, 1H), 7.37 (ddd, *J* = 8.3, 2.8, 1.4 Hz, 1H), 7.30 (t, *J* = 7.6 Hz, 1H), 7.20 (ddd, *J* = 8.0, 2.5, 1.2 Hz, 1H), 7.15 (ddd, *J* = 8.3, 4.7, 0.6 Hz, 1H), 5.82 (s, 1H), 1.33 (s, 12H).

^{13}C NMR (101 MHz, CDCl$_3$) δ 141.78, 141.76, 141.4, 140.2, 140.0, 129.0, 128.6, 125.0, 123.9, 123.2, 121.4, 84.0, 25.0.

^{11}B NMR (128 MHz, CDCl$_3$) δ 31.16

HRMS (EI) m/z: [M] calc'd for $C_{17}H_{21}N_2O_2B$: 296.1691, found: 296.1697

FTIR (ATR): $\tilde{\nu}$ [cm^{-1}] = 3220, 3098, 3041, 2992, 2977, 2962, 2924, 1598, 1572, 1485, 1433, 1362, 1318, 1238, 1148, 1072, 1054, 1024, 1006, 968, 918, 896, 809, 776, 738, 696, 637, 586, 541, 505, 446.

4-Methyl-*N*-(pyridin-3-yl)benzenesulfonamide (41):

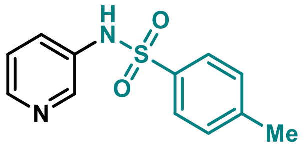

4-Methyl-*N*-(pyridin-3-yl)benzenesulfonamide was prepared according to **GP-2**, using Zn dust (2.0 mg, 10 mol%), Ni($^{4\text{-}t\text{Bu}}$stb)$_3$ (14.1 mg, 5 mol%), ^{t}Bu-TMG (0.11 mL, 0.55 mmol, 1.8 equiv.), 3-bromopyridine (29 µL, 0.3 mmol, 1.0 equiv.), and *p*-toluenesulfonamide (103.1 mg, 0.6 mmol, 2.0 equiv.) in DMA (0.3 mL) at 100 °C. Purification *via* preparative HPCL (150 mm YMC Pack Pro C18, 30 mm i.D., 5 µm,45:55 MeOH/H_2O to 95:5 MeOH/H_2O) afforded the title compound as a white solid (14.8 mg, 20%).

Spectroscopic data matches those reported in the literature.[19]

^{1}H NMR (400 MHz, DMSO-*d$_8$*) δ 10.48 (s, 1H), 8.27 (d, *J* = 2.7 Hz, 1H), 8.24 (dd, *J* = 4.7, 1.5 Hz, 1H), 7.67 – 7.62 (m, 2H), 7.49 (ddd, *J* = 8.3, 2.7, 1.5 Hz, 1H), 7.37 – 7.33 (m, 2H), 7.27 (ddd, *J* = 8.3, 4.7, 0.8 Hz, 1H), 2.33 (s, 3H).

^{13}C NMR (101 MHz, DMSO-*d$_8$*) δ 145.2, 143.6, 141.7, 136.2, 134.5, 129.8, 127.2, 126.7, 124.0, 21.0.

1,1-Diphenyl-*N*-(pyridin-3-yl)methanimine (42):

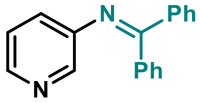

1,1-Diphenyl-*N*-(pyridin-3-yl)methanimine was prepared according to **GP-2**, using Zn dust (2.0 mg, 10 mol%), Ni($^{4\text{-}t\text{Bu}}$stb)$_3$ (14.1 mg, 5 mol%), ^{t}Bu-TMG (0.11 mL, 0.55 mmol, 1.8 equiv.), 3-bromopyridine (29 µL, 0.3 mmol, 1.0 equiv.), and benzophenone imine (100 µL, 0.6 mmol, 2.0 equiv.) in DMA (0.3 mL) at 100 °C. Purification *via* column chromatography (silica gel, 6:1 hexanes/EtOAc) afforded the title compound as a yellow oil (64.6 mg, 83%).

Spectroscopic data matches those reported in the literature.[20-21]

***R$_f$*:** 0.15 (6:1 hexanes/EtOAc)

^{1}H NMR (400 MHz, $CDCl_3$) δ 8.17 (dq, *J* = 3.7, 1.8 Hz, 1H), 8.04 (td, *J* = 2.4, 1.2 Hz, 1H), 7.77 (ddt, *J* = 8.8, 3.8, 1.6 Hz, 2H), 7.54 – 7.47 (m, 1H), 7.46 – 7.38 (m, 2H), 7.32 – 7.25 (m, 3H), 7.13 – 7.02 (m, 4H).

^{13}C NMR (101 MHz, $CDCl_3$) δ 170.5, 147.4, 144.5, 142.6, 139.1, 135.6, 131.4, 129.6, 129.5, 129.1, 128.4, 128.3, 128.2, 123.2.

5 mmol Scale Synthesis of *tert*-butyl 5-(pyridin-3-yl)hexahydropyrrolo[3,4-*c*]pyrrole-2(1*H*)-carboxylate (31):

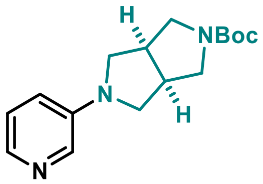

A dried 25 mL Schlenk flask equipped with a stirring bar and held under argon was charged with Zn dust (66.1 mg, 19 mol%), Ni($^{4\text{-}t\text{Bu}}$stb)$_3$ (470.8 mg, 10 mol%), DABCO (1.01 g, 9.0 mmol, 1.7 equiv.), and *tert*-butyl hexahydropyrrolo[3,4-*c*]pyrrole-2(1*H*)-carboxylate (2.13 g, 10.0 mmol, 1.9 equiv.), followed by one cycle of vacuum/argon. Then 3-bromopyridine (0.5 mL, 5.2 mmol, 1.0 equiv.) and DMA (5.0 mL) were added, and the reaction vessel was placed into a pre-heated oil-bath set to 60 °C. The reaction was stirred at this temperature for 24 h. The reaction was allowed to cool down to room temperature, and was then diluted with EtOAc (15 mL), quenched by the addition of an aqueous solution of HCl (1 M, 17 ml), followed by neutralization with a saturated aqueous solution of $NaHCO_3$ (22 mL). A saturated aqueous solution of LiCl (50 mL) was added, and the phases were separated. The organic phase was washed with a saturated aqueous solution of LiCl (3 x 50 mL), followed by back extraction of the combined aqueous layers with EtOAc (1 x 50 mL). The combined organic layers were dried over $MgSO_4$. Purification *via* column chromatography (silica gel, EtOAc) afforded the title compound as a beige solid (0.83 g, 55%).

Limitations of the Scope

The following C–N bond couplings were not successful under our reaction conditions:

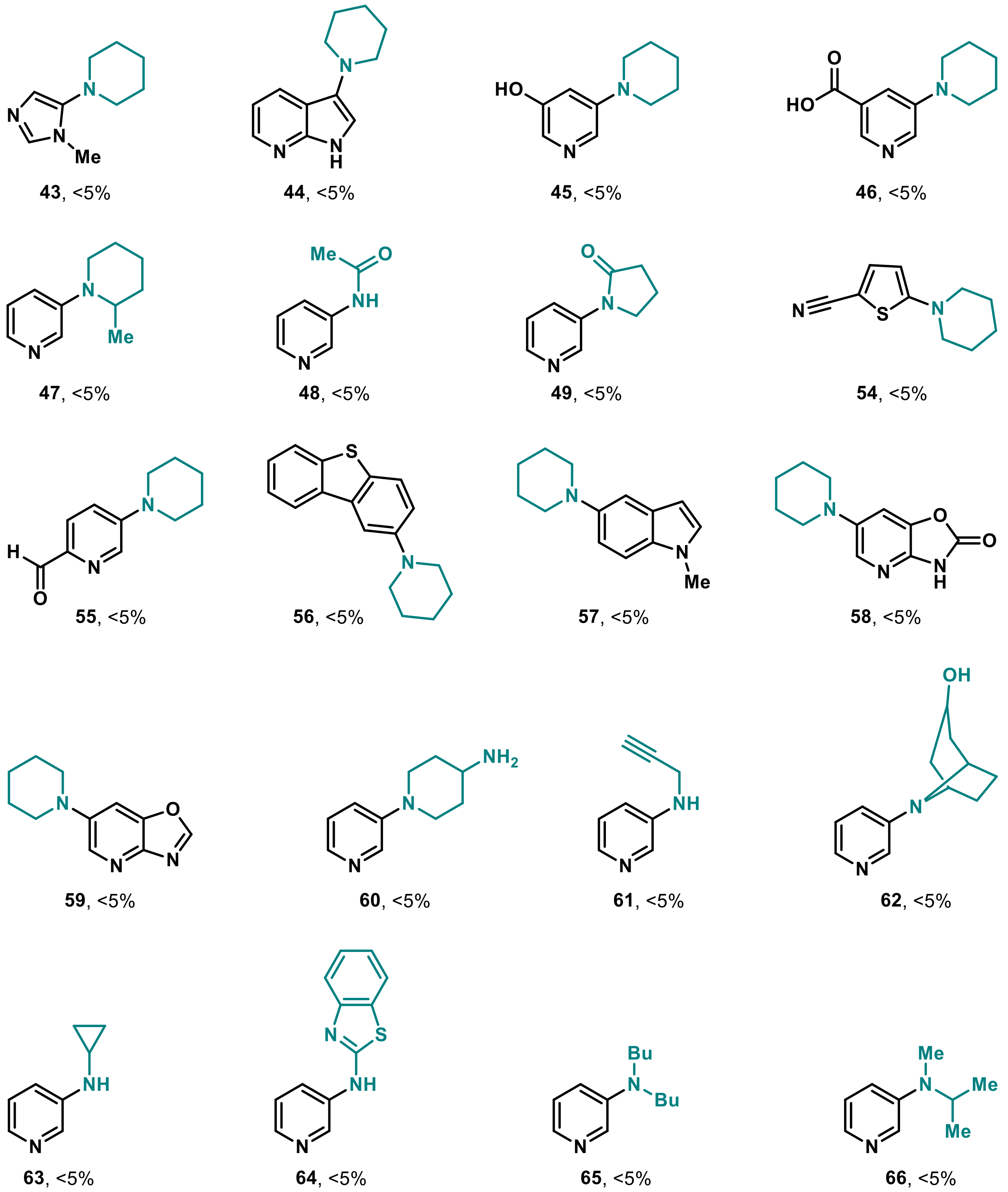

Figure S1: Unsuccessful substrates.

Coordination of Piperidine to Ni($^{4\text{-}tBu}$stb)$_3$

X-Ray:

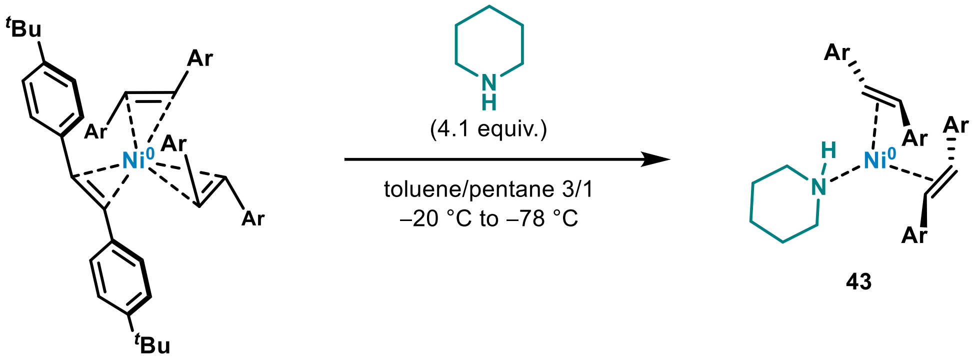

Ar = 4-^{t}Bu-C_4H_6

A 10 mL Schlenk flask was charged with Ni($^{4\text{-}tBu}$stb)$_3$ (60 mg, 0.064 mmol, 1.0 equiv.) and piperidine (26 µL, 0.263 mmol, 4.1 equiv.) and toluene (1 mL) at −20 °C. Pentane (0.3 mL) was added and the reaction temperature was slowly decreased to −65 °C over 72 h with the aid of a cryostat. The flask was then taken into a Dewar flask filled with dry ice (−78 °C) and stored for 1 week, during which orange single crystals of **50** suitable for XRD analysis started to form.

NMR:

^{1}H NMR (−40 °C, 600.20 MHz, THF-*d₈*) δ 7.34 – 7.25 (m, 8H), 6.80 (d, *J* = 7.5 Hz, 4H), 6.17 (brs, 4H), 4.21 (d, *J* = 11.1 Hz, 2H), 3.68 (d, *J* = 11.1 Hz, 2H), 2.62 (q, *J* = 12.1 Hz, 1H), 2.51 – 2.40 (m, 2H), 2.28 – 2.17 (m, 1H), 1.79 (d, *J* = 12.0 Hz, 1H), 1.61 (brs, 1H), 1.40 (s, 18H), 1.38 – 1.31 (m, 4H), 1.16 (s, 18H), 1.07 (d, *J* = 13.2 Hz, 1H).

^{13}C NMR (−40 °C, 150.94 MHz, THF-d_8) δ 146.4, 145.1, 141.7, 141.5, 126.2, 125.4 (2C), 124.5, 75.1, 66.0, 52.3, 52.0, 35.2, 34.8, 31.9, 31.6, 29.0, 28.7, 25.1

^{15}N NMR (−40 °C, 600.20 MHz, THF-d_8) δ −359.8.

Note 1: Besides **50**, 1.5 equiv. of free $^{4\text{-}tBu}$stb was detected in solution:

^{1}H NMR (−40 °C, 600.20 MHz, THF-d_8) δ 7.49 (d, *J* = 8.3 Hz, 6H), 7.38 (d, *J* = 8.2 Hz, 6H), 7.17 (s, 3H), 1.31 (s, 27H).

^{13}C NMR (−40 °C, 150.94 MHz, THF-d_8) δ 150.8, 135.7, 128.0, 126.9, 126.3, 35.2, 31.6.

Note 2: The VT NMR experiment was measured on an AVIII 500 MHz spectrometer. The VT NMR experiment was performed with 5.0 equiv. of piperidine, **50** was the major species detected. For characterization of **50** a Bruker AVNeo 600 MHz NMR spectrometer was used. The ideal temperature of −40 °C was chosen based on VT NMR results and only 1.0 equiv. of piperidine was used in order to ensure clear signals.

The following major Ni-species could be assigned in the mixture

User Report
SAX-SA-826-01

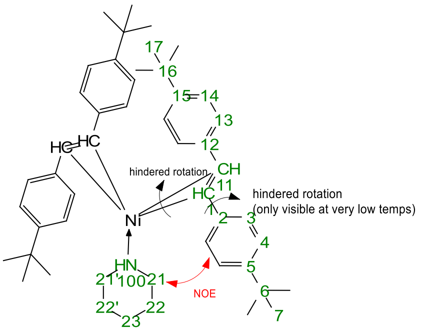

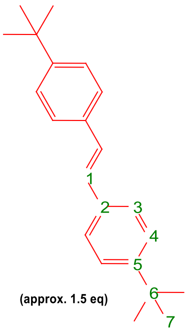

(approx. 1.5 eq)

Remarks

The sample contained approx. 1.5 eq. of free stilbene.
Based on the earlier request (page 4; SA-824), the best temperature for a characterisation was determined to be at 233K. Lower temperatures lead to an additional broadening due to hindered rotations around the C1-C2 (approx ~8.4 kcal/mol), C11-12 axis. At more elevated temperatures this was mostly averaged, whereas a "formal" rotation C=C->Ni axis (barrier based on VT NMR data ~13kcal/mol) was only visible as exchange cross peaks in a 2D ROESY, but not very significant observed in the spectrum.

The shielding of of C1(65.9 ppm) and C11(75.1 ppm) are characterisitic for the binding to the metal.

Based on the observed couplings, the piperidine ring seems to be mostly present in an chair conformation. By looking at the VT NMR data this seems to be the case in the whole VT range (where also no significant exchange with free piperdine via line broadening is visible).

An overview of all chemical shift assignments and 2D NMR correlations is given on the next page of this report.

P-ID: ML00xxx
Measured on: 03/11/2023
CHIFFRE:SAX-SA-826-01
ELNA#:11274
Client: Rakan Saeb
Group: Cornella
Spectroscopist: Leutzsch
Analysed on: 05/11/2023
Analysed by: Leutzsch
Amount: 20.0 mg
Solvent: THF
Reference: 1H+13C on solvent,other nuclei w/ xiref
Temperature: 233 K
Spectrometer: av600neo
Probe: cryoBBO
Experiments: 1H-zg30, 1H-zg30, 13C-zgpg30, 1H-13C-hsqcedetgpsisp2.3, 1H-1H-cosygpppqf, 1H-13C-hmbcetgpl3nd, 1H-1H-roesyadjsph, 1H-15N-hmbcgpndqf, 13C-deptsp135, 1H-seldigpzs

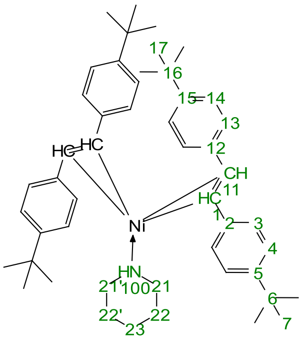

Atom	J	δ (ppm)	HSQC	COSY	HMBC	ROESY
1 C		65.949	1			
H	11.1(11)	4.212	1	11	3, 12	13, 21'ax, 21ax, 100
2 C		141.718			4, 11	
3 C		125.436	3		1	
H	7.5(4)	6.169	3			
4 C		125.436	4		4	
H	7.5(3)	6.799	4		2, 4	
5 C		145.130			7	
6 C		34.824			7	
7 C		31.612	7		7	
H3		1.164	7		5, 6, 7	
11 C		75.131	11		13	
H	11.1(1)	3.677	11	1	2, 13	13
12 C		141.535			1, 14	
13 C		124.503	13		11, 13	
H		7.308	13		11, 13, 15	1, 11
14 C		126.173	14		14	
H		7.286	14		12, 14, 16	
15 C		146.433			13, 17	

Shifts in regions with significant overlaps were extracted from the 2D HSQC and/or 1D selective TOCSY experiments.

Atom	J	δ (ppm)	HSQC	COSY	HMBC	ROESY
16 C		35.167			14, 17	
17 C		31.871	17		17	
H3		1.397	17		15, 16, 17	
21 C		51.974	21ax, 21eq			
Hax	12.0(22ax), 12.0(21eq), 12.0(100)	2.623	21	21eq, 22ax, 22eq, 100		1, 21eq, 22eq
Heq	12.0(21ax)	1.794	21	21'eq, 21ax, 22ax, 22eq		21ax, 22ax, 100
21' C		52.309	21'ax, 21'eq			
Hax	12.0(100)	2.222	21'	21'eq, 22', 100		1, 21'eq, 22'
Heq		2.454	21'	21'ax, 21eq, 22'		21'ax
22 C		29.034	22ax, 22eq			
Hax	12.0(21ax)	1.071	22	21ax, 21eq		21eq, 23eq, 100
Heq		1.355	22	21ax, 21eq		21ax
22' C		28.682	22'			
H2		1.360	22'	21'ax, 21'eq		21'ax, 100
23 C		25.111	23ax, 23eq			
Hax		1.349	23			23eq
Heq		1.613	23			22ax, 23ax
100 N		-359.800			100	
H	12.0(21'ax), 12.0(21ax)	2.455		21'ax, 21ax	100	1, 21eq, 22', 22ax

Figure S2: NMR analysis report, explaining some details and listing observed signals.

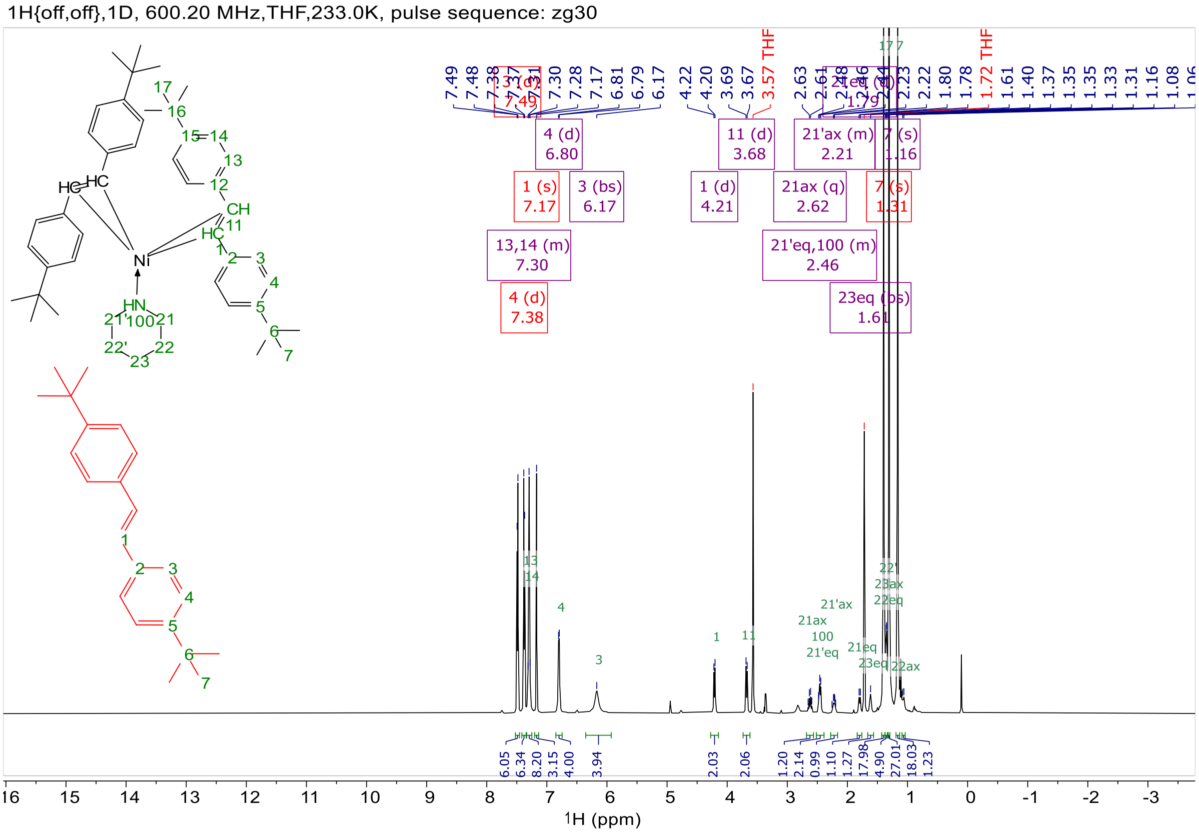

Variable temperature ^{1}H NMR of 50 (from 173 K to 298 K).

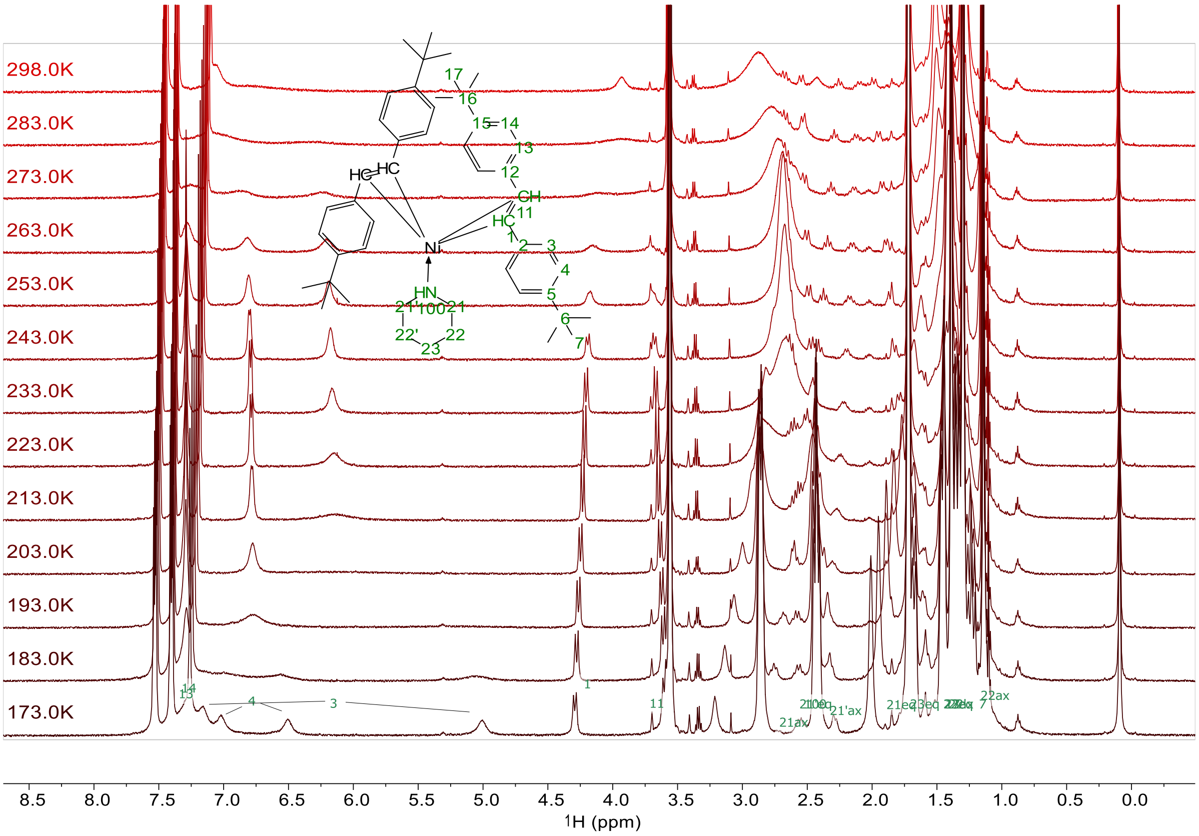

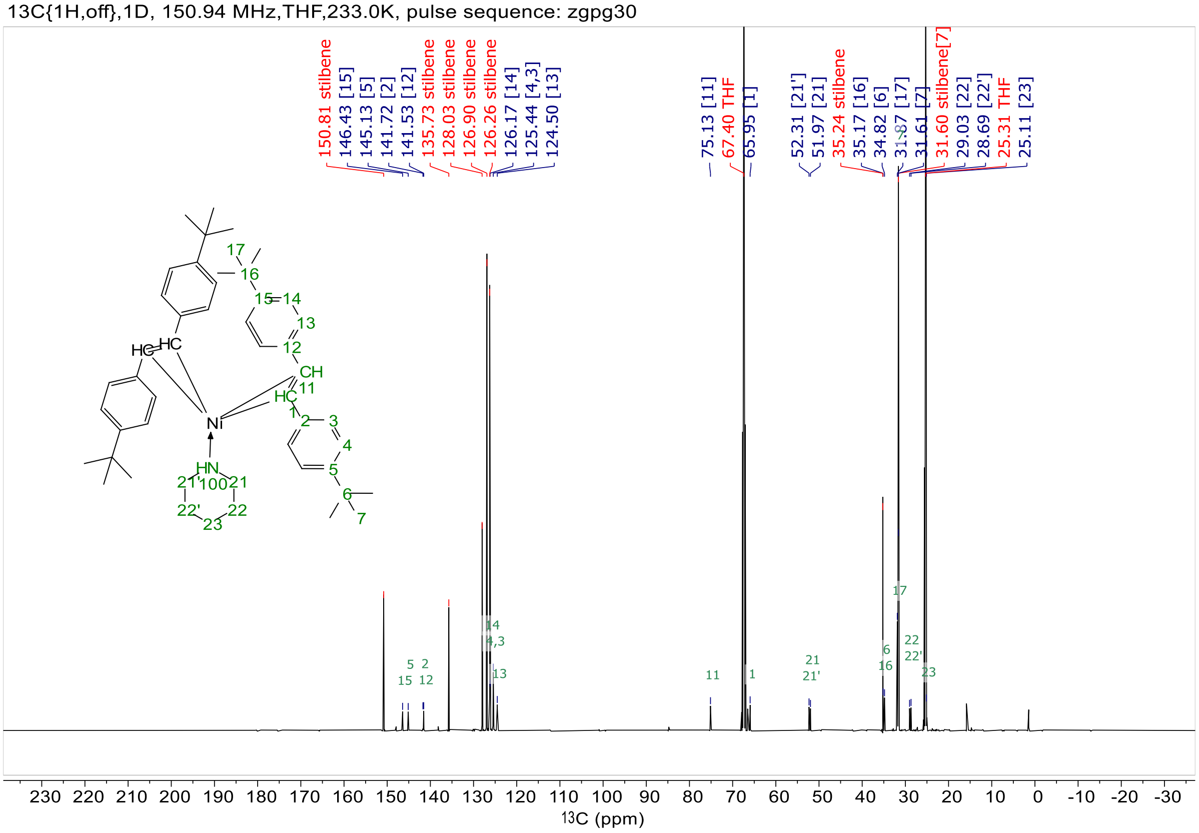
13C{1H,off},1D, 150.94 MHz,THF,233.0K, pulse sequence: zgpg30
150.81 stilbene
146.43 [15]
145.13 [5]
141.72 [2]
141.53 [12]
135.73 stilbene
128.03 stilbene
126.90 stilbene
126.26 stilbene
126.17 [14]
125.44 [4,3]
124.50 [13]
75.13 [11]
67.40 THF
65.95 [1]
52.31 [21']
51.97 [21]
35.24 stilbene
35.17 [16]
34.82 [6]
31.87 [17]
31.61 [7]
31.60 stilbene[7]
29.03 [22]
28.69 [22']
25.31 THF
25.11 [23]
13C (ppm)

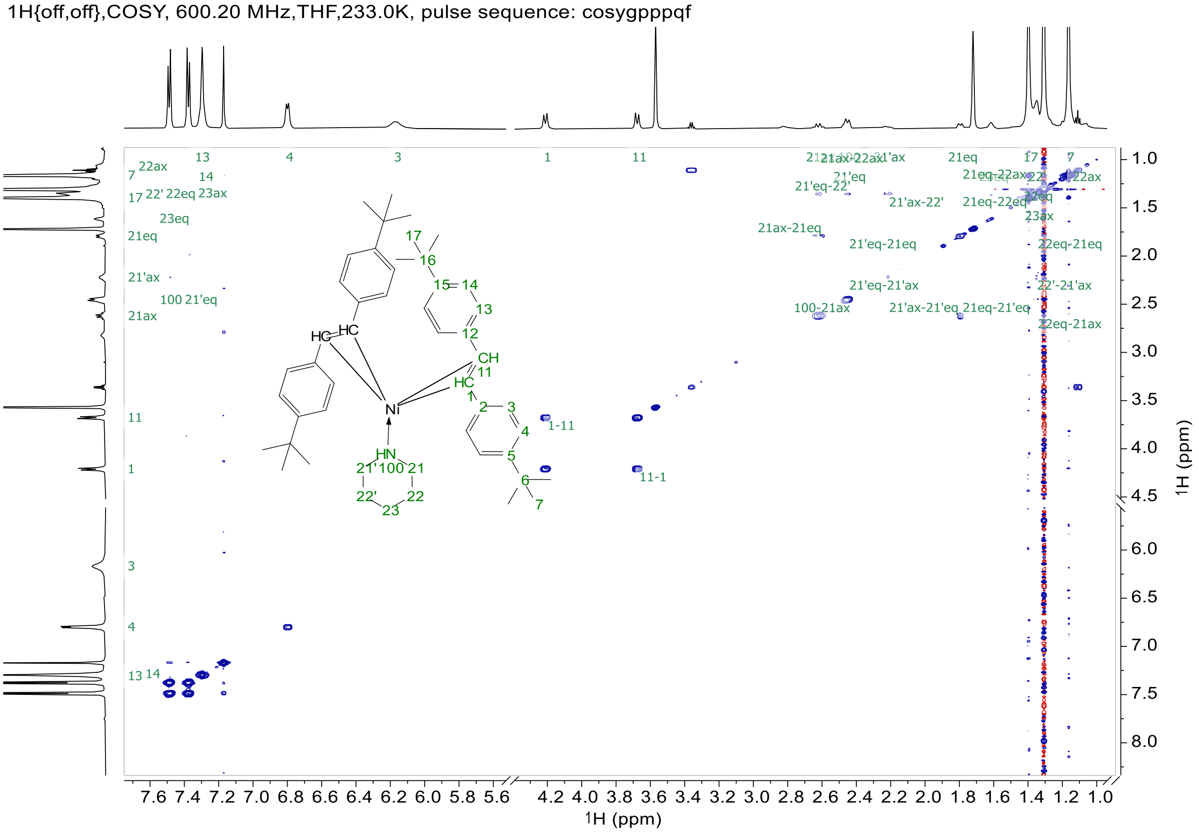
1H{off,off},COSY, 600.20 MHz,THF,233.0K, pulse sequence: cosygpppqf
1-11
11-1
21ax-21eq
21'eq-21eq
21'eq-21'ax
100-21ax
21'ax-21'eq
21eq-21'eq
22eq-21eq
22'-21'ax
22eq-21ax
21'ax-22'
21eq-22eq
1H (ppm)

1H{13C,off},HMBC, 600.20 MHz,THF,233.0K, pulse sequence: hmbcetgpl3nd

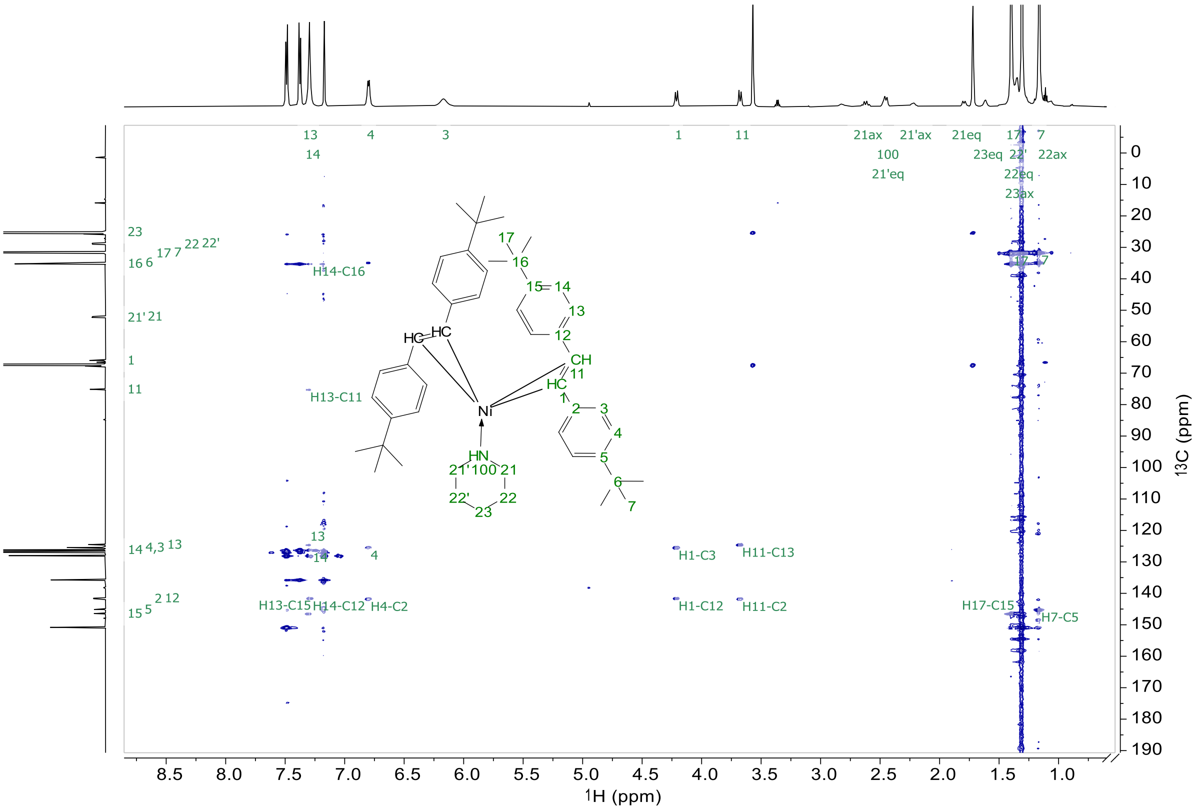

1H{off,off},ROESY, 600.20 MHz,THF,233.0K, pulse sequence: roesyadjsph

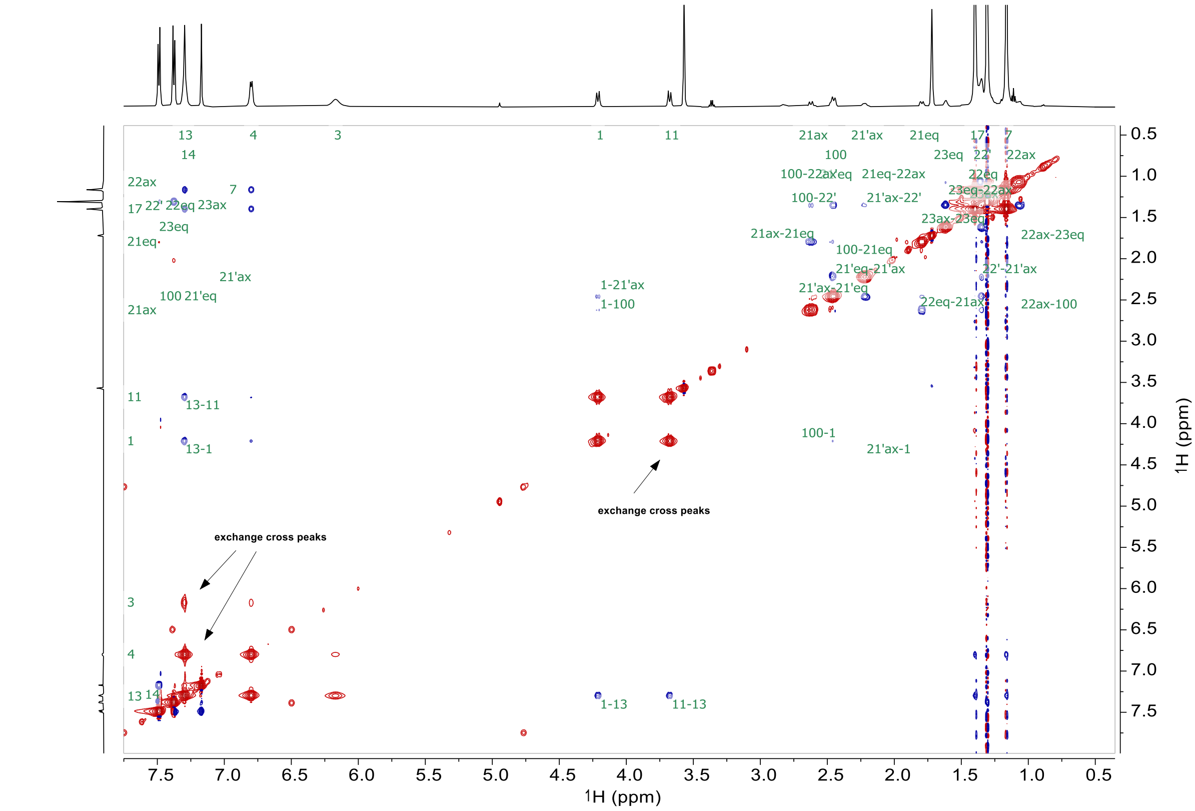

1H{15N,off},HMBC, 600.20 MHz,THF,233.0K, pulse sequence: hmbcgpndqf

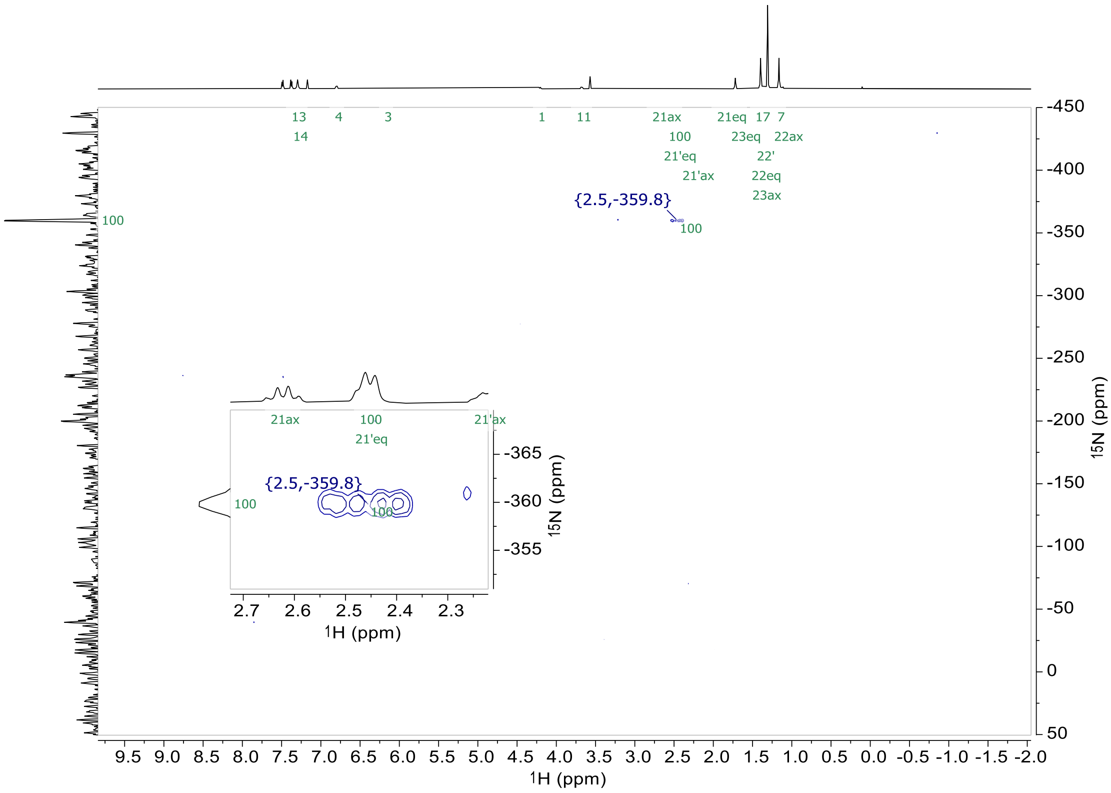

13C{1H,off},DEPT-135, 150.94 MHz,THF,233.0K, pulse sequence: deptsp135

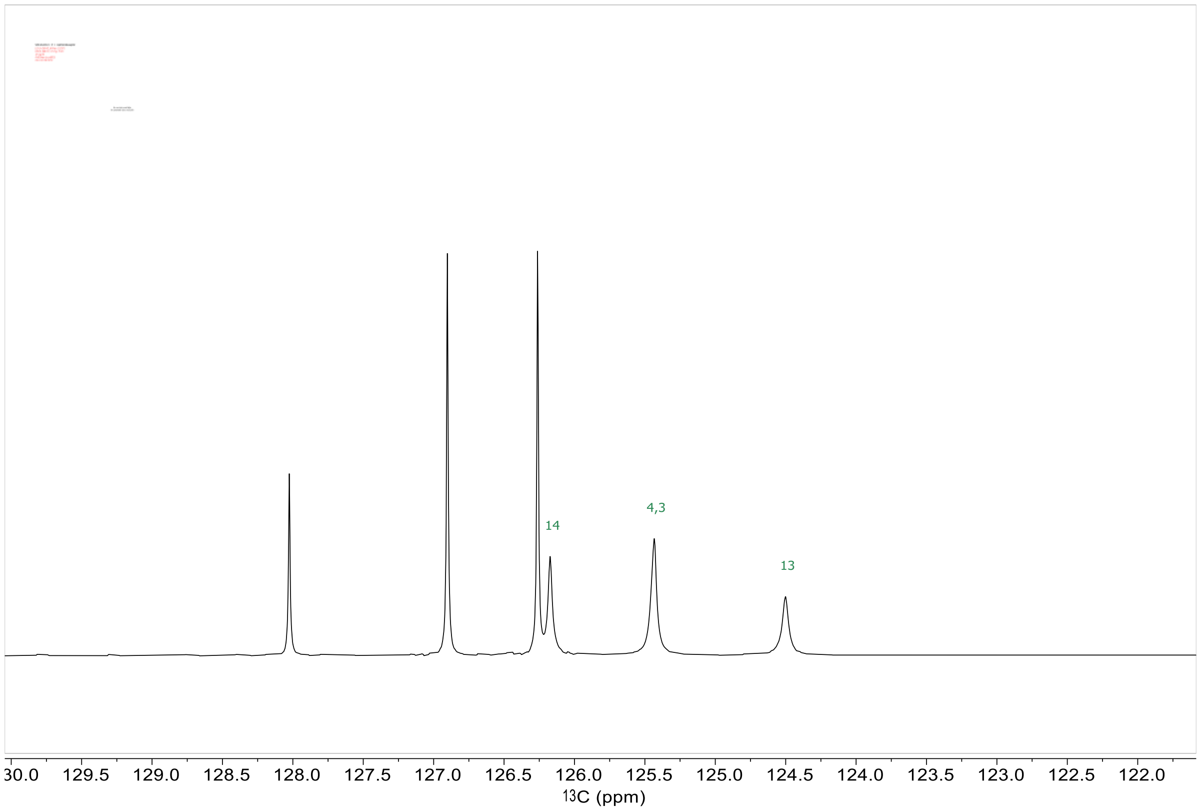

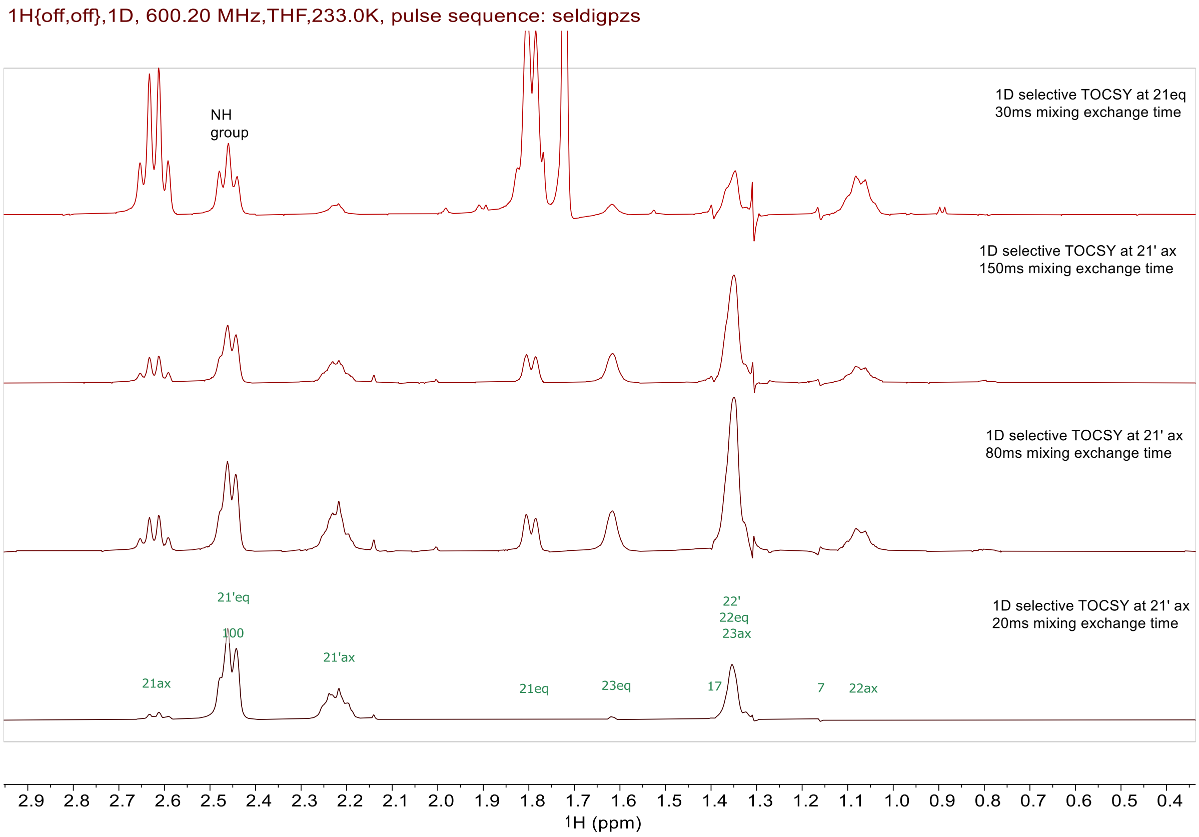

1H{off,off},1D, 600.20 MHz,THF,233.0K, pulse sequence: seldigpzs
NH
group
1D selective TOCSY at 21eq
30ms mixing exchange time
1D selective TOCSY at 21' ax
150ms mixing exchange time
1D selective TOCSY at 21' ax
80ms mixing exchange time
1D selective TOCSY at 21' ax
20ms mixing exchange time
21'eq
100
21ax
21'ax
21eq
23eq
17
22'
22eq
23ax
7
22ax
2.9 2.8 2.7 2.6 2.5 2.4 2.3 2.2 2.1 2.0 1.9 1.8 1.7 1.6 1.5 1.4 1.3 1.2 1.1 1.0 0.9 0.8 0.7 0.6 0.5 0.4
1H (ppm)

References

1. Nattmann, L.; Cornella, J., Ni($^{4\text{-tBu}}$stb)$_3$: A Robust 16-Electron Ni(0) Olefin Complex for Catalysis. *Organometallics* **2020,** *39*, 3295–3300.
2. Ahn, Y. M.; Yang, K.; Georg, G. I., A Convenient Method for the Efficient Removal of Ruthenium Byproducts Generated during Olefin Metathesis Reactions. *Org. Lett.* **2001,** *3*, 1411–1413.
3. Knight, B. J.; Tolchin, Z. A.; Smith, J. M., A predictive model for additions to *N*-alkyl pyridiniums. *Chem. Commun.* **2021,** *57*, 2693–2696.
4. Nimje, R. Y.; Vytla, D.; Kuppusamy, P.; Velayuthaperumal, R.; Jarugu, L. B.; Reddy, C. A.; Chikkananjaiah, N. K.; Rampulla, R. A.; Cavallaro, C. L.; Li, J.; Mathur, A.; Gupta, A.; Roy, A., Synthesis of Differentially Protected Azatryptophan Analogs via $Pd_2(dba)_3$/XPhos Catalyzed Negishi Coupling of N-Ts Azaindole Halides with Zinc Derivative from Fmoc-Protected *tert*-Butyl (*R*)-2-Amino-3-iodopropanoate. *J. Org. Chem.* **2020,** *85*, 11519–11530.
5. Alvarez, M.; Fernández, D.; Joule, J. A., Synthesis of 3-Aryl- and 3-Heteroaryl-7-azaindoles. *Synthesis* **1999,** *1999*, 615–620.
6. Manolikakes, G.; Gavryushin, A.; Knochel, P., An Efficient Silane-Promoted Nickel-Catalyzed Amination of Aryl and Heteroaryl Chlorides. *J. Org. Chem.* **2008,** *73*, 1429–1434.
7. Trowse, B. R.; Byrne, F. P.; Sherwood, J.; O'Brien, P.; Murray, J.; Farmer, T. J., 2,2,5,5-Tetramethyloxolane (TMO) as a Solvent for Buchwald–Hartwig Aminations. *ACS Sustainable Chem. Eng.* **2021,** *9*, 17330–17337.
8. Nguyen, M. H.; Smith, A. B., III, Copper-Catalyzed Electrophilic Amination of Organolithiums Mediated by Recoverable Siloxane Transfer Agents. *Org. Lett.* **2013,** *15*, 4872–4875.
9. Sun, R.; Qin, Y.; Nocera, D. G., General Paradigm in Photoredox Nickel-Catalyzed Cross-Coupling Allows for Light-Free Access to Reactivity. *Angew. Chem. Int. Ed.* **2020,** *59*, 9527–9533.
10. Hatakeyama, T.; Yoshimoto, Y.; Ghorai, S. K.; Nakamura, M., Transition-Metal-Free Electrophilic Amination between Aryl Grignard Reagents and N-Chloroamines. *Org. Lett.* **2010,** *12*, 1516–1519.
11. Quach, T. D.; Batey, R. A., Ligand- and Base-Free Copper(II)-Catalyzed C−N Bond Formation: Cross-Coupling Reactions of Organoboron Compounds with Aliphatic Amines and Anilines. *Org. Lett.* **2003,** *5*, 4397–4400.
12. Topchiy, M. A.; Dzhevakov, P. B.; Rubina, M. S.; Morozov, O. S.; Asachenko, A. F.; Nechaev, M. S., Solvent-Free Buchwald–Hartwig (Hetero)arylation of Anilines, Diarylamines, and Dialkylamines Mediated by Expanded-Ring N-Heterocyclic Carbene Palladium Complexes. *Eur. J. Org. Chem.* **2016,** *2016*, 1908–1914.
13. Zhou, J.; Wang, S.; Lu, Y.; Li, L.; Duan, W.; Wang, Q.; Wang, H.; Wei, W., Solvent-driven C(sp^3)–H thiocarbonylation of benzylamine derivatives under catalyst-free conditions. *Green Chem.* **2021,** *23*, 767–773.
14. Li, J.; Huang, C.; Wen, D.; Zheng, Q.; Tu, B.; Tu, T., Nickel-Catalyzed Amination of Aryl Chlorides with Amides. *Org. Lett.* **2021,** *23*, 687–691.
15. Wagaw, S.; Buchwald, S. L., The Synthesis of Aminopyridines: A Method Employing Palladium-Catalyzed Carbon−Nitrogen Bond Formation. *J. Org. Chem.* **1996,** *61*, 7240–7241.
16. Corcoran, E. B.; Pirnot, M. T.; Lin, S.; Dreher, S. D.; DiRocco, D. A.; Davies, I. W.; Buchwald, S. L.; MacMillan, D. W. C., Aryl amination using ligand-free Ni(II) salts and photoredox catalysis. *Science* **2016,** *353*, 279–283.
17. Ouyang, J.-S.; Zhang, X.; Pan, B.; Zou, H.; Chan, A. S. C.; Qiu, L., Solvent-Free Buchwald–Hartwig Amination of Heteroaryl Chlorides by N-Heterocyclic Carbene–Palladium Complex (SIPr)Ph_2Pd(cin)Cl at Room Temperature. *Org. Lett.* **2023,** *25*, 7491–7496.
18. Zhu, L.; Ye, Y.-M.; Shao, L.-X., Well-defined NHC–Pd(II)–Im (NHC=N-heterocyclic carbene; Im=1-methylimidazole) complex catalyzed C–N coupling of primary amines with aryl chlorides. *Tetrahedron* **2012,** *68*, 2414–2420.
19. Zhang, W.; Xie, J.; Rao, B.; Luo, M., Iron-Catalyzed N-Arylsulfonamide Formation through Directly Using Nitroarenes as Nitrogen Sources. *J. Org. Chem.* **2015,** *80*, 3504–3511.

20. Tundel, R. E.; Anderson, K. W.; Buchwald, S. L., Expedited Palladium-Catalyzed Amination of Aryl Nonaflates through the Use of Microwave-Irradiation and Soluble Organic Amine Bases. *J. Org. Chem.* **2006,** *71*, 430–433.
21. Shen, Q.; Shekhar, S.; Stambuli, J. P.; Hartwig, J. F., Highly Reactive, General, and Long-Lived Catalysts for Coupling Heteroaryl and Aryl Chlorides with Primary Nitrogen Nucleophiles. *Angew. Chem. Int. Ed.* **2005,** *44*, 1371–1375.

NMR Spectra

^{1}H NMR of $^{4\text{-}tBu}$stb ($CDCl_3$, 300 MHz)

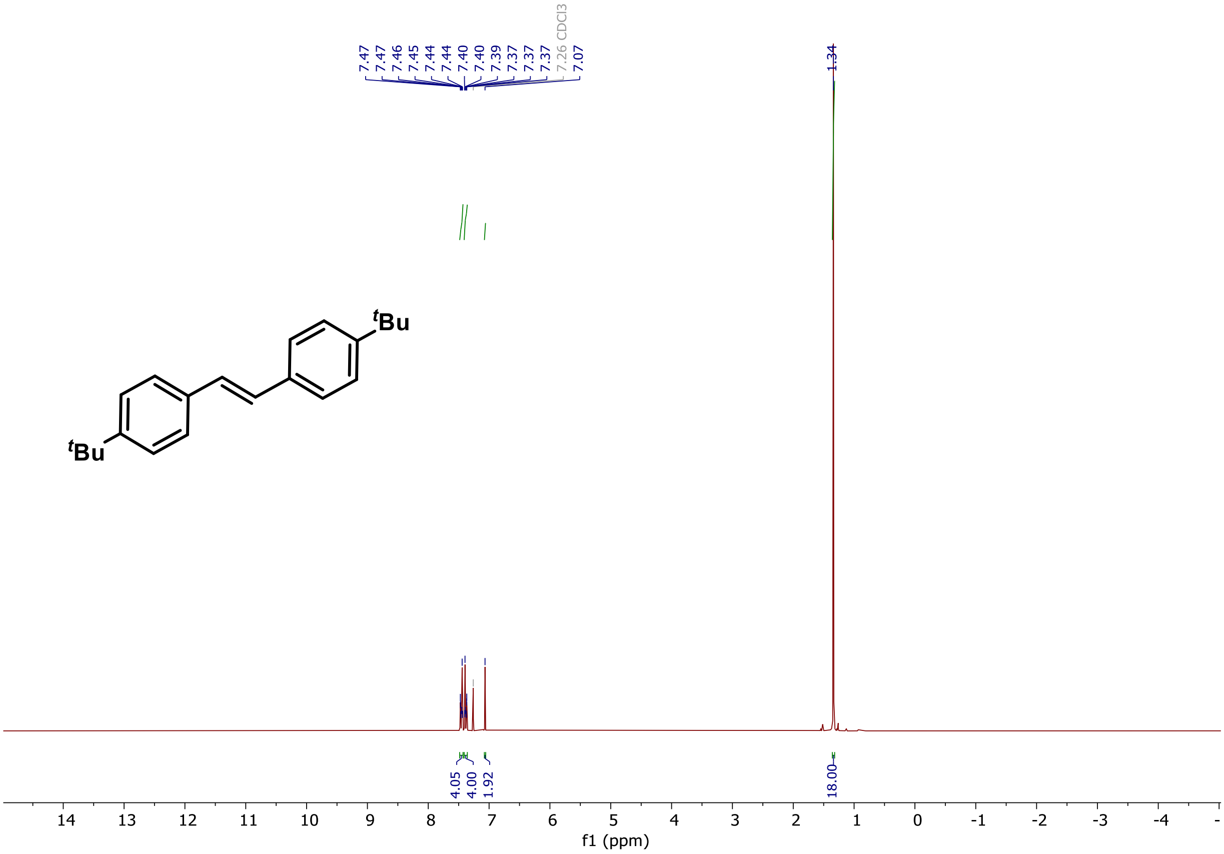

^{13}C NMR of $^{4\text{-}tBu}$stb ($CDCl_3$, 75 MHz)

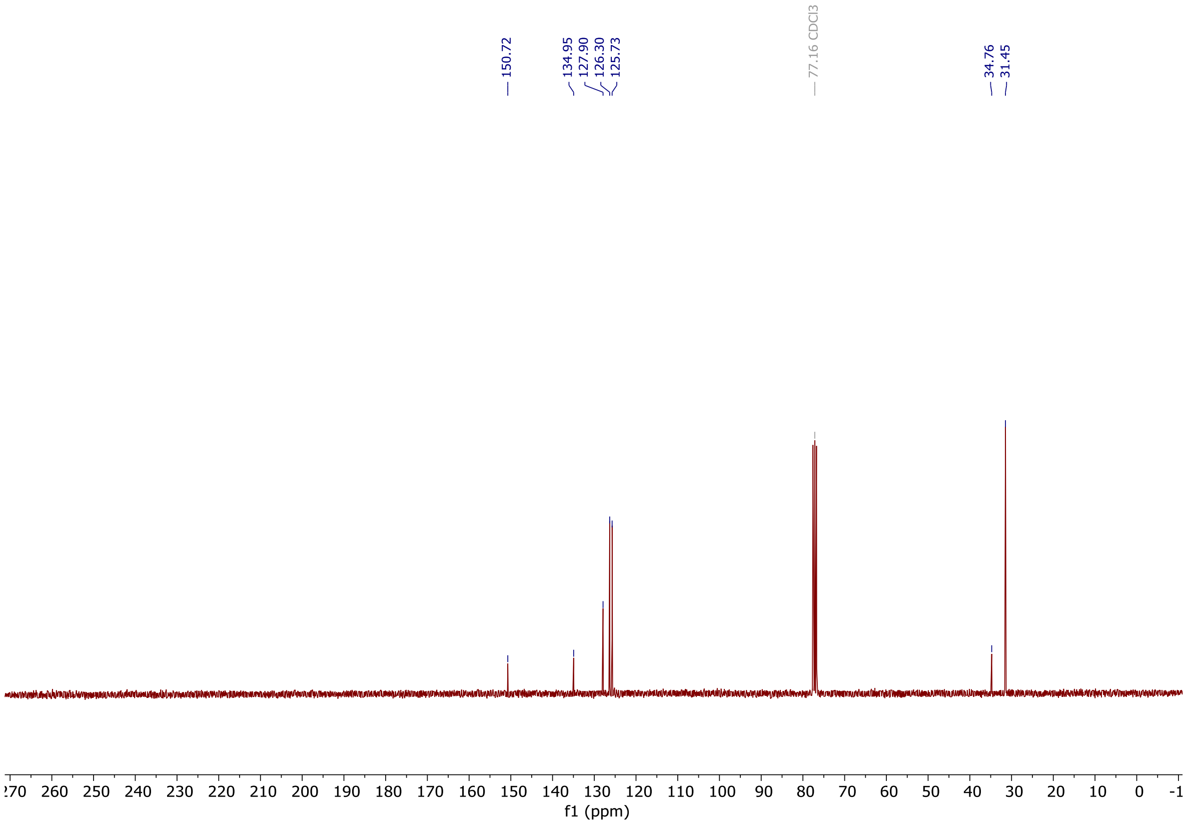

^{1}H NMR of Ni($^{4\text{-}tBu}$stb)$_3$ (−80 °C, 500 MHz, THF-d_8)

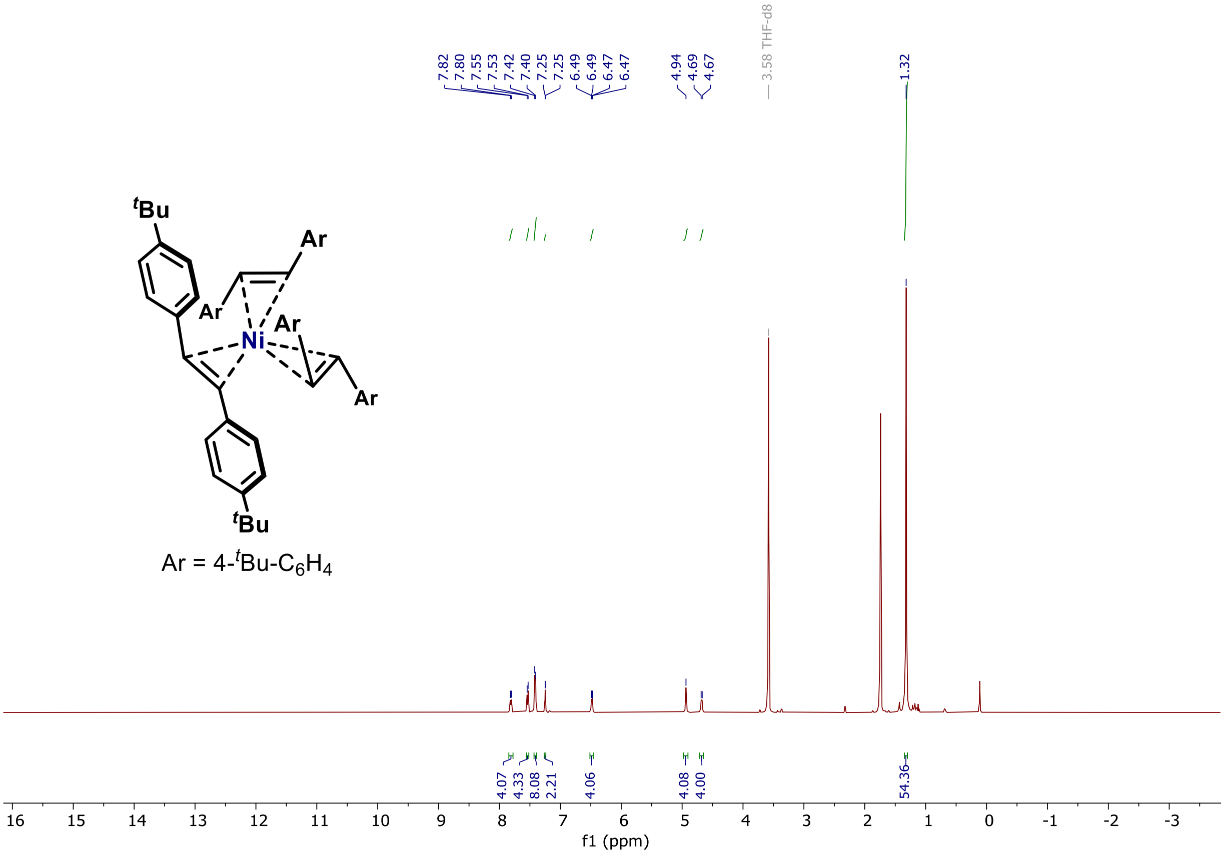

^{1}H NMR of SM1 (CDCl$_3$, 300 MHz)

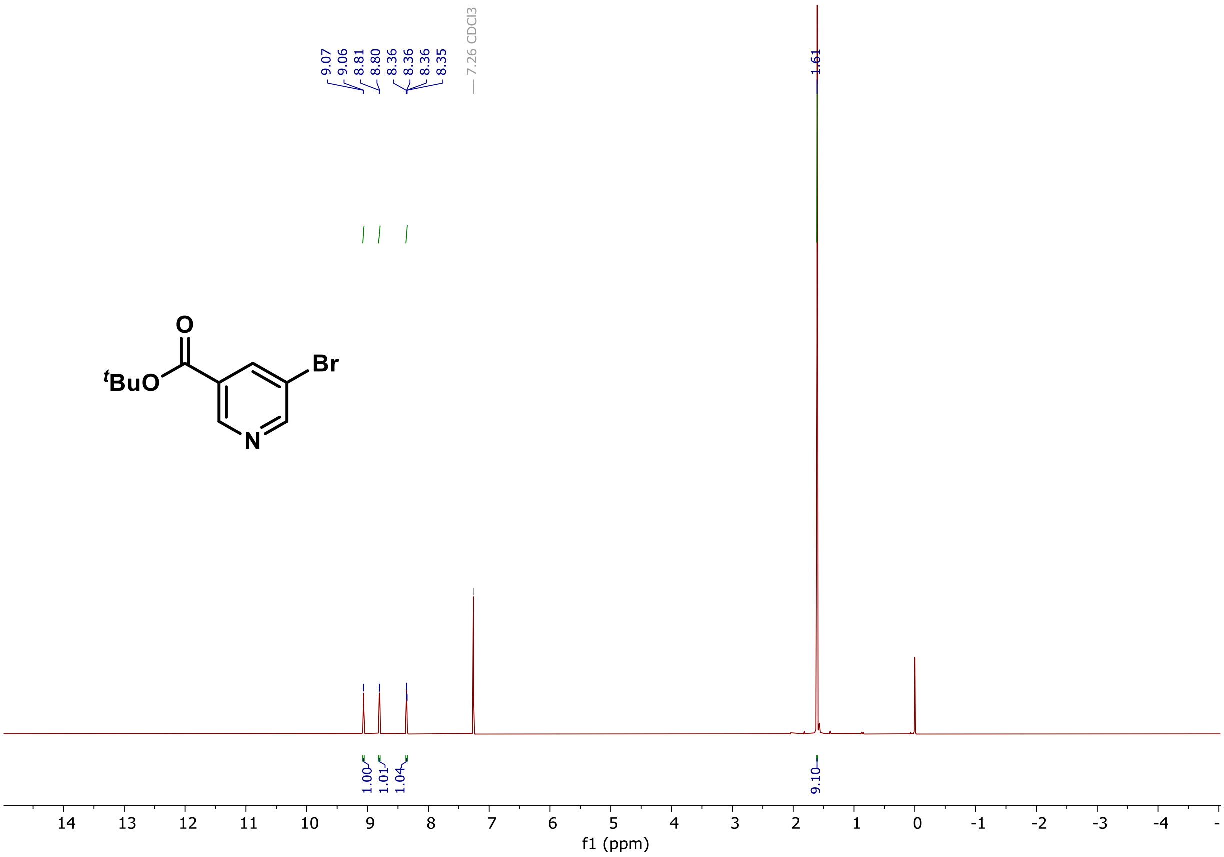

¹H NMR of SM2 ($CDCl_3$, 300 MHz)

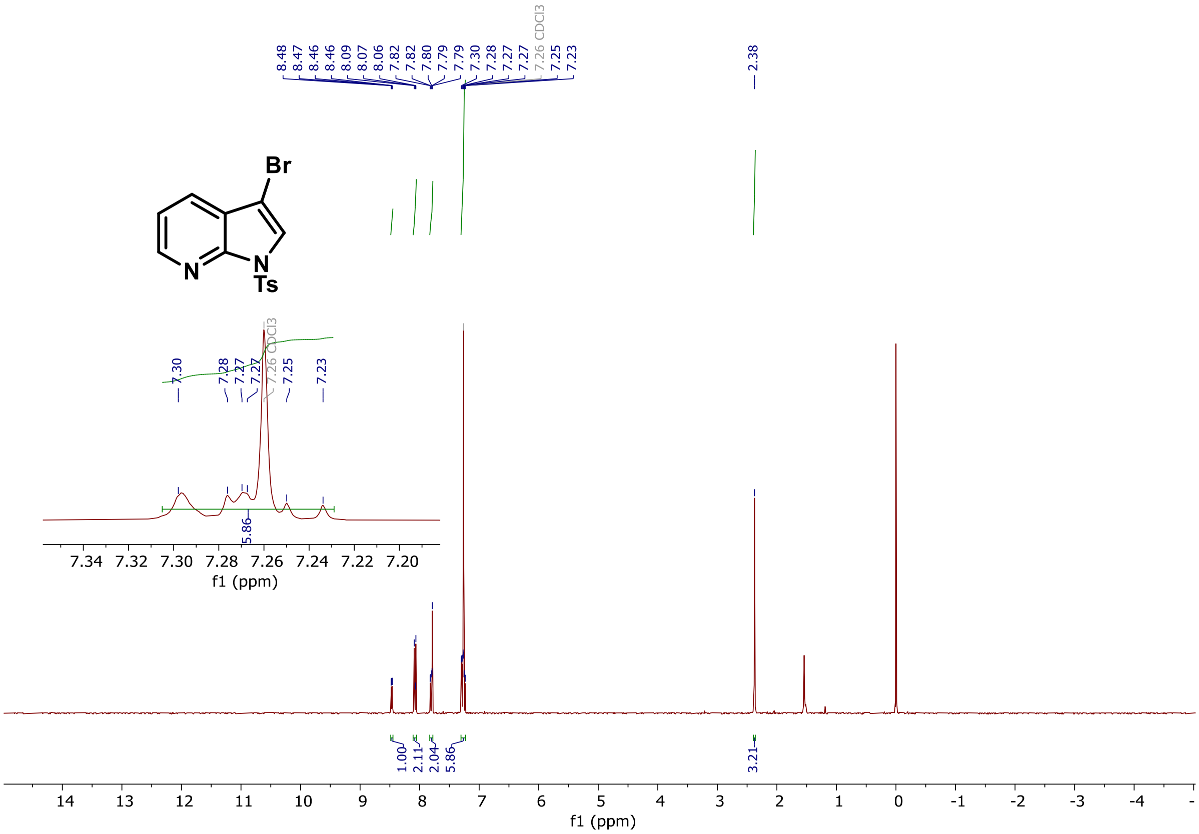

¹H NMR of 3 ($CDCl_3$, 300 MHz)

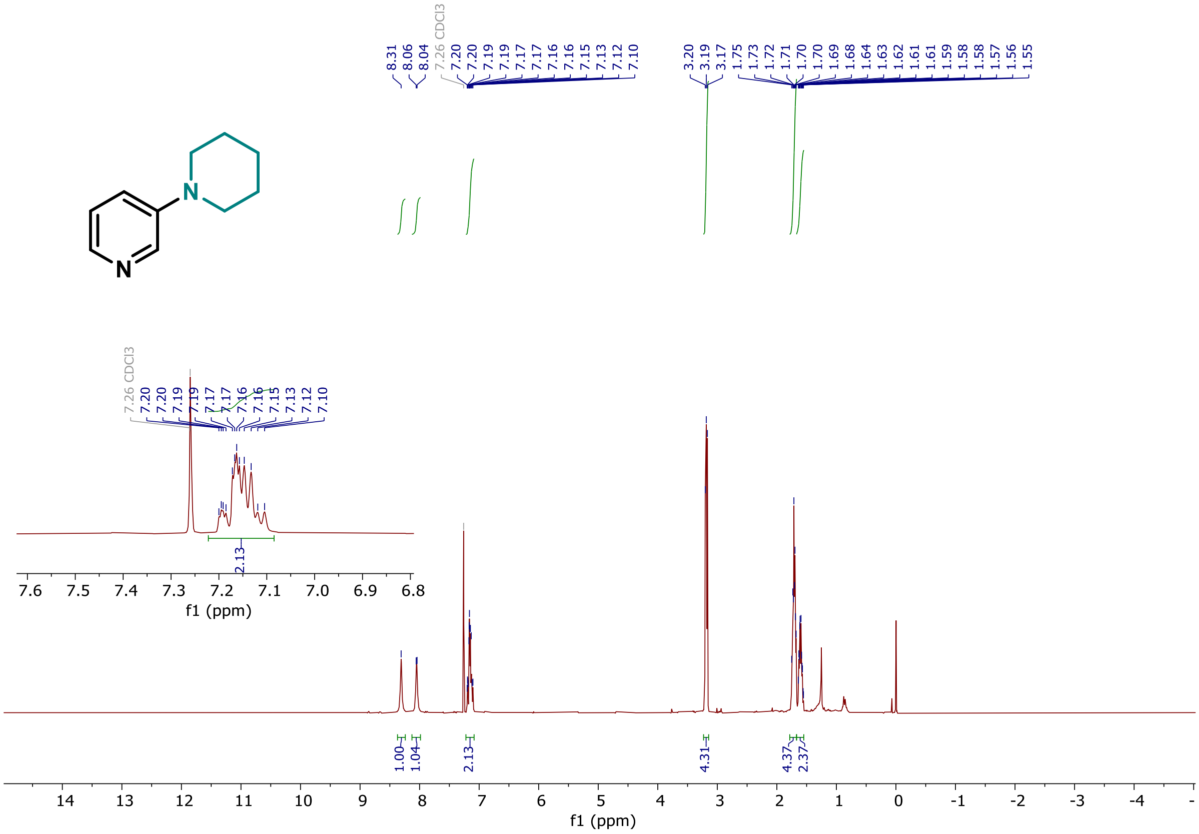

^{13}C NMR of 3 ($CDCl_3$, 101 MHz)

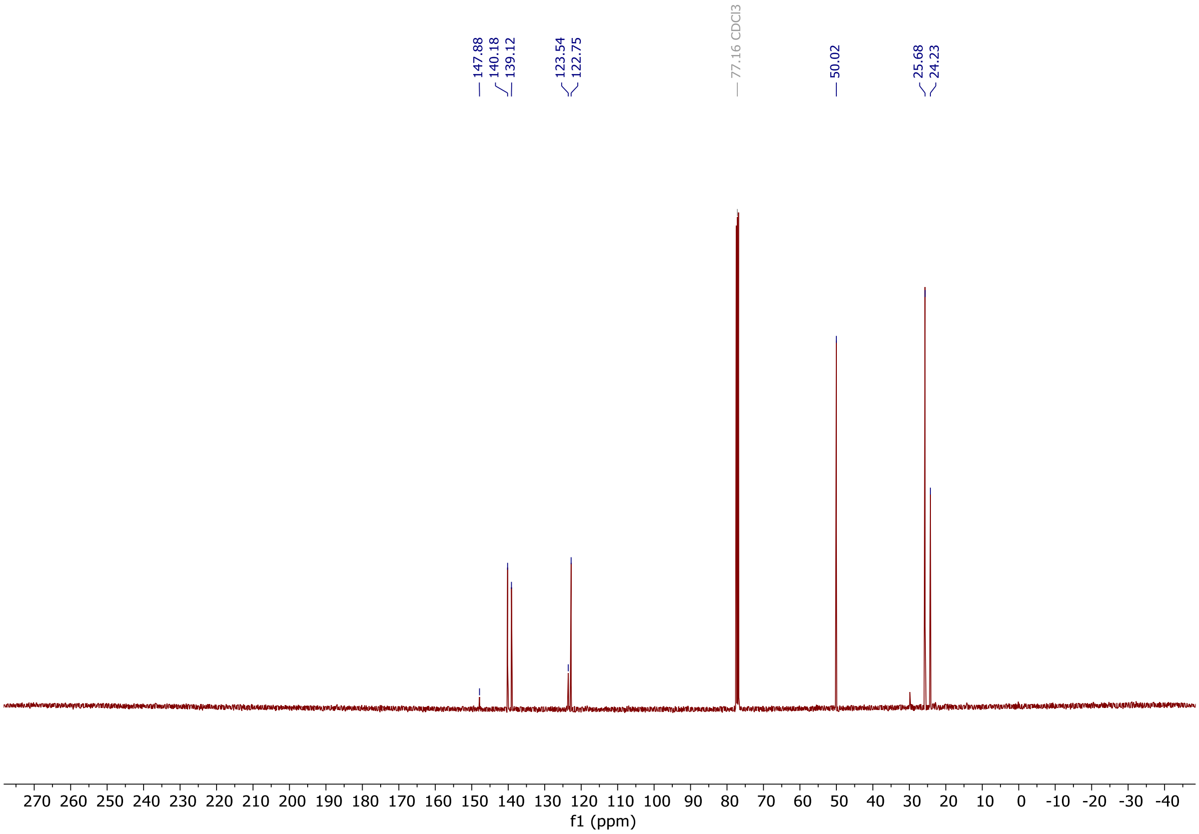

^{1}H NMR of 4 ($CDCl_3$, 300 MHz)

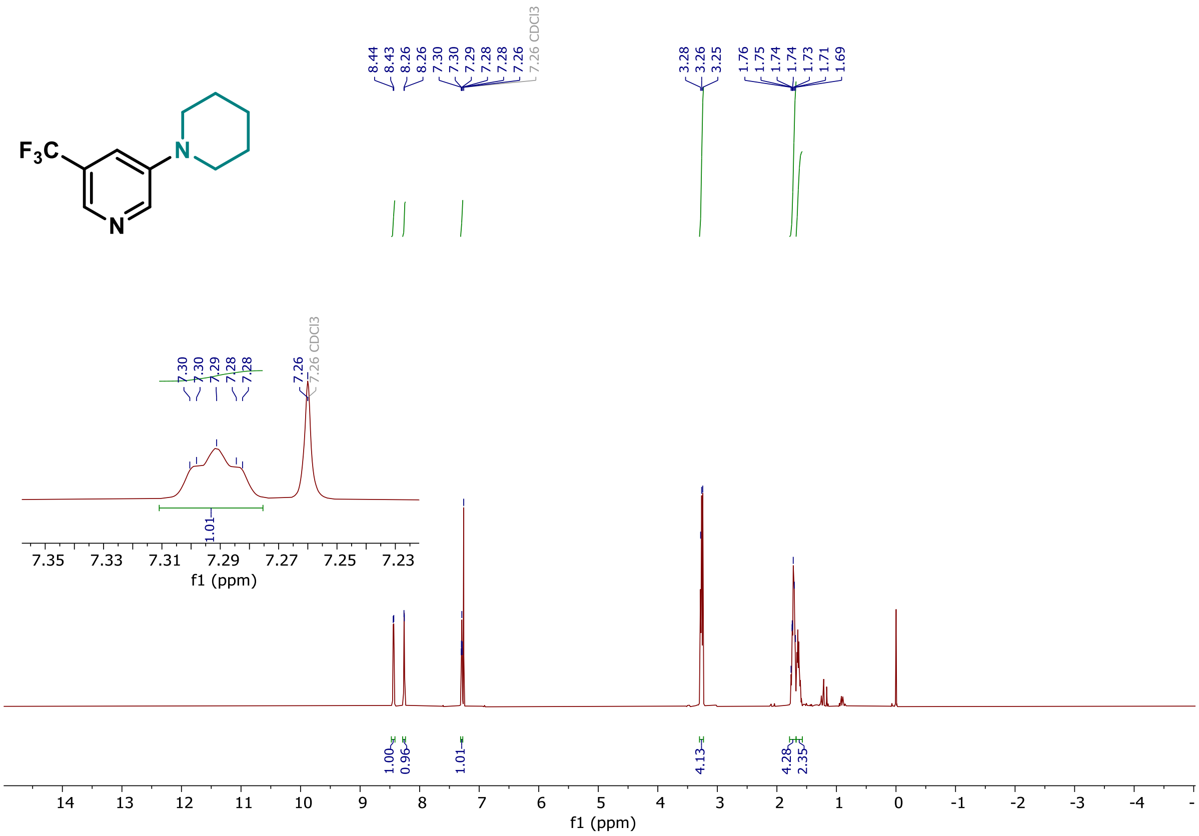

^{13}C NMR of 4 ($CDCl_3$, 75 MHz)

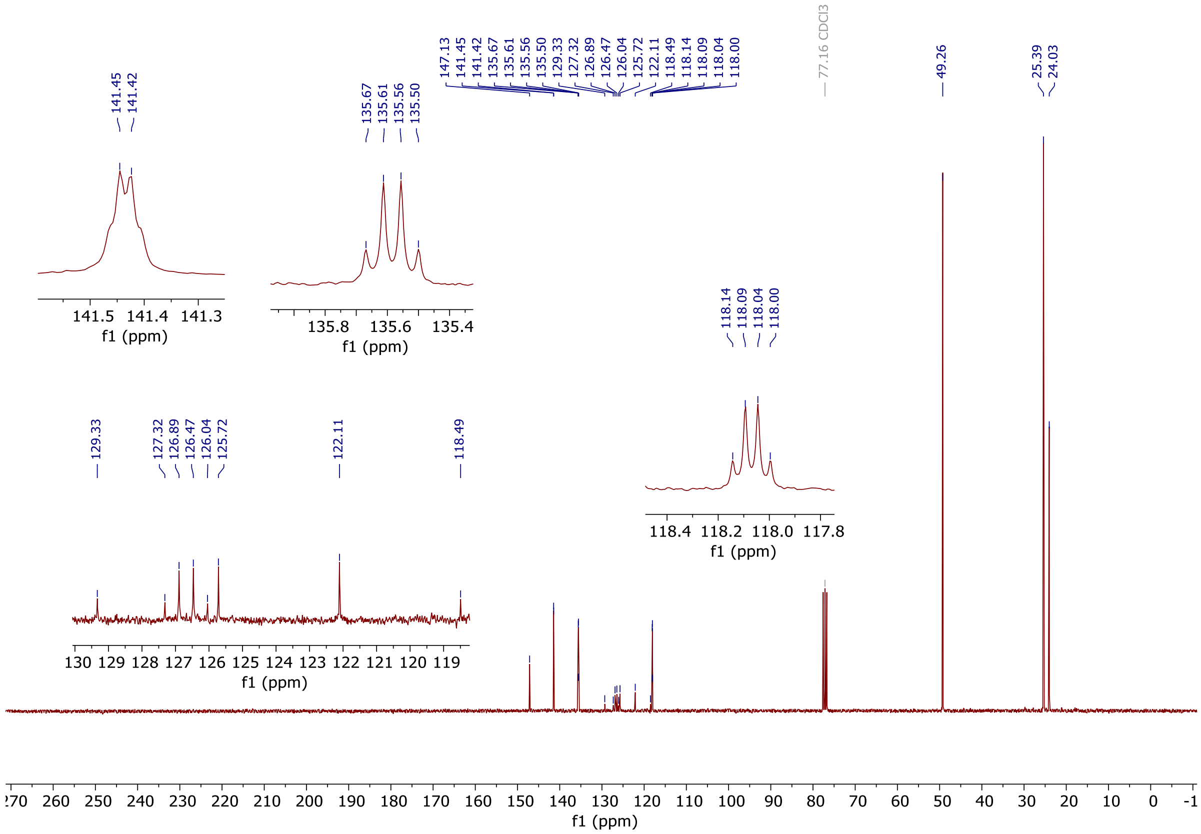

^{19}F NMR of 4 ($CDCl_3$, 282 MHz)

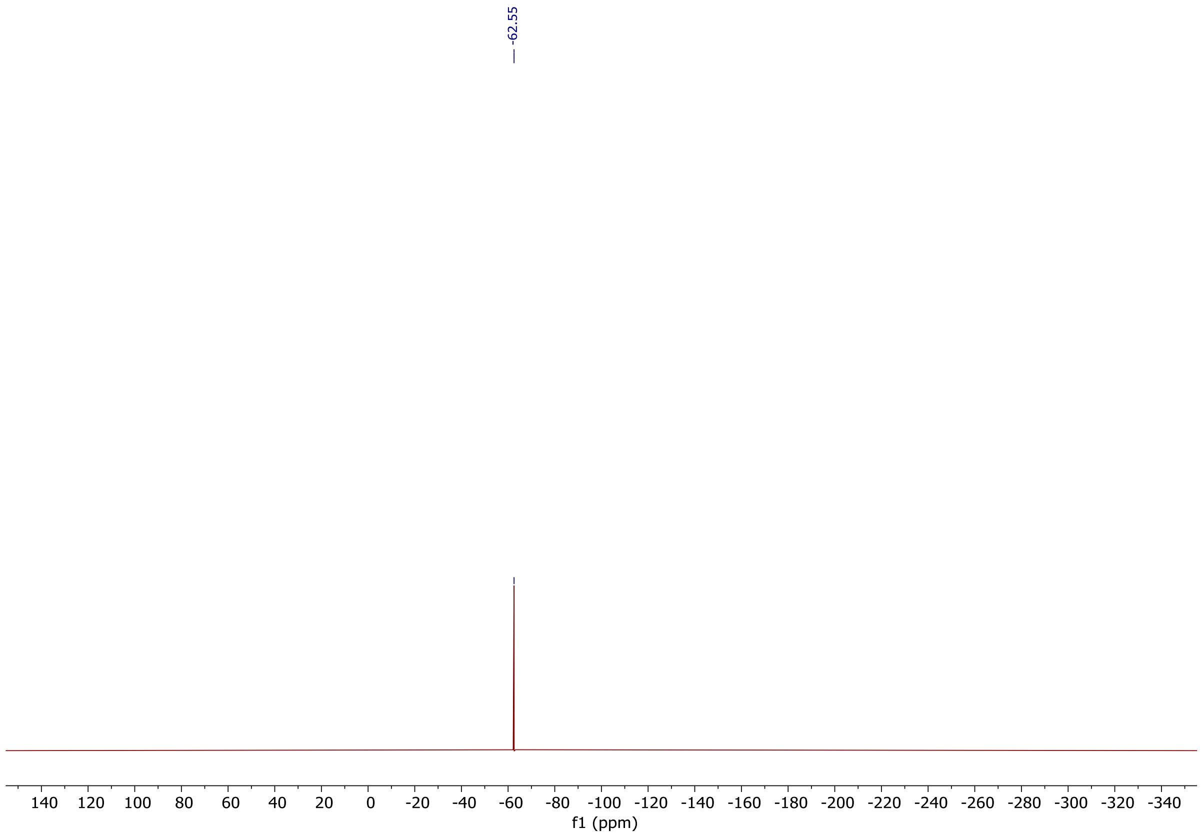

^{1}H NMR of 5 ($CDCl_3$, 400 MHz)

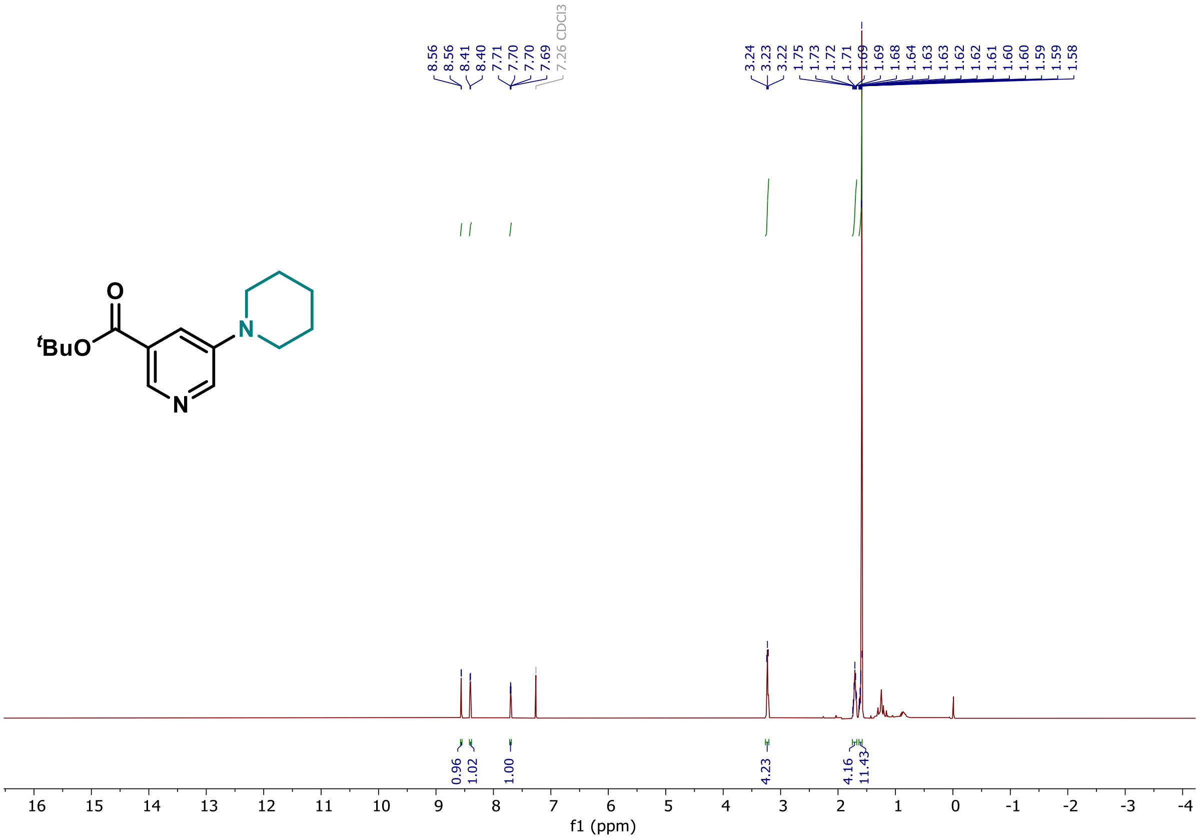

^{13}C NMR of 5 ($CDCl_3$, 101 MHz)

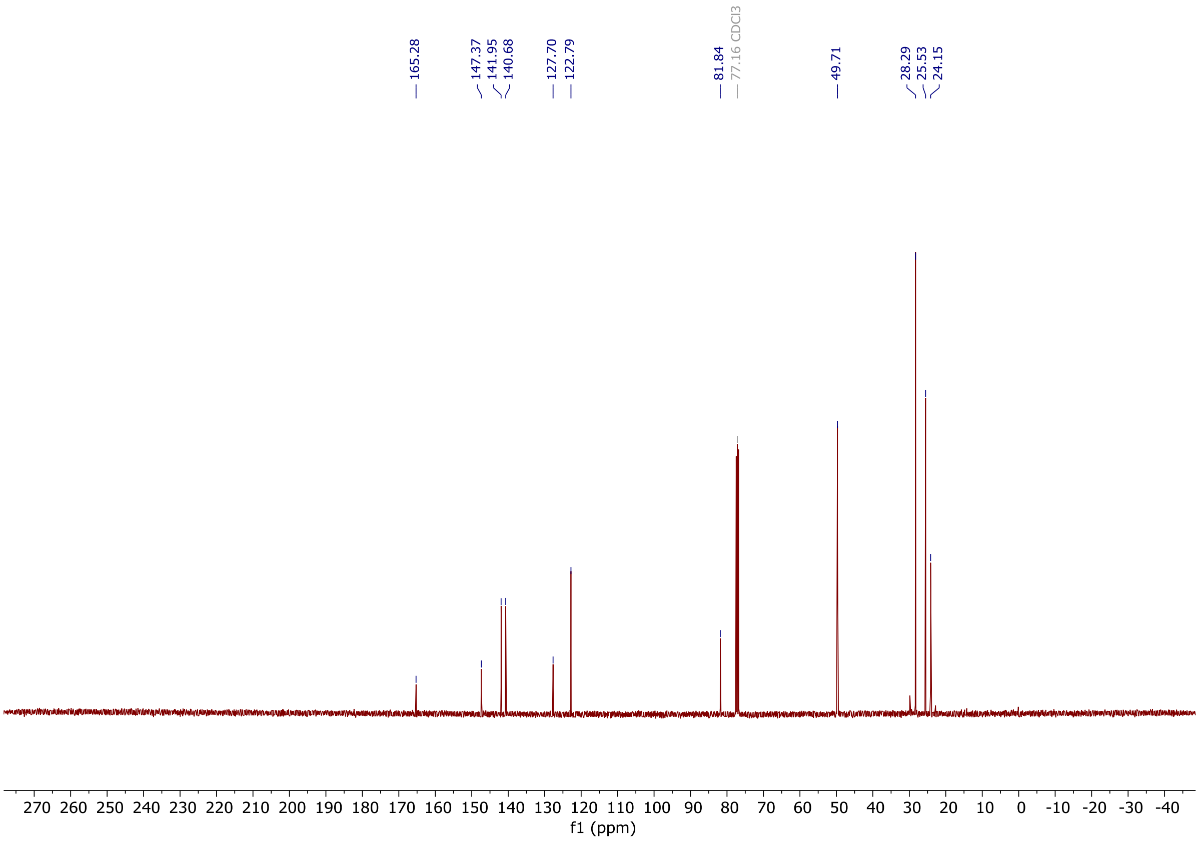

^{1}H NMR of 6 ($CDCl_3$, 300 MHz)

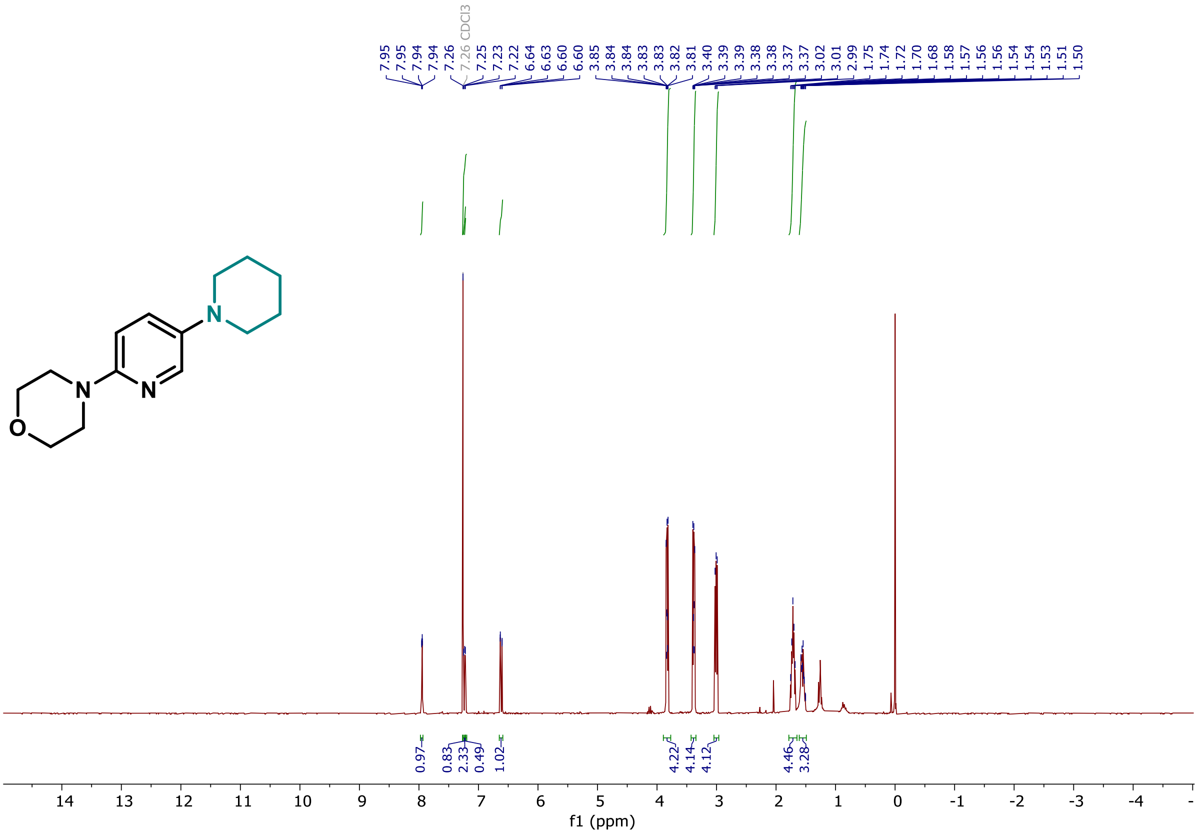

^{13}C NMR of 6 ($CDCl_3$, 101 MHz)

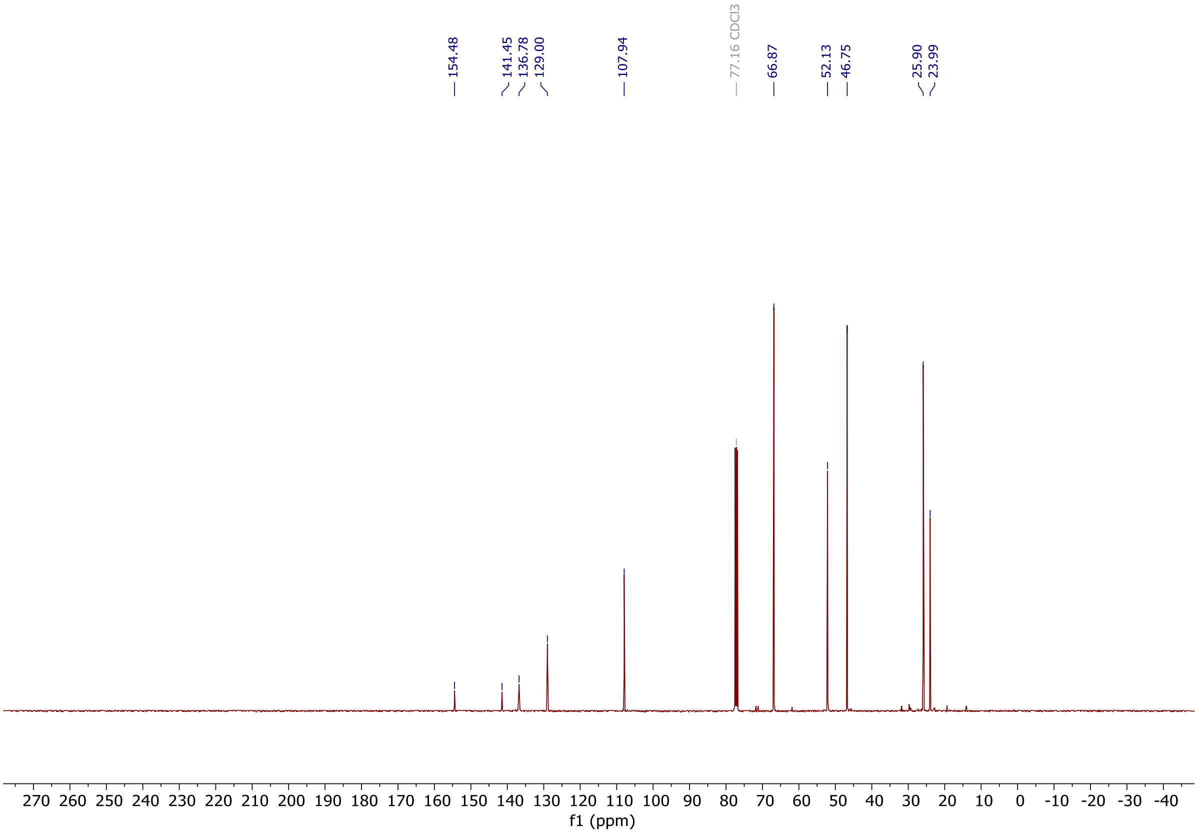

^{1}H NMR of 7 ($CDCl_3$, 300 MHz)

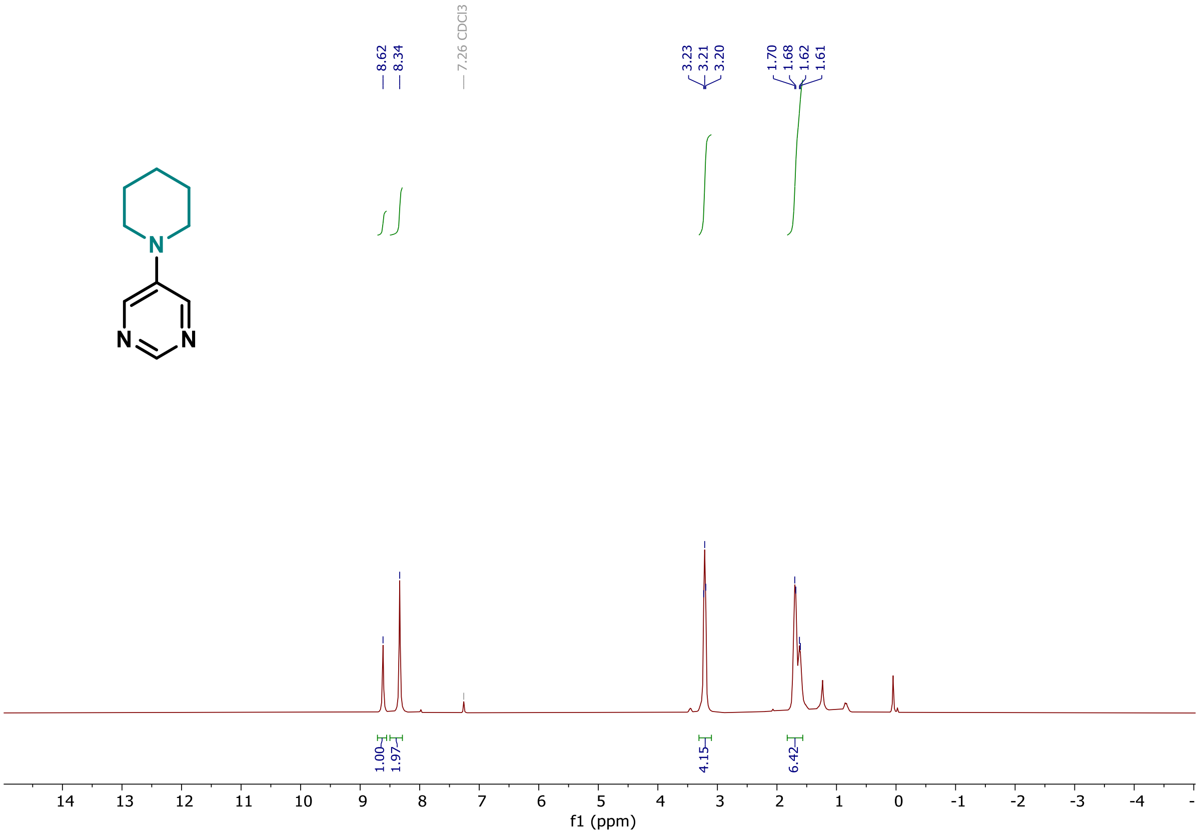

^{13}C NMR of 7 ($CDCl_3$, 75 MHz)

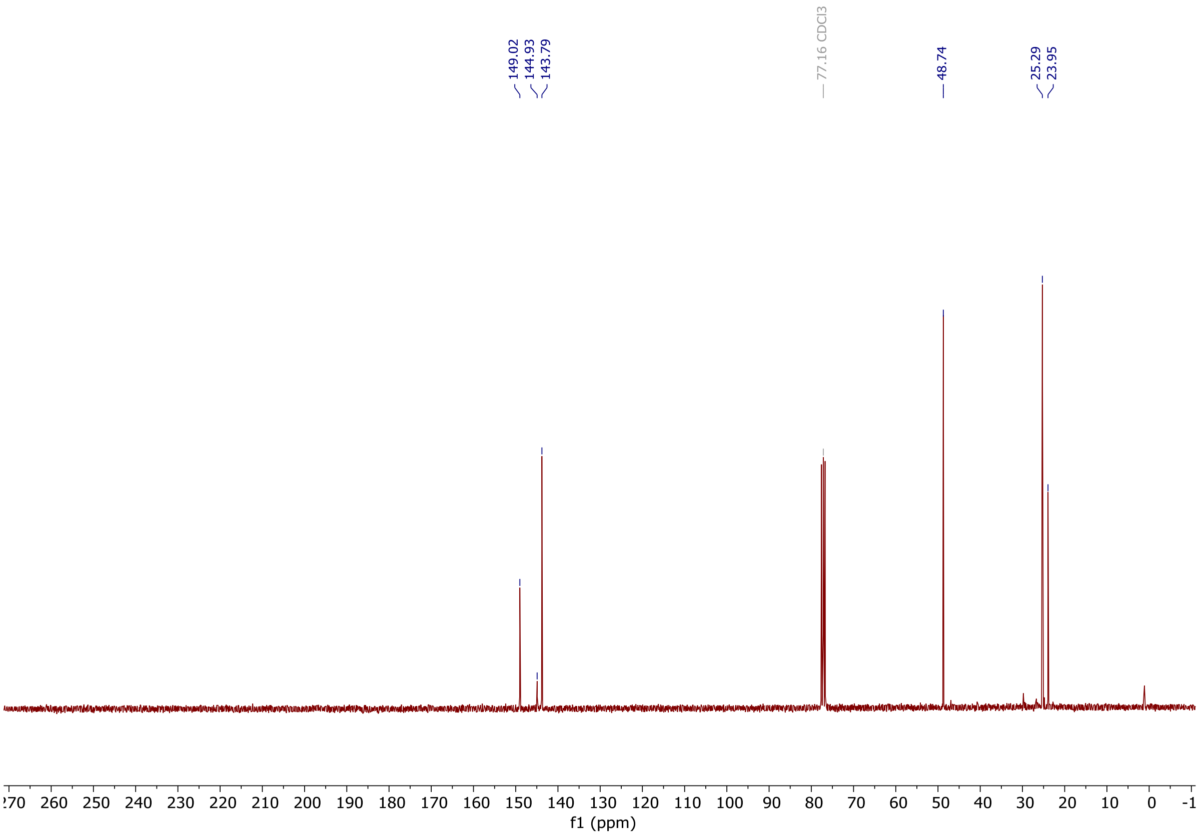

^{1}H NMR of 8 ($CDCl_3$, 400 MHz)

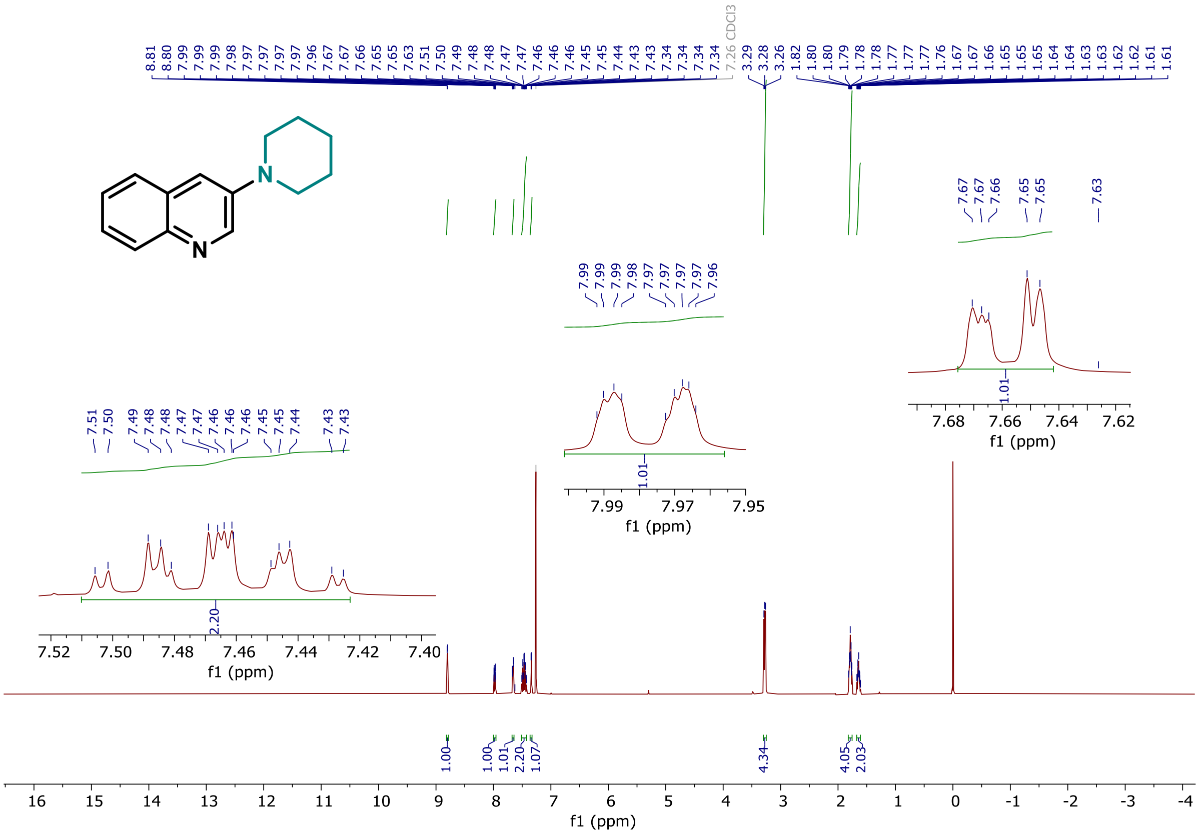

^{13}C NMR of 8 ($CDCl_3$, 101 MHz)

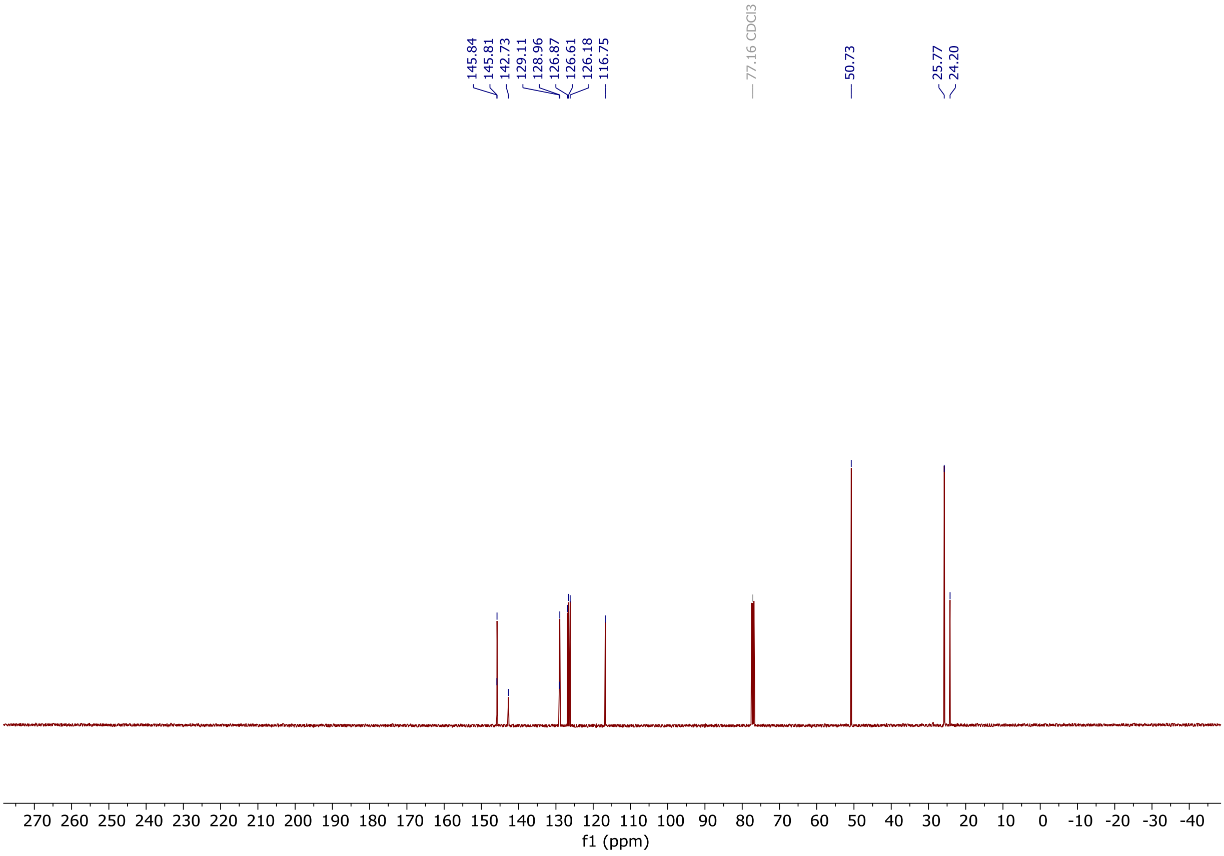

^{1}H NMR of 9 ($CDCl_3$, 300 MHz)

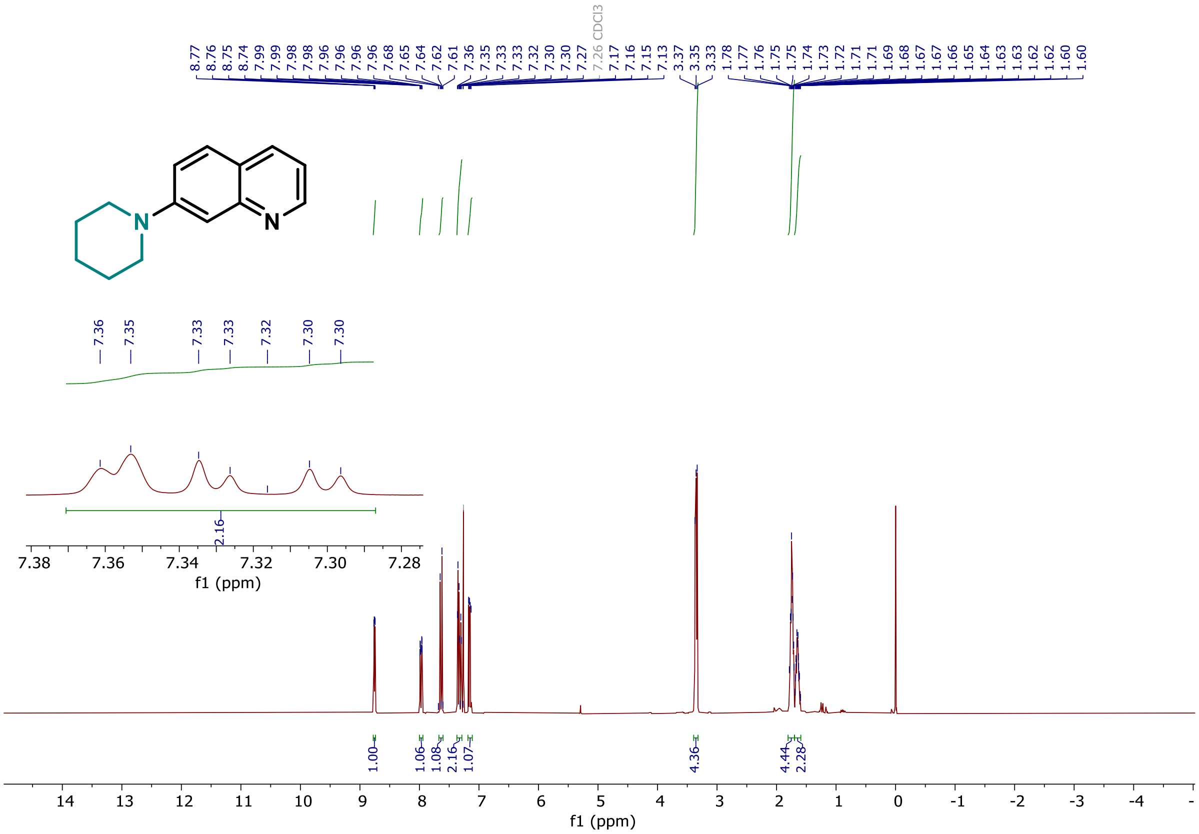

^{13}C NMR of 9 ($CDCl_3$, 101 MHz)

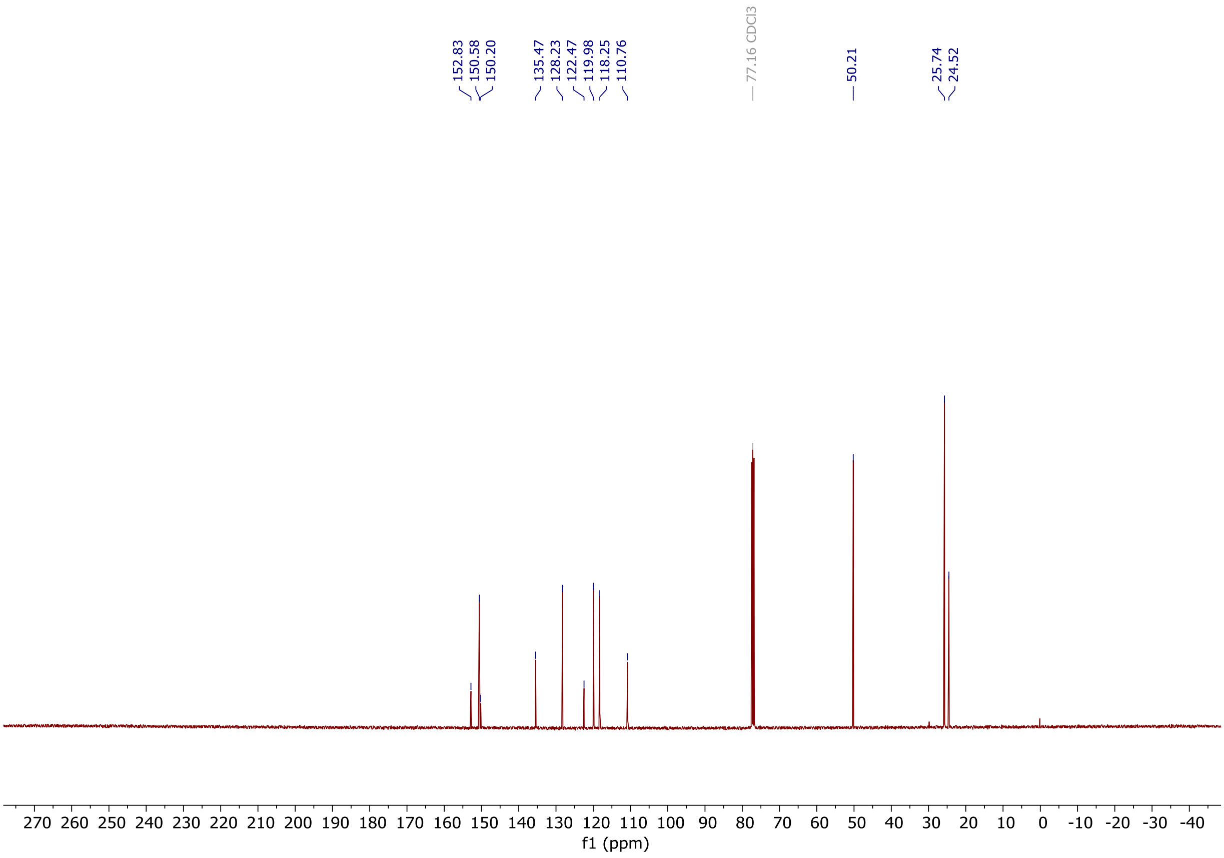

^{1}H NMR of 10 ($CDCl_3$, 400 MHz)

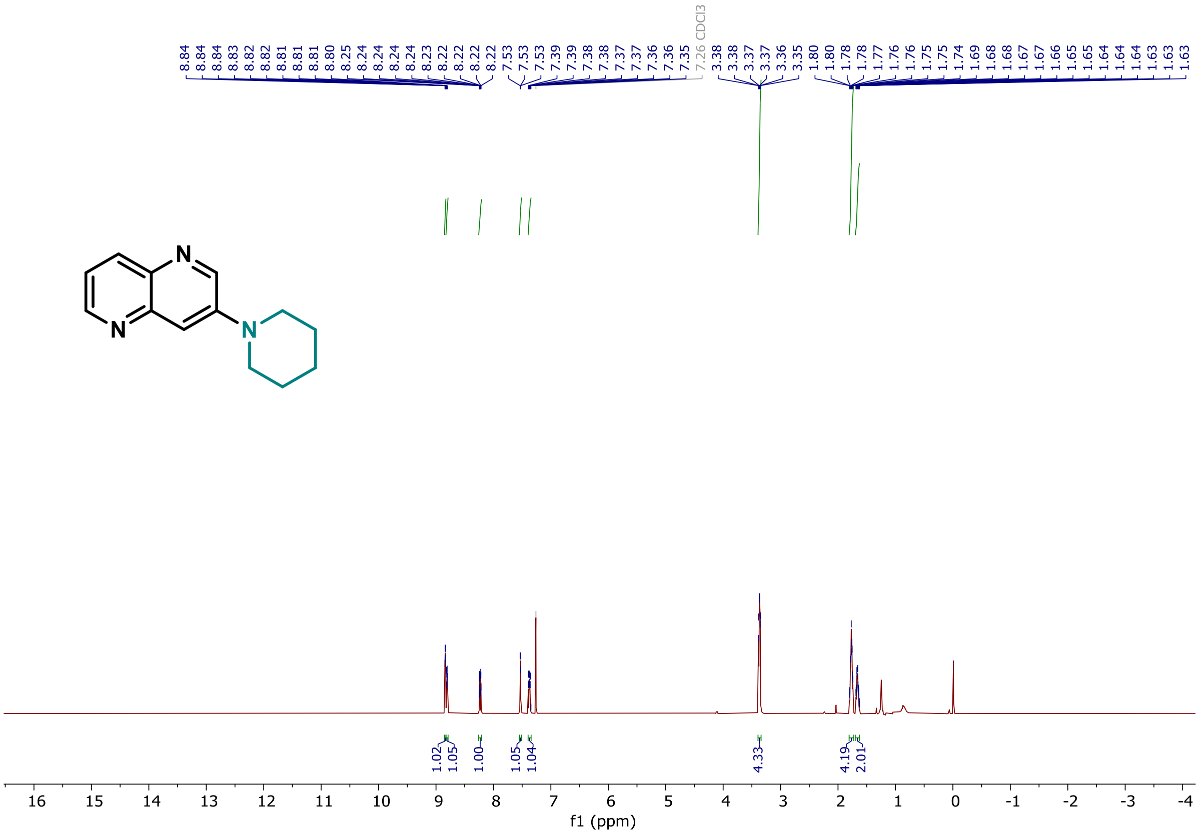

^{13}C NMR of 10 ($CDCl_3$, 101 MHz)

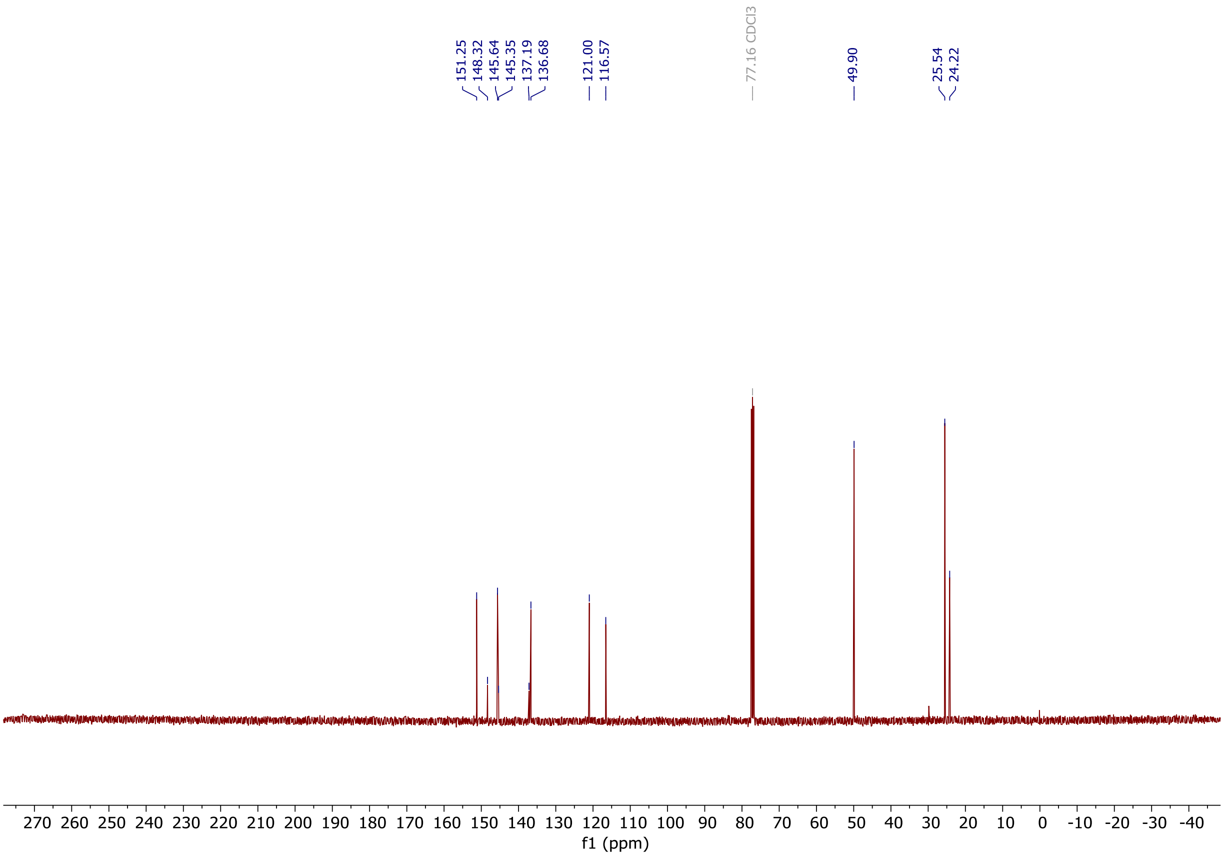

^{1}H NMR of 11 ($CDCl_3$, 400 MHz)

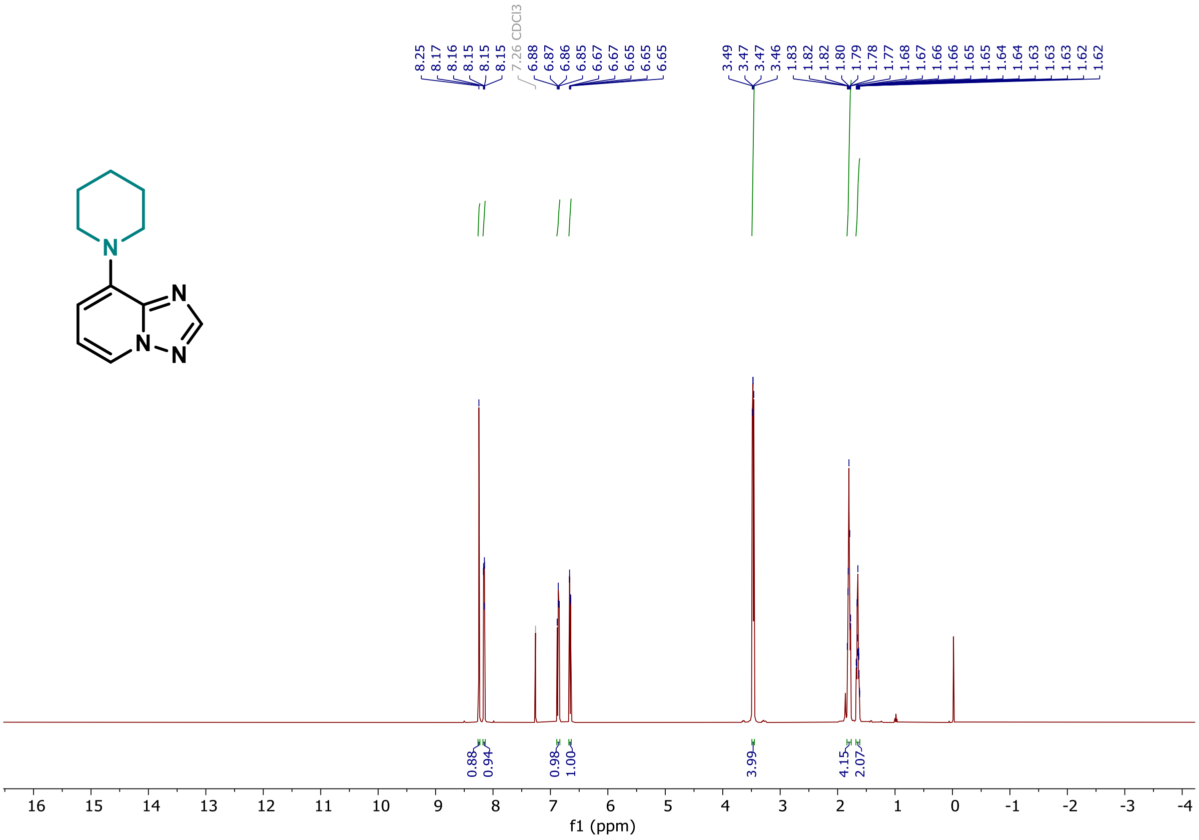

^{13}C NMR of 11 ($CDCl_3$, 101 MHz)

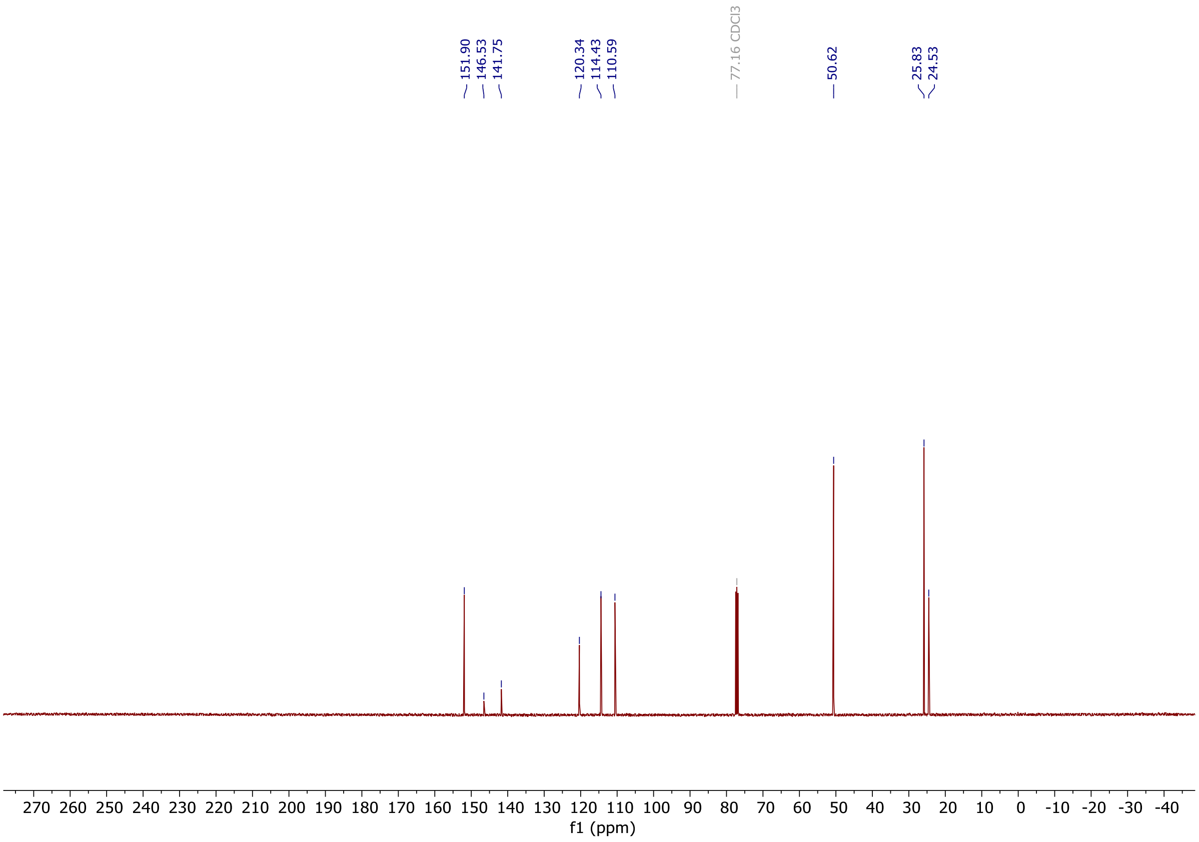

^{1}H NMR of 12 ($CDCl_3$, 400 MHz)

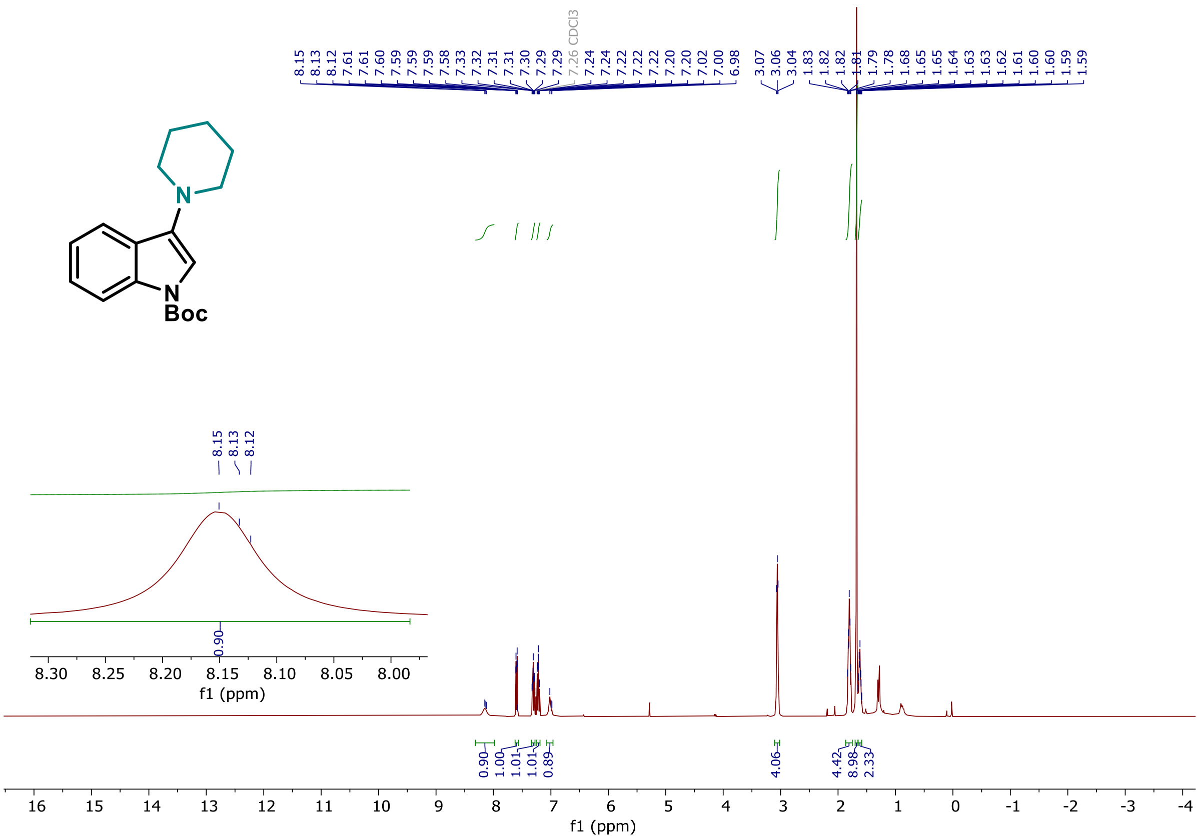

^{13}C NMR of 12 ($CDCl_3$, 101 MHz)

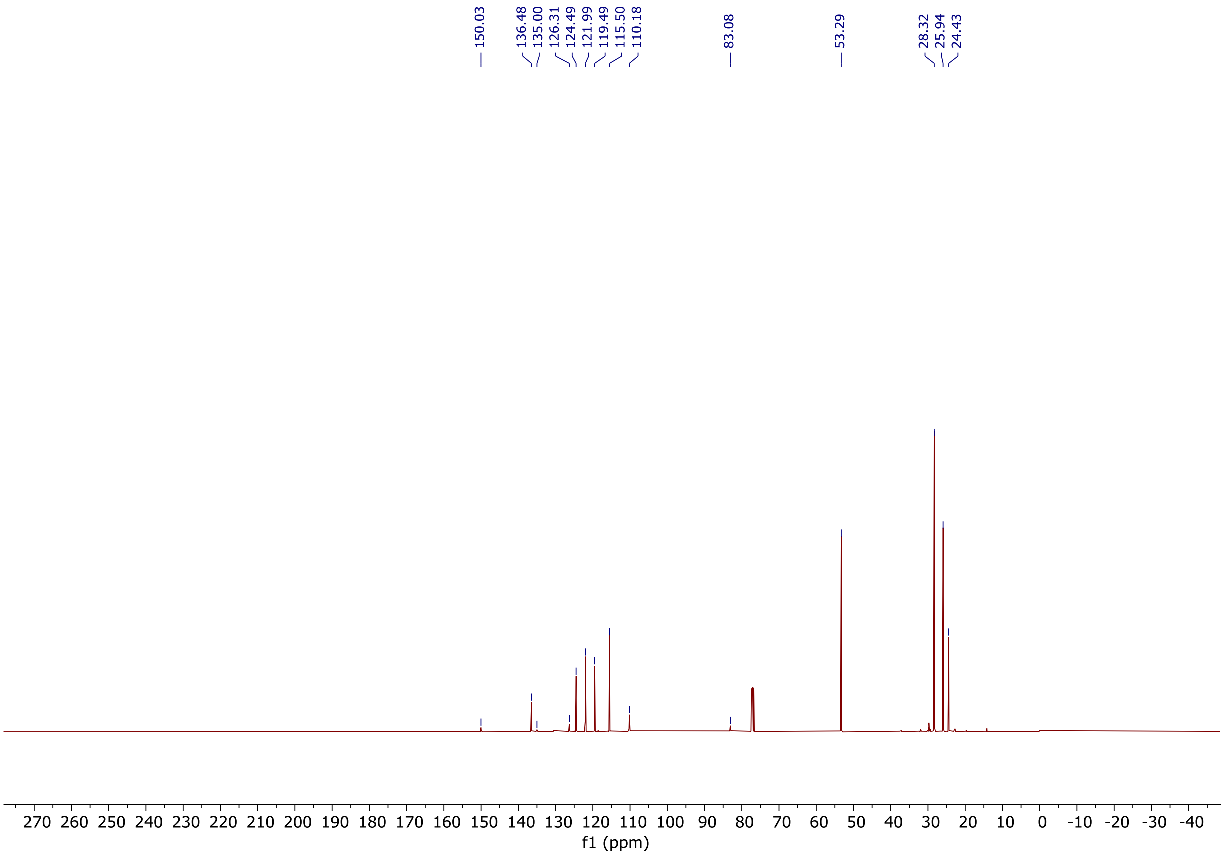

^{1}H NMR of 13 ($CDCl_3$, 400 MHz)

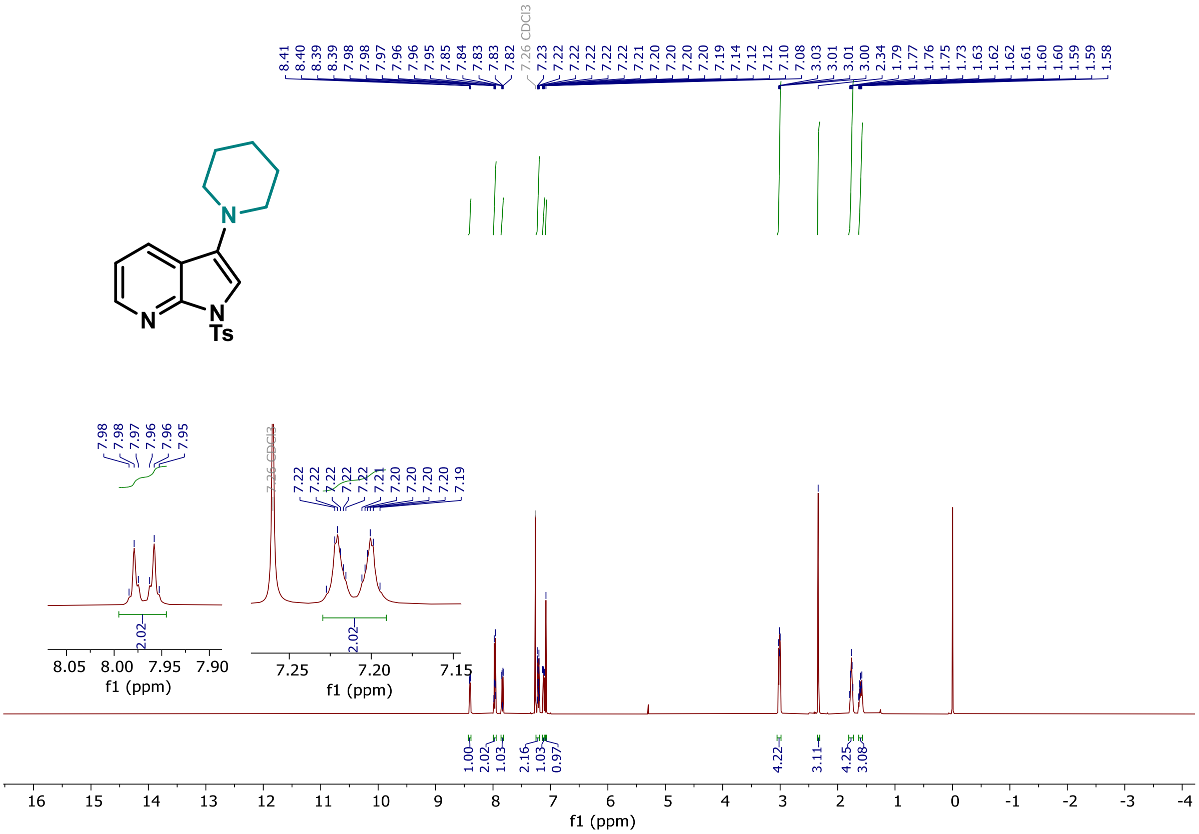

^{13}C NMR of 13 ($CDCl_3$, 101 MHz)

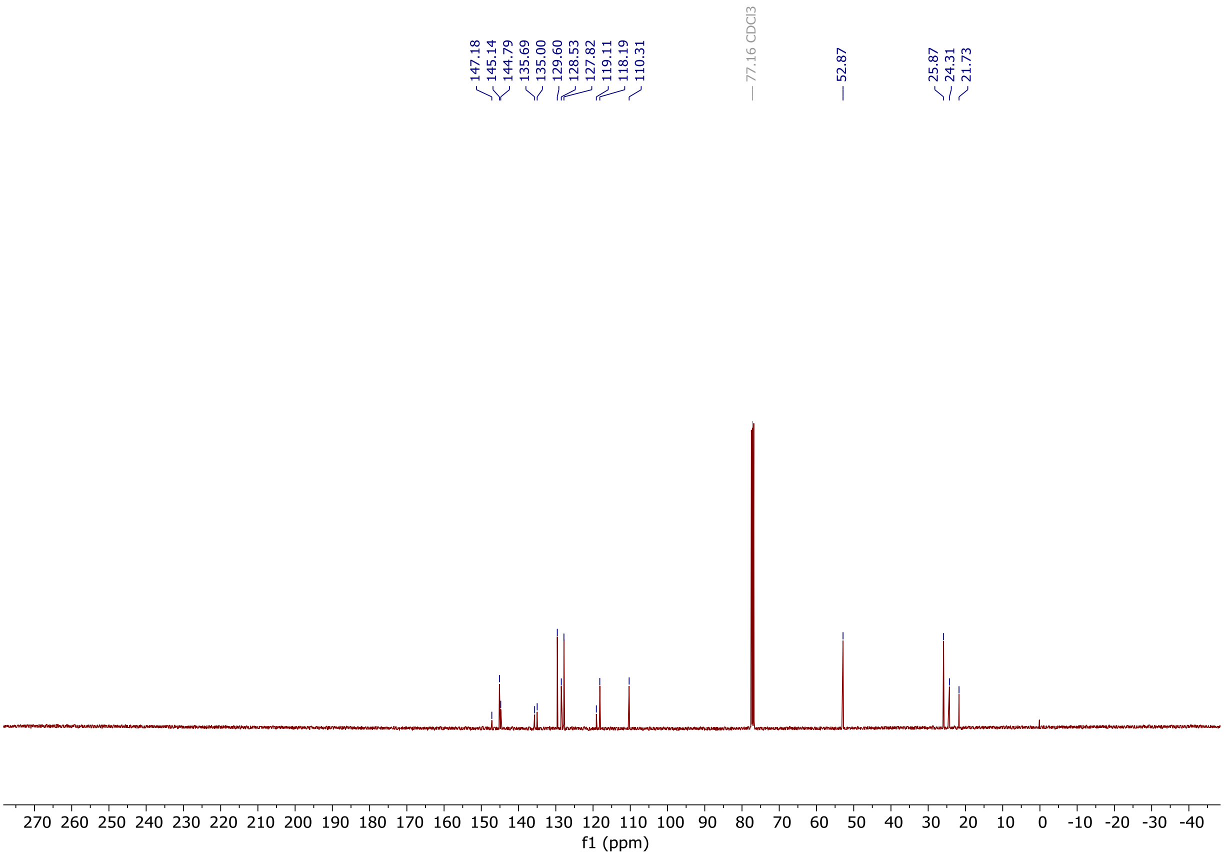

^{1}H NMR of 14 ($CDCl_3$, 300 MHz)

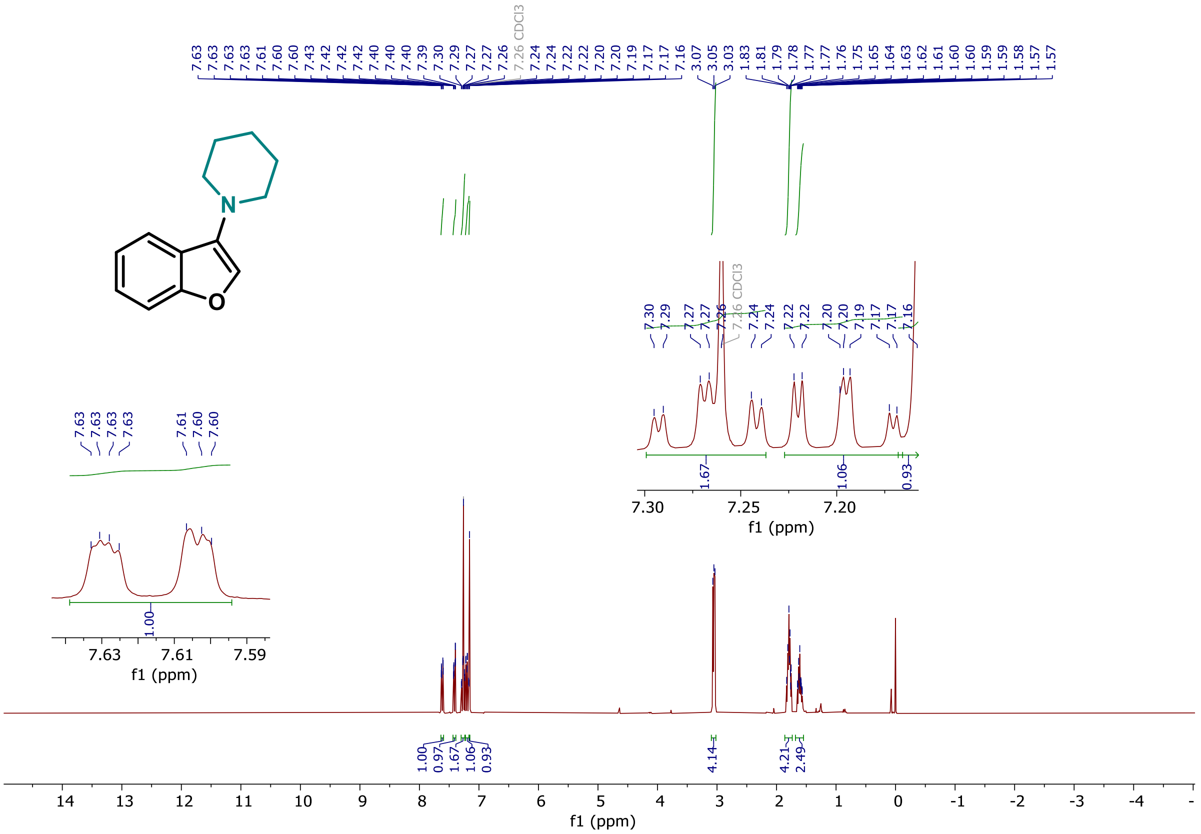

^{13}C NMR of 14 ($CDCl_3$, 101 MHz)

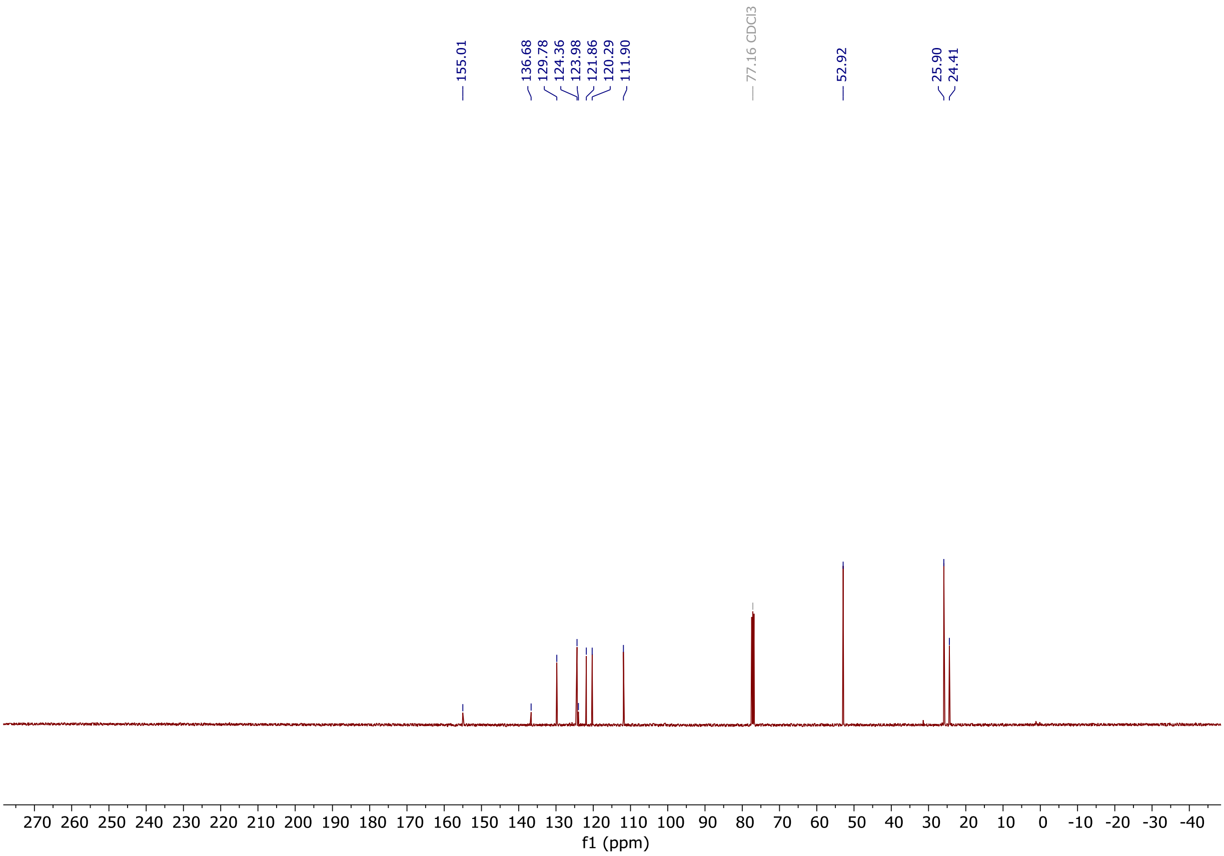

¹H NMR of 15 ($CDCl_3$, 300 MHz)

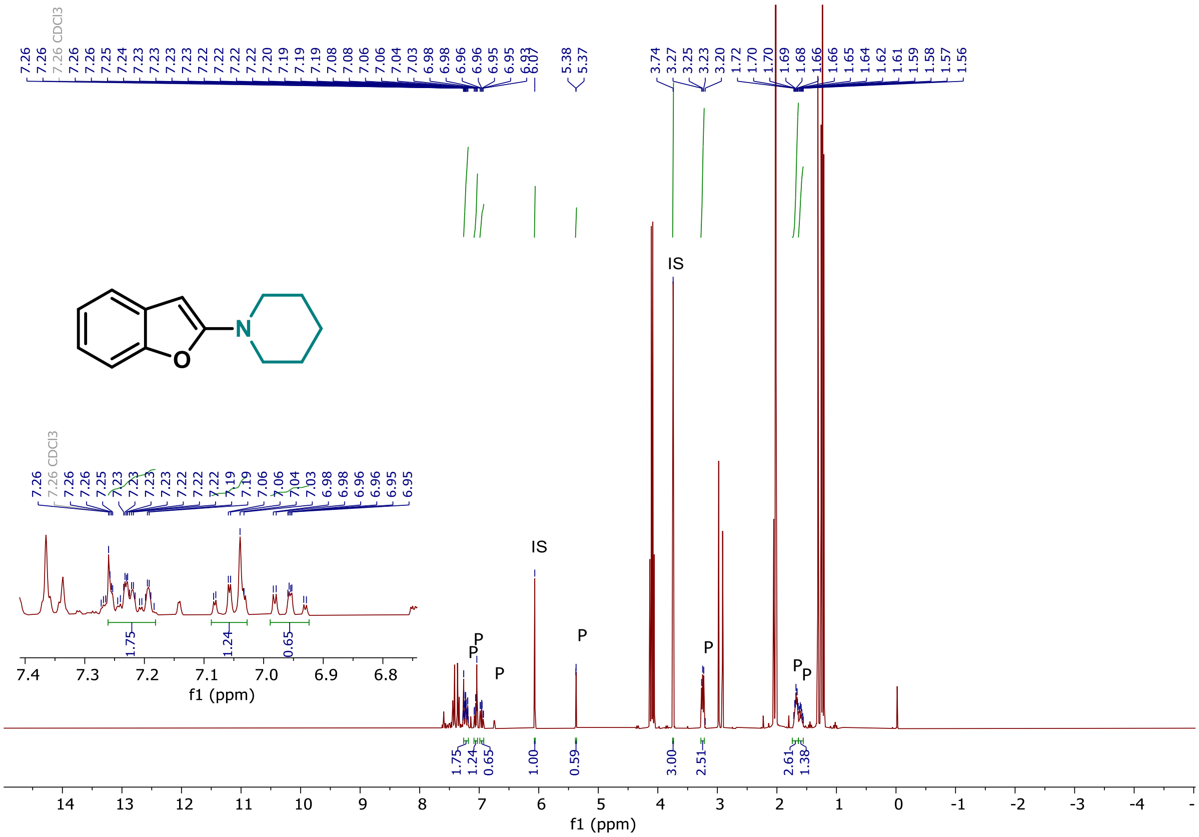

¹H NMR of 16 ($CDCl_3$, 400 MHz)

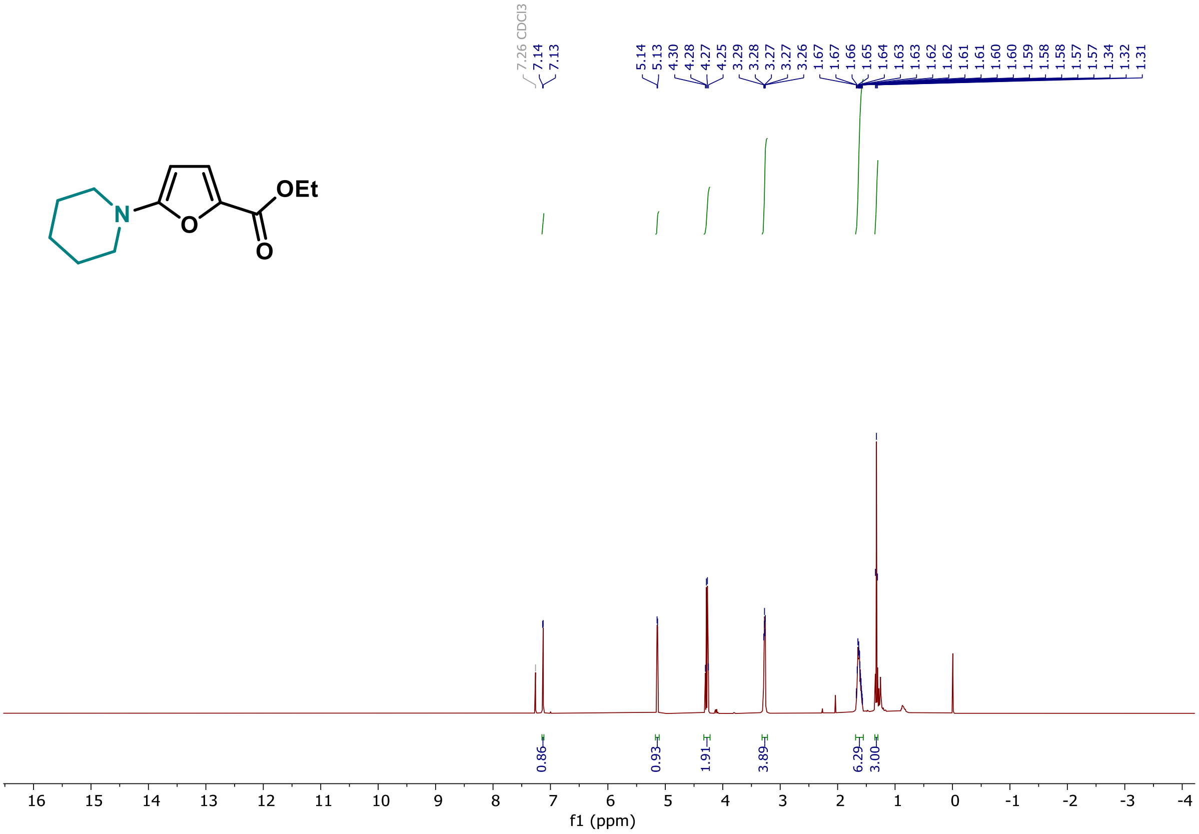

^{13}C NMR of 16 ($CDCl_3$, 101 MHz)

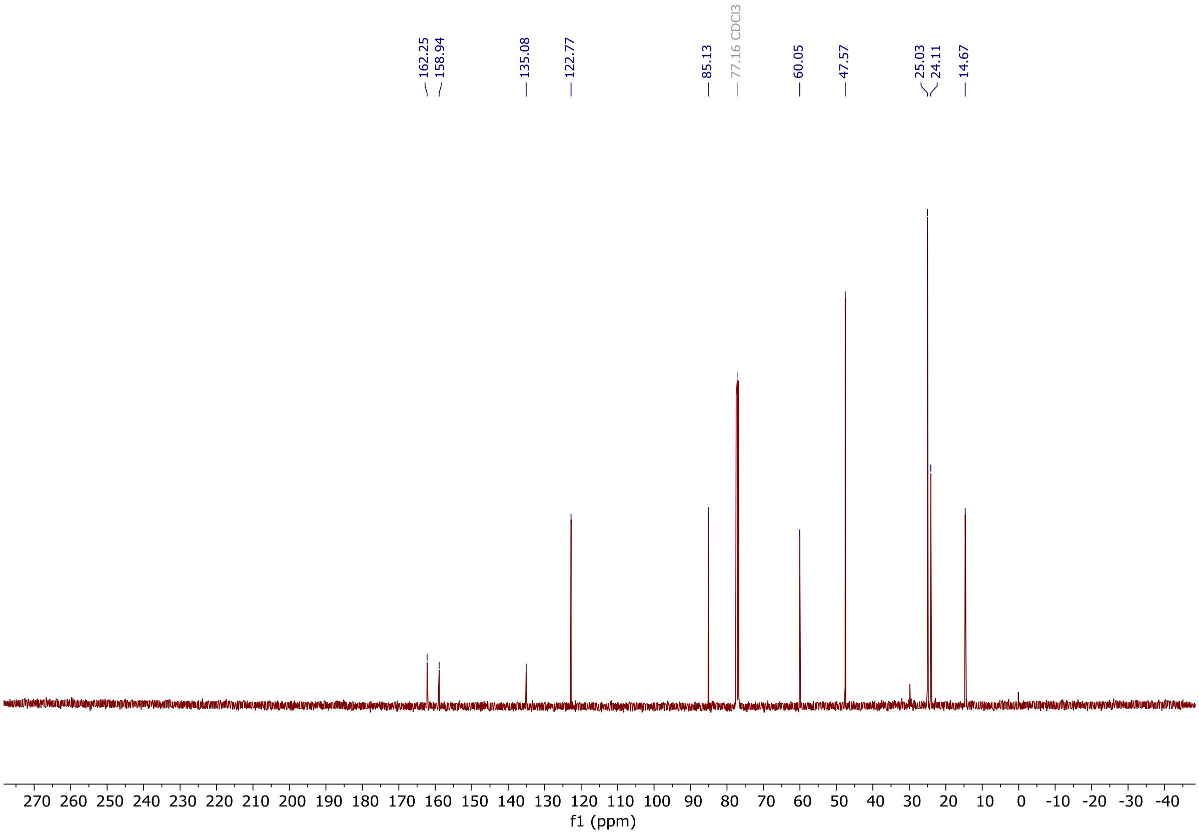

^{1}H NMR of 17 ($CDCl_3$, 400 MHz)

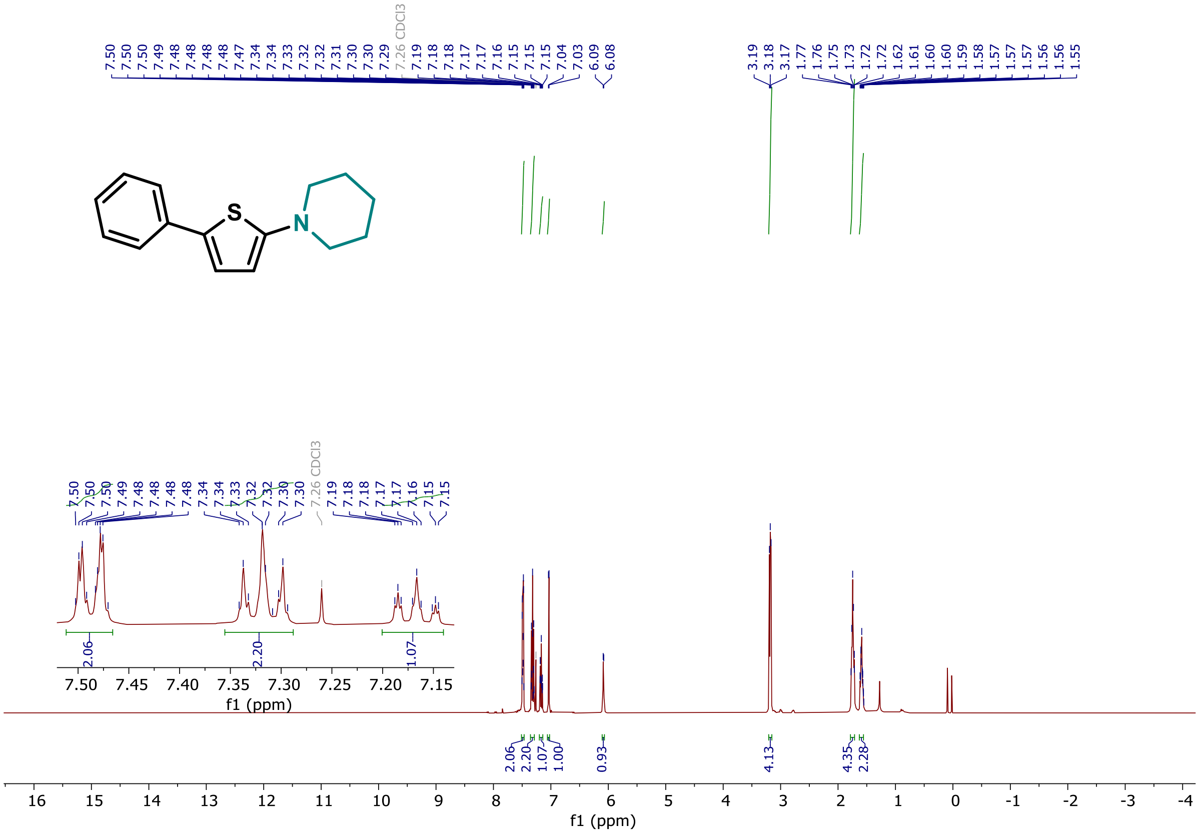

^{13}C NMR of 17 ($CDCl_3$, 101 MHz)

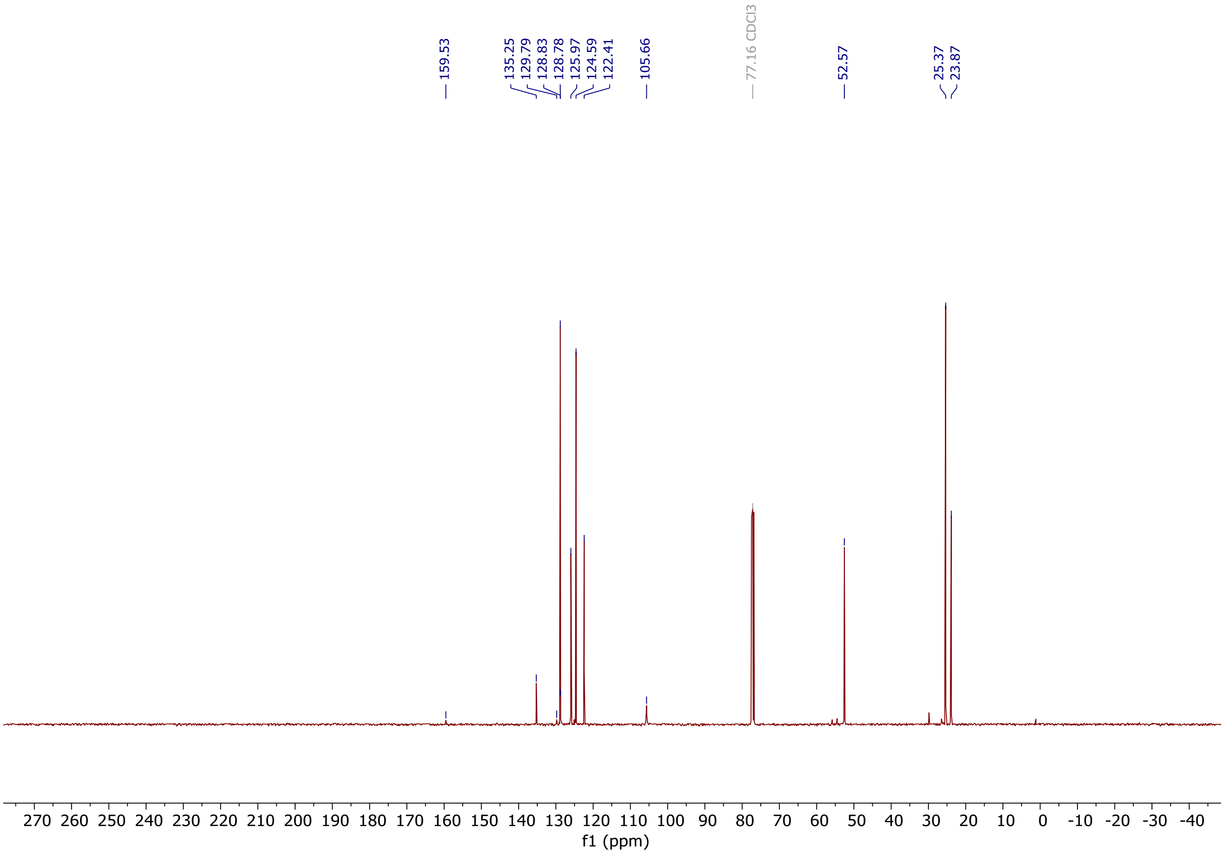

^{1}H NMR of 18 ($CDCl_3$, 400 MHz)

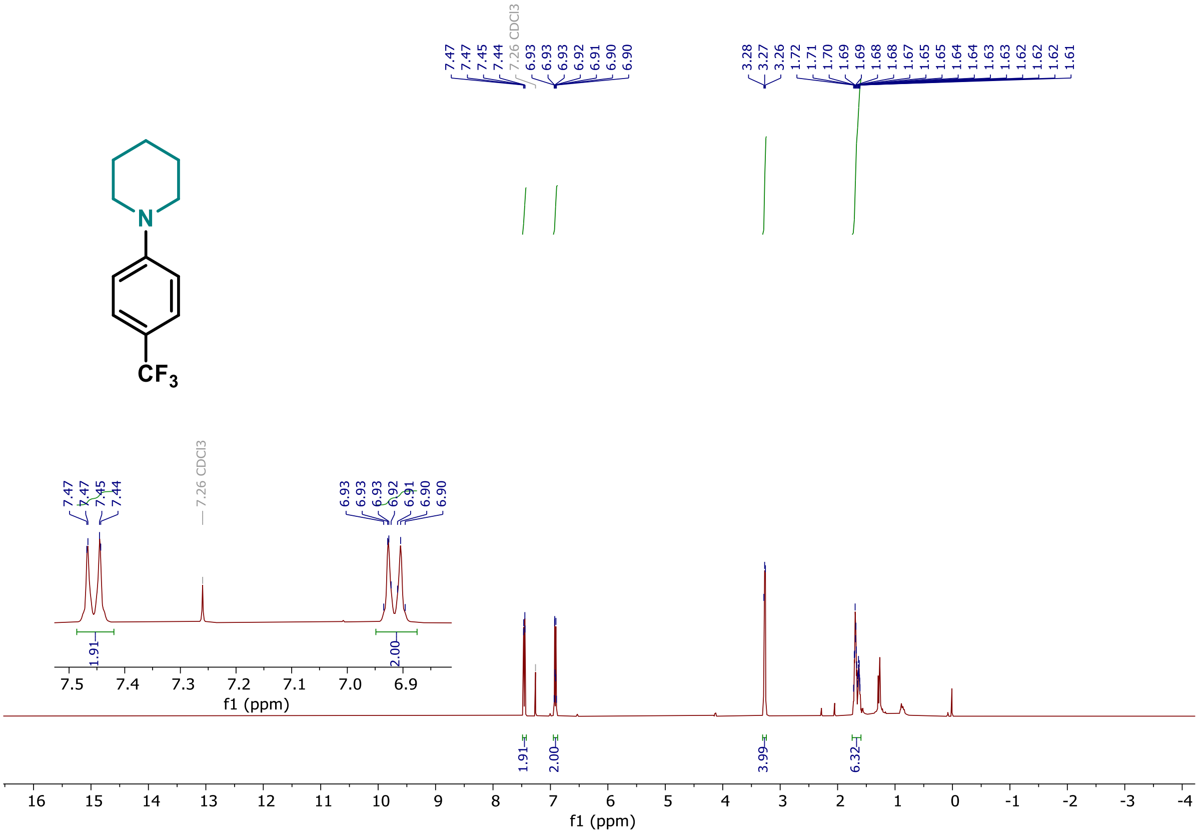

^{13}C NMR of 18 ($CDCl_3$, 101 MHz)

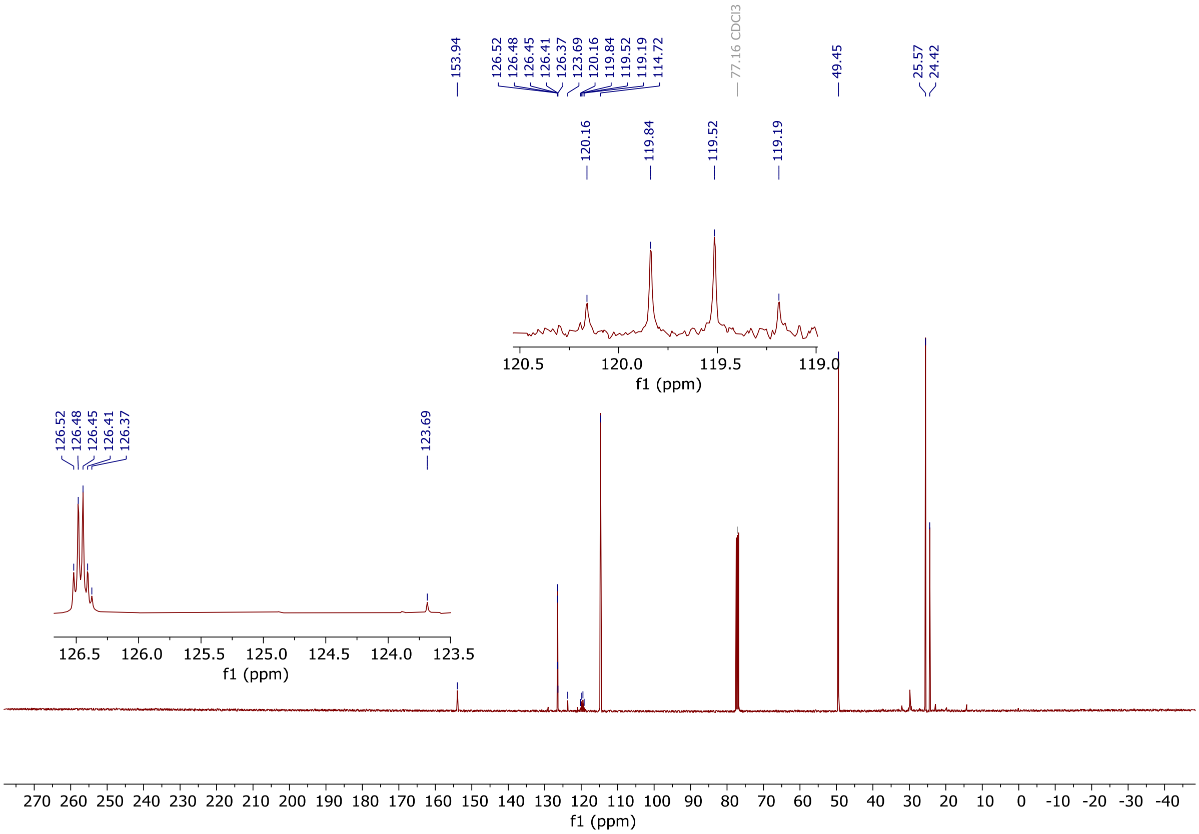

^{19}F NMR of 18 ($CDCl_3$, 282 MHz)

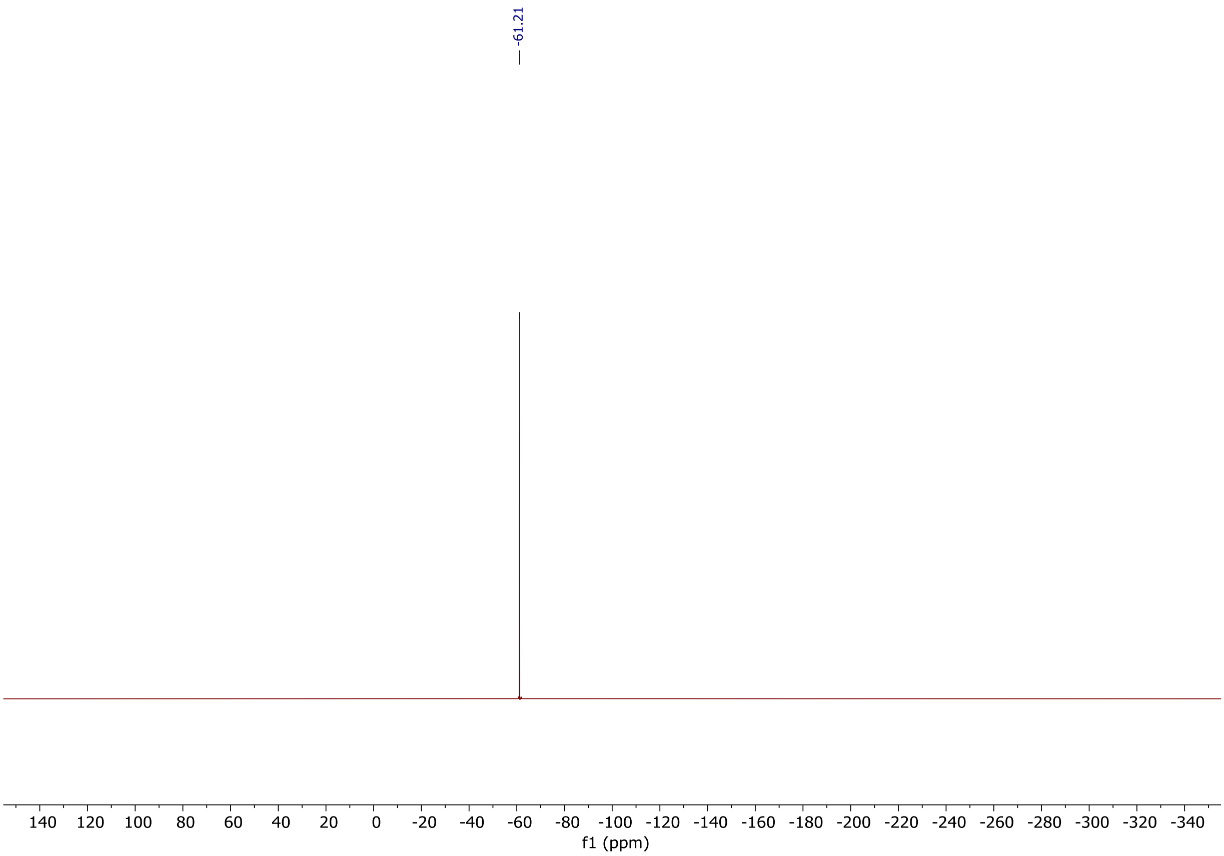

^{1}H NMR of 19 ($CDCl_3$, 300 MHz)

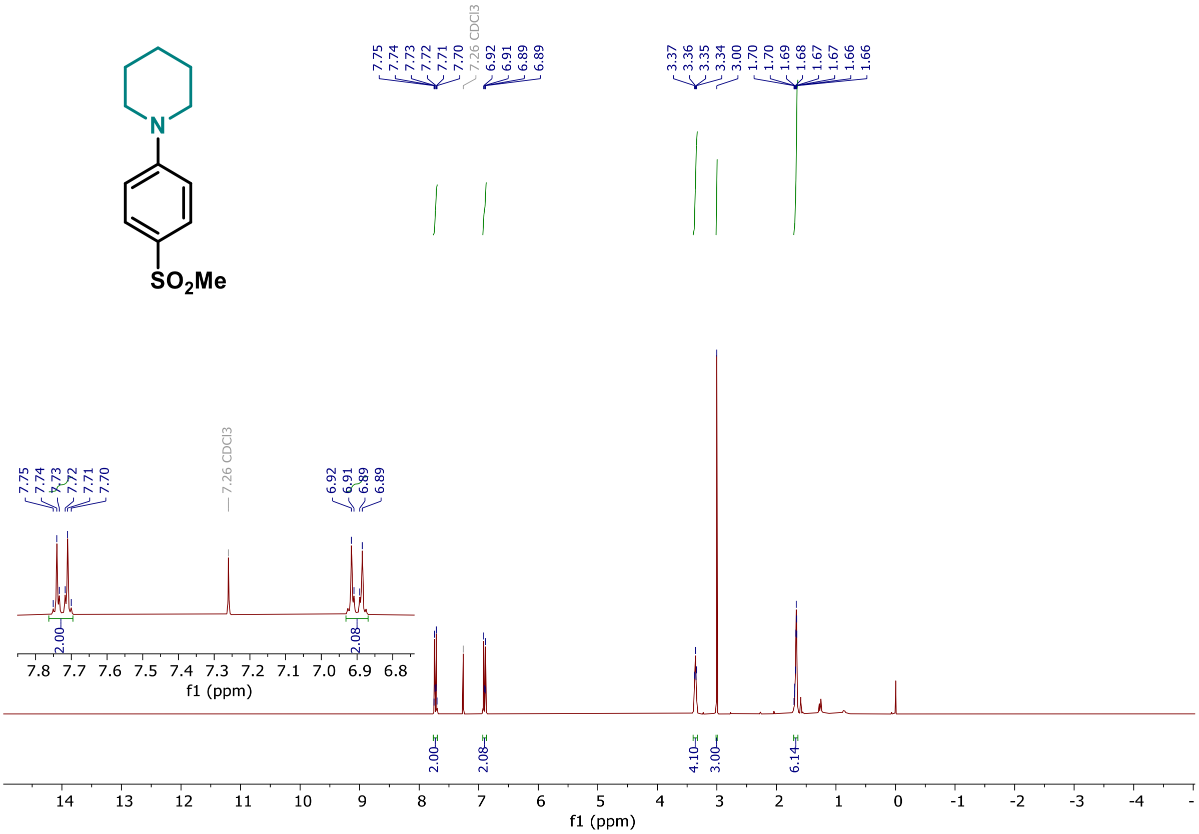

^{13}C NMR of 19 ($CDCl_3$, 75 MHz)

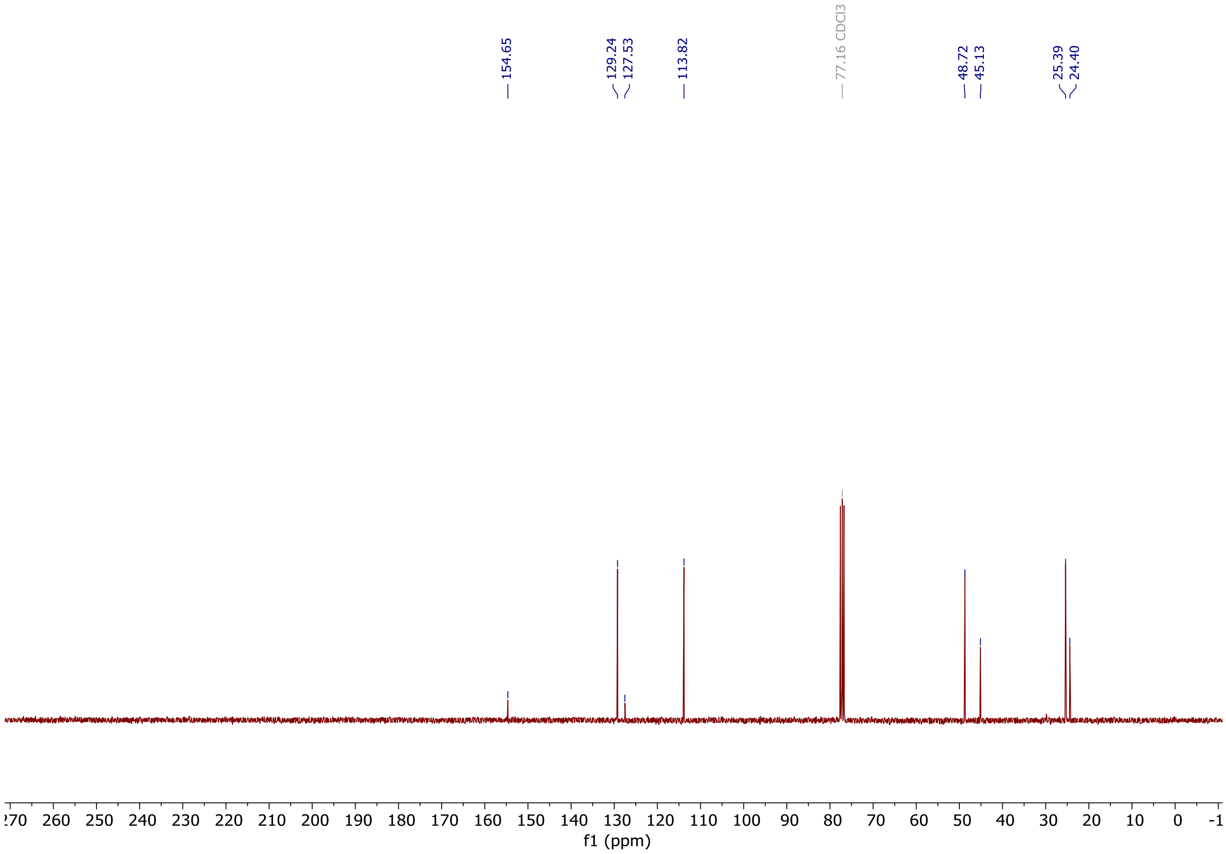

^{1}H NMR of 20 (DMSO-d_6, 400 MHz)

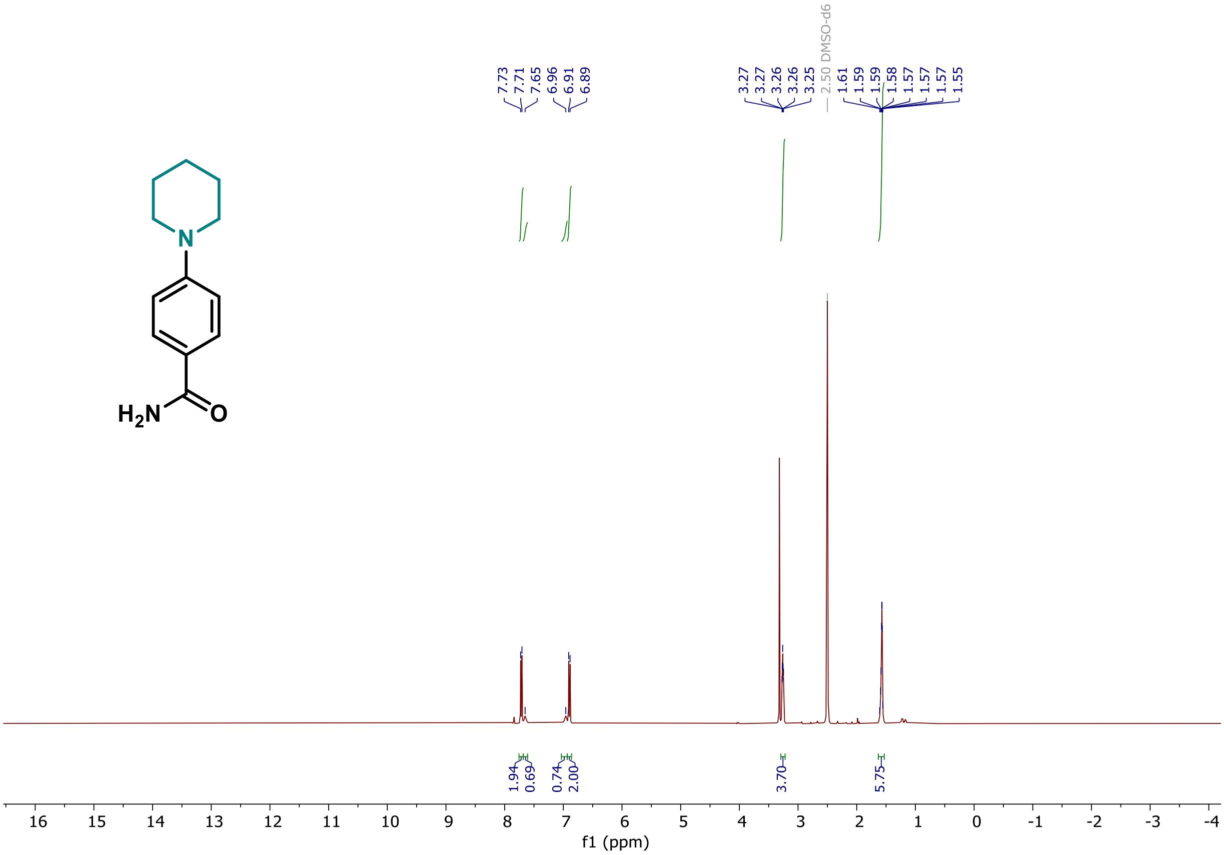

^{13}C NMR of 20 (DMSO-d_6, 101 MHz)

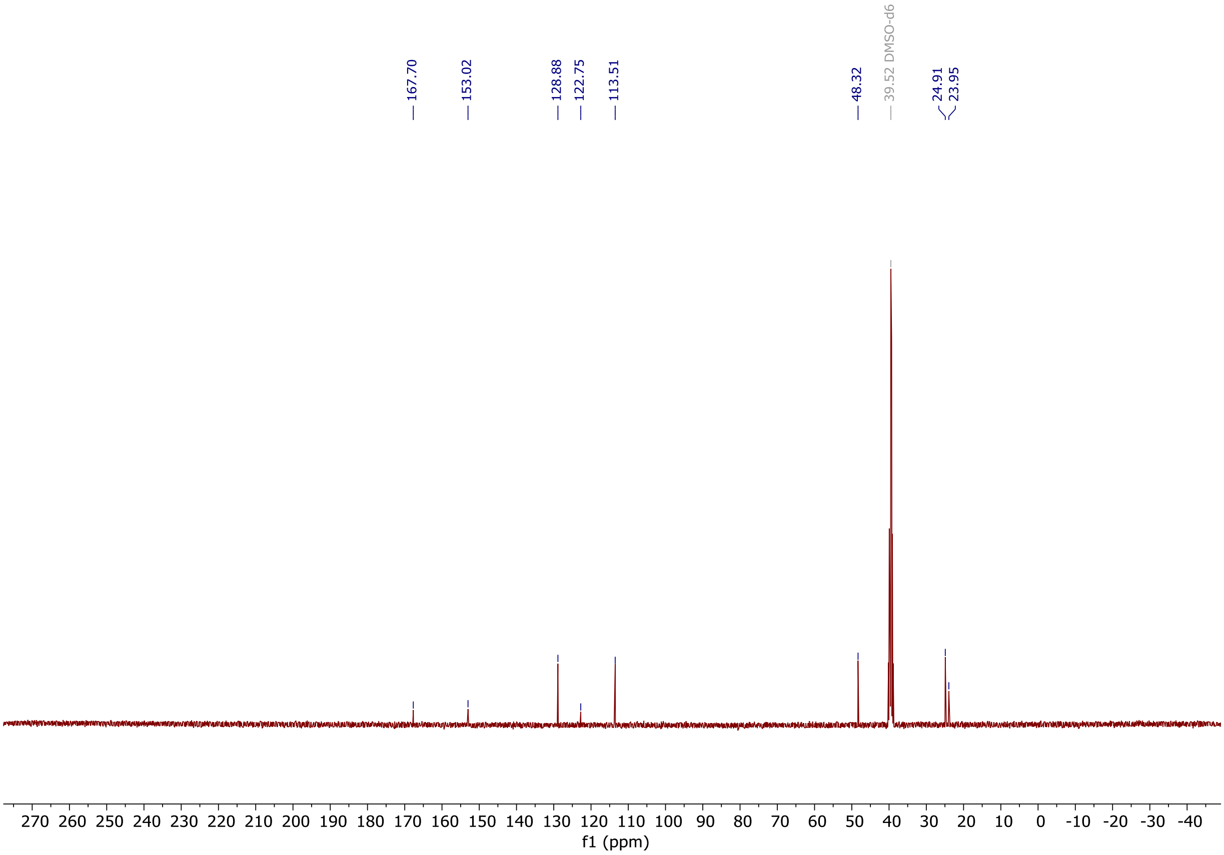

^{1}H NMR of 21 ($CDCl_3$, 400 MHz)

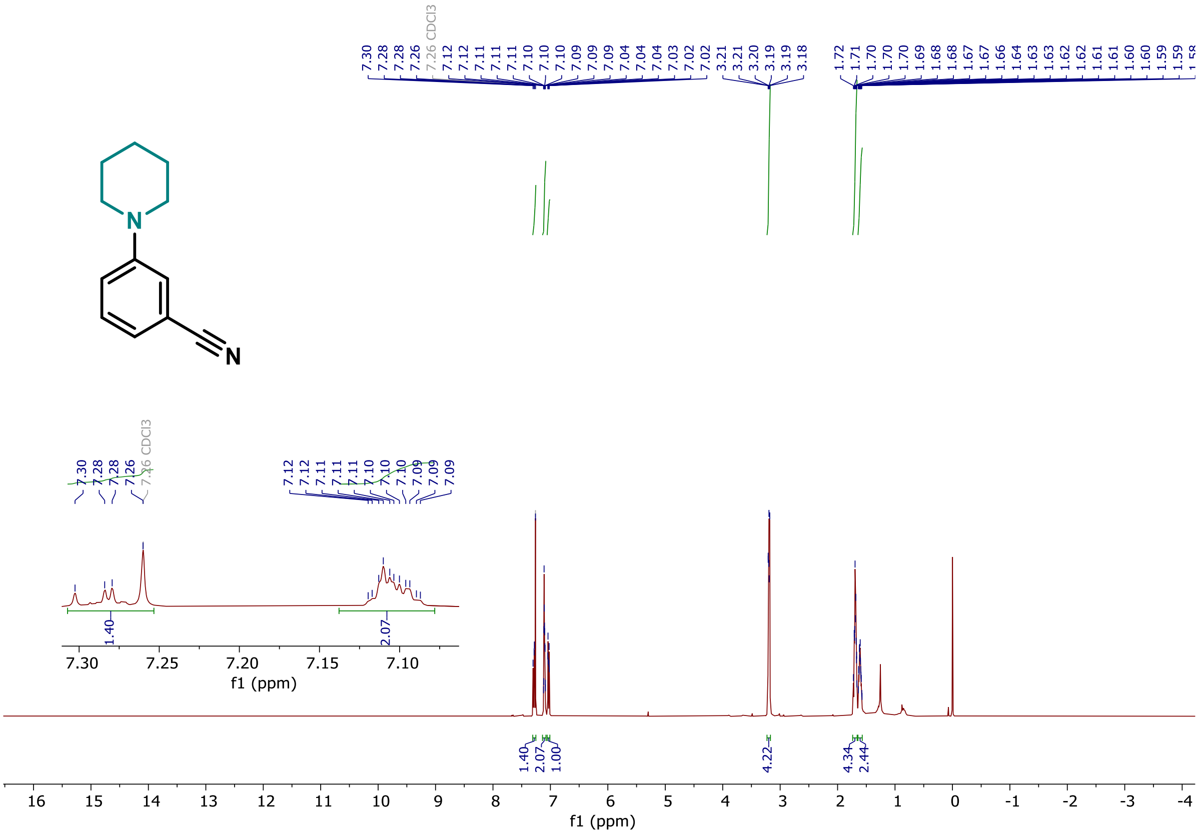

^{13}C NMR of 21 ($CDCl_3$, 101 MHz)

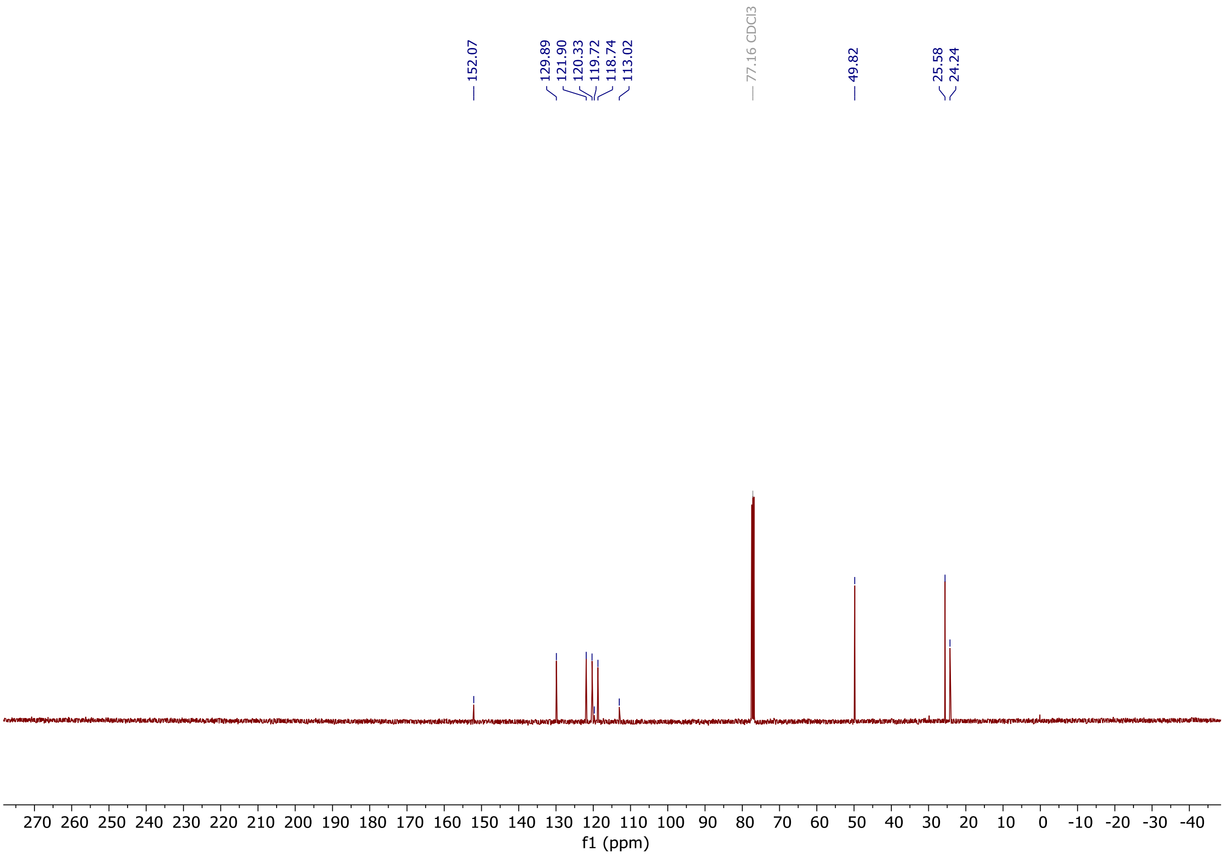

^{1}H NMR of 22 ($CDCl_3$, 300 MHz)

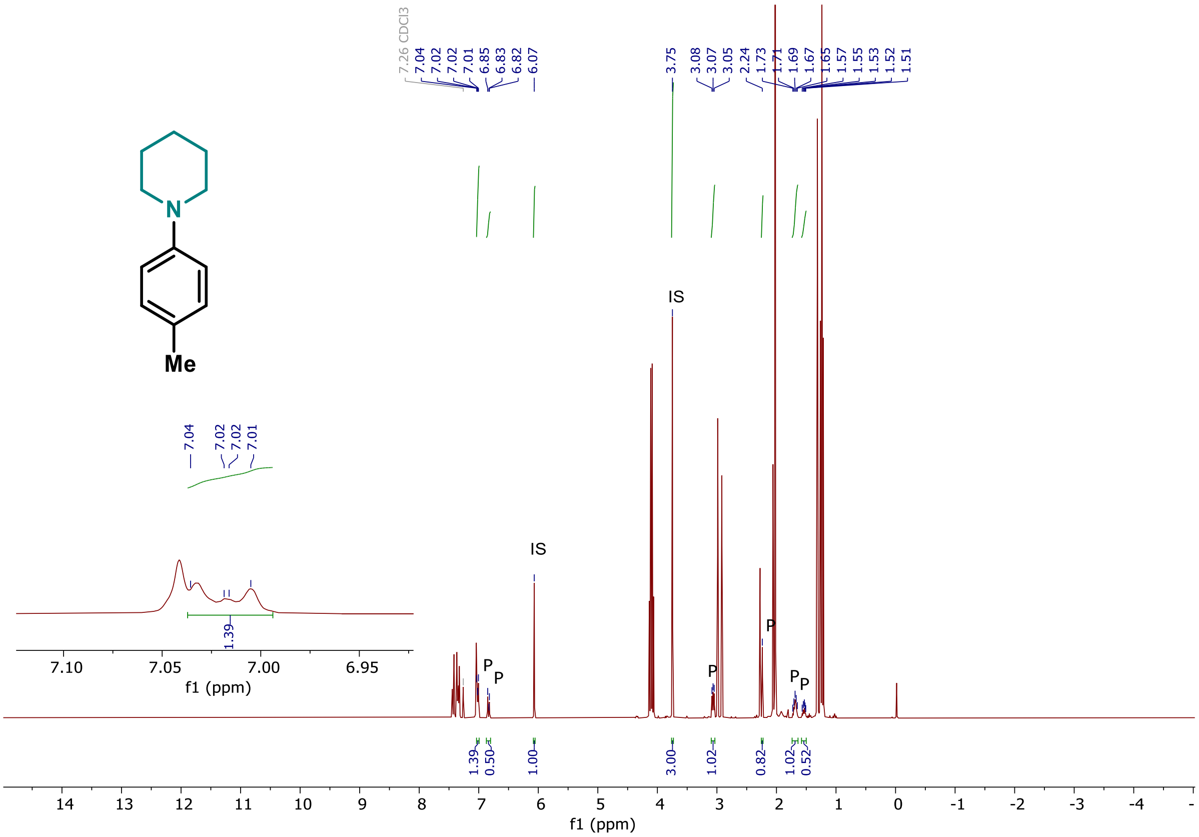

^{1}H NMR of 23 ($CDCl_3$, 300 MHz)

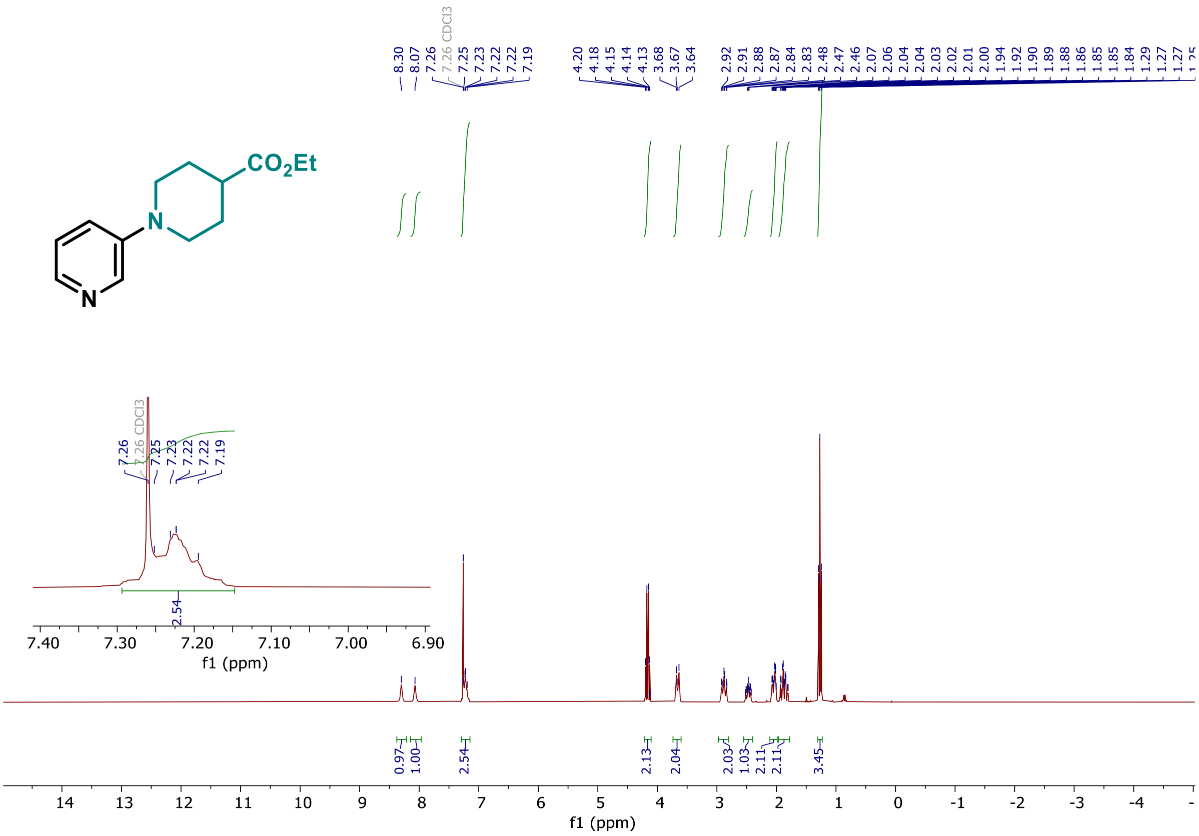

^{13}C NMR of 23 ($CDCl_3$, 75 MHz)

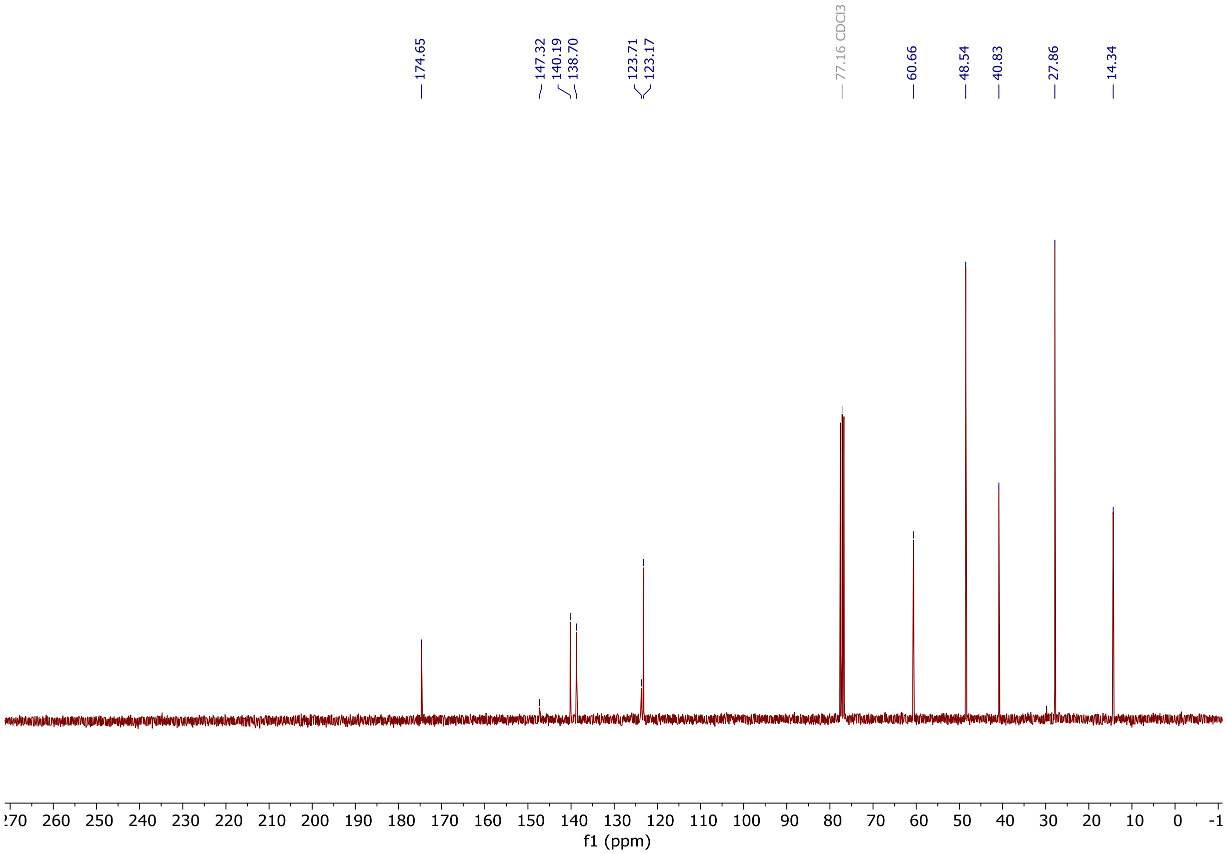

^{1}H NMR of 24 ($CDCl_3$, 400 MHz)

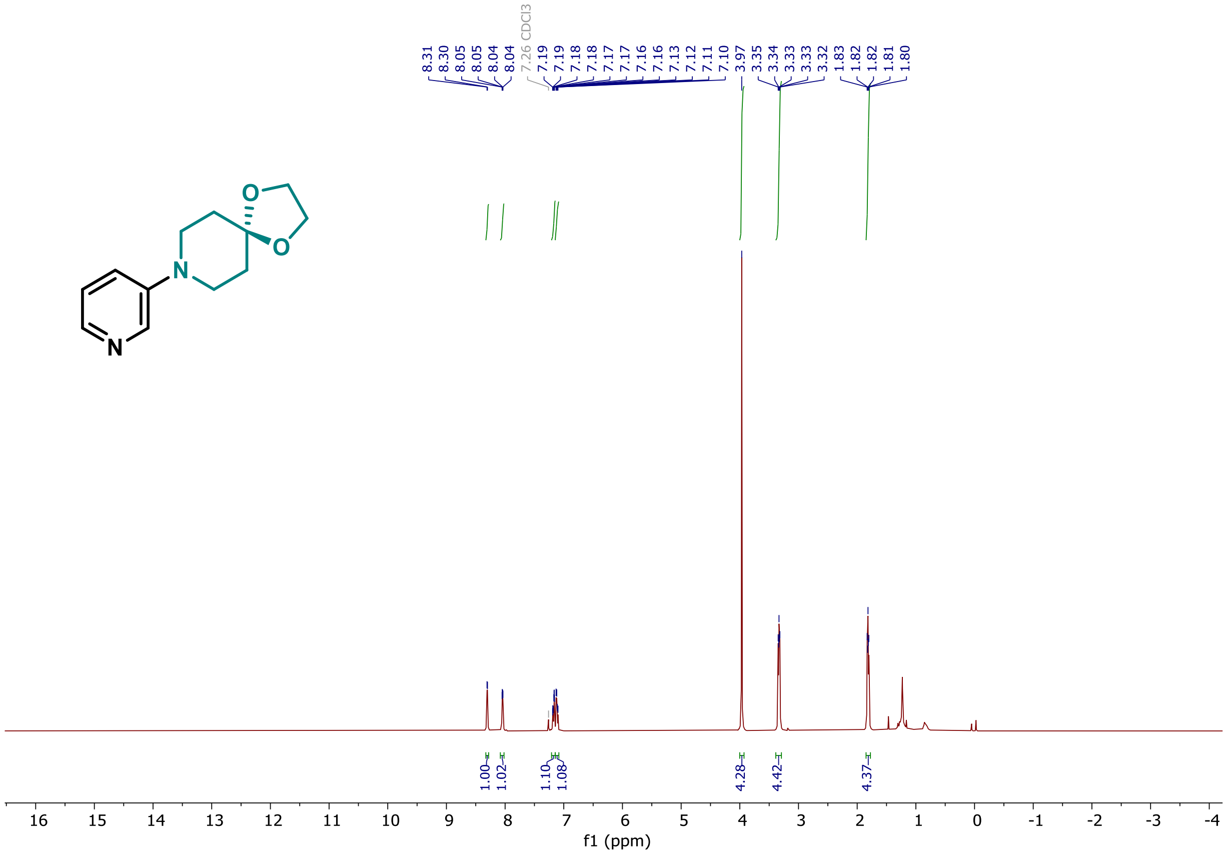

^{13}C NMR of 24 ($CDCl_3$, 101 MHz)

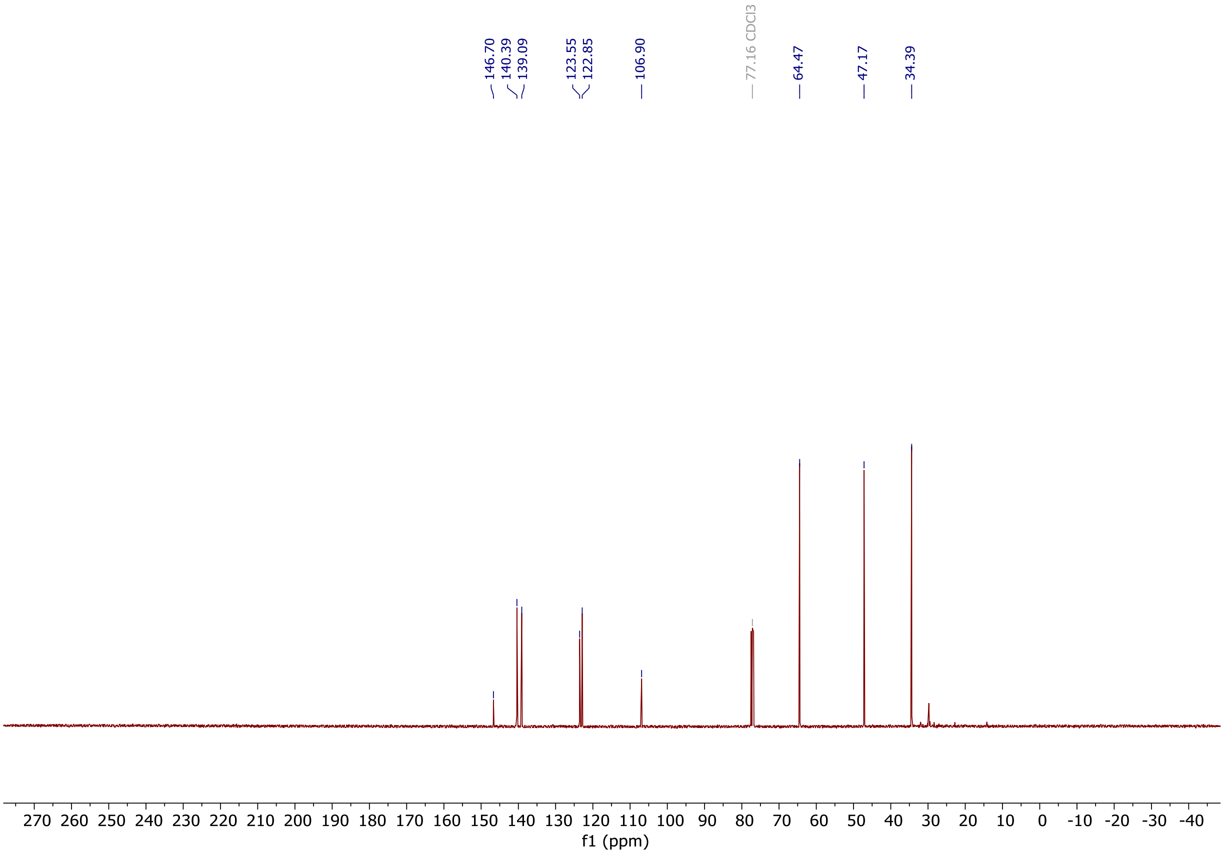

^{1}H NMR of 25 ($CDCl_3$, 400 MHz)

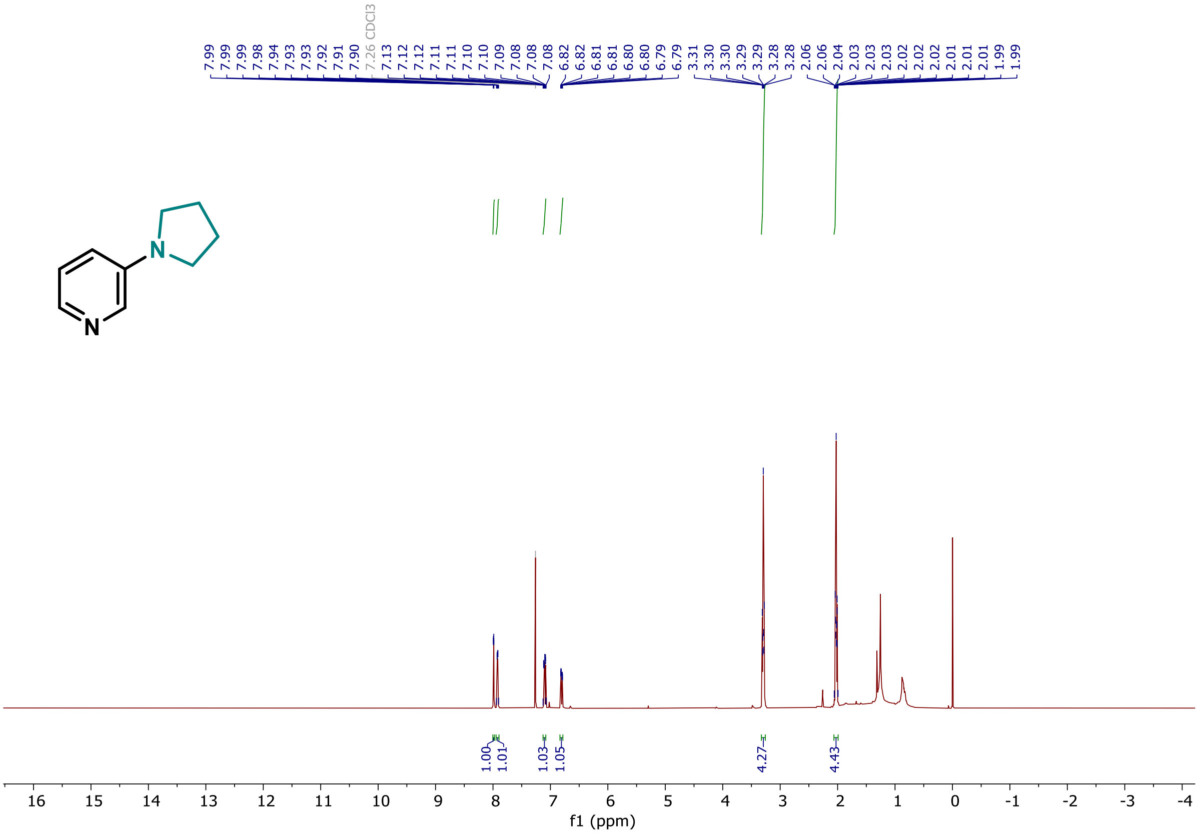

^{13}C NMR of 25 ($CDCl_3$, 101 MHz)

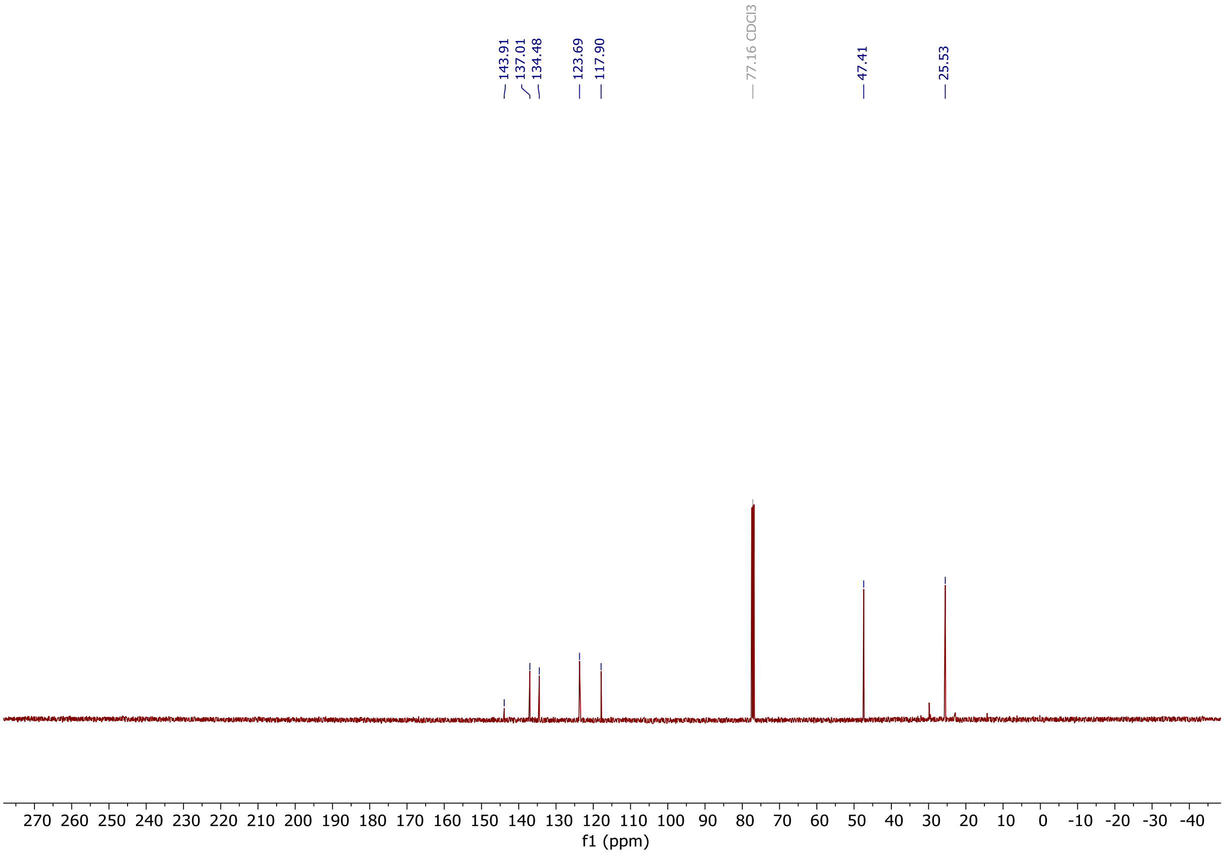

^{1}H NMR of 26 ($CDCl_3$, 400 MHz)

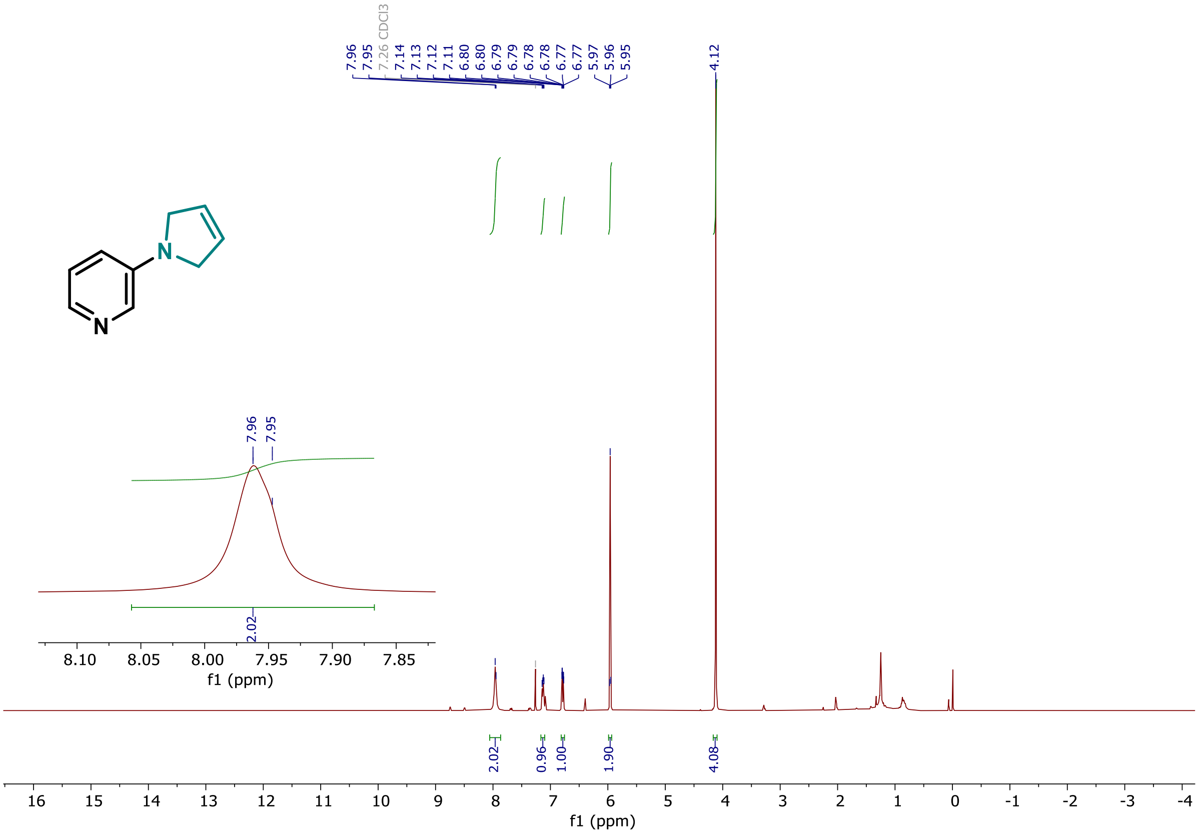

^{13}C NMR of 26 ($CDCl_3$, 101 MHz)

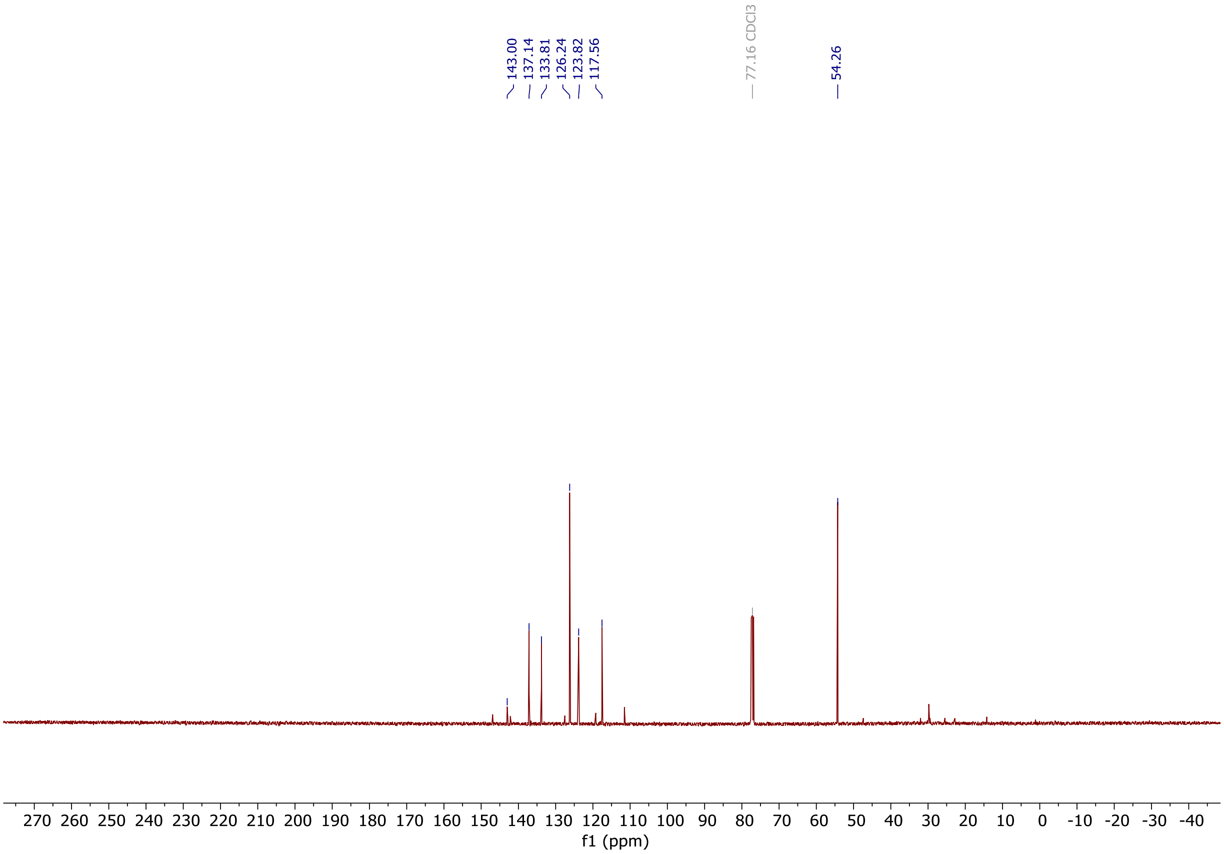

COSY of 26 ($CDCl_3$, 400 MHz)

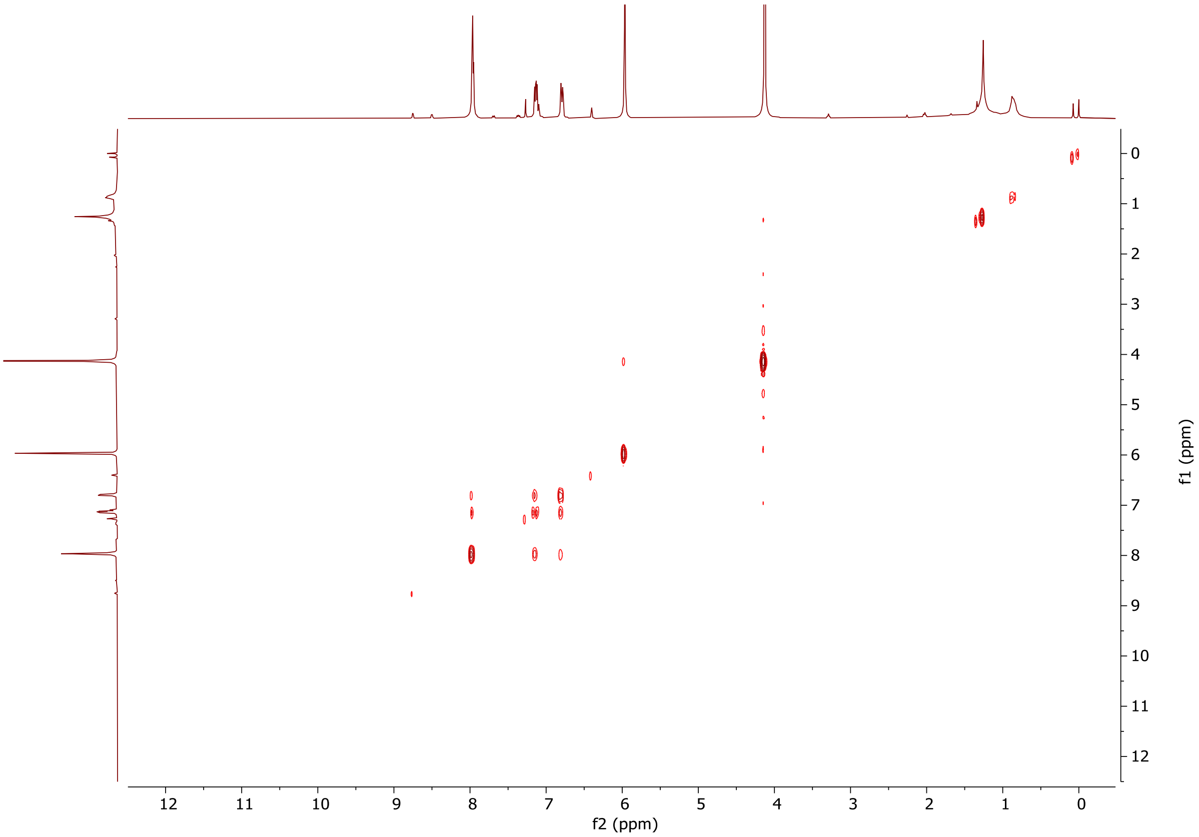

HSQC of 26 ($CDCl_3$, 400 MHz)

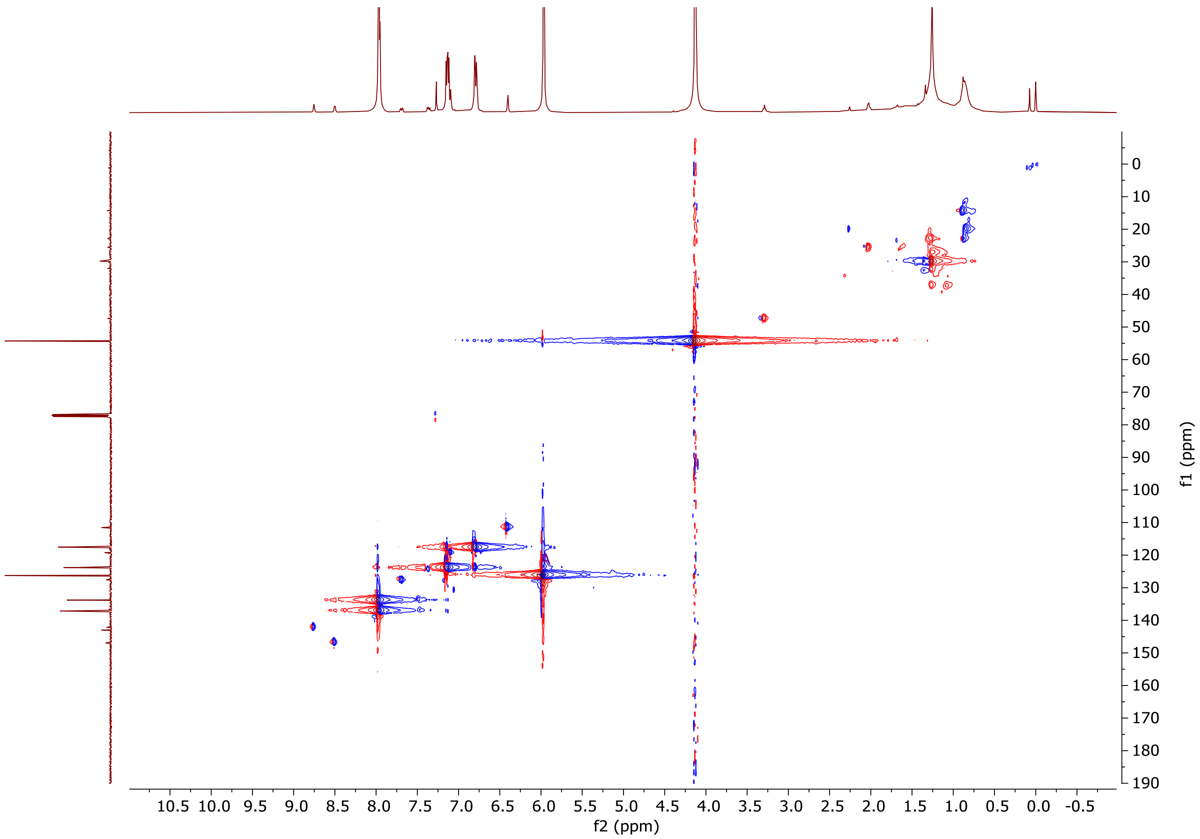

HMBC of 26 ($CDCl_3$, 400 MHz)

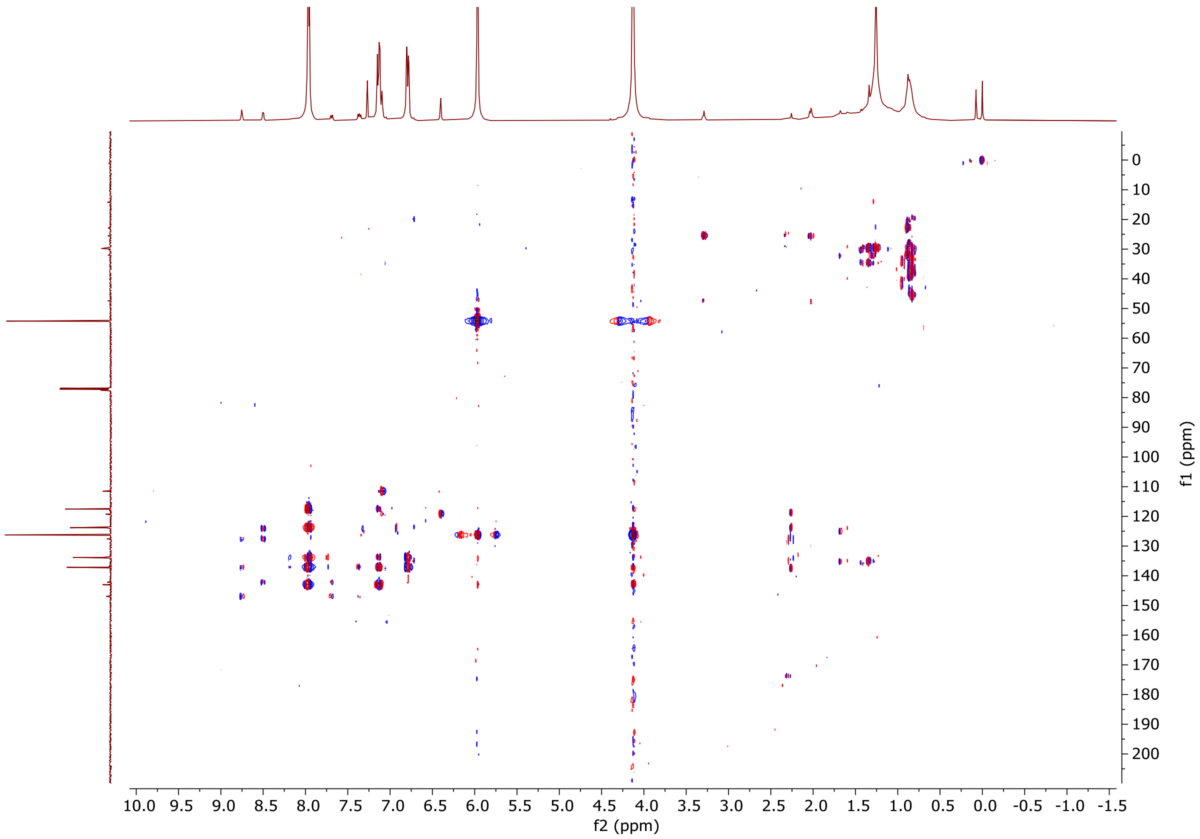

^{1}H NMR of 27 ($CDCl_3$, 300 MHz)

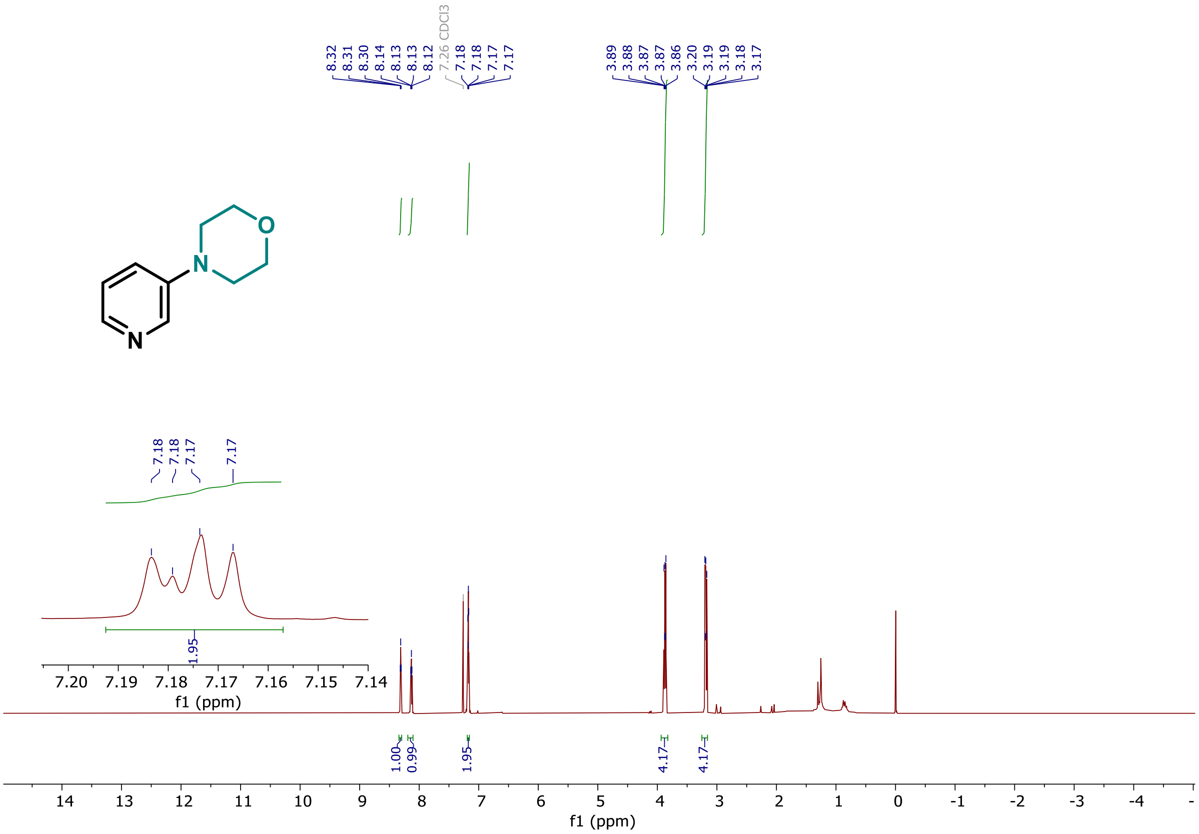

^{13}C NMR of 27 ($CDCl_3$, 101 MHz)

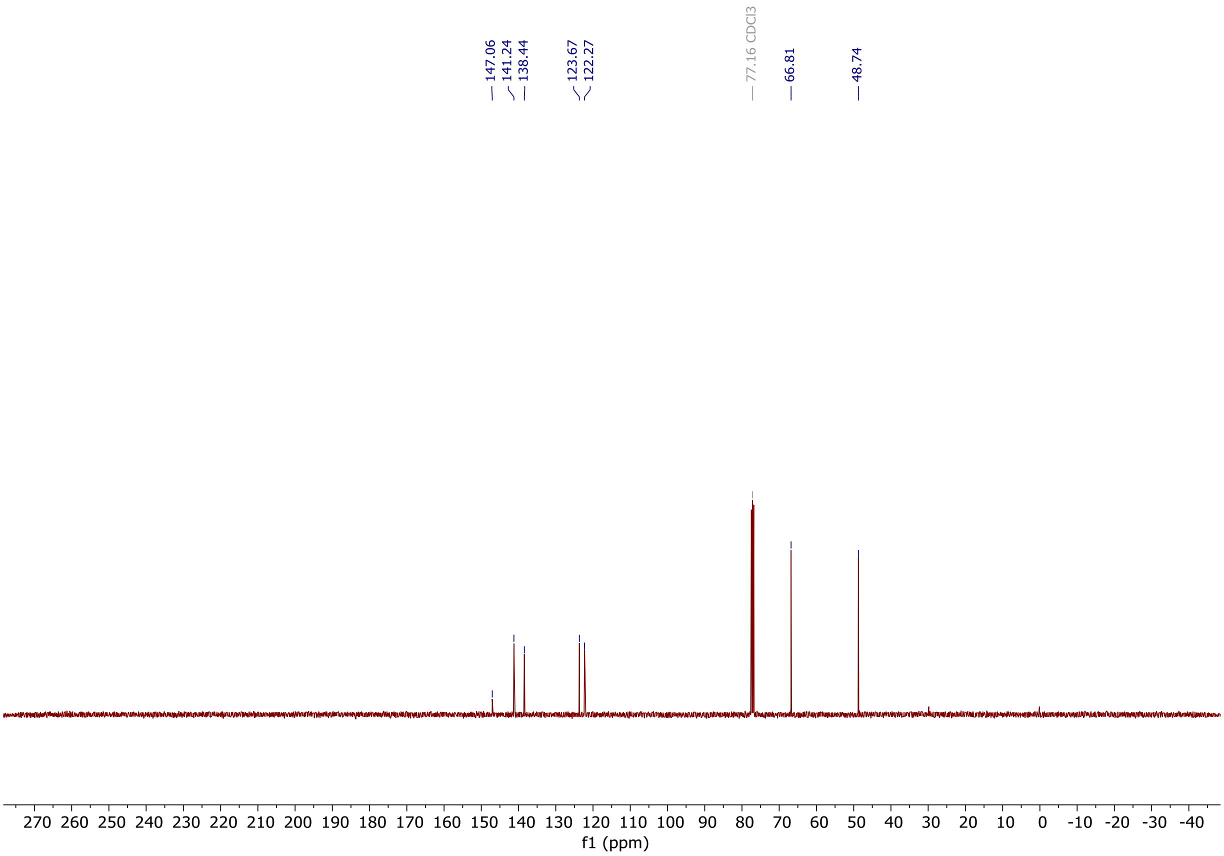

^{1}H NMR of 28 ($CDCl_3$, 400 MHz)

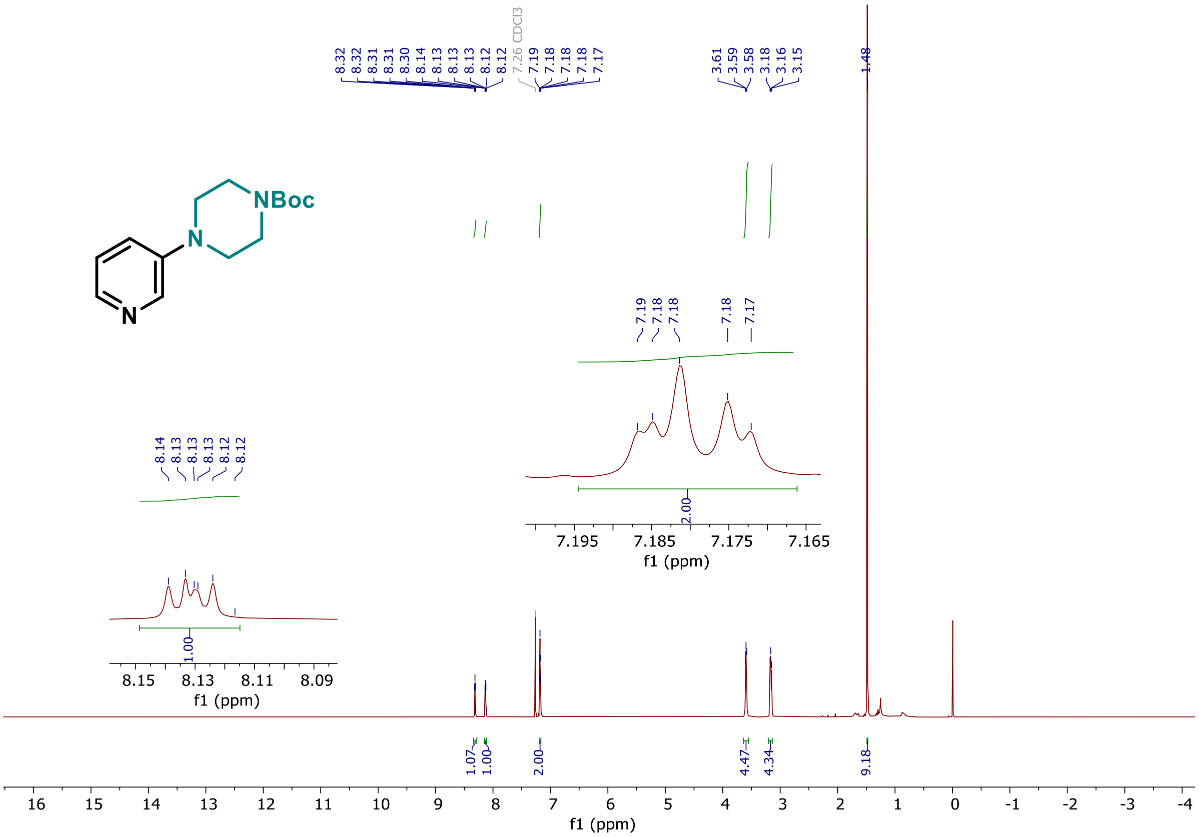

^{13}C NMR of 28 ($CDCl_3$, 101 MHz)

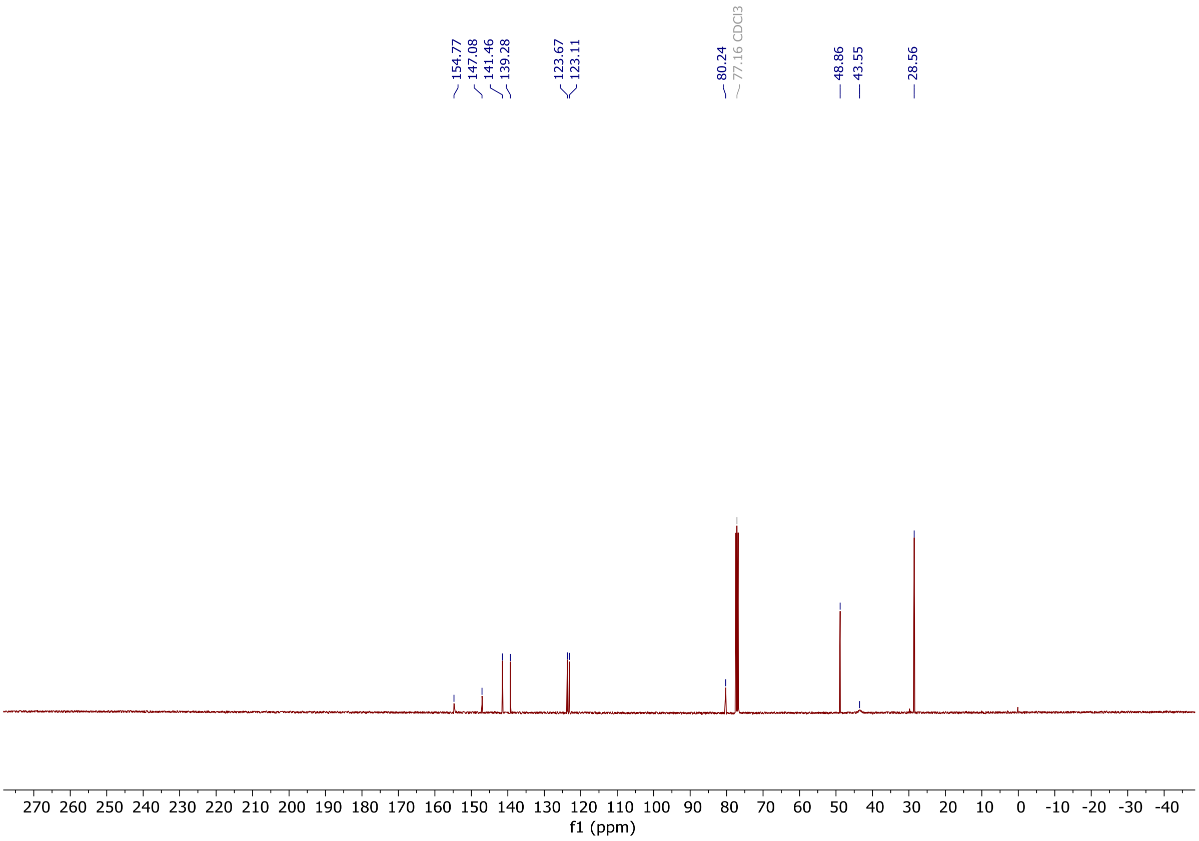

^{1}H NMR of 29 ($CDCl_3$, 400 MHz)

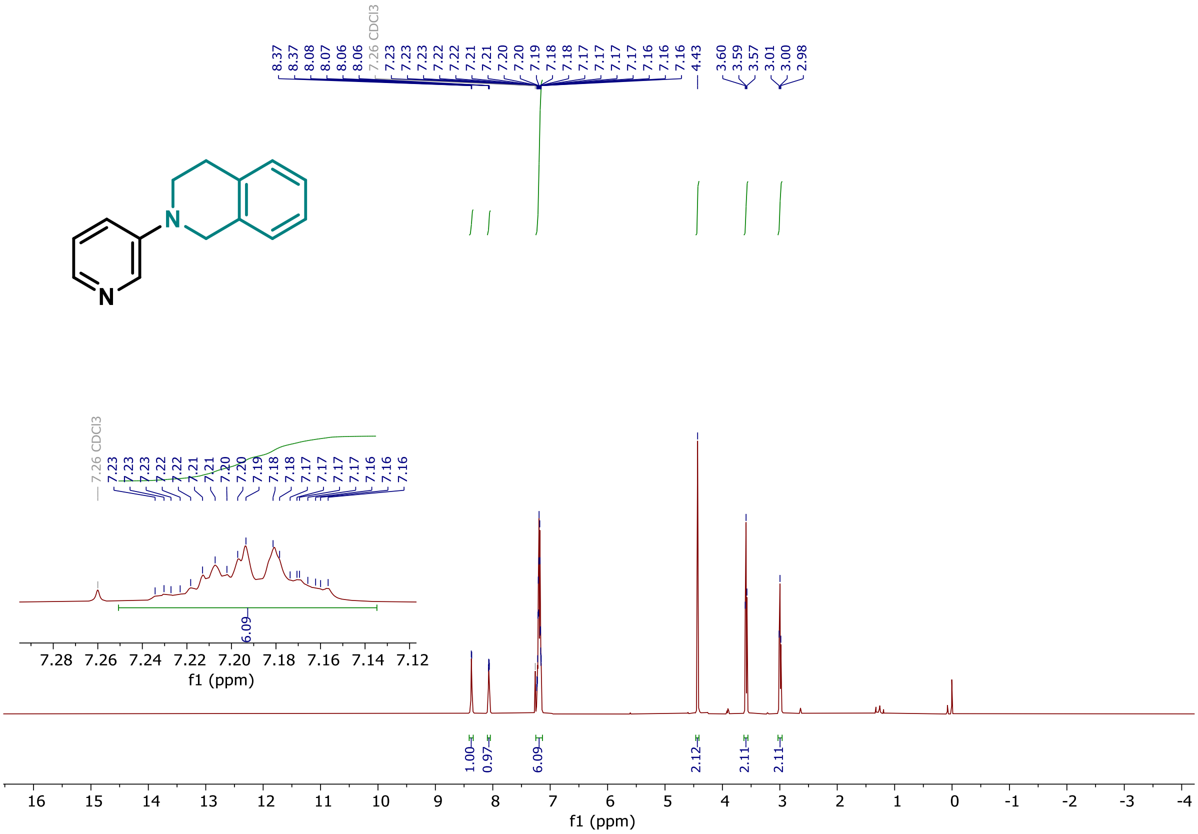

^{13}C NMR of 29 ($CDCl_3$, 101 MHz)

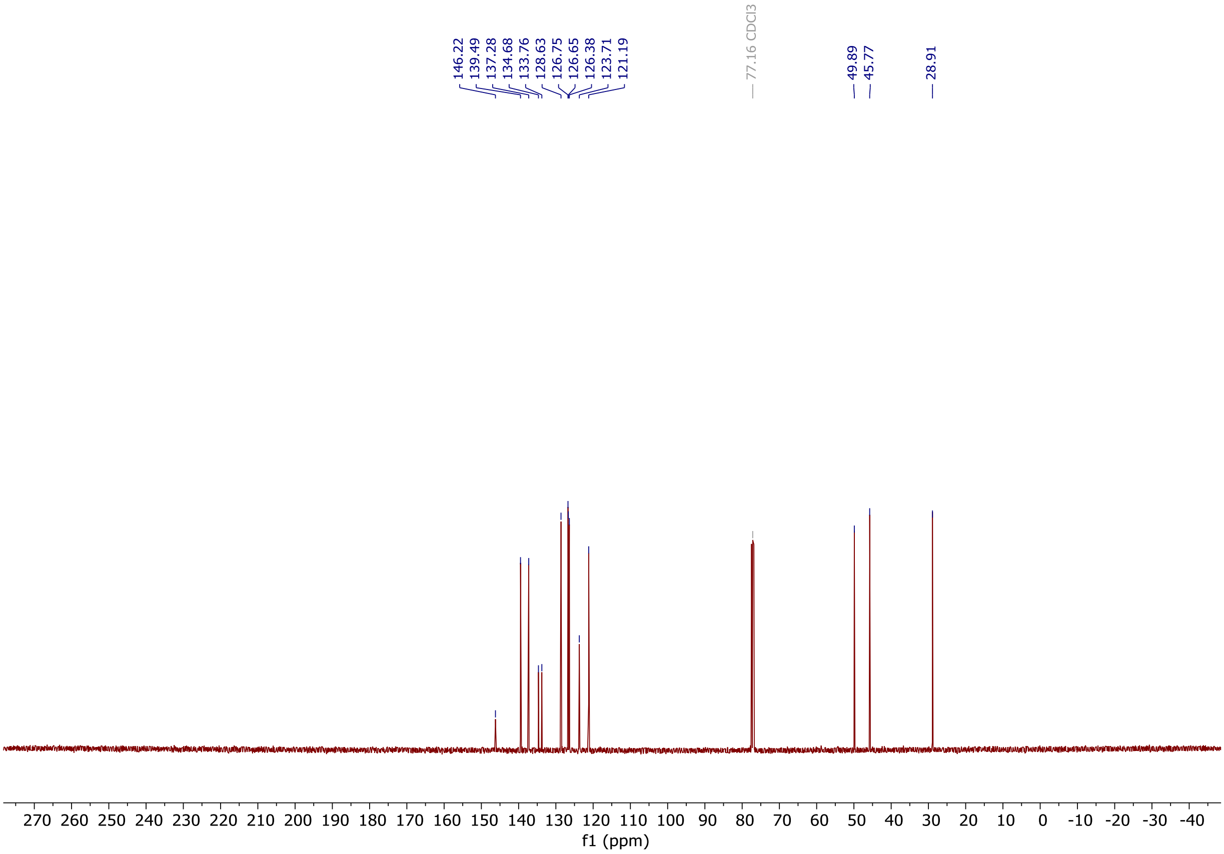

^{1}H NMR of 30 ($CDCl_3$, 400 MHz)

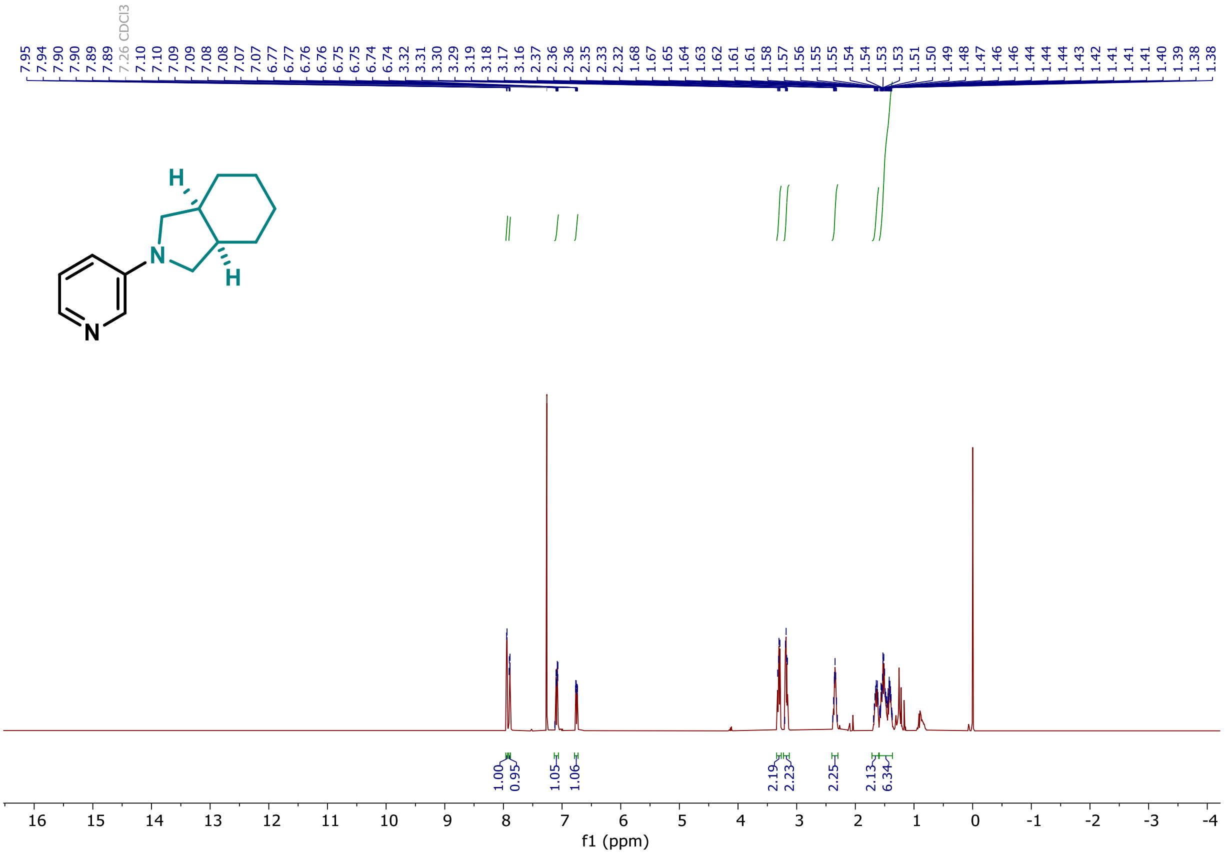

^{13}C NMR of 30 ($CDCl_3$, 101 MHz)

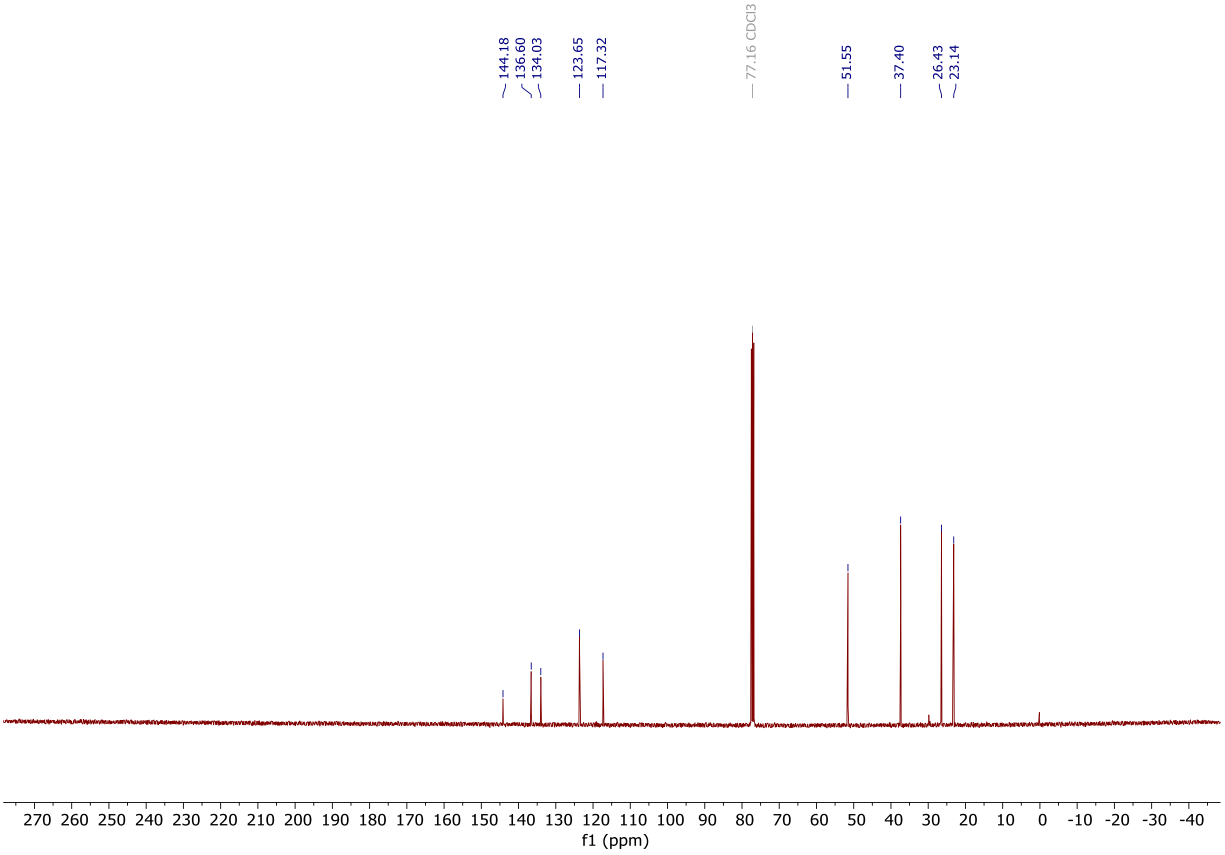

^{1}H NMR of 31 ($CDCl_3$, 400 MHz)

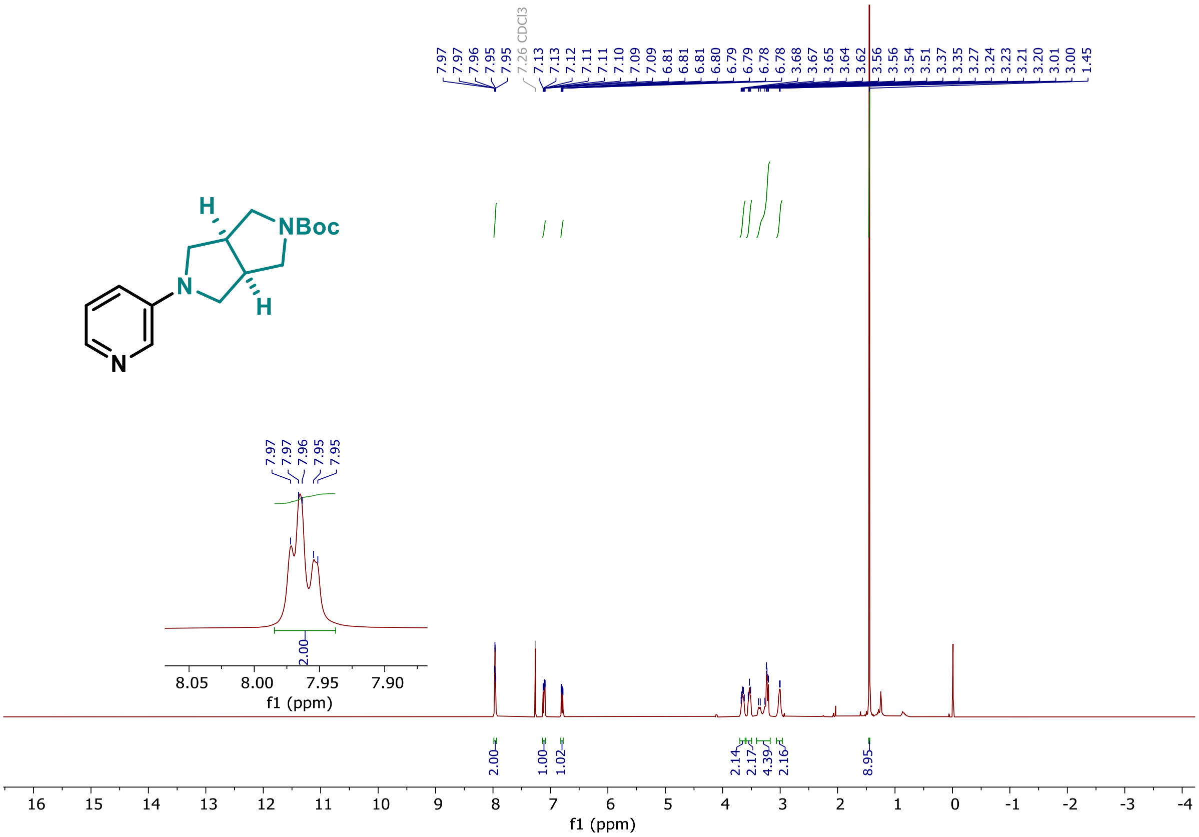

^{13}C NMR of 31 ($CDCl_3$, 101 MHz)

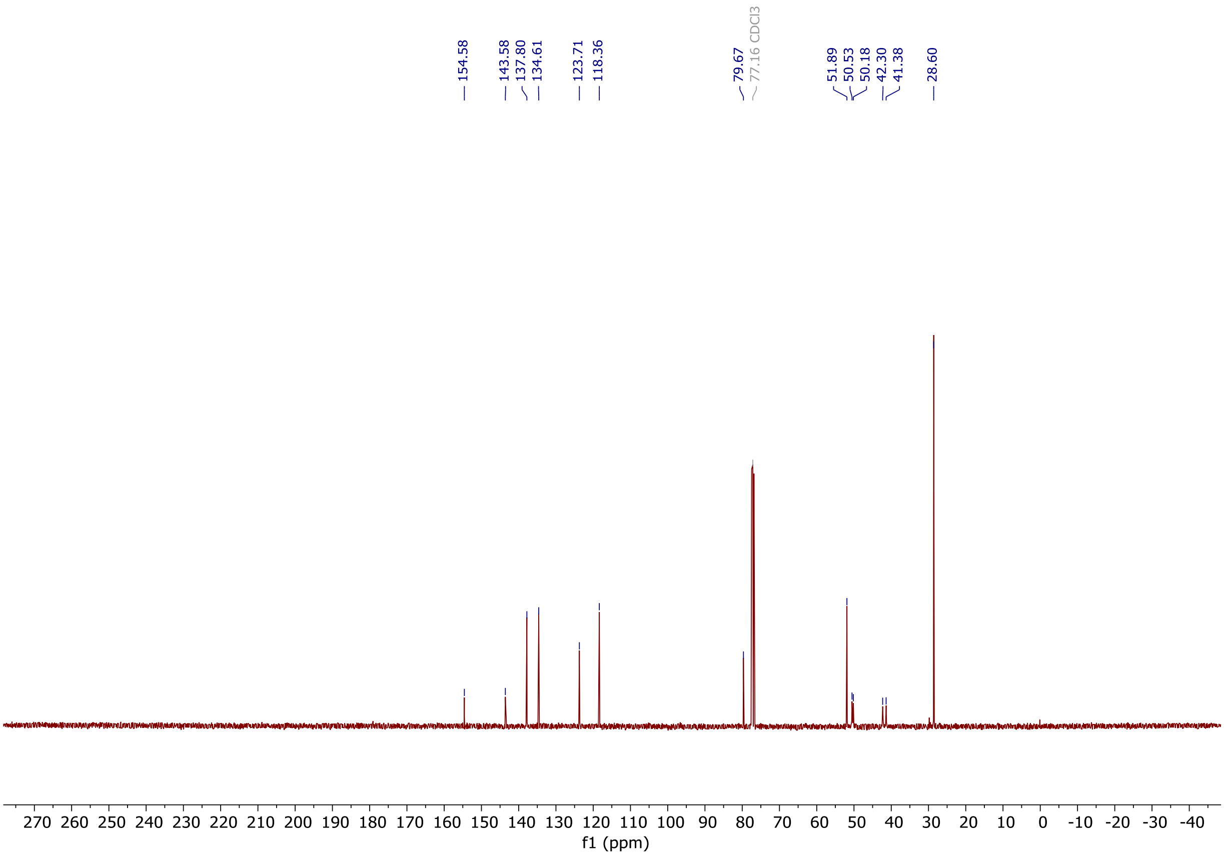

^{1}H NMR of 32 ($CDCl_3$, 300 MHz)

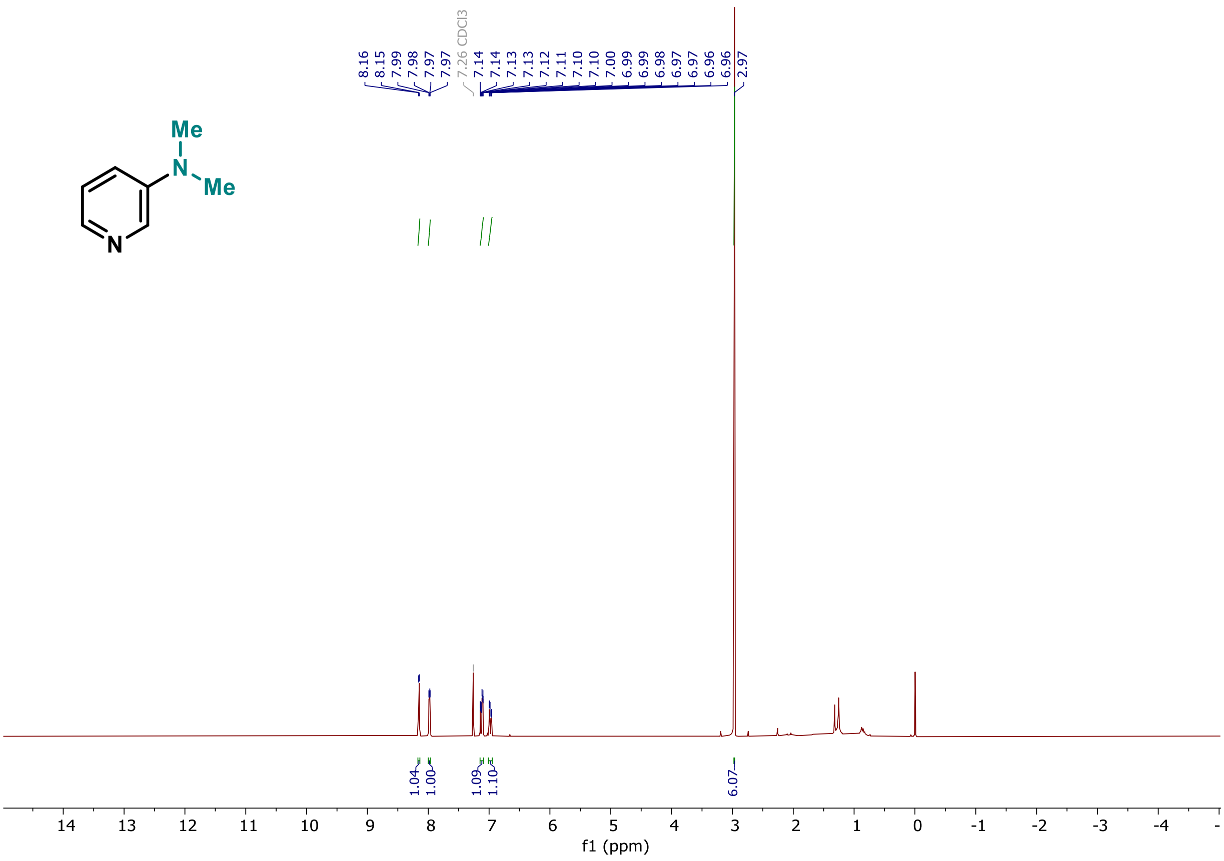

^{13}C NMR of 32 ($CDCl_3$, 75 MHz)

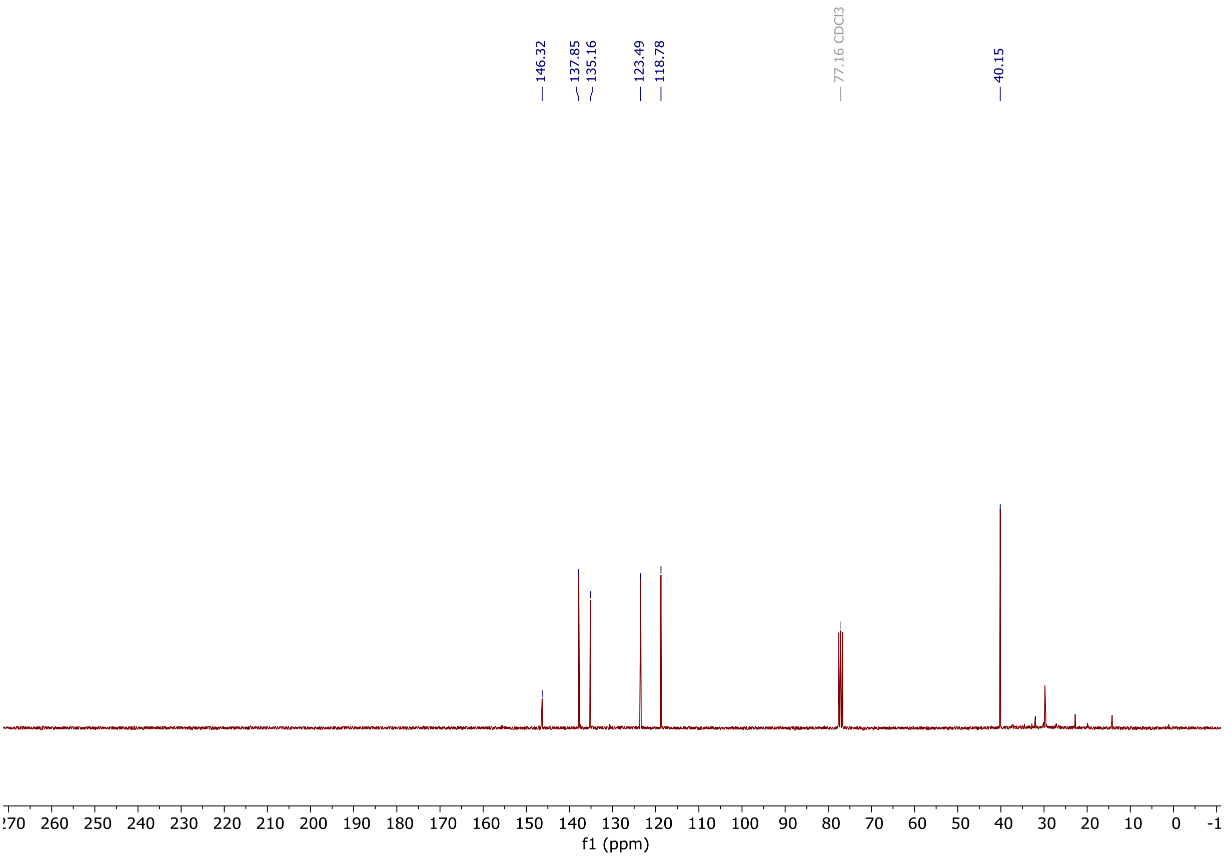

^{1}H NMR of 33 ($CDCl_3$, 400 MHz)

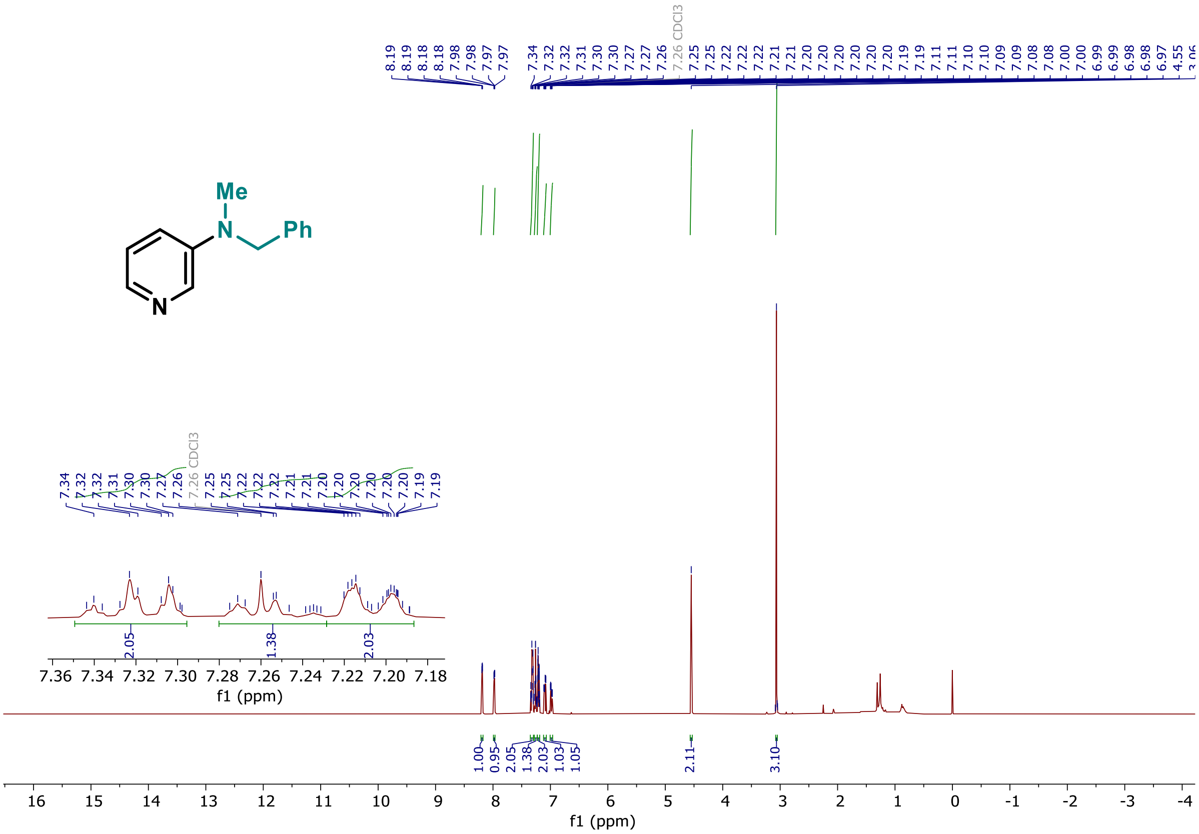

^{13}C NMR of 33 ($CDCl_3$, 101 MHz)

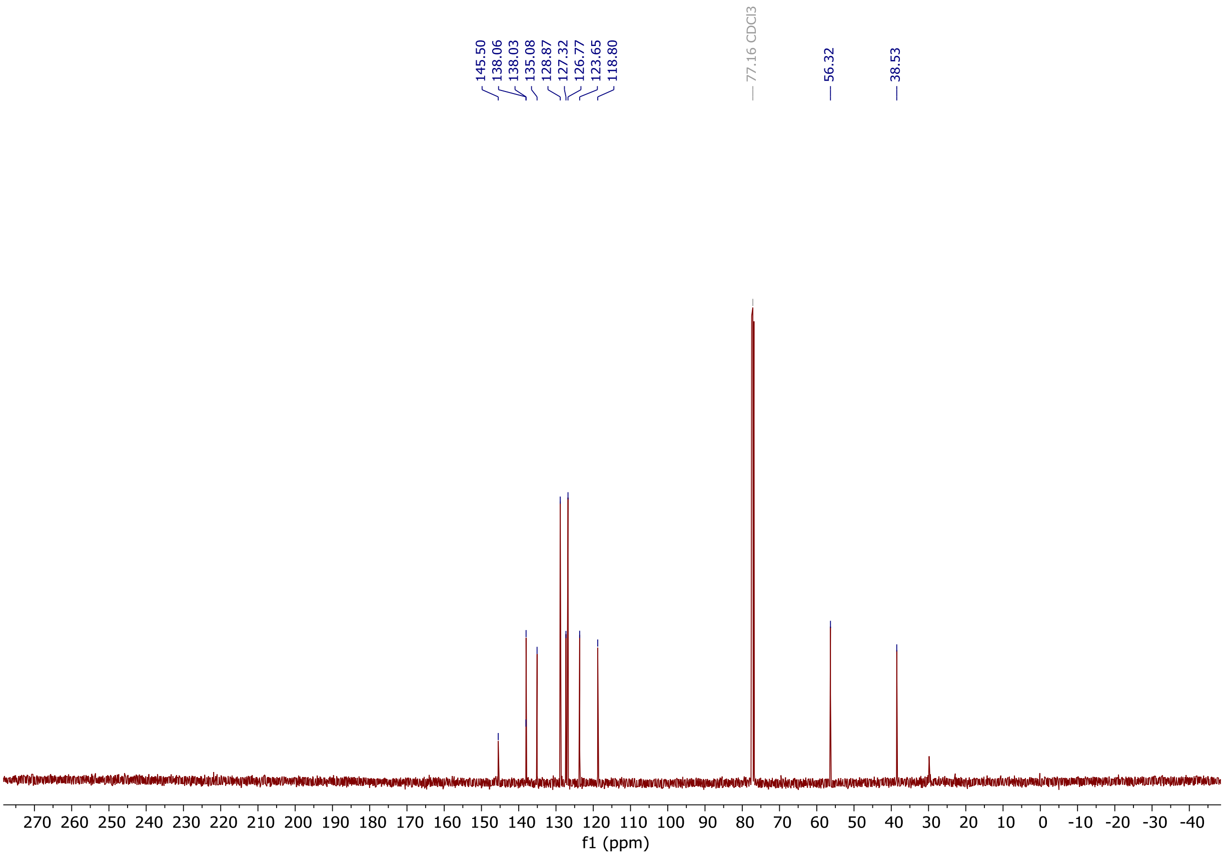

^{1}H NMR of 34 ($CDCl_3$, 400 MHz)

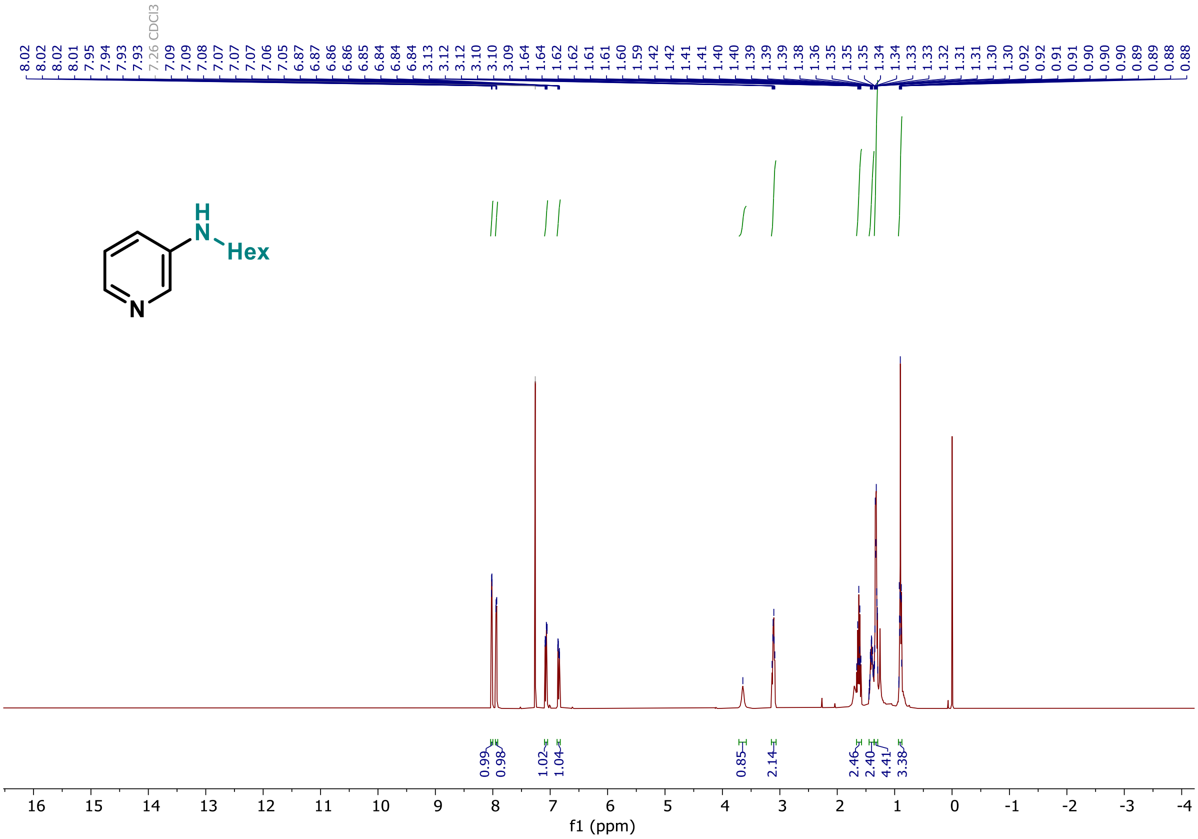

^{13}C NMR of 34 ($CDCl_3$, 101 MHz)

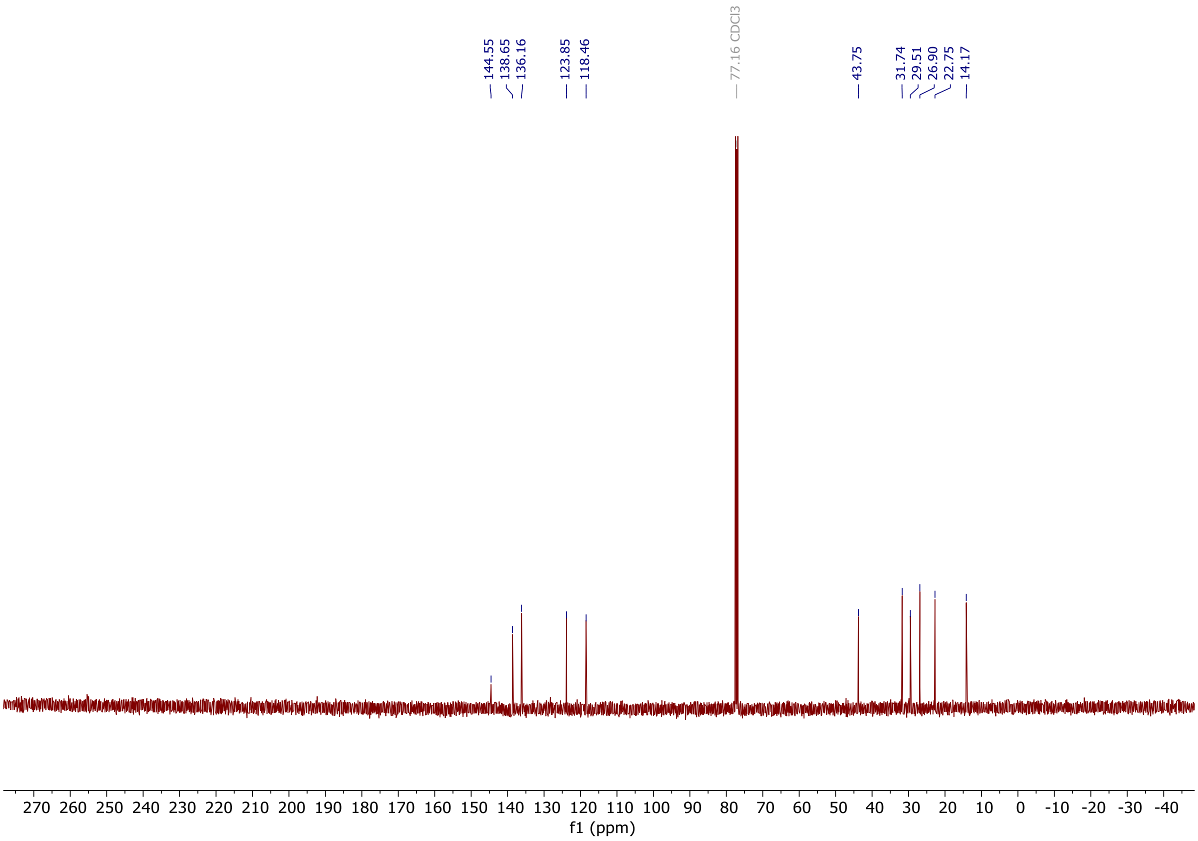

^{1}H NMR of 35 ($CDCl_3$, 300 MHz)

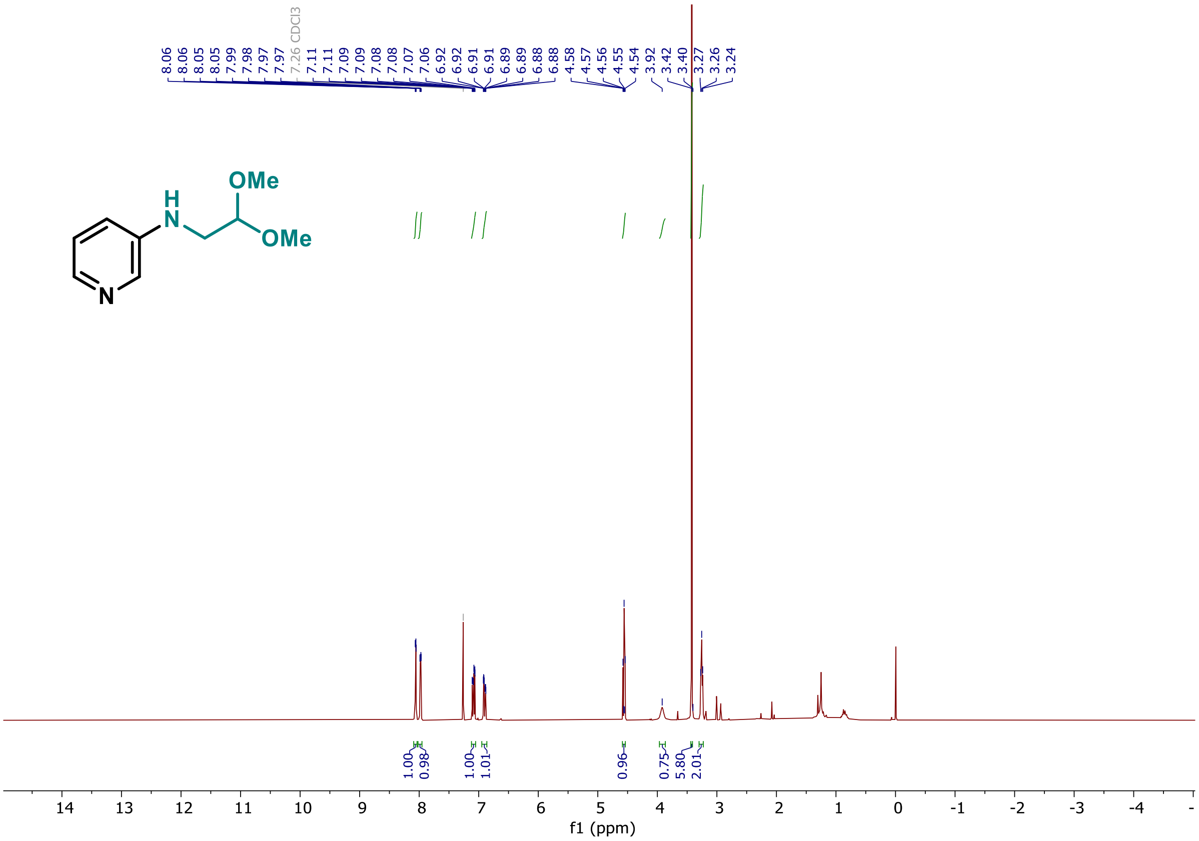

^{13}C NMR of 35 ($CDCl_3$, 101 MHz)

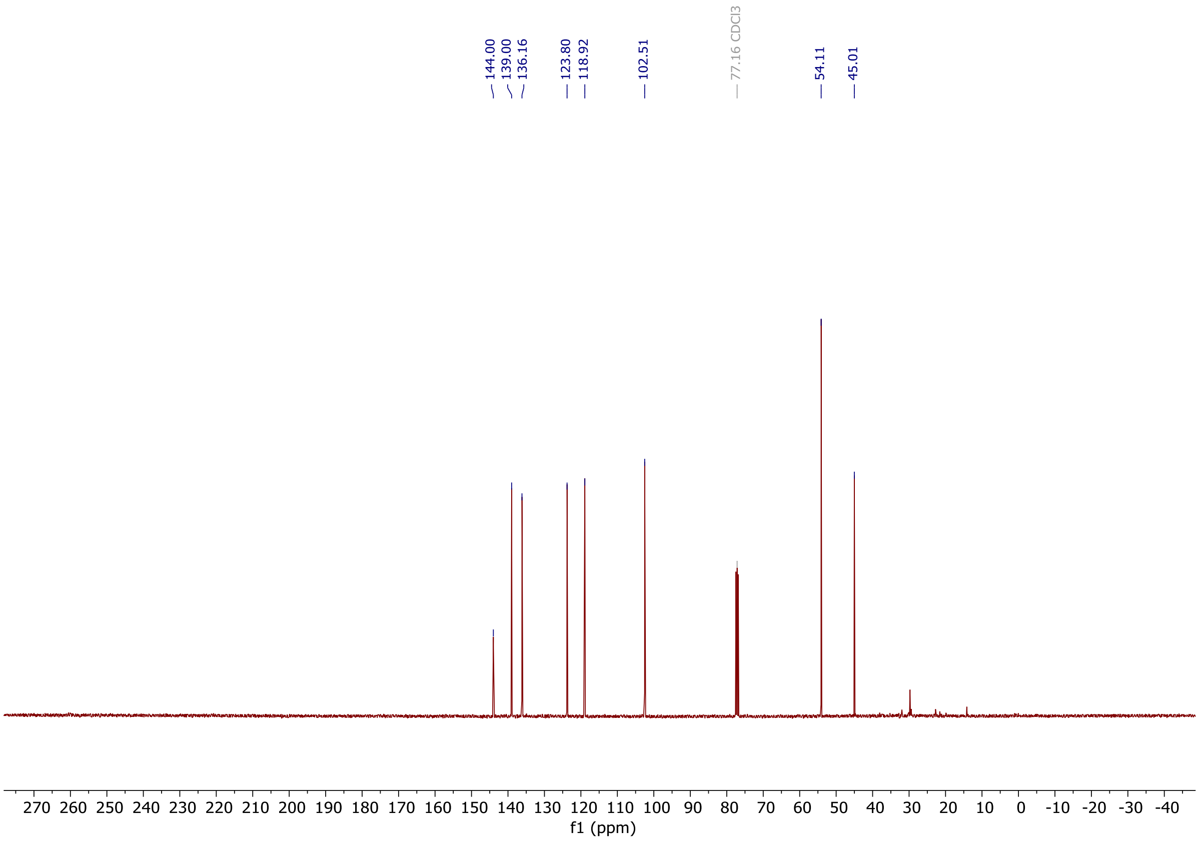

^{1}H NMR of 36 ($CDCl_3$, 400 MHz)

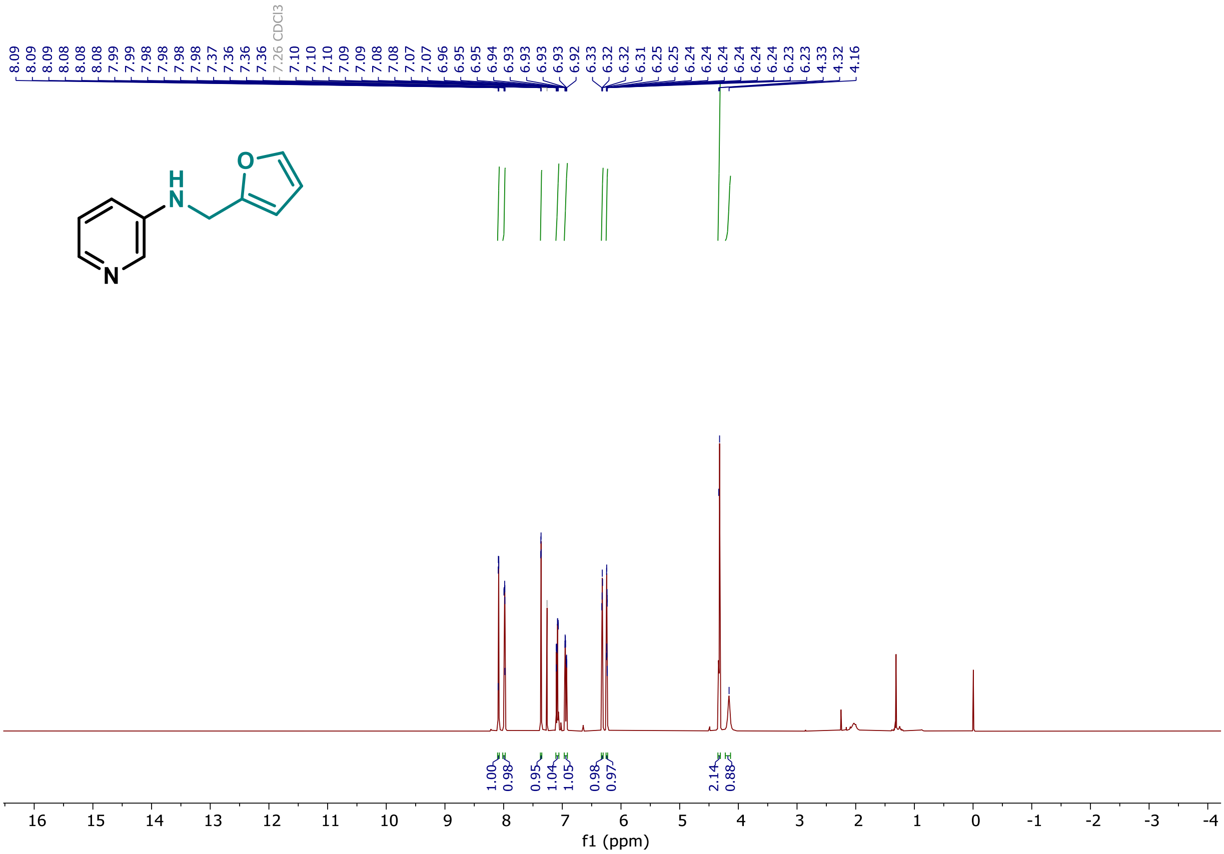

^{13}C NMR of 36 ($CDCl_3$, 101 MHz)

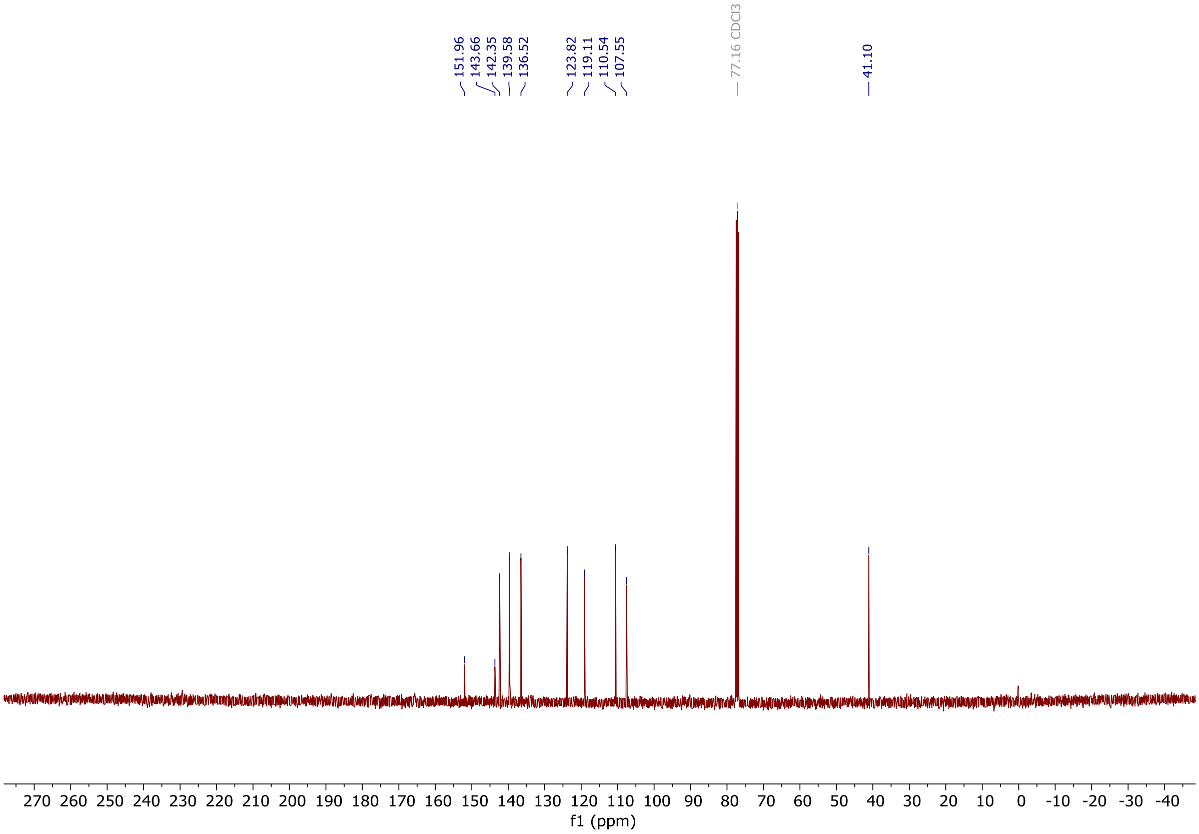

^{1}H NMR of 37 ($CDCl_3$, 400 MHz)

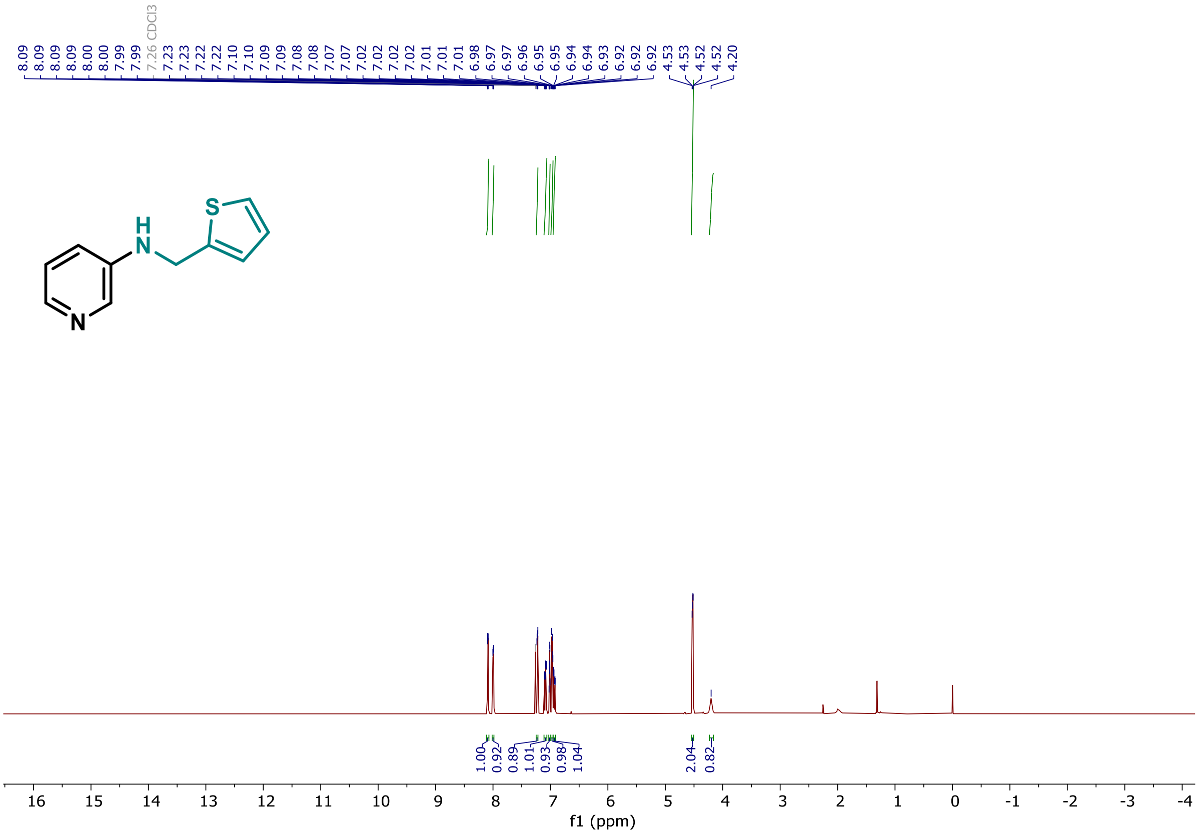

^{13}C NMR of 37 ($CDCl_3$, 101 MHz)

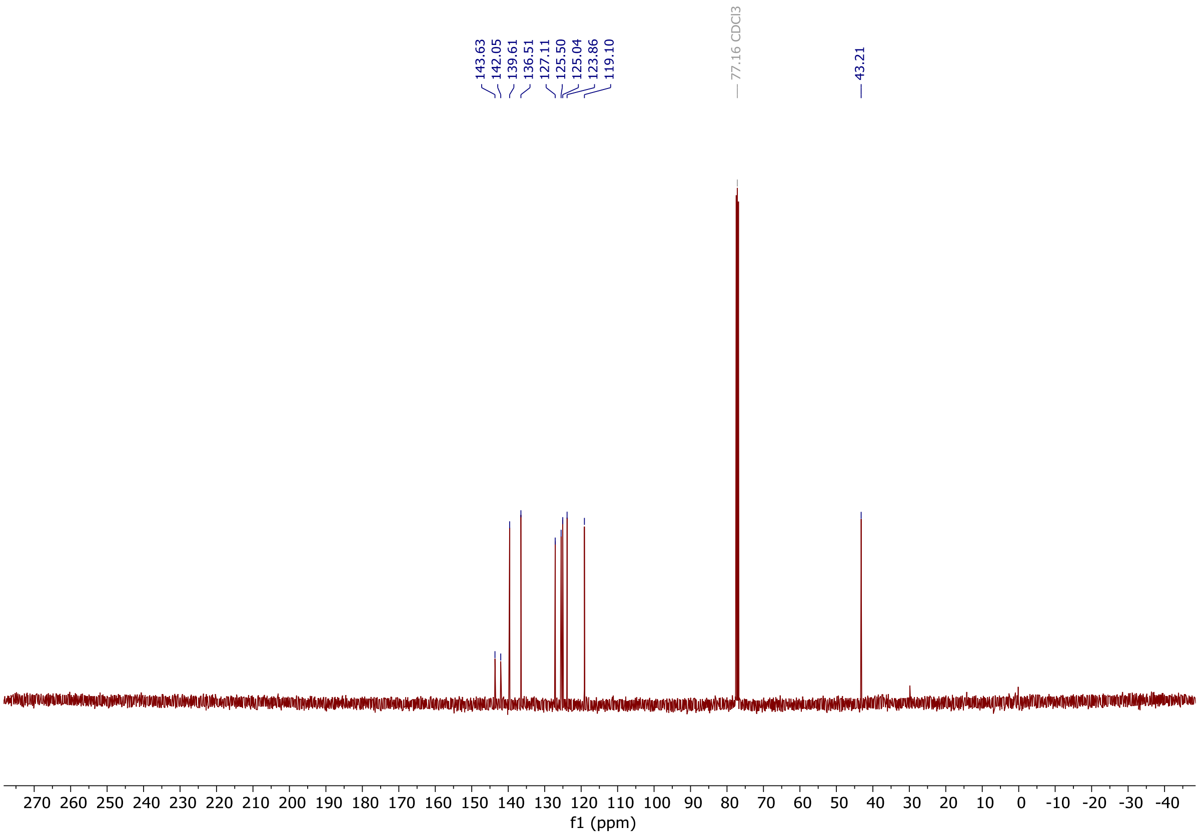

^{1}H NMR of 38 ($CDCl_3$, 300 MHz)

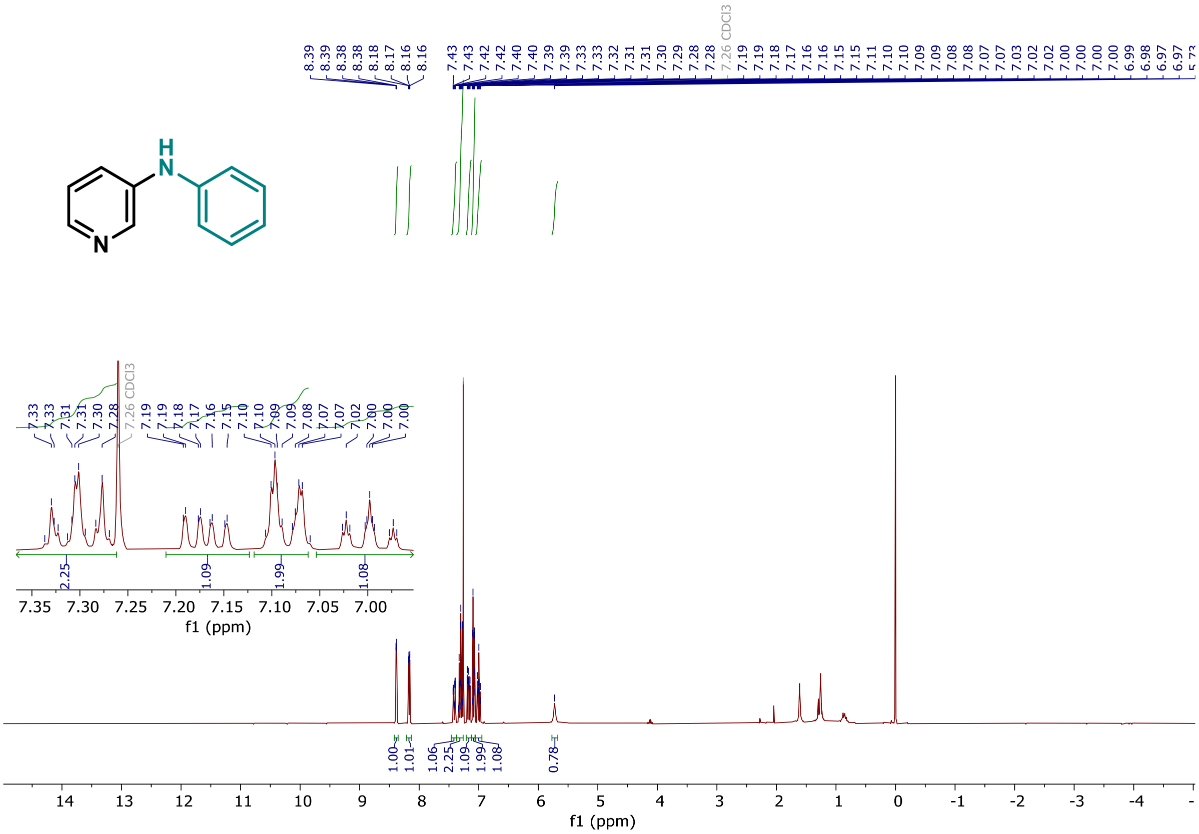

^{13}C NMR of 38 ($CDCl_3$, 101 MHz)

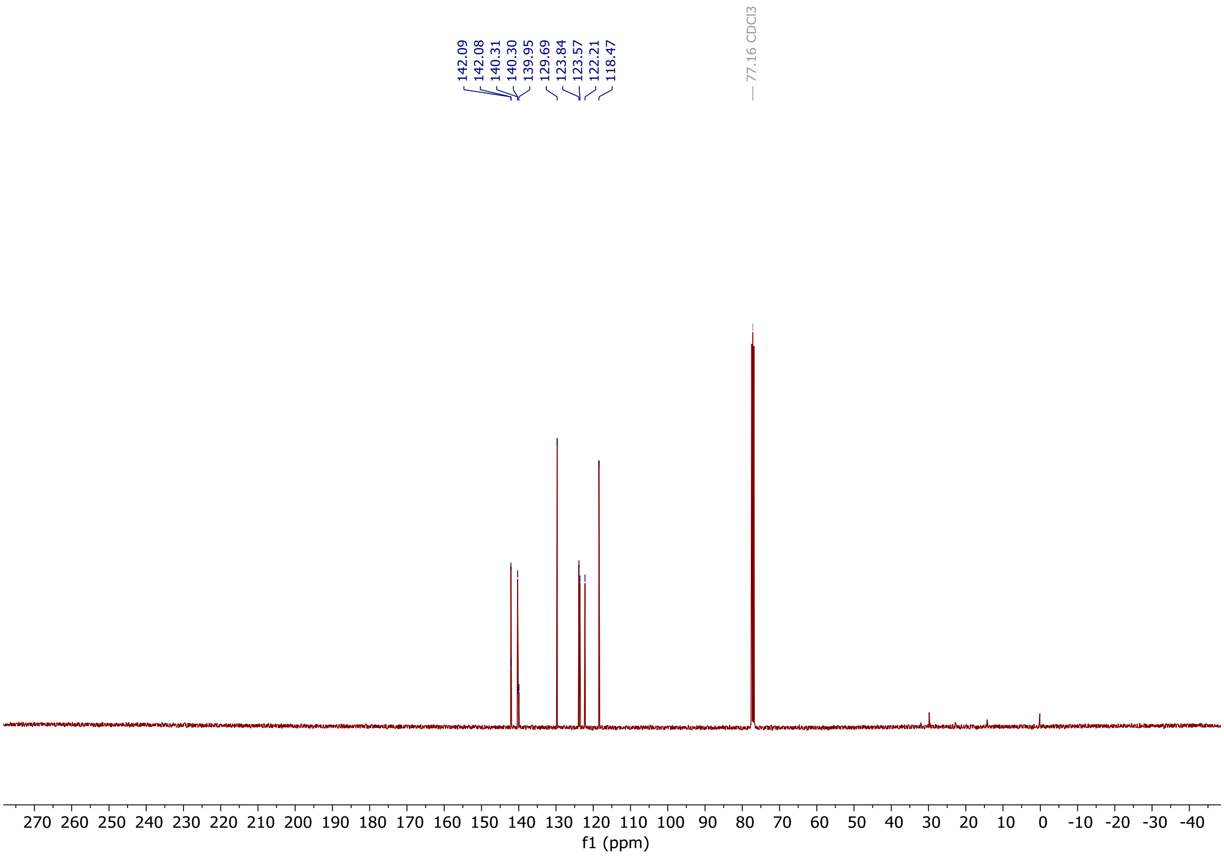

^{1}H NMR of 39 ($CDCl_3$, 400 MHz)

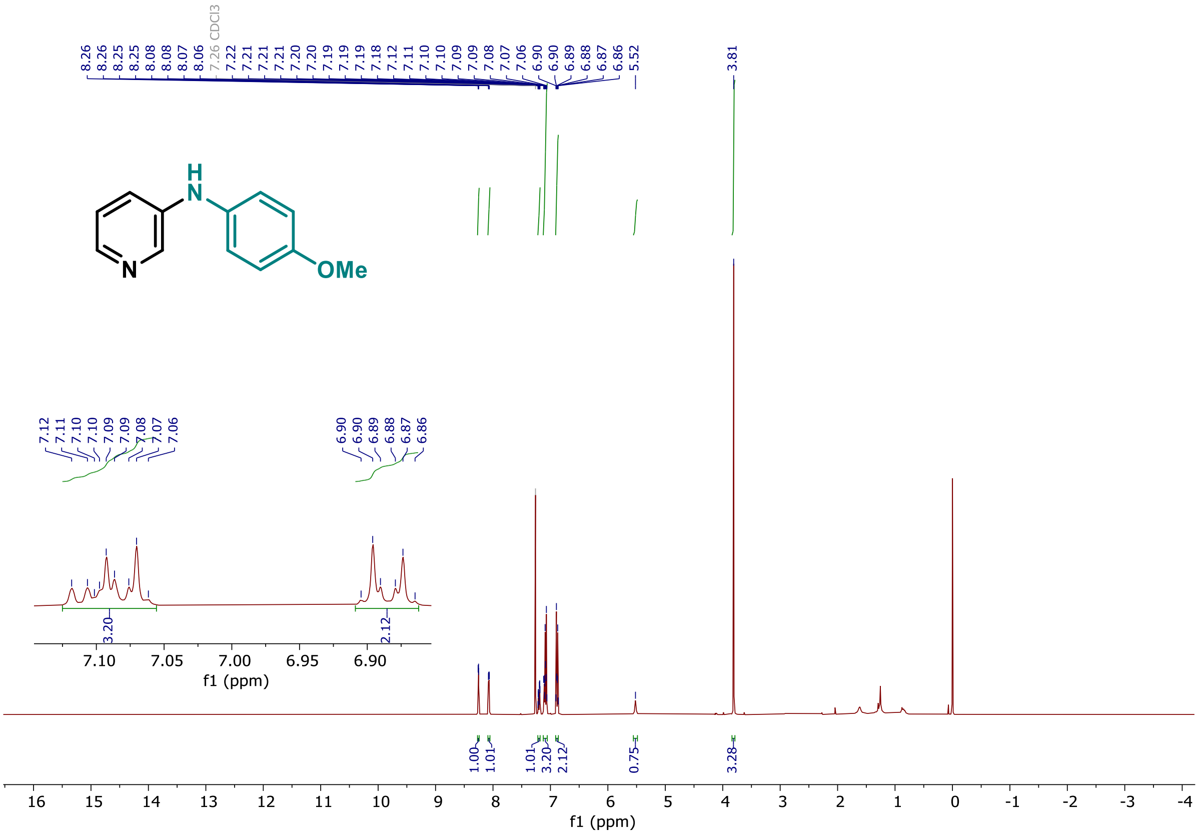

^{13}C NMR of 39 ($CDCl_3$, 101 MHz)

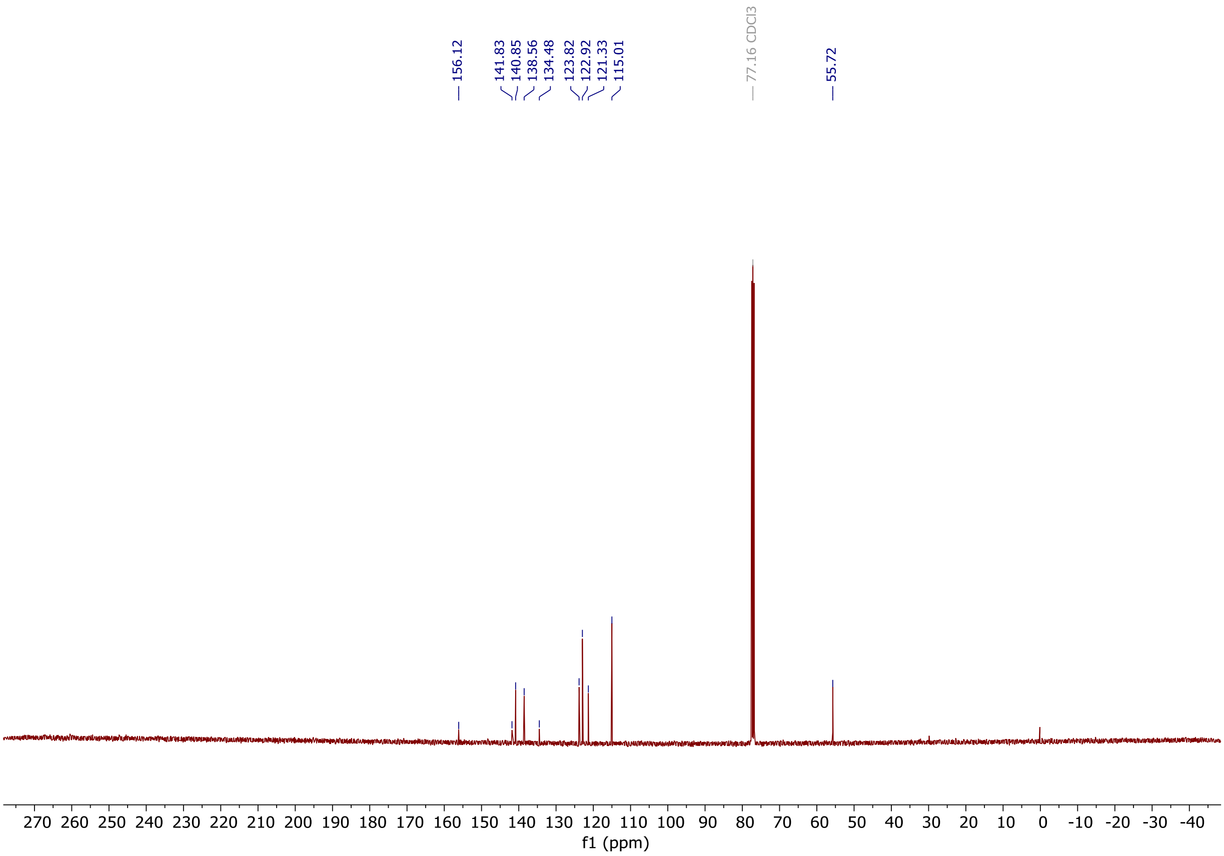

^{1}H NMR of 40 ($CDCl_3$, 400 MHz)

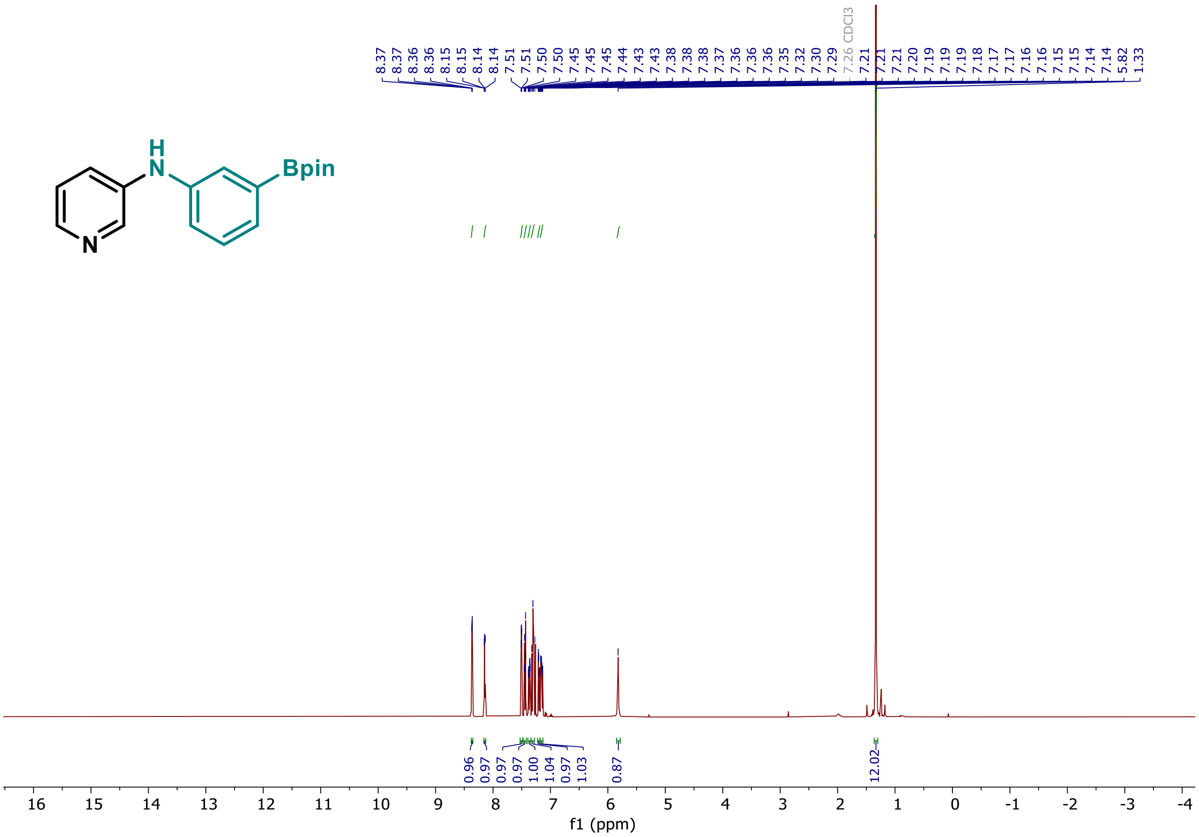

^{13}C NMR of 40 ($CDCl_3$, 101 MHz)

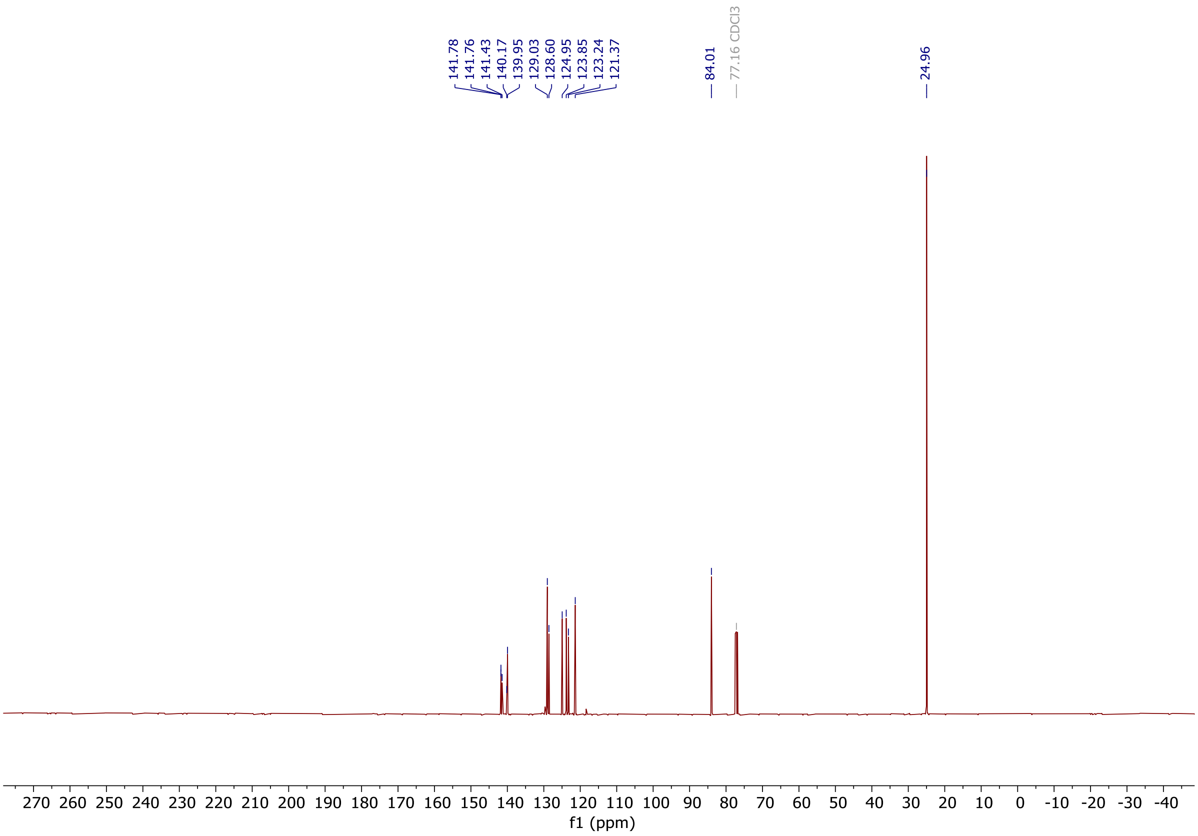

^{11}B NMR of 40 ($CDCl_3$, 128 MHz)

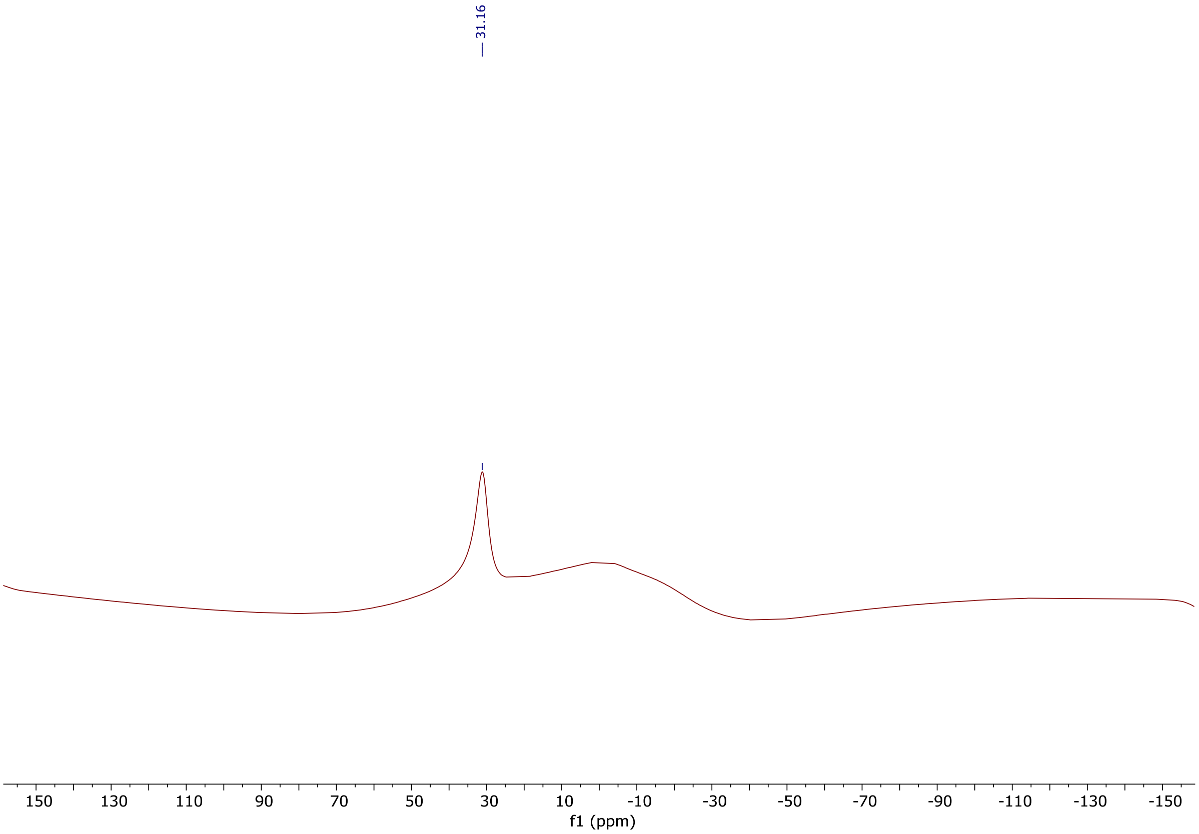

^{1}H NMR of 41 (DMSO-d_6, 400 MHz)

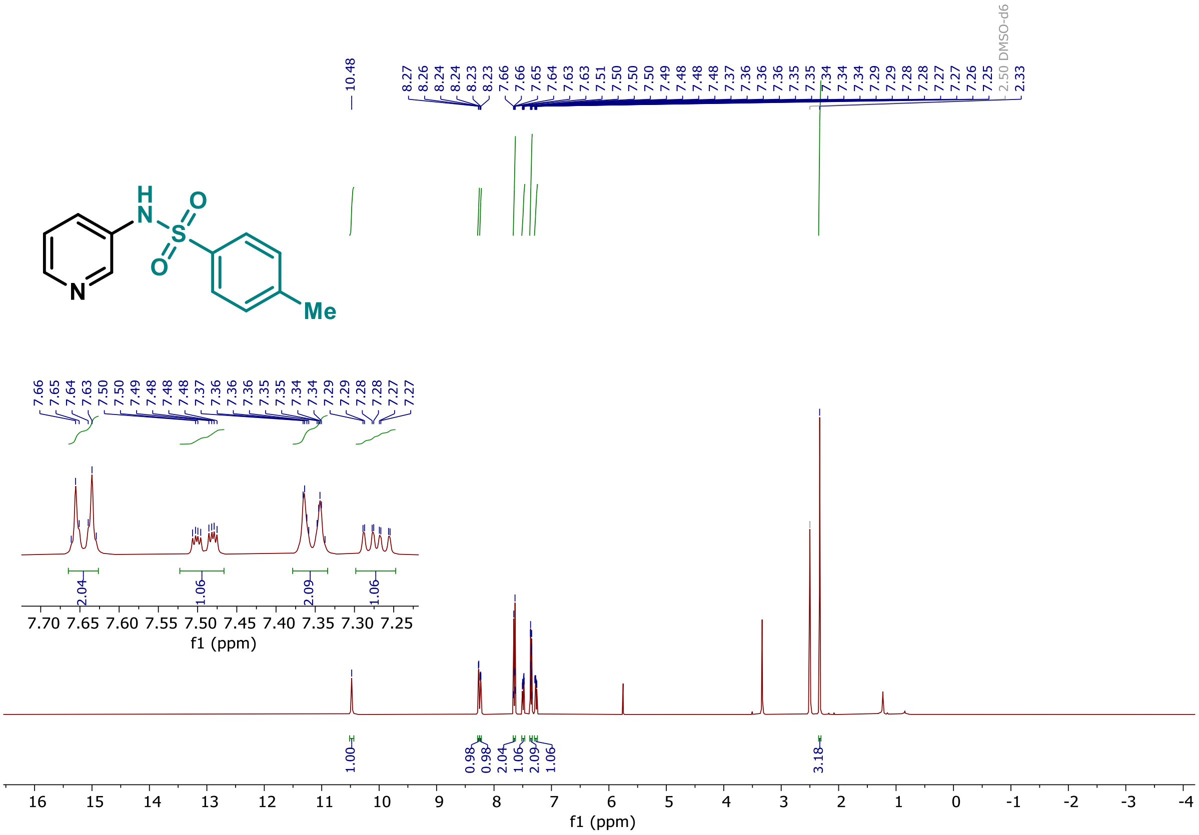

^{13}C NMR of 41 (DMSO-d_6, 101 MHz)

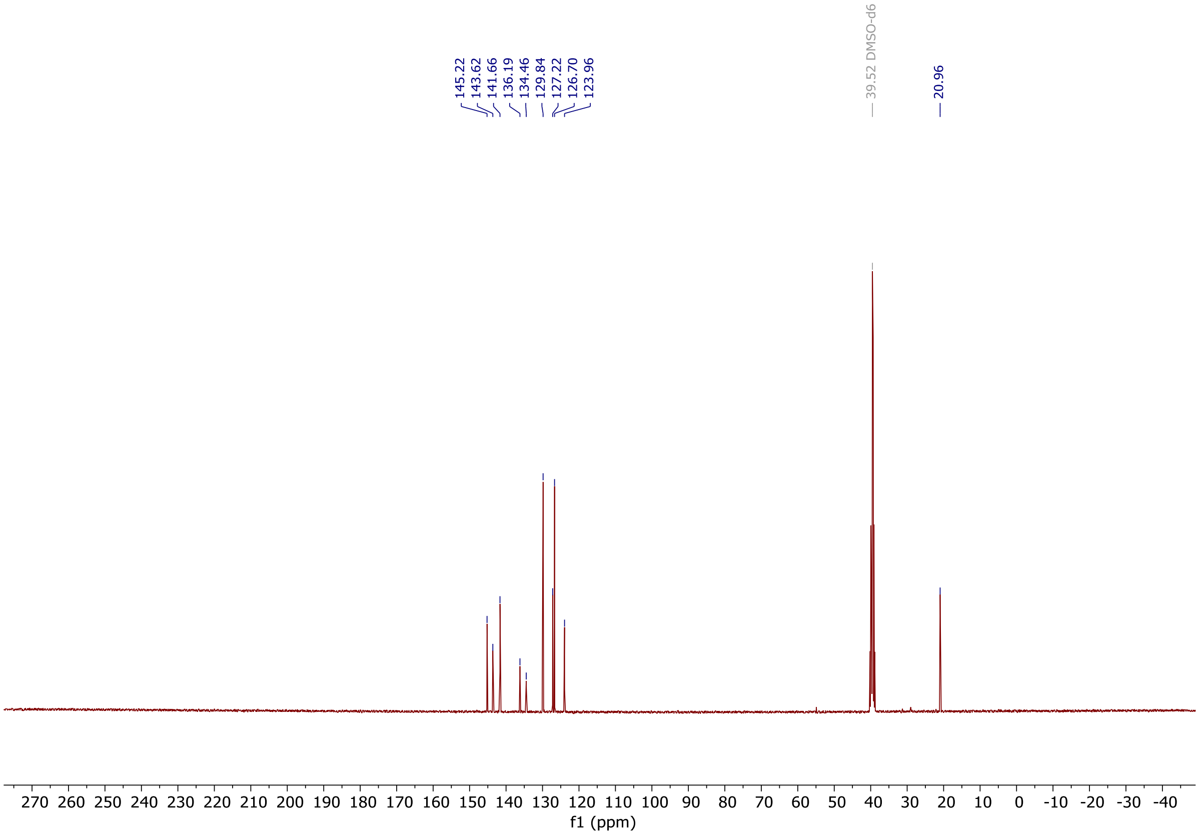

^{1}H NMR of 42 ($CDCl_3$, 400 MHz)

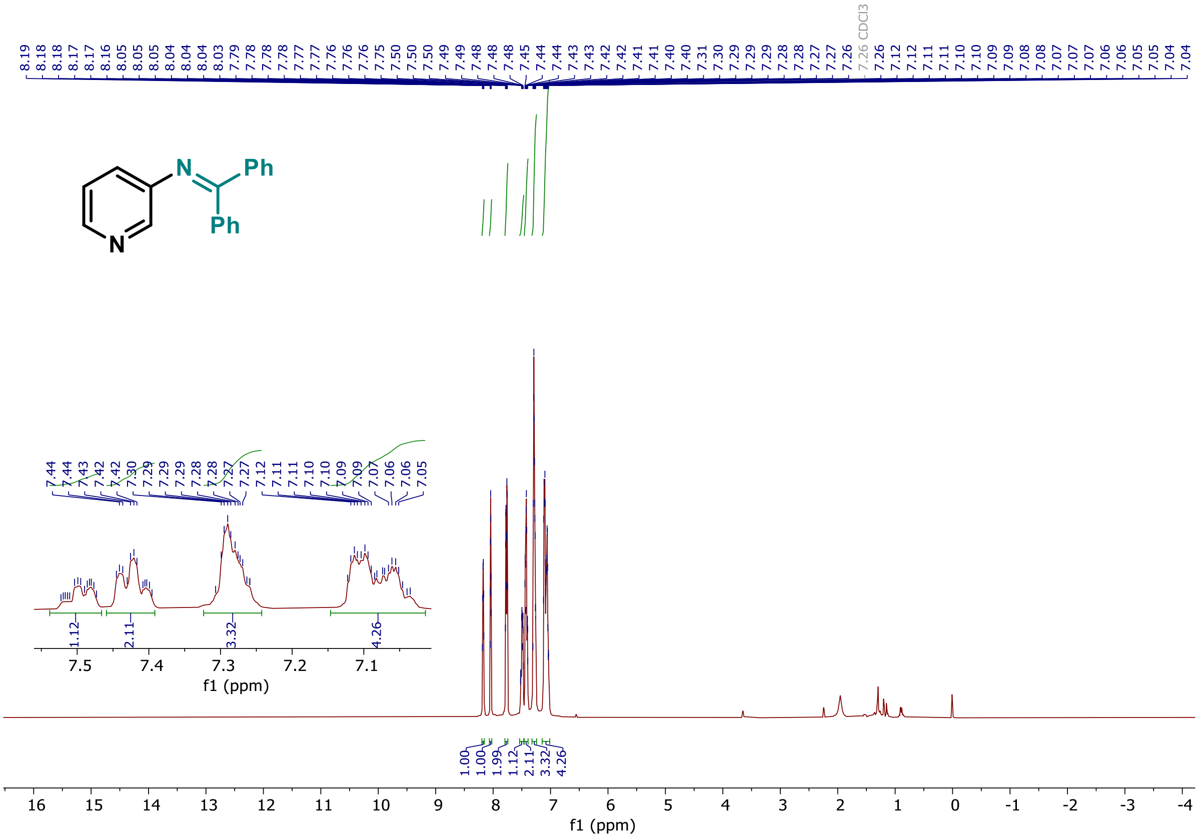

^{13}C NMR of 42 ($CDCl_3$, 101 MHz)

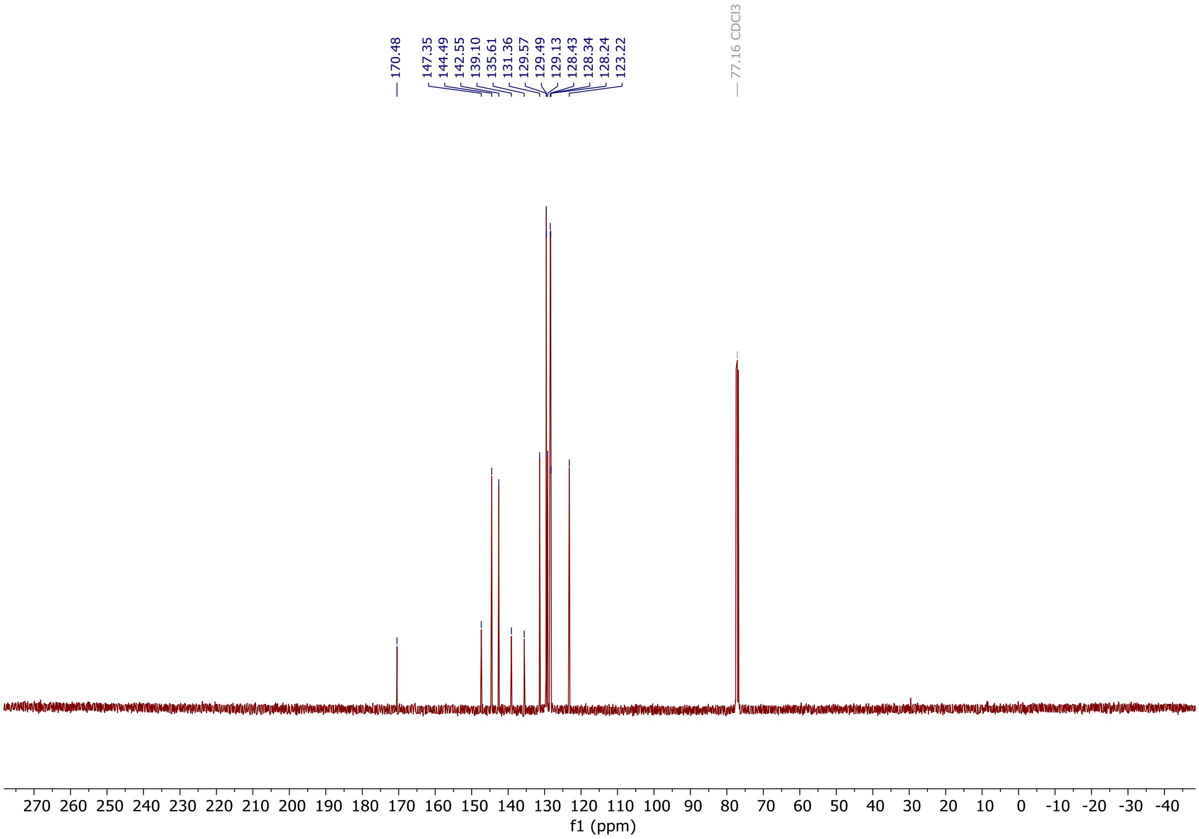

Crystallographic Data

Single crystal structure analysis of 15244

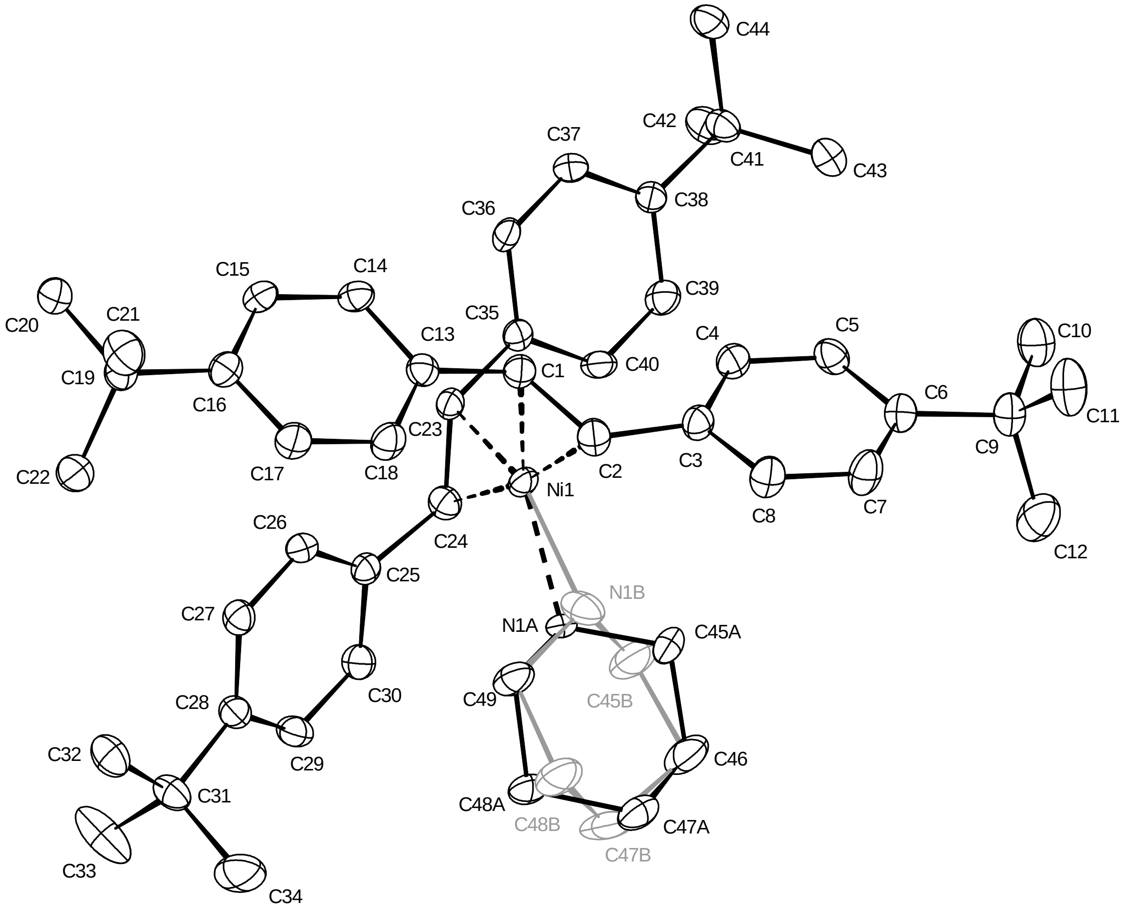

Figure S3. The molecular structure of complex **50**. H atoms have been removed for clarity, minor part of disorder is shown in grey.

X-ray Crystal Structure Analysis of complex 50: C_{49} H_{67} N Ni, M_r = 728.74 g mol^{-1}, orange plate, crystal size 0.055 x 0.044 x 0.024 mm^3, monoclinic, space group $P2_1$/n [14], a = 11.1703(4) Å, b = 32.2589(10) Å, c = 11.8214(3) Å, β = 94.326(2)°, V = 4247.6(2) Å^3, T = 100(2) K, Z = 4, D_{calc} = 1.140 g·cm^3, λ = 0.71073 Å, $\mu(Mo\text{-}K_{\alpha})$ = 0.489 mm^{-1}, analytical absorption correction ($T_{\min}$ = 0.97173, $T_{\max}$ = 0.99323), Bruker-AXS Kappa Mach3 with APEX-II detector and IµS microfocus source, 1.262 < θ < 28.281°, 102890 measured reflections, 10531 independent reflections, 6462 reflections with $I > 2\sigma(I)$, R_{int} = 0.1179. The structure was solved by *SHELXT* and refined by full-matrix least-squares (*SHELXL*) against F^2 to R_1 = 0.0477 [$I > 2\sigma(I)$], wR_2 = 0.0964, 508 parameters and 508 restraints.

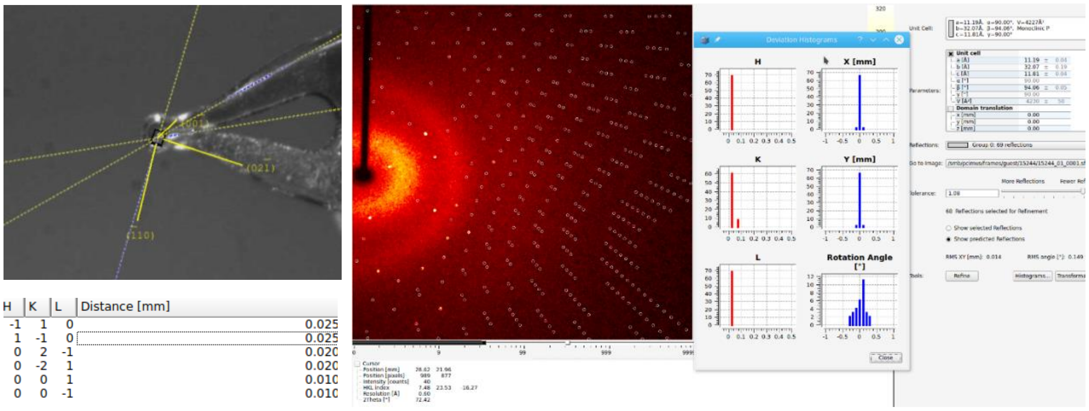

Figure S4: Crystal faces and unit cell determination of complex **50**.

INTENSITY STATISTICS FOR DATASET

Resolution	#Data	#Theory	%Complete	Redundancy	Mean I	Mean I/s	Rmerge	Rsigma
Inf - 2.83	251	251	100.0	16.79	65.57	77.11	0.0240	0.0096
2.83 - 1.87	589	589	100.0	18.01	35.97	67.89	0.0289	0.0102
1.87 - 1.48	834	834	100.0	18.22	23.22	56.73	0.0356	0.0121
1.48 - 1.29	834	834	100.0	18.16	15.15	45.00	0.0458	0.0150
1.29 - 1.16	904	904	100.0	17.55	12.53	38.50	0.0550	0.0178
1.16 - 1.08	806	806	100.0	15.82	12.04	34.13	0.0604	0.0207
1.08 - 1.01	926	926	100.0	12.21	10.47	26.92	0.0689	0.0274
1.01 - 0.96	825	825	100.0	10.36	8.43	20.57	0.0836	0.0360
0.96 - 0.92	802	802	100.0	9.11	6.40	15.79	0.1012	0.0477
0.92 - 0.88	951	951	100.0	8.19	5.29	12.76	0.1219	0.0608
0.88 - 0.85	824	824	100.0	7.74	4.66	10.67	0.1362	0.0715
0.85 - 0.83	649	649	100.0	7.55	4.74	10.39	0.1334	0.0731
0.83 - 0.80	1035	1035	100.0	7.24	4.76	10.02	0.1435	0.0779
0.80 - 0.78	826	826	100.0	7.06	4.48	9.34	0.1562	0.0857
0.78 - 0.76	832	832	100.0	6.78	4.49	8.99	0.1609	0.0904
0.76 - 0.74	1007	1007	100.0	6.61	3.78	7.62	0.1890	0.1083
0.74 - 0.73	531	531	100.0	6.47	3.57	7.15	0.2047	0.1180
0.73 - 0.71	1160	1160	100.0	6.29	3.06	6.03	0.2267	0.1402
0.71 - 0.70	630	630	100.0	6.10	2.94	5.60	0.2454	0.1510
0.70 - 0.69	620	620	100.0	5.94	2.67	5.05	0.2638	0.1687
0.69 - 0.68	839	1028	81.6	3.33	2.27	3.58	0.2749	0.2844
0.78 - 0.68	5619	5808	96.7	5.85	3.27	6.34	0.2100	0.1397
Inf - 0.68	16675	16864	98.9	9.92	9.08	20.53	0.0598	0.0399

The piperidine is disordered over two positions. The occupancy was refined with fixed values 70:30% for the two parts. ISOR instruction was applied to the two N atoms. The position of the H atom at the N could be found in the residual electron density map.

Complete .cif-data of the compound are avaliable under the CCDC number **CCDC-2312434**.

Table 1. . Crystal data and structure refinement of complex **50**.

Identification code	15244
Empirical formula	$C_{49} H_{67} N Ni$

Color	orange	
Formula weight	728.74 g·mol^{-1}	
Temperature	100(2) K	
Wavelength	0.71073 Å	
Crystal system	Monoclinic	
Space group	P 1 21/n 1, (no. 14)	
Unit cell dimensions	a = 11.1703(4) Å	α= 90°.
	b = 32.2589(10) Å	β= 94.326(2)°.
	c = 11.8214(3) Å	γ = 90°.
Volume	4247.6(2) Å^3	
Z	4	
Density (calculated)	1.140 Mg·m^{-3}	
Absorption coefficient	0.489 mm^{-1}	
F(000)	1584 e	
Crystal size	0.055 x 0.044 x 0.024 mm^3	
θ range for data collection	1.262 to 28.281°.	
Index ranges	$-14 \le h \le 14$, $-43 \le k \le 43$, $-15 \le l \le 15$	
Reflections collected	102890	
Independent reflections	10531 [R_{int} = 0.1179]	
Reflections with I>2σ(I)	6462	
Completeness to θ = 25.242°	100.0 %	
Absorption correction	Gaussian	
Max. and min. transmission	0.99323 and 0.97173	
Refinement method	Full-matrix least-squares on F^2	
Data / restraints / parameters	10531 / 12 / 508	
Goodness-of-fit on F^2	0.998	
Final R indices [I>2σ(I)]	R_1 = 0.0477	wR^2 = 0.0802
R indices (all data)	R_1 = 0.1090	wR^2 = 0.0964
Extinction coefficient	n/a	
Largest diff. peak and hole	0.287 and -0.368 e·Å^{-3}	

Table 2. Bond lengths [Å] and angles [°] of complex **50**.

C(1)-N(1)	1.379(4)	C(1)-N(2)	1.380(4)
C(1)-Ru(1)	2.004(3)	C(2)-H(2)	0.9500

C(2)-C(3)	1.342(4)	C(2)-N(1)	1.385(4)
C(3)-H(3)	0.9500	C(3)-N(2)	1.390(4)
C(4)-C(5)	1.399(4)	C(4)-C(9)	1.397(4)
C(4)-N(1)	1.449(4)	C(5)-C(6)	1.397(4)
C(5)-C(10)	1.520(5)	C(6)-H(6)	0.9500
C(6)-C(7)	1.381(5)	C(7)-H(7)	0.9500
C(7)-C(8)	1.378(5)	C(8)-H(8)	0.9500
C(8)-C(9)	1.388(4)	C(9)-C(13A)	1.47(2)
C(9)-C(13B)	1.64(3)	C(10)-H(10)	1.0000
C(10)-C(11)	1.537(5)	C(10)-C(12)	1.530(5)
C(11)-H(11A)	0.9800	C(11)-H(11B)	0.9800
C(11)-H(11C)	0.9800	C(12)-H(12A)	0.9800
C(12)-H(12B)	0.9800	C(12)-H(12C)	0.9800
C(16)-C(17)	1.398(4)	C(16)-C(21)	1.401(4)
C(16)-N(2)	1.439(4)	C(17)-C(18)	1.394(4)
C(17)-C(22)	1.518(4)	C(18)-H(18)	0.9500
C(18)-C(19)	1.379(5)	C(19)-H(19)	0.9500
C(19)-C(20)	1.389(5)	C(20)-H(20)	0.9500
C(20)-C(21)	1.389(4)	C(21)-C(25)	1.521(4)
C(22)-H(22)	1.0000	C(22)-C(23)	1.532(5)
C(22)-C(24)	1.527(5)	C(23)-H(23A)	0.9800
C(23)-H(23B)	0.9800	C(23)-H(23C)	0.9800
C(24)-H(24A)	0.9800	C(24)-H(24B)	0.9800
C(24)-H(24C)	0.9800	C(25)-H(25)	1.0000
C(25)-C(26)	1.530(5)	C(25)-C(27)	1.523(5)
C(26)-H(26A)	0.9800	C(26)-H(26B)	0.9800
C(26)-H(26C)	0.9800	C(27)-H(27A)	0.9800
C(27)-H(27B)	0.9800	C(27)-H(27C)	0.9800
C(28)-C(28)#1	1.344(6)	C(28)-H(28)	0.9500
C(28)-N(3)	1.388(4)	C(29)-H(29)	0.9500
C(29)-N(3)	1.331(3)	C(29)-N(3)#1	1.331(3)
C(30)-C(31)	1.395(5)	C(30)-C(35)	1.400(4)
C(30)-N(3)	1.454(4)	C(31)-C(32)	1.395(4)
C(31)-C(36)	1.519(4)	C(32)-H(32)	0.9500
C(32)-C(33)	1.382(4)	C(33)-H(33)	0.9500
C(33)-C(34)	1.388(5)	C(34)-H(34)	0.9500

C(34)-C(35)	1.392(4)	C(35)-C(39)	1.516(4)
C(36)-C(37C)	1.500(14)	C(36)-C(38C)	1.680(12)
C(36)-C(37B)	1.519(14)	C(36)-C(38B)	1.457(13)
C(36)-C(38A)	1.530(13)	C(36)-C(37A)	1.589(10)
C(39)-H(39)	1.0000	C(39)-C(40)	1.543(5)
C(39)-C(41)	1.531(4)	C(40)-H(40A)	0.9800
C(40)-H(40B)	0.9800	C(40)-H(40C)	0.9800
C(41)-H(41A)	0.9800	C(41)-H(41B)	0.9800
C(41)-H(41C)	0.9800	C(42)-H(42A)	1.04(5)
C(42)-H(42B)	0.91(5)	C(42)-Cl(4A)	1.819(18)
C(42)-Cl(5A)	1.59(4)	C(42)-Cl(4B)	1.724(9)
C(42)-Cl(5B)	1.827(16)	Cl(1)-Ru(1)	2.4289(7)
Cl(1)-Ru(1)#1	2.5300(7)	Cl(2)-Ru(1)	2.3938(7)
Cl(3)-Ru(1)	2.4118(7)	Cl(3)-Ru(1)#1	2.4118(7)
C(13A)-H(13A)	1.0000	C(13A)-C(14A)	1.54(2)
C(13A)-C(15A)	1.511(14)	C(14A)-H(14A)	0.9800
C(14A)-H(14B)	0.9800	C(14A)-H(14C)	0.9800
C(15A)-H(15A)	0.9800	C(15A)-H(15B)	0.9800
C(15A)-H(15C)	0.9800	C(43A)-H(43A)	0.9900
C(43A)-H(43B)	0.9900	C(43A)-Cl(6A)	1.772(7)
C(43A)-Cl(7A)	1.759(6)	C(37C)-H(37A)	0.9800
C(37C)-H(37B)	0.9800	C(37C)-H(37C)	0.9800
C(38C)-H(38A)	0.9800	C(38C)-H(38B)	0.9800
C(38C)-H(38C)	0.9800	C(43B)-H(43C)	0.9900
C(43B)-H(43D)	0.9900	C(43B)-Cl(6B)	1.770(16)
C(43B)-Cl(7B)	1.737(15)	C(13B)-H(13B)	1.0000
C(13B)-C(14B)	1.52(3)	C(13B)-C(15B)	1.52(2)
C(14B)-H(14D)	0.9800	C(14B)-H(14E)	0.9800
C(14B)-H(14F)	0.9800	C(15B)-H(15D)	0.9800
C(15B)-H(15E)	0.9800	C(15B)-H(15F)	0.9800
C(37B)-H(37D)	0.9800	C(37B)-H(37E)	0.9800
C(37B)-H(37F)	0.9800	C(38B)-H(38D)	0.9800
C(38B)-H(38E)	0.9800	C(38B)-H(38F)	0.9800
C(38A)-H(38G)	0.9800	C(38A)-H(38H)	0.9800
C(38A)-H(38I)	0.9800	C(37A)-H(37G)	0.9800
C(37A)-H(37H)	0.9800	C(37A)-H(37I)	0.9800

C(44A)-H(44A)	0.9900	C(44A)-H(44B)	0.9900
C(44A)-Cl(8A)	1.769(11)	C(44A)-Cl(9A)	1.745(11)
Ni(1)-C(1)	1.999(2)	Ni(1)-C(2)	2.010(2)
Ni(1)-C(23)	1.9983(19)	Ni(1)-C(24)	2.022(2)
Ni(1)-N(1A)	2.043(6)	Ni(1)-N(1B)	1.940(17)
C(1)-H(1)	1.0000	C(1)-C(2)	1.405(3)
C(1)-C(13)	1.468(3)	C(2)-H(2)	1.0000
C(2)-C(3)	1.472(3)	C(3)-C(4)	1.392(3)
C(3)-C(8)	1.394(3)	C(4)-H(4)	0.9500
C(4)-C(5)	1.383(3)	C(5)-H(5)	0.9500
C(5)-C(6)	1.395(3)	C(6)-C(7)	1.386(3)
C(6)-C(9)	1.529(3)	C(7)-H(7)	0.9500
C(7)-C(8)	1.377(3)	C(8)-H(8)	0.9500
C(9)-C(10)	1.524(3)	C(9)-C(11)	1.528(3)
C(9)-C(12)	1.538(3)	C(10)-H(10A)	0.9800
C(10)-H(10B)	0.9800	C(10)-H(10C)	0.9800
C(11)-H(11A)	0.9800	C(11)-H(11B)	0.9800
C(11)-H(11C)	0.9800	C(12)-H(12A)	0.9800
C(12)-H(12B)	0.9800	C(12)-H(12C)	0.9800
C(13)-C(14)	1.398(3)	C(13)-C(18)	1.398(3)
C(14)-H(14)	0.9500	C(14)-C(15)	1.383(3)
C(15)-H(15)	0.9500	C(15)-C(16)	1.388(3)
C(16)-C(17)	1.398(3)	C(16)-C(19)	1.530(3)
C(17)-H(17)	0.9500	C(17)-C(18)	1.378(3)
C(18)-H(18)	0.9500	C(19)-C(20)	1.534(3)
C(19)-C(21)	1.535(3)	C(19)-C(22)	1.538(3)
C(20)-H(20A)	0.9800	C(20)-H(20B)	0.9800
C(20)-H(20C)	0.9800	C(21)-H(21A)	0.9800
C(21)-H(21B)	0.9800	C(21)-H(21C)	0.9800
C(22)-H(22A)	0.9800	C(22)-H(22B)	0.9800
C(22)-H(22C)	0.9800	C(23)-H(23)	1.0000
C(23)-C(24)	1.403(3)	C(23)-C(35)	1.472(3)
C(24)-H(24)	1.0000	C(24)-C(25)	1.466(3)
C(25)-C(26)	1.398(3)	C(25)-C(30)	1.401(3)
C(26)-H(26)	0.9500	C(26)-C(27)	1.379(3)

C(27)-H(27)	0.9500	C(27)-C(28)	1.396(3)
C(28)-C(29)	1.388(3)	C(28)-C(31)	1.530(3)
C(29)-H(29)	0.9500	C(29)-C(30)	1.386(3)
C(30)-H(30)	0.9500	C(31)-C(32)	1.523(3)
C(31)-C(33)	1.526(3)	C(31)-C(34)	1.539(3)
C(32)-H(32A)	0.9800	C(32)-H(32B)	0.9800
C(32)-H(32C)	0.9800	C(33)-H(33A)	0.9800
C(33)-H(33B)	0.9800	C(33)-H(33C)	0.9800
C(34)-H(34A)	0.9800	C(34)-H(34B)	0.9800
C(34)-H(34C)	0.9800	C(35)-C(36)	1.391(3)
C(35)-C(40)	1.400(3)	C(36)-H(36)	0.9500
C(36)-C(37)	1.382(3)	C(37)-H(37)	0.9500
C(37)-C(38)	1.387(3)	C(38)-C(39)	1.396(3)
C(38)-C(41)	1.531(3)	C(39)-H(39)	0.9500
C(39)-C(40)	1.379(3)	C(40)-H(40)	0.9500
C(41)-C(42)	1.537(3)	C(41)-C(43)	1.538(3)
C(41)-C(44)	1.530(3)	C(42)-H(42A)	0.9800
C(42)-H(42B)	0.9800	C(42)-H(42C)	0.9800
C(43)-H(43A)	0.9800	C(43)-H(43B)	0.9800
C(43)-H(43C)	0.9800	C(44)-H(44A)	0.9800
C(44)-H(44B)	0.9800	C(44)-H(44C)	0.9800
C(46)-H(46C)	0.9900	C(46)-H(46D)	0.9900
C(46)-H(46A)	0.9900	C(46)-H(46B)	0.9900
C(46)-C(45A)	1.532(4)	C(46)-C(47A)	1.521(6)
C(46)-C(45B)	1.570(9)	C(46)-C(47B)	1.567(14)
C(49)-H(49A)	0.9900	C(49)-H(49B)	0.9900
C(49)-H(49C)	0.9900	C(49)-H(49D)	0.9900
C(49)-N(1A)	1.464(7)	C(49)-C(48A)	1.532(5)
C(49)-N(1B)	1.563(16)	C(49)-C(48B)	1.523(12)
N(1A)-H(1A)	1.0000	N(1A)-C(45A)	1.489(7)
C(45A)-H(45A)	0.9900	C(45A)-H(45B)	0.9900
C(47A)-H(47A)	0.9900	C(47A)-H(47B)	0.9900
C(47A)-C(48A)	1.545(7)	C(48A)-H(48A)	0.9900
C(48A)-H(48B)	0.9900	N(1B)-H(1B)	1.0000
N(1B)-C(45B)	1.414(19)	C(45B)-H(45C)	0.9900
C(45B)-H(45D)	0.9900	C(47B)-H(47C)	0.9900

C(47B)-H(47D)	0.9900	C(47B)-C(48B)	1.43(2)
C(48B)-H(48C)	0.9900	C(48B)-H(48D)	0.9900
C(1)-Ni(1)-C(2)	41.02(8)	C(1)-Ni(1)-C(24)	129.72(8)
C(1)-Ni(1)-N(1A)	137.26(16)	C(2)-Ni(1)-C(24)	170.16(9)
C(2)-Ni(1)-N(1A)	99.51(15)	C(23)-Ni(1)-C(1)	91.30(8)
C(23)-Ni(1)-C(2)	129.37(9)	C(23)-Ni(1)-C(24)	40.83(8)
C(23)-Ni(1)-N(1A)	130.76(15)	C(24)-Ni(1)-N(1A)	90.32(15)
N(1B)-Ni(1)-C(1)	125.1(4)	N(1B)-Ni(1)-C(2)	86.0(3)
N(1B)-Ni(1)-C(23)	143.6(4)	N(1B)-Ni(1)-C(24)	103.8(3)
Ni(1)-C(1)-H(1)	115.2	C(2)-C(1)-Ni(1)	69.89(13)
C(2)-C(1)-H(1)	115.2	C(2)-C(1)-C(13)	123.94(19)
C(13)-C(1)-Ni(1)	108.08(15)	C(13)-C(1)-H(1)	115.2
Ni(1)-C(2)-H(2)	114.9	C(1)-C(2)-Ni(1)	69.09(12)
C(1)-C(2)-H(2)	114.9	C(1)-C(2)-C(3)	124.33(19)
C(3)-C(2)-Ni(1)	109.60(15)	C(3)-C(2)-H(2)	114.9
C(4)-C(3)-C(2)	124.46(19)	C(4)-C(3)-C(8)	116.8(2)
C(8)-C(3)-C(2)	118.69(19)	C(3)-C(4)-H(4)	119.4
C(5)-C(4)-C(3)	121.15(19)	C(5)-C(4)-H(4)	119.4
C(4)-C(5)-H(5)	119.0	C(4)-C(5)-C(6)	121.9(2)
C(6)-C(5)-H(5)	119.0	C(5)-C(6)-C(9)	123.69(19)
C(7)-C(6)-C(5)	116.6(2)	C(7)-C(6)-C(9)	119.66(19)
C(6)-C(7)-H(7)	119.1	C(8)-C(7)-C(6)	121.7(2)
C(8)-C(7)-H(7)	119.1	C(3)-C(8)-H(8)	119.1
C(7)-C(8)-C(3)	121.8(2)	C(7)-C(8)-H(8)	119.1
C(6)-C(9)-C(12)	109.14(19)	C(10)-C(9)-C(6)	112.87(18)
C(10)-C(9)-C(11)	107.7(2)	C(10)-C(9)-C(12)	108.73(19)
C(11)-C(9)-C(6)	109.29(19)	C(11)-C(9)-C(12)	109.0(2)
C(9)-C(10)-H(10A)	109.5	C(9)-C(10)-H(10B)	109.5
C(9)-C(10)-H(10C)	109.5	H(10A)-C(10)-H(10B)	109.5
H(10A)-C(10)-H(10C)	109.5	H(10B)-C(10)-H(10C)	109.5
C(9)-C(11)-H(11A)	109.5	C(9)-C(11)-H(11B)	109.5
C(9)-C(11)-H(11C)	109.5	H(11A)-C(11)-H(11B)	109.5
H(11A)-C(11)-H(11C)	109.5	H(11B)-C(11)-H(11C)	109.5
C(9)-C(12)-H(12A)	109.5	C(9)-C(12)-H(12B)	109.5
C(9)-C(12)-H(12C)	109.5	H(12A)-C(12)-H(12B)	109.5

H(12A)-C(12)-H(12C)	109.5	H(12B)-C(12)-H(12C)	109.5
C(14)-C(13)-C(1)	119.72(19)	C(18)-C(13)-C(1)	124.49(19)
C(18)-C(13)-C(14)	115.70(19)	C(13)-C(14)-H(14)	118.8
C(15)-C(14)-C(13)	122.4(2)	C(15)-C(14)-H(14)	118.8
C(14)-C(15)-H(15)	119.1	C(14)-C(15)-C(16)	121.7(2)
C(16)-C(15)-H(15)	119.1	C(15)-C(16)-C(17)	116.0(2)
C(15)-C(16)-C(19)	123.74(19)	C(17)-C(16)-C(19)	120.26(19)
C(16)-C(17)-H(17)	118.8	C(18)-C(17)-C(16)	122.5(2)
C(18)-C(17)-H(17)	118.8	C(13)-C(18)-H(18)	119.2
C(17)-C(18)-C(13)	121.6(2)	C(17)-C(18)-H(18)	119.2
C(16)-C(19)-C(20)	112.37(18)	C(16)-C(19)-C(21)	108.51(18)
C(16)-C(19)-C(22)	110.27(18)	C(20)-C(19)-C(21)	108.61(19)
C(20)-C(19)-C(22)	107.53(18)	C(21)-C(19)-C(22)	109.51(19)
C(19)-C(20)-H(20A)	109.5	C(19)-C(20)-H(20B)	109.5
C(19)-C(20)-H(20C)	109.5	H(20A)-C(20)-H(20B)	109.5
H(20A)-C(20)-H(20C)	109.5	H(20B)-C(20)-H(20C)	109.5
C(19)-C(21)-H(21A)	109.5	C(19)-C(21)-H(21B)	109.5
C(19)-C(21)-H(21C)	109.5	H(21A)-C(21)-H(21B)	109.5
H(21A)-C(21)-H(21C)	109.5	H(21B)-C(21)-H(21C)	109.5
C(19)-C(22)-H(22A)	109.5	C(19)-C(22)-H(22B)	109.5
C(19)-C(22)-H(22C)	109.5	H(22A)-C(22)-H(22B)	109.5
H(22A)-C(22)-H(22C)	109.5	H(22B)-C(22)-H(22C)	109.5
Ni(1)-C(23)-H(23)	115.1	C(24)-C(23)-Ni(1)	70.50(11)
C(24)-C(23)-H(23)	115.1	C(24)-C(23)-C(35)	123.36(19)
C(35)-C(23)-Ni(1)	109.10(13)	C(35)-C(23)-H(23)	115.1
Ni(1)-C(24)-H(24)	115.9	C(23)-C(24)-Ni(1)	68.67(11)
C(23)-C(24)-H(24)	115.9	C(23)-C(24)-C(25)	124.6(2)
C(25)-C(24)-Ni(1)	104.13(14)	C(25)-C(24)-H(24)	115.9
C(26)-C(25)-C(24)	123.2(2)	C(26)-C(25)-C(30)	116.39(19)
C(30)-C(25)-C(24)	120.04(19)	C(25)-C(26)-H(26)	119.2
C(27)-C(26)-C(25)	121.6(2)	C(27)-C(26)-H(26)	119.2
C(26)-C(27)-H(27)	119.0	C(26)-C(27)-C(28)	122.0(2)
C(28)-C(27)-H(27)	119.0	C(27)-C(28)-C(31)	122.67(19)
C(29)-C(28)-C(27)	116.4(2)	C(29)-C(28)-C(31)	120.9(2)
C(28)-C(29)-H(29)	119.0	C(30)-C(29)-C(28)	122.0(2)
C(30)-C(29)-H(29)	119.0	C(25)-C(30)-H(30)	119.3

C(29)-C(30)-C(25)	121.4(2)	C(29)-C(30)-H(30)	119.3
C(28)-C(31)-C(34)	108.28(19)	C(32)-C(31)-C(28)	112.47(19)
C(32)-C(31)-C(33)	108.2(2)	C(32)-C(31)-C(34)	108.0(2)
C(33)-C(31)-C(28)	110.01(19)	C(33)-C(31)-C(34)	109.9(2)
C(31)-C(32)-H(32A)	109.5	C(31)-C(32)-H(32B)	109.5
C(31)-C(32)-H(32C)	109.5	H(32A)-C(32)-H(32B)	109.5
H(32A)-C(32)-H(32C)	109.5	H(32B)-C(32)-H(32C)	109.5
C(31)-C(33)-H(33A)	109.5	C(31)-C(33)-H(33B)	109.5
C(31)-C(33)-H(33C)	109.5	H(33A)-C(33)-H(33B)	109.5
H(33A)-C(33)-H(33C)	109.5	H(33B)-C(33)-H(33C)	109.5
C(31)-C(34)-H(34A)	109.5	C(31)-C(34)-H(34B)	109.5
C(31)-C(34)-H(34C)	109.5	H(34A)-C(34)-H(34B)	109.5
H(34A)-C(34)-H(34C)	109.5	H(34B)-C(34)-H(34C)	109.5
C(36)-C(35)-C(23)	120.57(19)	C(36)-C(35)-C(40)	116.03(19)
C(40)-C(35)-C(23)	123.35(19)	C(35)-C(36)-H(36)	118.8
C(37)-C(36)-C(35)	122.4(2)	C(37)-C(36)-H(36)	118.8
C(36)-C(37)-H(37)	119.2	C(36)-C(37)-C(38)	121.6(2)
C(38)-C(37)-H(37)	119.2	C(37)-C(38)-C(39)	116.36(19)
C(37)-C(38)-C(41)	123.33(19)	C(39)-C(38)-C(41)	120.29(19)
C(38)-C(39)-H(39)	118.9	C(40)-C(39)-C(38)	122.2(2)
C(40)-C(39)-H(39)	118.9	C(35)-C(40)-H(40)	119.3
C(39)-C(40)-C(35)	121.5(2)	C(39)-C(40)-H(40)	119.3
C(38)-C(41)-C(42)	108.75(18)	C(38)-C(41)-C(43)	110.36(17)
C(42)-C(41)-C(43)	108.75(18)	C(44)-C(41)-C(38)	111.67(18)
C(44)-C(41)-C(42)	109.14(18)	C(44)-C(41)-C(43)	108.12(18)
C(41)-C(42)-H(42A)	109.5	C(41)-C(42)-H(42B)	109.5
C(41)-C(42)-H(42C)	109.5	H(42A)-C(42)-H(42B)	109.5
H(42A)-C(42)-H(42C)	109.5	H(42B)-C(42)-H(42C)	109.5
C(41)-C(43)-H(43A)	109.5	C(41)-C(43)-H(43B)	109.5
C(41)-C(43)-H(43C)	109.5	H(43A)-C(43)-H(43B)	109.5
H(43A)-C(43)-H(43C)	109.5	H(43B)-C(43)-H(43C)	109.5
C(41)-C(44)-H(44A)	109.5	C(41)-C(44)-H(44B)	109.5
C(41)-C(44)-H(44C)	109.5	H(44A)-C(44)-H(44B)	109.5
H(44A)-C(44)-H(44C)	109.5	H(44B)-C(44)-H(44C)	109.5
H(46C)-C(46)-H(46D)	108.4	H(46A)-C(46)-H(46B)	108.2
C(45A)-C(46)-H(46A)	109.8	C(45A)-C(46)-H(46B)	109.8

C(47A)-C(46)-H(46A)	109.8	C(47A)-C(46)-H(46B)	109.8
C(47A)-C(46)-C(45A)	109.4(3)	C(45B)-C(46)-H(46C)	110.1
C(45B)-C(46)-H(46D)	110.1	C(47B)-C(46)-H(46C)	110.1
C(47B)-C(46)-H(46D)	110.1	C(47B)-C(46)-C(45B)	108.0(6)
H(49A)-C(49)-H(49B)	108.1	H(49C)-C(49)-H(49D)	107.5
N(1A)-C(49)-H(49A)	109.6	N(1A)-C(49)-H(49B)	109.6
N(1A)-C(49)-C(48A)	110.5(3)	C(48A)-C(49)-H(49A)	109.6
C(48A)-C(49)-H(49B)	109.6	N(1B)-C(49)-H(49C)	108.5
N(1B)-C(49)-H(49D)	108.5	C(48B)-C(49)-H(49C)	108.5
C(48B)-C(49)-H(49D)	108.5	C(48B)-C(49)-N(1B)	115.1(9)
Ni(1)-N(1A)-H(1A)	106.3	C(49)-N(1A)-Ni(1)	111.6(4)
C(49)-N(1A)-H(1A)	106.3	C(49)-N(1A)-C(45A)	110.6(4)
C(45A)-N(1A)-Ni(1)	115.2(3)	C(45A)-N(1A)-H(1A)	106.3
C(46)-C(45A)-H(45A)	109.8	C(46)-C(45A)-H(45B)	109.8
N(1A)-C(45A)-C(46)	109.3(3)	N(1A)-C(45A)-H(45A)	109.8
N(1A)-C(45A)-H(45B)	109.8	H(45A)-C(45A)-H(45B)	108.3
C(46)-C(47A)-H(47A)	109.9	C(46)-C(47A)-H(47B)	109.9
C(46)-C(47A)-C(48A)	109.1(3)	H(47A)-C(47A)-H(47B)	108.3
C(48A)-C(47A)-H(47A)	109.9	C(48A)-C(47A)-H(47B)	109.9
C(49)-C(48A)-C(47A)	109.1(4)	C(49)-C(48A)-H(48A)	109.9
C(49)-C(48A)-H(48B)	109.9	C(47A)-C(48A)-H(48A)	109.9
C(47A)-C(48A)-H(48B)	109.9	H(48A)-C(48A)-H(48B)	108.3
Ni(1)-N(1B)-H(1B)	103.4	C(49)-N(1B)-Ni(1)	112.5(10)
C(49)-N(1B)-H(1B)	103.4	C(45B)-N(1B)-Ni(1)	121.7(11)
C(45B)-N(1B)-C(49)	110.0(10)	C(45B)-N(1B)-H(1B)	103.4
C(46)-C(45B)-H(45C)	108.6	C(46)-C(45B)-H(45D)	108.6
N(1B)-C(45B)-C(46)	114.7(10)	N(1B)-C(45B)-H(45C)	108.6
N(1B)-C(45B)-H(45D)	108.6	H(45C)-C(45B)-H(45D)	107.6
C(46)-C(47B)-H(47C)	109.9	C(46)-C(47B)-H(47D)	109.9
H(47C)-C(47B)-H(47D)	108.3	C(48B)-C(47B)-C(46)	109.0(10)
C(48B)-C(47B)-H(47C)	109.9	C(48B)-C(47B)-H(47D)	109.9
C(49)-C(48B)-H(48C)	108.7	C(49)-C(48B)-H(48D)	108.7
C(47B)-C(48B)-C(49)	114.2(10)	C(47B)-C(48B)-H(48C)	108.7
C(47B)-C(48B)-H(48D)	108.7	H(48C)-C(48B)-H(48D)	107.6
